# Dissociating water clusters via polyhydroxy quaternized interface for enhanced water permeation in nanochannels

**DOI:** 10.1038/s41467-026-76291-z

**Published:** 2026-08-05

**Authors:** Lei Xu, Chenghai Lu, Yanyan Zhang, Fuyi Wang, Guangzhi He, Chengzhi Hu, Jiuhui Qu

**Affiliations:** 1 Key Laboratory of Environmental Aquatic Chemistry, State Key Laboratory of Regional Environment and Sustainability, Research Center for Eco-Environmental Sciences, Chinese Academy of Sciences, Beijing, China; 2 University of Chinese Academy of Sciences, Beijing, China; 3 Beijing National Laboratory for Molecular Sciences, CAS Key Laboratory of Analytical Chemistry for Living Biosystems, Institute of Chemistry, Chinese Academy of Sciences, Beijing, China

**Keywords:** Pollution remediation, Pollution remediation

## Abstract

Water clusters with distinct structures in confined nanochannels exhibit unusual transport phenomena such as fast permeation, yet how to functionalize nanochannels to regulate this process and enhance water transport remains elusive. Herein, we report a water transmembrane transport mechanism based on the dissociation of water clusters and, accordingly, develop a highly permeable nanofiltration membrane with polyhydroxy quaternized interface. The results, observed by in situ liquid time-of-flight secondary ion mass spectrometry in combination with molecular dynamics simulation, reveal that the functional groups (-OH and quaternary-N⁺) of membrane pore entrances can enthalpically/entropically favorably dissociate water clusters, i.e., (H_2_O)_5_H^+^, into smaller species, i.e., (H_2_O)_3_H^+^ with higher transport mobility through hydrogen bond interactions based hydration competition, which reduces water transmembrane transport energy barriers and broadens the cross-sectional area available to water molecules, thus improving the water permeance. The polyhydroxy quaternized membrane exhibits high permeability with a membrane flux of 43.01 L m^−2^ h^−1^ bar^−1^, while maintaining favorable divalent salt retention performance and mechanical properties, which effectively improves the selectivity-permeability upper bound of the nanofiltration membrane. Our findings demonstrate an interface-functionalized strategy for regulating the water cluster structure, showing implications for various applications including nanofluidics, desalination, bio-medicine, and energy technology, etc.

## Abstract (web summary)

By dissociating water clusters, the quaternized polyhydroxy-functionalized interface lowers the water transport energy barrier and enhances water permeance in nanofiltration membrane.

## Introduction

**Fig. 1:**
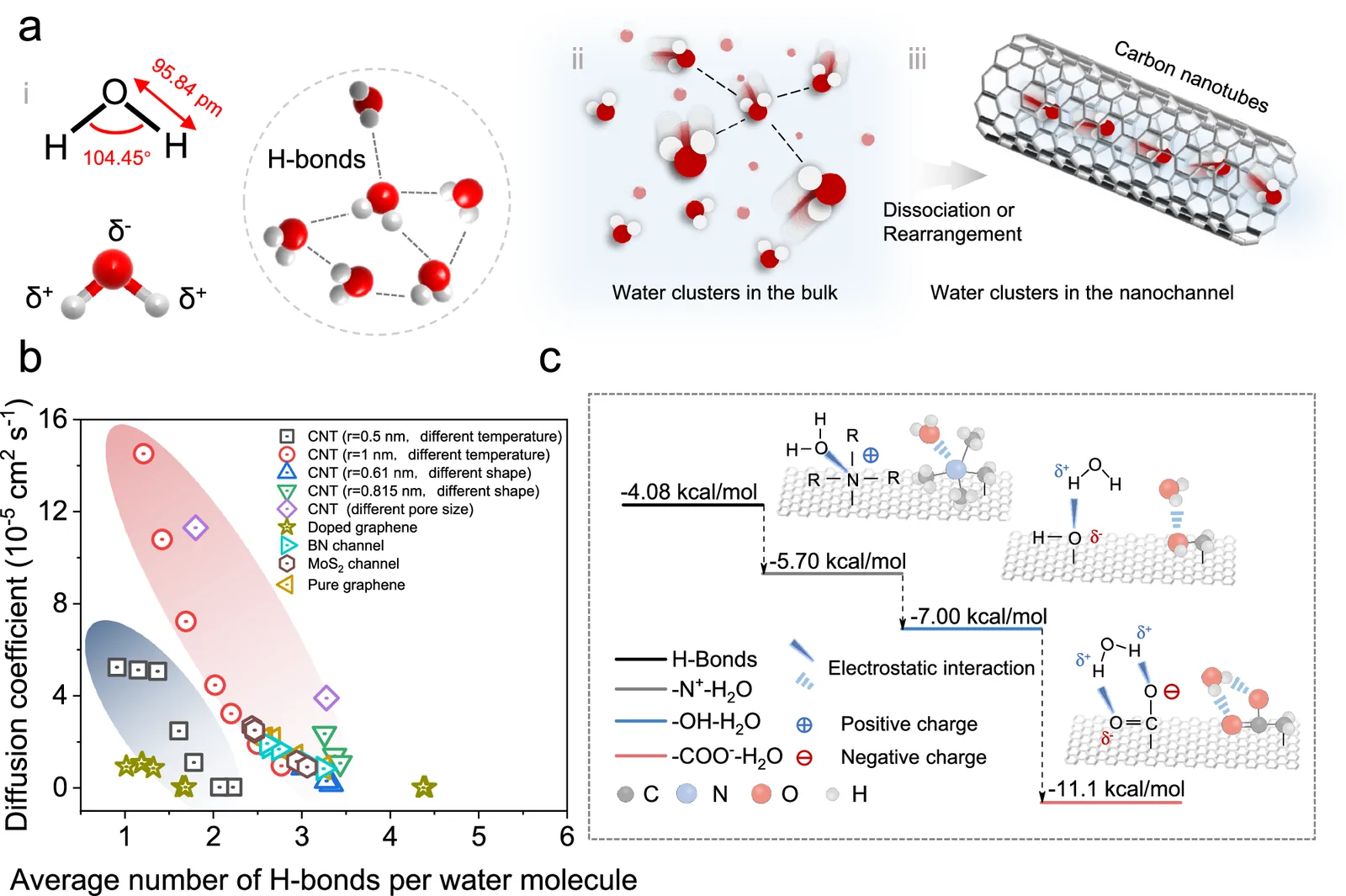
Discussion of promoting water molecule transport through water cluster-dissociation. **a** Water clusters in the bulk solution and their transport in nanochannels due to dissociation, δ^−^ and δ^+^ denote partial negative and partial positive charges, respectively. Colors of atoms: O, red; H, white. **b** The relationship between the average number of H-bonds per water molecule in water clusters and their diffusion coefficients within different nanochannels^10,13,20–22^ (details in Supplementary Table 1), the shaded region highlights the trend that fewer water–water H-bonds correspond to faster water mobility. **c** The interaction energy between a single water molecule and the three functional groups i.e., -R_3_N^+^, -OH, -COO^-^ and the self-association energy of the water molecule. Inset: schematic diagram of the interaction between three functional groups and a water molecule.

Water molecules in aqueous solution typically interconnect through hydrogen-bond (H-bond) to form complex water cluster networks^1^ (Fig. 1a-i). Fundamentally, their structural changes govern the dynamic physicochemical properties of water such as permeation behavior within confined nanochannels^2^. This is crucial to purification technologies that aims at efficient water acquisition. Among them, nanofiltration (NF), an important and widely employed separation technique in water-related applications^3–6^, enables water to permeate through nanochannels in the membrane barrier while rejecting impurities. Currently, fundamental understanding of how water clusters transform in nanochannels remains elusive, which has constrained the development of high-performance NF membranes and given rise to a series of technical challenges. For instance, the intrinsic trade-off between permeance and selectivity in polymeric NF membranes typically leads to practical issues, including low energy-efficiency and brine management burden^7,8^. Essentially, the permeance of water inside nanochannels of the NF membrane involves the dynamic displacement/reconstruction/rearrangement of the water clusters^9^. Therefore, tailored design of the membrane structure to regulate or optimize the transport behavior of water clusters within nanochannels, e.g., reducing the size of the H-bond network for energy-favorable water transport^10^, may fundamentally provide a technical pathway to enhancing water permeability and break through the upper bound in NF separation.

Extensive research has been devoted to improving the water permeance without compromising its solute rejection. One strategy is to develop an active layer by increasing the water transport pathways, i.e., a rougher active layer, which can be achieved by using nanocrystals/nanoparticles templating^5^. Another approach is to reduce the thickness of the active layer thereby shortening the water transport pathway^11^. Although these enhancement strategies for water permeance—based on the water resistance and membrane structure relationship—have been reported effective to some extent, however, a molecular picture of the mechanism that governs how water molecules move through the nanochannel is not well understood, and significant variations of water transport properties inside polyamide-based membrane is challenging to achieve as water molecules are confined by the tortuous polymeric nanochannels^5^. Few researchers have focused on the matching relationship between the water molecule transport behavior and permeance enhancement, which could be fundamentally crucial to surpassing the limitation of permeance and selectivity trade-off. For instance, the magic number effect of (H_2_O)_n_Na^+^ indicates that (H_2_O)_3_Na^+^ moves significantly faster than that of other (H_2_O)_n_Na^+^ ^12^. Moreover, in various nano-confined systems, e.g., carbon nanotube porins (CNTPs)^13^ and fluorous-functionalized nanochannels^14^, it has been observed that water clusters with smaller sizes have higher permeability. These exciting findings suggest that functionalizing membrane materials to regulate the transport behavior of water molecules to enhance permeability could be an attractive strategy for developing high-performance membrane materials for the next generation.

According to traditional dehydration theories, water clusters tend to rearrange the structure and even lose their bound water for reducing size under extreme confinement^15^. Nevertheless, this size-assisted dehydration typically incurs a high energy barrier, which is unfavorable for water transport^16^. Many studies have reported that the electrostatic interactions or hydrophobic interactions based on highly electronegative functional groups can inhibit the formation of hydrogen bonds within water clusters, thus reducing their size to facilitate water transport^10,14^. Some researchers also claimed that charged carboxyl and amino groups of the polyamide-based membrane can also capture water molecules through electrostatic attraction to regulate the structure of water clusters^17^—although, due to the lack of appropriate *operando* characterization techniques, these hypotheses have not yet been experimentally supported.

Herein, we presented a facile strategy to improve membrane permeance based on functionalized interfacial polymerization that leverages hydration competition for water clusters’ dissociation. First, through density functional theory (DFT) and molecular dynamics (MD) simulations, we confirmed that quaternary-N⁺, -OH, and -COO^-^, which act as hydrogen bond acceptors/donors, can enthalpically/entropically favorably dissociate water clusters via the strong interactions between functional group and water molecule. Following this mechanism, the fresh polyamide composite membranes were directly modified by multi-hydroxy quaternary ammonium salt monomer (QBTS). In combination with in situ liquid time-of-flight secondary ion mass spectrometry (ToF-SIMS) and MD simulation, we found that the QBTS active sites, i.e., -OH and quaternary-N⁺ dissociated the water clusters with larger sizes, i.e., (H_2_O)_5_H^+^ into smaller species, i.e., (H_2_O)_3_H^+^ with higher mobility at less entropic and enthalpic cost. This reduces the energy barriers of water’s transmembrane transport and increases their effective transport area, thus leading to higher permeability. Under optimal conditions, the functionalized membrane shows high permeability with a membrane flux of 43.01 L m^−2^ h^−1^ bar^−1^, while maintaining over 97.63% Na_2_SO_4_ retention performance and long-term operational stability. Our study demonstrates an interface-functionalized approach that overcomes the trade-off between permeability and solute-solute selectivity by introducing active sites for water cluster dissociation. The findings provide deeper insights into nanofluidics and interfacial water–related applications such as membrane separation, catalysis, and sensing.

## Results

### Dissociation of water clusters based on hydration competition between water molecules and functional groups

The structural rearrangement of water clusters during transport can lead to reduced size or increased order, thereby facilitating fast transport of water molecules inside nanochannels^13,18,19^. A typical scenario is the high permeability of one-dimensional single-file chain water molecules inside carbon nanotubes (CNTs) (Fig. 1a-iii). As shown in Fig. 1b, we have summarized the relationship between the average number of H-bonds per water molecule in water clusters and their diffusion coefficients within different nanochannels^10,13,20–22^, e.g., CNTPs (details in Supplementary Table 1). The results indicate an approximately inverse relationship between the water permeability and the average number of H-bonds per water molecule in water clusters inside nanochannels—as the H-bonds in water clusters decrease or rearrange, the transport resistance of water molecules could be reduced. The dehydration phenomenon by confinement effect has been widely reported to reduce the size of water clusters, however, this requires overcoming high energy barriers to destroy strong H-bond interactions inside water clusters^23,24^. In contrast, based on the formation mechanism of the water clusters with a hydrogen bonding network, hydration competition between functional groups with H-bonds donor/acceptor and water molecules could be another energy-favorable strategy to achieve dissociation of water clusters^25,26^. Specifically, according to the previous studies on the disruption of water cluster networks by functional groups such as hydroxyl groups^27–29^, when functional groups possess H-bond acceptors/donors that can interact more strongly with water molecules, they tend to form stronger local interactions with water preferentially, thereby “capture” water molecules and create oriented anchoring, cutting off or weakening the original water-water H-bond connectivity, reducing the thermodynamic benefit of maintaining the tetrahedral network, and transforming the continuous ordered water network into a smaller and unrestrained local structure^27–29^. Compared with the destruction of water clusters by extreme confinement, this mechanism is expected to be more favorable for water transport in nanochannels, because the functional group-water interactions can partially compensate the enthalpic loss associated with water-cluster dissociation^29–31^, while the increased availability of water molecular orientations helps reduce the entropic penalty during transmembrane transport.

**Fig. 2:**
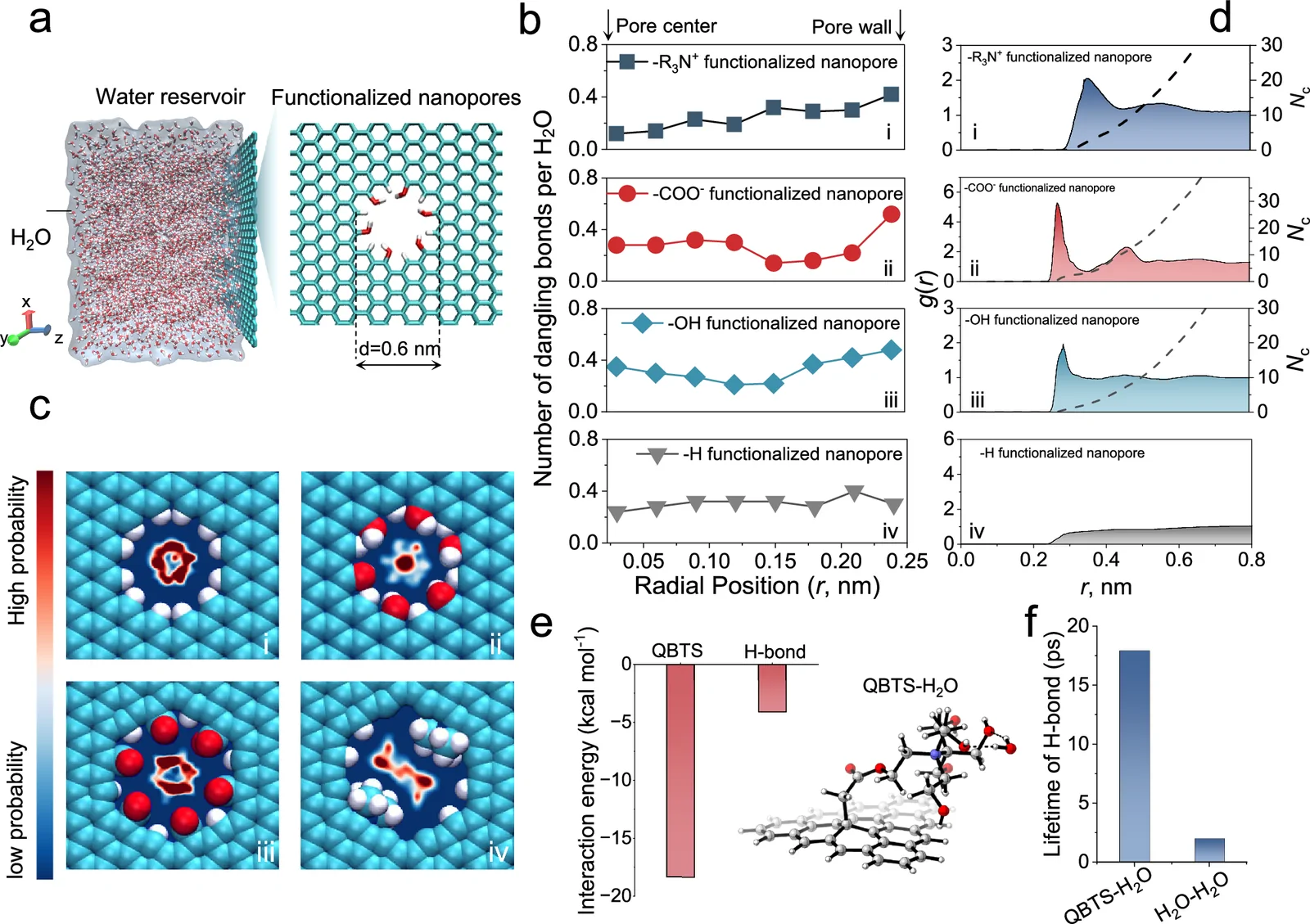
Mechanisms of water cluster-dissociation from hydration competition by functional groups. **a** Side view of the MD simulation system investigated in this work. Colors of atoms: O, red; H, white. **b** Dangling bond radial distribution of water clusters in different functionalized pores. **c** Oxygen density maps in different functionalized pores, i-iv represent -H, -OH, -COO^−^ and -R_3_N^+^ functionalized pores, respectively. Colors of atoms: O, red; H, white; N, blue; C, cyan. **d** RDFs between water molecule and the functional groups, and the corresponding coordination numbers (*N*_c_), i.e., N-O_w_, O-H_w_, and H-O_w_ for -R_3_N^+^, -COO^−^ and -OH, respectively. Solid curves show *g*(*r*) and dashed curves show the running coordination number, *N*_c_, calculated by integrating the corresponding RDF curves. **e** The interaction energy between a single water molecule and the QBTS functional group. The inset shows the double-site interaction between QBTS and water molecule. Colors of atoms: O, red; H, white; N, blue; C, gray. **f** The H-bond lifetime of QBTS-water and water-water calculating by MD simulation.

To verify this, we selected three typical functional groups modified onto membranes in NF processes—carboxyl (-COO^-^), hydroxyl (-OH), and quaternary ammonium (-R_3_N^+^, R represents Methyl) to explore their interactions with water molecules (Fig. 1c and Supplementary Fig. 1) using DFT calculations. Compared to the average bond energies, i.e., self-association energies within water cluster hydrogen networks^32^ (−4.08 kcal mol^−1^), the binding energies of all three functional groups with single water molecule were higher, measured at −11.1, −7.0, and −5.7 kcal mol^−1^ for -COO^-^, -OH and -R_3_N^+^, respectively. The N^+^ interacts with the oxygen atom in the water molecule through electrostatic attraction. The two negatively charged oxygen atoms in -COO^−^, as the H-bond acceptor, can interact with hydrogen atoms in water molecules to form hydrogen bonds. For -OH, the lone pair of electrons on its oxygen atom interact with hydrogen atoms in water molecules to form new H-bonds. Subsequently, we employed MD simulations to analyze this hydration competition mechanism. As illustrated in Fig. 2a and Supplementary Fig. 2, a series of inert graphene pores functionalized at the edges with -H, -OH, -COO^−^, and -R₃N^+^ were constructed to investigate the influence of functional groups on structural changes of water clusters during transport. We first evaluated the radial distribution of the number of dangling bonds (DBs) around different functional groups in the pore rim of perforated graphene. In our study, any H atom that was not hydrogen-bonded to other water molecules was defined as a dangling bond (DB). Therefore, the DB represents a free water molecule that has “escaped” from the water cluster^14,33^. The more DBs there are, the less favorable the formation of water clusters^14,33^. In the control case of the H-functionalized pore, the fluctuation in DBs of the water cluster along the radial direction was minimal, indicating limited structural perturbation of water clusters by the inert pore edges^14^. As the water cluster approached the -OH and -COO^−^, the number of DBs initially decreased and then increased (Fig. 2b). This trend can be attributed to the competition between functional group-water interactions and the internal hydrogen-bond network of the water clusters^34,35^. Specifically, as the water cluster approaches the -OH and -COO^−^, water molecules at the cluster edge form additional hydrogen bonds with the -OH and -COO^−^, leading to an initial reduction in DBs. However, upon further approach, the interaction between the -OH/-COO^−^ and water molecules begins to dominate, potentially distorting the internal structure of the water cluster, thereby increasing the number of DBs. The monotonically increased DBs in -R₃N^+^ functionalized pore could be due to its significant steric hindrance and the hydrophobic nature of the alkyl chains^14^, which suppresses the formation of hydrogen bonds. The calculated density maps for oxygen atoms of water molecules inside functionalized pores (Fig. 2c) show the structural rearrangement of water clusters. In -OH and -COO^-^ functionalized pores, the water clusters were pulled toward the functional groups along the pore walls, resulting in a broadening of the cross-sectional area available to water molecules. In contrast, in -R_3_N^+^ functionalized pore, a pronounced squeezing effect on water clusters due to steric hindrance was observed. Furthermore, the radial distribution functions (RDFs) of the interactions between water and the functional group exhibited distinct first peaks for all three functional groups (Fig. 2d), indicating strong hydration. The strength of water-functional group interaction follows the order: -COO⁻ > -OH > -R_3_N^+^, consistent with the interaction energy calculations presented in Fig. 1c. In summary, a water cluster-breakage near the functionalized pore edges was found due to the hydration competition, which, according to published claims^14,29–31^, would be beneficial for enlarging the water transport area and reducing the transmembrane resistance^29^. Such disruption/dissociation of water cluster structures, resulting from the stronger interactions between hydroxyl/carboxyl groups and water molecules, has also been reported in various scenarios, such as electrode interfaces, solution chemistry, and nanofluidics^27,31,36,37^.

Typically, polyamide nanofiltration membranes feature -COO^-^ at their surfaces^38^. These isolated -COO^-^ may promote the dissociation of water clusters at the membrane surface, but internal hydrogen bonding between excessive carboxyl could attenuate this effect^39^. In contrast, we propose that the polyhydroxy quaternary ammonium structure could further facilitate the dissociation of water clusters—the positively charged quaternary-N⁺ center attracts water clusters to the active sites via electrostatic adsorption, and the polyhydroxy network with rich hydrogen-bond donating sites, can more effectively promote cluster dissociation. DFT calculation results show that the polyhydroxy quaternary ammonium structure indeed exhibits much stronger functional group-water interaction energies (−18.3 kcal mol^−1^) than carboxyl and hydroxyl groups (Fig. 1c and Fig. 2e). Meanwhile, H-bonds with longer lifetimes relative to the water-water network can form near QBTS (Fig. 2f and Supplementary Fig. 3), indicating that compared with water itself, water molecules indeed prefer to interact with the polyhydroxy structure. Combining these results, we speculate that the polyhydroxy quaternary ammonium structure may facilitate the dissociation of water clusters, showing potential for facile water transport. Based on the above results, in the next section, we will introduce the QBTS monomer into the active layer of the NF membrane, which is expected to promote the transmembrane transport of water molecules via the water cluster dissociation mechanism.

### Fabricating an NF membrane with a polyhydroxy quaternized interface to dissociate water clusters

**Fig. 3:**
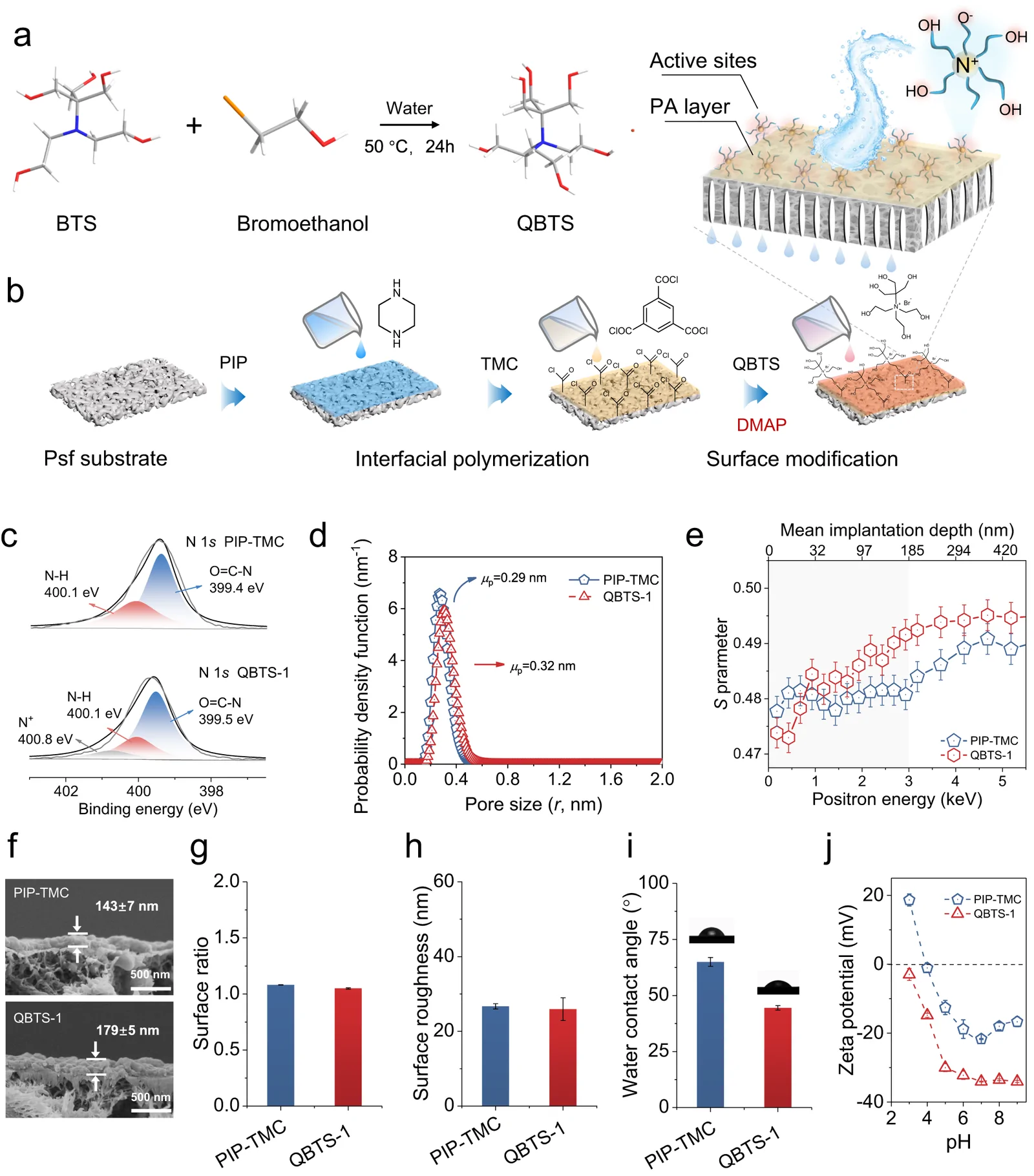
Fabrication of QBTS-functionalized NF membrane and its structure characterization. **a** The synthesis reaction of QBTS using BTS and 2-bromoethanol as monomer. Colors in the ball-and-stick models: O, red; C, gray; H, white; N, blue; Br, orange. **b** Schematic preparation of the pristine polyamide membrane (PIP-TMC) functionalized by QBTS molecules, and schematic illustration of membrane structures after QBTS functionalization. **c** N 1*s* X-ray photoelectron spectra of PIP-TMC and QBTS-1 membranes, respectively. **d** Pore size distribution curves of the PIP-TMC and QBTS-1 membranes. **e** The *S* parameters as a function of positron energy for PIP-TMC and QBTS-1 membranes, were revealed by the positron annihilation Doppler broadening energy spectroscopy (DBES). The gray shaded region corresponds to the QBTS-1 functional layer from 0 to 179 nm, indicating the thickness range over which the corresponding *S* parameter profile was analyzed. **f** Scanning electron microscope (SEM) characterization of membrane cross sections. **g**–**j** Surface roughness, surface area ratio, water contact angle, and zeta potential of the PIP-TMC and QBTS-1 membranes, respectively. The inset in (**i**) shows the water contact angle photograph. For data presented with error bars, unless otherwise stated, data are presented as mean ± standard deviation (SD) from at least three replicate measurements.

An NF membrane was functionalized for water cluster dissociation using multi-hydroxy quaternary ammonium salts with a specific spatial structure as active functional groups. The quaternary ammonium salt monomer QBTS, *N*-(trimethylolmethane)-*N*,*N*,*N*-tris (2-hydroxyethyl) ammonium bromide, was successfully synthesized using 2-bromoethanol and bis (2-hydroxyethyl) amino-tris (hydroxymethyl) methane (Bis-Tris), with its structure characterized by ^1^H NMR (Fig. 3a and Supplementary Fig. 4)^40^. An initial thin-film composite membrane, designated as PIP-TMC, was prepared on a polysulfone ultrafiltration support using piperazine (PIP) and trimesoyl chloride (TMC) through interfacial polymerization^8,40^. The QBTS aqueous solution was then applied to the newly synthesized PIP-TMC surface for interface functionalization, resulting in the modified membrane named QBTS-1 (Fig. 3b, details in Supplementary Fig. 5)^40,41^. A new XPS peak at 400.8 eV in N 1*s* core level spectra is seen from QBTS-1 (Fig. 3c, Supplementary Figs. 6 and 7), which is assigned to quaternary-N⁺ due to the incorporation of QBTS^42^, confirming the successful modification of PIP-TMC by the QBTS monomers. Additionally, FTIR analysis of the membrane modified with QBTS monomers reveals new absorption peaks at 984 cm⁻¹ and 1676 cm⁻¹, corresponding to quaternary-N⁺ and ester groups^43^, further validating the successful modification of PIP-TMC by QBTS monomers (Supplementary Fig. 8).

We found that the thickness of the active layer increased from 143 to 179 nm after QBTS functionalization due to the interface reaction between QBTS monomer and unreacted acyl chloride groups (Fig. 3f and Supplementary Fig. 9). This may result in a top-to-bottom structure rearrangement in the active layer which loosens the uppermost active layer for higher water permeability^44^. The functionalized QBTS-1 membrane also showed numerous nodular structures (Supplementary Fig. 10) that are conducive to water permeation^45^. Below the new functional layer, the denser polyamide layer effectively hinders ion transport^40^, ensuring that the QBTS-1 membrane facilitates rapid water transport while efficiently retaining ions. Interfacial modification typically has a significant impact on the membrane pore size and hence the transport resistance of water^42^. However, as shown in Fig. 3d and Supplementary Table 3, we observed almost identical pore size distributions of the membranes before and after QBTS functionalization. The pore structure of PIP-TMC and QBTS-1 membranes was further characterized using positron annihilation-DBES. Typically, the longitudinal *S*-parameter correlates positively with void volume in nanofiltration membranes^46^. Given that the membrane thickness ranges from approximately 143 to 179 nm (Fig. 3f), positron energies of 0–3 keV were selected to compare the free volume of PIP-TMC and QBTS-1 membranes. As shown in Fig. 3e, the free volume of the PIP-TMC membrane remains largely unchanged with increasing positron energy, suggesting a homogeneous void structure. In contrast, the *S*-value of QBTS-1 initially falls below that of PIP-TMC before surpassing it, indicating that the QBTS-1 membrane’s active layer transitions from a loose structure to a dense one with increasing depth, and then gradually loosens again. Despite this variation, both membranes exhibit comparable pore size and pore size distribution (Fig. 3d), implying similar overall void-structure characteristics. In the *S-W* plot, the three red fitted lines from left to right correspond to the interfacial modification layer, the polyamide (PA) layer, and the porous polysulfone (PSF) supporting layer in the QBTS-1 membrane (Supplementary Fig. 11), confirming its distinct three-layer structure^47,48^.

On the other hand, the surface area ratio and surface roughness of the active layer remained relatively unchanged before and after QBTS functionalization, as compared to the PIP-TMC membrane (Fig. 3g, h and Supplementary Fig. 12). These results indicate that, in this study, the chemical characteristics of the functional groups, rather than the structural properties of the modified QBTS membrane, primarily influence the transport of water molecules. In addition, the QBTS membrane exhibited a more hydrophilic surface (Fig. 3i) and a more negative zeta potential (Fig. 3j). According to previous studies^49–52^, higher hydrophilicity may originate from the stronger interaction between the polyhydroxy structure of QBTS near the membrane pores and water molecules, which helps distort the local network of water clusters and spread along the membrane surface rather than aggregating into clusters. Meanwhile, the richer local negative charge near the membrane pores could help disrupt the H-bond network between water molecules through electrostatic interactions and reduce the entropic penalty. Therefore, QBTS on the membrane surface was expected to promote the partitioning of water molecules from aqueous into the membrane thereby enhancing water flux^53^.

### Performance evaluation on QBTS functionalized NF membrane

**Fig. 4:**
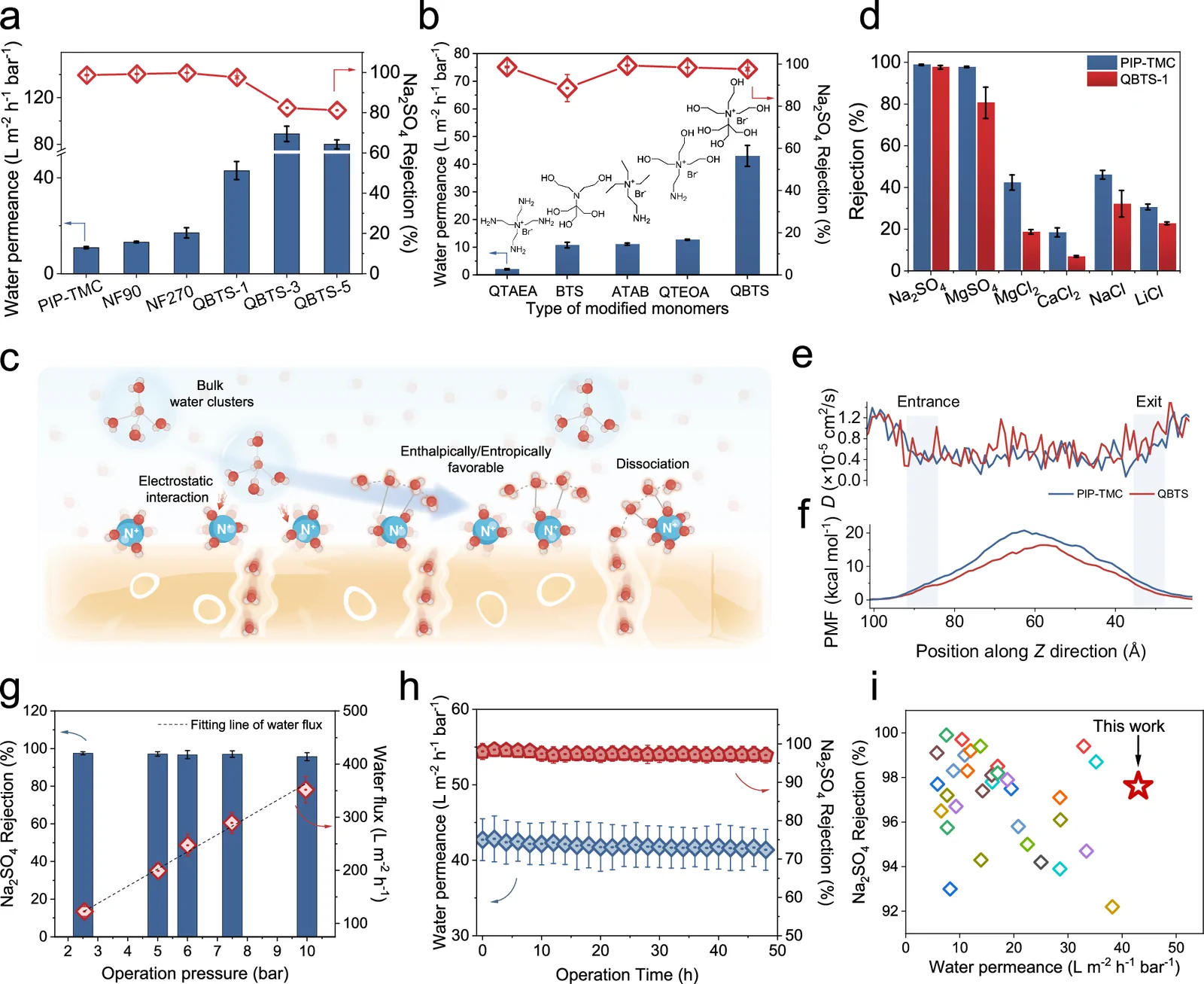
Filtration performance of QBTS functionalized NF membranes. **a** Effect of QBTS monomer concentration on the separation performance of NF membranes (feed: 1000 ppm Na_2_SO_4_, 6 bar pressure). **b** Filtration performance of NF membranes modified with different monomers (feed: 1000 ppm Na_2_SO_4_, 6 bar pressure). **c** Schematic diagram of the suppositional mechanism of functional groups promoting the dissociation of water clusters. The dashed lines between water molecules indicate that the original water‑water hydrogen bonds are weakened by the polyhydroxy structure of QBTS. Colors of atoms: O, red; H, white; N, blue. **d** Salts rejection of Na_2_SO_4_, MgSO_4_, MgCl_2_, CaCl_2_, LiCl and NaCl by PIP-TMC and QBTS-1 membranes (Salt concentration, 1000 ppm; applied pressure, 6 bar). **e** The potential of mean force (PMF) and **f** diffusion coefficients (*D*) of Cl⁻ along the diffusion coordinate in PIP TMC and QBTS simulated nanochannels. The filtration performance of QBTS-1 under different (**g**) operation pressure and (**h**) operation Time (feed: 1000 ppm Na_2_SO_4_, 6 bar pressure). **i** Performance comparison of QBTS-1 membrane with the state-of-the-art NF membranes using surface modification in reported literature (The specific values and corresponding references in this figure are provided in Supplementary Table 2). Diamonds indicate membrane performance data reported in the literature, and the star indicates the membrane performance obtained in this work. For data presented with error bars, unless otherwise stated, data are presented as mean ± standard deviation (SD) from at least three replicate measurements.

We first evaluated the water flux of the functionalized membranes with different QBTS monomer concentrations, i.e., QBTS-1 (1 wt%), QBTS-3 (3 wt%), and QBTS-5 (5 wt%) (Fig. 4a). As the concentration of QBTS monomer increases, the water flux of the membrane gradually increases, but with the rejection of Na_2_SO_4_ gradually decreases. We found that functionalizing PIP-TMC with 1 wt% QBTS shows the optimal membrane separation performance. Specifically, the water flux of QBTS-1 reaches 43.01 L m^−2^ h^−1^ bar^−1^ with a satisfied Na_2_SO_4_ rejection rate of 97.63%, which is four times that of the pristine  PIP-TMC NF membrane. The increase in QBTS monomer concentration facilitates the incorporation of residual acyl chloride into the PA layer during interfacial modification, while also promoting the structural rearrangement of the newly formed polyamide network. This process likely results in an enlarged pore size, thereby compromising salt rejection performance^8,44,54^. Nevertheless, we found that all QBTS-functionalized membranes exhibited superior water permeability compared to commercial membranes NF 90 and NF 270, with the most significant improvement observed under optimal conditions, i.e., QBTS-1 (Fig. 4a).

To gain deeper insights into the mechanism by which surface functional groups on the active layer enhance water flux, we selected five amine monomers with similar spatial structures but different functional groups to functionalize the PIP-TMC membrane, i.e., *N*,*N*,*N*,*N*-Tetra (2-aminoethyl) ammonium bromide (QTAEA), Bis-(2-Hydroxyethyl) amino-tris (Hydroxymethyl) methane (BTS), *N*,*N*,*N*-triethyl-*N*-(2-aminoethyl) ammonium bromide (ATAB), *N*,*N*,*N*-Tris (2-hydroxyethyl)-*N*-(2-aminoethyl) ammonium bromide (QTEOA) and QBTS (details in Fig. 4b, Supplementary Figs. 5 and 13). The water flux order of the functional membranes is as follows: QTAEA < BTS < ATAB < QTEOA < QBTS (Fig. 4b). In the absence of hydrophilic hydroxyl groups, the highly reactive -NH_2_ of QTAEA undergoes multi‑point reactions with residual acyl chlorides, forming a locally dense structure that is unfavorable for water permeation^55,56^; consequently, its water permeance is even lower than that of PIP‑TMC. For BTS, although it contains a relatively high number of hydroxyl groups, it lacks a quaternary-N^+^, showing a modest improvement in water flux. Previous studies have shown that the hydration free energy of quaternary ammonium cations is significantly more favorable than that of neutral amines, meaning that the quaternary-N^+^ center itself is a good hydration site^57^—as reflected by the slight increase in water flux observed for ATAB compared with PIP‑TMC. QTEOA possesses both a polyhydroxy structure and a quaternary-N^+^ center, but its high reactive -NH_2_ likely leads to a relatively dense active layer^55^. In contrast, QBTS is grafted via a less reactive esterification reaction and possesses both polyhydroxy groups and a quaternary-N⁺ center, thus exhibiting a significantly enhanced water-transport effect compared with the other monomers. Overall, we suppose that when a monomer contains both abundant polyhydroxy structures and a quaternary-N^+^ center, it enhances the water permeance of the membrane.

The physicochemical characteristics and hydraulic permeability results of QBTS (Supplementary Fig. 14) also indicate that the enhancement in water permeance mainly originates from the membrane surface chemistry. Based on the results shown in Fig. 2b, c, e, f and previous reports, the polyhydroxy structure of QBTS is expected to exhibit stronger interactions with water molecules, thereby perturbing or even dissociating water clusters^27–29^. This could help form a more disordered, defect-rich, higher-entropy local structure^27–29^. Moreover, the positively charged quaternary-N^+^ center has also been reported to attract water molecules, facilitating the formation of a hydration layer^57^. Based on these facts, we speculate the following mechanism for the enhanced water permeance of QBTS. Specifically, as shown in Fig. 4c, during the partitioning process where water clusters from the aqueous phase go into the membrane phase driven by pressure, larger water clusters approach the active sites through the electrostatic adsorption by quaternary-N⁺ in QBTS. Subsequently, the polyhydroxy network in QBTS preferentially interacts with water molecules, disrupting the original water-water network and forming a locally loose water structure. Numerous previous studies have shown that such functional group-water interactions can lower the enthalpic barrier for dissociation through a compensation effect^29,30^ and reduce the entropic penalty by providing more available orientations for water molecules^29^. This entropically and enthalpically favorable effect likely contributes to high water flux of QBTS. We will discuss the mechanism of QBTS for enhancing water transport in more detail in the next section.

We further explore the ion selectivity of the QBTS functionalized membranes. The results show that the QBTS functionalized membranes maintained good monovalent/divalent ion selectivity (Fig. 4d), which matches the typical trends observed in negatively charged polyamide-based NF membrane stems from the synergy of size exclusion and the Donnan effect. Simultaneously, we observed that QBTS-1 slightly reduced the retention of the four salts, particularly for salts containing monovalent Cl^-^, i.e., MgCl_2_, CaCl_2_, LiCl and NaCl. We thus used molecular dynamics to investigate ion rejection behavior in simulated polyamide nanochannels (details in Supplementary Materials and Methods). As shown in Fig. 4e, f, we calculated the diffusion coefficients and potential of mean force (PMF) of Cl^-^ along the diffusion coordinate in PIP-TMC and QBTS simulated nanochannels. Unexpectedly, the PMF of Cl^-^ in QBTS was lower, decreasing from 20.75 to 16.39 kcal mol^−1^ (Fig. 4f). Meanwhile, as shown in Fig. 4e, during transport through the QBTS pore, Cl^−^ exhibited several favorable transport regions, such as near the pore entrance (~90 Å), showing higher diffusion coefficients. Using the inhomogeneous solubility-diffusion model, the calculated permeability coefficient across the entire membrane increased correspondingly from 1.10 × 10^−13^ to 1.20 × 10^−10 ^cm s^−1^, indicating that Cl^−^ is more easily transported in QBTS. These simulation results are consistent with the experimentally observed lower rejection of MgCl_2_, CaCl_2_, NaCl, and LiCl by QBTS-1. This phenomenon seems counterintuitive for QBTS, which has a more negative zeta potential. However, similar observations have been reported in many nanopores with -OH-modified entrances^29,58,59^. Based these previous studies, we speculate two possible reasons for the reduction of Cl^-^ rejection: i) the polyhydroxy structure in QBTS can interact with Cl⁻ through a “dehydration compensation” mechanism^29,30,60^, replacing some of the bound water lost from the hydrated Cl^−^, thereby stabilizing the dehydration process of Cl^-^ and lowering the dehydration energy barrier; ii) the quaternary-N^+^ near the pore entrance may help direct Cl^−^ toward the pore by electrostatic adsorption, making Cl^−^ transport more favorable. In addition, the polyhydroxy quaternary structure of QBTS can complex with cations^61^, particularly Mg^2+^, further enhancing the affinity between the cations and the membrane. These Mg^2+^ adsorbed onto the membrane surface further induce charge neutralization^62^, thereby weakening the repulsion of the membrane surface negative charge against co‑ions. These effects together result in a more pronounced decrease in rejection of MgCl_2_, NaCl, LiCl, and CaCl_2_ after QBTS modification.

Meanwhile, QBTS-1 demonstrated a high flux of other salts (Supplementary Fig. 15). This indicates that in the presence of competing coexisting salt ions, QBTS can still maintain high water permeance. As a polyhydroxy quaternary ammonium salt, QBTS also endows the modified membrane surface with good antifouling and antibacterial properties (Supplementary Figs. 16 and 17). After QBTS interfacial modification, the overall mechanical strength of QBTS-1 remained excellent (Supplementary Fig. 18). In addition, we evaluated the stability of the QBTS membrane performance under different operating pressures, various salt concentrations, and long‑term operation. As shown in Fig. 4g, QBTS‑1 maintained good rejection and permeation performance within the operating pressure range of 2.5–10 bar. Even under high‑salt conditions of 10 g L^−1^, it still exhibited good salt rejection (Supplementary Fig. 19). Furthermore, it also demonstrated long‑term operational stability (Fig. 4h), indicating that the promoting effect of QBTS on water transport does not fail after long‑term operation. Overall, compared with other reported performances of advanced membranes using surface modification (Fig. 4i and Supplementary Table 2), the fabricated QBTS-functionalized NF membrane showed high water permeability while maintaining good salt rejection performance, which can be attributed to the dissociation effect of QBTS active sites on the water clusters.

### Transport mechanism of water molecule in NF membrane with polyhydroxy quaternized interface

**Fig. 5:**
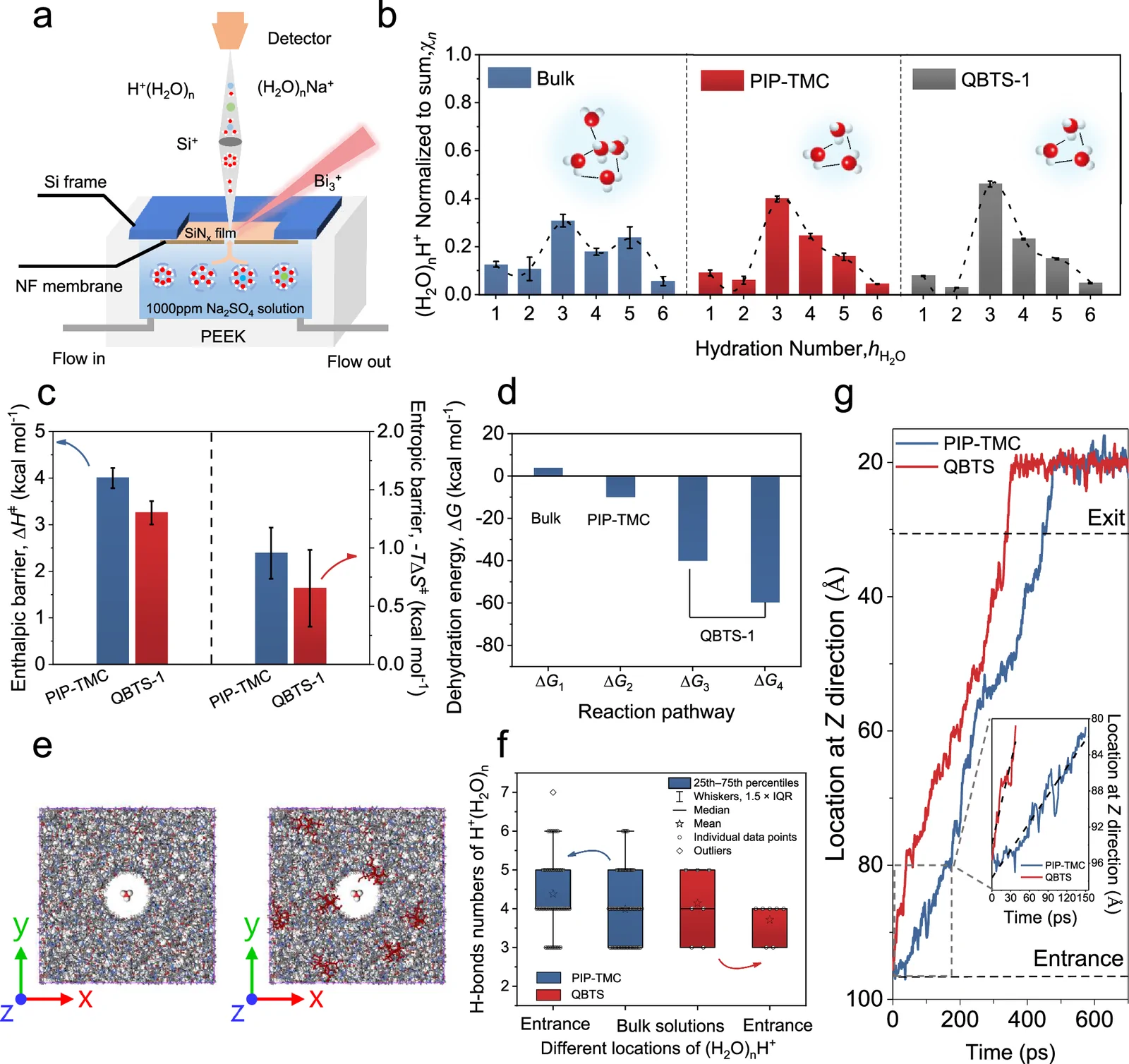
Mechanisms of transport of water molecules in QBTS functionalized NF membrane based on water clusters dissociation. **a** Schematic diagram of in situ liquid ToF-SIMS analysis combined with the microfluidic nanofiltration platform. PEEK denotes polyether ether ketone. Colors: O, red; C, gray; H, white; N, blue; Cl, purple. The dark red structures on the model surface in the right-side image represent the grafted QBTS monomer. **b** Hydration number distribution of water clusters $({h}_{{{{\rm{H}}}}_{2}{{\rm{O}}}})$ in 1000 ppm Na_2_SO_4_ before and after filtration by two different membranes. Insets show representative hydrated proton clusters, including (H_2_O)_5_H^+^ in the bulk solution and (H_2_O)_3_H^+^ after filtration through the PIP-TMC and QBTS-1 membranes, respectively. The changes in their relative abundances indicate the conversion of larger hydrated proton clusters into smaller hydrated species during membrane filtration. Dashed lines between bars are shown only as visual guides. Colors of atoms: O, red; H, white. **c** Enthalpic and entropic barriers for the transport of water through the PIP-TMC and QBTS-1 membrane. The energy barriers were calculation fitted by intrinsic water permeability measured at 298.15, 303.15, 308.15, and 313.15 K (details in Supplementary Materials and Methods). **d** Gibbs free energy (Δ*G*) of a serial of typical dehydration process with and without participation of functional groups. **e** Modeling of pristine  PIP-TMC (left) and QBTS modified (right) nanochannels. **f** H-bonds numbers of the water clusters in the bulk solution and at the entrance of the PIP-TMC and QBTS nanochannels, respectively. **g** The relationship between the Z-direction position and traversing time of the water clusters in the nanochannel. The inset shows the linear fitting results of water clusters’ transport velocities at the entrance of two nanochannels. For data presented with error bars, unless otherwise stated, data are presented as mean ± standard deviation (SD) from at least three replicate measurements.

As shown in Fig. 5a and Supplementary Fig. 20, we used an in situ liquid ToF-SIMS microfluidic filtration platform to analyze the changes in hydration species of water clusters in solution before and after filtration by the pristine and functionalized NF membranes, in order to *operando* analyze the dissociation of water clusters during transmembrane transport (details are provided in Supplementary Materials and Methods). Our previous studies have demonstrated that in situ liquid ToF-SIMS is applicable for detecting solvation structures and weak water-water interactions within the solution phase and even during hydrate transport through the polymeric membrane^23,63,64^. The spectral peak intensities of (H_2_O)_n_H⁺ (*n* = 1–6) were normalized to the total water cluster mass spectral intensity to obtain the hydration distributions (denoted as ${h}_{{{{\rm{H}}}}_{2}{{\rm{O}}}}$, Fig. 5b). Briefly, during the measurement, when the SiN_x_ film is perforated by the Bi_3_^+^, hydrated species in the aqueous solution, such as (H_2_O)_n_H^+^, are driven by the external vacuum pressure difference to pass through the nanofiltration membrane and reach the detection liquid surface. The hydrated species in the liquid phase are immediately ionized and detected *operando* (denoted as “after filtration”). For the bulk solution, we removed the nanofiltration membrane shown in Fig. 5a and performed the measurement following the same procedure. The species detected in this way represent the hydration state of (H_2_O)_n_H⁺ in the bulk solution, i.e., the “before filtration”. Therefore, compared with ${h}_{{{{\rm{H}}}}_{2}{{\rm{O}}}}$ before filtration, the ${h}_{{{{\rm{H}}}}_{2}{{\rm{O}}}}$ of water clusters after filtration reflects the influence of the physicochemical characteristics of the nanofiltration membrane, such as surface functional groups and membrane pore size, on their hydration state.

In bulk solution (before nanofiltration), the water clusters showed a probability distribution with water trimer, tetramer and pentamer as the dominant water species, which is consistent with the previously reported works^65^. We then compared the ${h}_{{{{\rm{H}}}}_{2}{{\rm{O}}}}$ after being filtrated by  PIP-TMC and QBTS-1 membranes, respectively (Fig. 5b, middle and right panels). After filtration by NF membrane, it shows that the proportion of (H_2_O)_5_H^+^ decreased, while that of (H_2_O)_3_H^+^ significantly increased compared to the bulk solution (Fig. 5b, left panels). Water clusters are indeed transported through nanochannels in a reduced size form as previous study claimed^66^. We thus hypothesize that the steric hindrance of the confined nanochannels may contribute to the dissociation of water clusters as we mentioned earlier^23^ (Fig. 1c). Previous studies have also shown that water clusters are transported through polyamide nanochannel in a smaller form compared to that in the bulk phase^66^. More importantly, in the ${h}_{{{{\rm{H}}}}_{2}{{\rm{O}}}}$ after filtration through the QBTS-functionalized membrane, this increase in the proportion of (H_2_O)_3_H^+^ is more pronounced compared with that of  PIP-TMC membrane, as the proportion of (H_2_O)_5_H^+^ decreased from 0.24 to 0.15, while that of (H_2_O)_3_H^+^ increased from 0.30 to 0.46 (Fig. 5b). These results are consistent with the observation of more DBs near the functional groups in Fig. 2b. As we mentioned earlier, the polyhydroxy quaternary ammonium structure is expected to promote the dissociation of water clusters through strong interactions with water molecules. Therefore, the observed increase in the proportion of (H_2_O)_3_H^+^ is likely evidence that the polyhydroxy quaternary ammonium structure promotes the generation of more small-sized clusters, facilitating the formation of more small-sized water cluster species, thereby further promoting transmembrane water transport.

The enthalpic barrier (∆*H* ^‡^) and entropic barrier (∆*S*^‡^) of water were calculated using transition-state theory in membrane separation^23^. As shown in Fig. 5c and Supplementary Fig. 21, compared to the PIP-TMC membrane, water molecules traverse the QBTS-1 membrane with a lower enthalpic energy barrier, decreasing from 4.00 kcal mol^-1^ to 3.25 kcal mol^−1^. Additionally, water molecules experience a lower entropic barrier when crossing the QBTS-1 membrane, suggesting a higher degree of freedom during permeation, which further facilitates their transmembrane transport (Fig. 5c). Using the migration data of water molecules in different nanochannels from molecular dynamics simulations (Fig. 5g), the entropic penalty calculated based on the simplified Arrhenius kinetic model further confirms that water diffusion in QBTS is entropically more favorable (Supplementary Materials and Methods). After QBTS modification, water clusters show a lower entropic barrier and greater degrees of freedom, consistent with our MD observations of increased DBs near the functional groups and the expanding of water clusters. The calculated apparent activation energy also suggests a significant reduction in the energy required for water molecules to cross the modified membrane (Supplementary Fig. 22).

Overall, the results indicate that QBTS-1 is more conducive to water-molecule transport with lower transport resistance. This corresponds with our ToF-SIMS results that the smaller transmembrane water clusters can contribute to higher water permeability (Fig. 5b). We calculated the Gibbs free energy (Δ*G*) of a serial of typical dehydration process with and without the participation of functional groups to further evaluate their roles in water clusters dissociation (details in Supplementary Fig. 23). For the process of dehydration from (H_2_O)_6_H^+^ to (H_2_O)_3_H^+^, the positive Δ*G*_1_ showed a non-spontaneous dehydration process in the bulk solution. In contrast, the involvement of functional groups (-COO^−^, -OH, and quaternary-N⁺) in the dehydration process (details in Supplementary Fig. 23) led to a negative Δ*G* (Fig. 5d), indicating that the hydration competition is favorable for water clusters to spontaneous dissociation to transform as smaller water species. The lower Δ*G*_3_ and Δ*G*_4_ signified that the QBTS functional groups on the active layer make the dissociation of water clusters easier compared to the -COOH in polyamide (Fig. 5d), which also corresponds well with our previous observation by in situ liquid ToF-SIMS (Fig. 5b) and membrane performance evaluation (Fig. 4a). Based on the dehydration compensation mechanism^29,30^, we speculate that when QBTS participates in the dissociation of water clusters, the interaction between the functional groups and the defective water clusters takes over part of the stabilization role originally provided by the water-water H-bond network. This compensates for the energetic cost of losing water–water H-bonds.

We then conducted MD simulations to further explore the transport behavior of water clusters across the functionalized NF membrane. The polymeric membrane was simplified into a nanochannel with a radius of approximately 0.3 nm within polyamide bulk (Fig. 5e and Supplementary Fig. 24), the QBTS monomers are grafted onto the surface of PIP-TMC to obtain functional sites without significantly affecting the pore size. As shown in Fig. 5f and Supplementary Fig. 25, at the pore entrance of QBTS-nanochannel, the H-bonds number in water clusters is limited from 3-5 in the bulk solution to 3-4, coinciding with a decreasing average hydration number. This reveals that the QBTS active sites indeed lead to reduced association and smaller size of the water clusters. Hence, the simulation results are consistent with our observations obtained using in situ liquid ToF-SIMS (Fig. 5b). In contrast, the pristine  PIP-TMC nanochannel exhibits no significant dissociation effect on the water clusters. The transport mobility of the water clusters, observed as the relationship between the Z-direction position and traversing time of the water clusters in the nanochannel (Fig. 5g), also supported our conclusion. Specifically, the water clusters can more rapidly enter the entrance of QBTS nanochannel, as their transport speed at the entrance of QBTS nanochannel is approximately three times that at the entrance of PIP-TMC nanochannel (Fig. 5g inset), which can be attributed to the additional “acceleration” to water clusters through energy compensation of hydration competition^67^. This phenomenon supports the significantly enhanced water permeance observed in QBTS‑1 (Fig. 4a). Finally, we further analyzed the potential of mean force (PMF) during transmembrane water transport in the PIP-TMC and QBTS-simulated nanochannels. As shown in Supplementary Fig. 26, the PMF experienced during water transport is lower after QBTS grafting, which also supports the observations of the lower enthalpic barrier (Fig. 5c) and favored water permeance in QBTS (Fig. 4a).

Although it is undeniable that the stronger interaction between the QBTS and water molecules may introduce unfavorable viscous resistance^58,68^, however, it promotes water permeation through the following effects. Specifically, based on existing knowledge of the influence of functional groups on water transport^27,69^ and these observations, the polyhydroxy quaternary ammonium structure, through its stronger interactions with water molecules, disrupts the water cluster network and dissociates it into more small-sized water clusters^14,27,69,70^, thereby lowering the enthalpic barrier. Meanwhile, the polyhydroxy quaternary ammonium structure provides more accessible orientations for water molecules near the pore entrance^29^, thereby reducing the entropic penalty for water clusters entering the confined environment. As a result, the overall transport velocity of water clusters is higher in QBTS nanochannel—although that inside the PIP-TMC and QBTS are similar due to the identical internal environments (Fig. 5g). Our results indicate that the pore entrance functionalized with QBTS functional groups can promote transmembrane transport of water molecules by providing additional “acceleration” due to hydration competition, further reducing water transport resistance, and improving water flux.

## Discussion

Water clusters, as the fundamental units of transmembrane water transport, inherently influence the fluid characteristics within confined channels. Although previous studies have observed that the extreme confinement environment of dense polyamide membranes forces water clusters to reduce their hydration size to complete transport at the cost of energy consumption^10,66^, and have established a fundamental understanding of the relationship between water cluster structure and water flux^10,14,66^, how to precisely design membrane structures to regulate the confined transport structure of water clusters and thereby enhance flux remains an open question. In this study, using in situ liquid ToF-SIMS in combination with MD simulations, we demonstrated that the NF membrane with polyhydroxy quaternized interface can dissociate water clusters through the hydration competition effect of -OH and quaternary-N⁺, thereby reducing water transport resistance and further enhancing membrane flux. Under optimal modified conditions, the QBTS-functionalized NF membranes significantly increased water flux while maintaining high Na_2_SO_4_ rejection, achieving a membrane water flux of 43.01 L m⁻² h⁻¹ bar⁻¹. From the practical application viewpoint, this modified method has the potential for large-scale production due to the minor deviation from traditional interface polymerization. In conclusion, the results presented in our study provide deeper insights into the mechanisms governing water permeability regarding the regulation of interfacial water cluster structures. More importantly, in other scenarios such as electrode interfaces process and nanofluidics^27,31,36,37^, carboxyl and hydroxyl groups have also been observed to disrupt water cluster structures through hydration competition, oriented locking of water molecules, and local electric field construction, thereby affecting water-related interfacial processes. This indicates that the dissociation of water clusters by functional groups observed in our study is referenceable. The transport mechanisms of water molecules based on the dissociation of water clusters can be extended to other interfacial water-related applications, including energy technology, bio-medicine, water treatment and catalysis, etc.

## Methods

### Synthesis of control monomers

To synthesize QBTS, Bis (2-hydroxyethyl) amino-(trihydroxymethyl) methane (14.65 g) and 2-bromoethanol (12.5 g) were added to a 100 mL flask and stirred at 50 °C for 24 h. The precipitate was collected and washed three times with ethyl acetate. The final product was dried in a vacuum oven at 50 °C for 12 h. The synthesis methods of other modification monomers, i.e., QTAEA, BTS, ATAB, and QTEOA, can be found in Supplementary Materials and Methods.

### Nanofiltration Membrane Preparation

For the preparation of PIP-TMC NF membranes, PIP aqueous solution (0.3 wt%) was poured onto the surface of the PSf supporting membrane, maintained for 3 min, and the superfluous PIP solution was removed. Subsequently, a hexane solution of TMC (0.3 wt%) was poured onto the membrane and left for 1 min. After that, the membrane was heated at 50 °C for 10 min. Following the reaction, the membrane was rinsed with DI water to obtain the PIP-TMC TFCMs, which were stored in DI water.

For the preparation of QBTS NF membranes, QBTS was grafted onto the membrane surface via the reaction of its hydroxyl groups with residual acyl chloride groups of TMC in the freshly prepared polyamide active layer, i.e., an esterification reaction between acyl chloride and hydroxyl groups. This method is also commonly used to graft functional groups containing hydroxyl groups onto polyamide membranes. As shown in Supplementary Fig. 5, a freshly prepared PIP-TMC NF membrane was treated with an aqueous solution of QBTS (1 wt%) and DMAP (1 wt%) for 10 min. The residual acyl chloride groups of TMC undergo an esterification reaction with the hydroxyl groups of QBTS under the catalysis of 4‑dimethylaminopyridine (DMAP), a nucleophilic acyl transfer catalyst, thereby immobilizing QBTS onto the polyamide network. It is important to note that the PIP-TMC NF membrane was not dried prior to the QBTS modification. The residual reaction solution was then removed, and the membrane was heated at 50 °C for 10 min. After rinsing with DI water, the membrane was stored in DI water. The PIP-TMC NF membranes were modified with QBTS solutions at different mass fractions of 1 wt%, 3 wt%, and 5 wt%. The resulting NF membranes are denoted as QBTS-1, QBTS-3, and QBTS-5, respectively. Modifications of the PIP-TMC NF membrane with other monomers (QTAEA, BTS, ATAB, and QTEOA) followed similar protocols. Detailed explanation of the reaction mechanisms of the other monomers was shown in Supplementary Materials and Methods.

### Fabrication of the microfluidic filtration device

The microfluidic platform for in situ liquid ToF-SIMS analysis was fabricated following established protocols^23,71^. Specifically, a 100 nm-thick SiN_x_ film supported by a silicon frame (window size: 0.5 mm × 0.5 mm) served as the analytical window. A membrane sample (3.5 mm × 3.5 mm) was affixed beneath the SiN_x_ film, with the active layer oriented toward the vacuum to minimize ion rehydration. The SiN_x_ film was integrated into a PEEK block containing a liquid chamber (6.0 mm × 5.2 mm × 1.0 mm, length × width × depth), also sealed with epoxy resin. Two PEEK tubes were inserted and sealed on opposite sides of the chamber, with a 1/16-in. tube as the outlet and a 1/32-inch tube as the inlet. After introducing 1000 ppm Na₂SO₄ solution through the inlet, the chamber was sealed via valve closure.

### Analysis of hydration distributions by in situ Liquid ToF-SIMS

The hydration distribution of (H_2_O)_n_H^+^ (*n* = 1-6) $({h}_{{{{\rm{H}}}}^{+}})$ before and after filtration through PIP-TMC and QBTS-1 was assessed. Measurements were conducted in positive ion mode using a ToF-SIMS V instrument (ION-TOF GmbH, Münster, Germany) under in situ liquid SIMS conditions, with minor modifications to established protocols^63,64,71–73^. A 30 keV Bi_3_^+^ primary ion beam (10 kHz) was focused to ~350 nm and rastered over a 2 μm-diameter circular area at the center of the SiN_x_ window (pulse width: 162.5 ns; current: ~0.27 pA). Upon breakthrough of the membrane by the beam, electrolyte solution from the microfluidic device was driven across the membrane toward the vacuum side (1–2 × 10⁻⁶ mbar). To enhance mass resolution, the pulse width was immediately reduced to 100 ns. Mass spectra of the individual hydrated species were acquired immediately upon their passage through the membrane, allowing real-time characterization of the hydration distribution (Supplementary Figs. 27 and 28). Specifically, the hydrates were ionized directly upon exiting the nanochannels and detected within ~10–200 μs via time-of-flight analysis, effectively preserving their pristine hydration structures during transmembrane transport.

### Evaluation of nanofiltration performance

The permeation performance of the membranes was evaluated using a cross-flow filtration setup. The effective membrane area was 20.6 cm², and the solution temperature was maintained at 25 °C using a heat exchanger. To stabilize the membranes, they were pre-pressurized at 8 bar for 0.5 h, followed by stable operation at 6 bar for 1 h before sampling.

Water permeability (*J*) was determined using Eq. 1:

1 $$J=\frac{m}{A\cdot {{\rm{\cdot }}}\Delta t\cdot {{\rm{\cdot }}}\Delta p\cdot {{\rm{\cdot }}}{\rho }_{{{\rm{w}}}}}$$

where *J* is the water permeability (L m⁻² h⁻¹ bar⁻¹), *m* (kg) is the mass of the collected permeate over the filtration time Δ*t* (h), *ρ*_w_ (1 g cm^-^³) is the density of the permeate, *A* (m^2^) is the effective membrane area, and Δ*p* (bar) is the transmembrane pressure.

The salt rejection rate (*R*) was calculated using Eq. 2:

2 $$R=\frac{{C}_{0}-{C}_{{{\rm{p}}}}}{{C}_{0}}\times 100\%$$

Where *R* (%) represents the salt rejection rate, *c*_0_ and *c*_p_ are the concentrations of the feed and permeate solutions, respectively, salt concentrations are measured with the conductivity meter.

### Radial distribution function and hydration number calculation

The microscopic distribution of water molecules and ions was characterized using radial distribution function (RDF) analysis^74^. The RDF between species *i* and *j*, *g*_*ij*_(*r*) was used to quantify the relative probability of finding species *j* at a distance *r* from species *i*. It was calculated according to Eq. 3:

3 $${g}_{{ij}}(r)=\frac{d{N}_{{ij}}(r)}{4\pi {r}^{2}{\rho }_{j}{dr}}$$

where d*N*_*ij*_(*r*) is the average number of species *j* in a spherical shell between *r* and *r*+d*r* around species *i*, *ρ*_*j*_ is the average number density of species *j*, and *r* is the distance from the selected reference species.

The coordination number, *N*_c_, was further calculated from the RDF by integrating the hydration shell, as described in Eq. 4:

4 $${N}_{c}=4\pi {\rho }_{j}{\int }_{\!\!\!\!\!0}^{r}{r}^{2}{g}_{{ij}}(r){dr}$$

### Reporting summary

Further information on research design is available in the Nature Portfolio Reporting Summary linked to this article.

## Supplementary information

## Source data

## Data Availability

The data generated in this study are available within the paper and the Supplementary Information. Source data are provided with this paper.
