# Supplementary material for: Dissociating water clusters via polyhydroxy quaternized interface for enhanced water permeation in nanochannels
Source: Nat Commun. 2026 Aug 5;17:9435. doi: 10.1038/s41467-026-76291-z (PMC13538467; doi:10.1038/s41467-026-76291-z)
Supplement: Supplementary file 1 [file 41467_2026_76291_MOESM1_ESM.pdf]

Supplementary Information

Dissociating Water Clusters via Polyhydroxy Quaternized Interface for Enhanced Water Permeation in Nanochannels

Authors

Lei Xu[1, 2 #], Chenghai Lu[1, 2 #], Yanyan Zhang[2, 3], Fuyi Wang[2, 3], Guangzhi He[1, 2], Chengzhi Hu[1, 2 *], Jiuhui Qu[1, 2]

Affiliations

1 Key Laboratory of Environmental Aquatic Chemistry, State Key Laboratory of Regional Environment and Sustainability, Research Center for Eco-Environmental Sciences, Chinese Academy of Sciences, Beijing 100085, China.

2 University of Chinese Academy of Sciences, Beijing 100049, China.

3 Beijing National Laboratory for Molecular Sciences, CAS Key Laboratory of Analytical Chemistry for Living Biosystems, Institute of Chemistry, Chinese Academy of Sciences, Beijing 100190, China.

*Corresponding author Email: czhu@rcees.ac.cn

#These authors contributed equally to this work

List of content

Supplementary Materials and Methods

Supplementary Figs 1 to 28

Supplementary Tables 1 to 3

Supplementary References

Supplementary Materials and Methods

Chemicals and Materials

Polysulfone (PSf) membranes (molecular weight cut-off of 50 kDa) were purchased from Ande Filter Membrane Co., Ltd., Beijing. Trimesoyl chloride (TMC, 98%), Bovine serum albumin (BSA, Fraction V), Triethylamine (TEA), and Bis(2-hydroxyethyl) amino-tris(hydroxymethyl)methane ($C_8H_{19}NO_5$, 98%) were purchased from Aladdin Biochemical Technology Co., Ltd., Shanghai. Tris(2-aminoethyl) amine ($C_6H_{18}N_4$, 98%), 2-bromoethylamine hydrobromide ($C_2H_7Br_2N$, 98%), 2-bromoethanol (C_2H_5BrO, 95%), 4-dimethylaminopyridine (DMAP, 99%), and ethyl acetate ($C_4H_8O_2$, chromatographic grade) were purchased from Innochem Technology Co., Ltd., Beijing. Sodium alginate (SA), N,N-Dimethylformamide (DMF, ≥99%), triethanolamine ($C_6H_{15}NO_3$, analytical grade, ≥78%), piperazine (PIP, ≥99%), sodium sulfate (Na_2SO_4, ≥99%), sodium chloride (NaCl, ≥99.8%), magnesium sulfate ($MgSO_4$•$7H_2O$, ≥99%), magnesium chloride ($MgCl_2$•$6H_2O$), Calcium chloride ($CaCl_2$, ≥96%), Lithium chloride (LiCl), polyethylene glycol (≥99%, MWs of 200, 400, 600, and 800 Da), ethylene glycol ($C_2H_6O_2$, ≥99.5%), glycerol ($C_3H_8O_3$, ≥99%), sucrose ($C_{12}H_{22}O_{11}$, analytical grade), and glucose ($C_6H_{12}O_6$, analytical grade) were purchased from Sinopharm Chemical Regent Co. Ltd., China. Deionized (DI) water (>18.2 megaohms·cm) produced from a MilliQ ultrapure water purification system (Millipore Milli-Q) was used for preparing solutions. The silicon nitride (SiN_x) film window was obtained from Norcada Inc., Edmonton, Canada.

Materials characterizations

The surface morphology and cross-sectional features of the membranes were examined using scanning electron microscopy (SEM, JSM7401F, JEOL, Japan). The Cross-sectional morphology of the membrane was observed by Transmission Electron Microscope (TEM, H-7500, HITACHI, Japan). The chemical composition of the membranes was analyzed through attenuated total reflection Fourier-transform infrared spectroscopy (ATR-FTIR, Nicolet 8700, Thermo Fisher Scientific) and X-ray photoelectron spectroscopy (XPS, ESCALAB 250Xi, Thermo Fisher Scientific, USA). Atomic force microscopy (AFM, Multimode8, Bruker, USA) was employed to evaluate the surface structural characteristics. The surface zeta potential was assessed using a

solid surface potential analyzer (Anton Paar surpass3, Austrian). The water contact angle (WCA) droplets on the membrane surfaces were measured with a contact angle goniometer (OCA200, Delphi, Germany). Mechanical properties were tested using a mechanical property testing machine. The pore size distribution of the membranes was evaluated using an inert solute and pore transport model, with neutral organic molecules quantified by a total organic carbon (TOC) analyzer (TOC-VCPH, Shimadzu, Japan).

Synthesis of control modified monomers

To synthesize QTEOA, 2-bromoethylamine hydrobromide (20 g) was dissolved in 60 mL of DMF. Then, triethanolamine (10 g) was added and stirred at 50°C for 24 hours. The white precipitate was filtered and washed three times with DMF. The purified product was dried in a vacuum oven at 50°C for 12 hours to obtain QTEOA.

To synthesize QTEAE, 2-bromoethylamine hydrobromide (10 g) was dissolved in 30 mL of DMF. Then, tri(2-aminoethyl) amine (5 g) was added and stirred at 50 °C for 24 hours. The white precipitate was filtered and washed three times with DMF. The purified product was dried in a vacuum oven at 50°C for 12 hours to obtain QTEAE.

To synthesize ATAB, 2-bromoethylamine hydrobromide (7.3 g) was dissolved in 20 mL of DMF. Then, triethylamine (3 g) was added and stirred at 50°C for 24 hours. The white precipitate was filtered and washed three times with DMF. The purified product was dried in a vacuum oven at 50°C for 12 hours to obtain ATAB.

Determination details of hydration distributions (h_{H_2O}) using *in situ* liquid ToF-SIMS

The determination principle of the *in situ* liquid ToF-SIMS technique is shown in our previous work[1,2]. As shown in Fig. 5a and Supplementary Fig. 20, the feed solution, 1000 ppm Na_2SO_4, was injected into the home-made microfluidic nanofiltration device. A pulsed Bi_3^+ primary ion beam is constantly applied to the SiN_x film for punching through the detective window. Typically, as shown in Supplementary Fig. 27, after approximately 440s, the SiN_x film was completely punched through, the feed solution was then transported across the membrane and reached the detective area driven by the ultrahigh vacuum pressure difference. Thus, the $(H_2O)_nH^+$ in feed solution were

consequently exposed to the Bi_3^+ primary ion beam and were ionized to secondary ions, forming the instantaneous mass spectrum of interested $(H_2O)_nH^+$.

It should be particularly noted that the water clusters from permeate will be ionized immediately once they exit the nanochannel. In other words, the water clusters were ionized immediately in the aqueous phase rather than in the gas phase, which helps to ensure that the detected SIMS signals maintain the hydration properties of ions from permeate[1–4]. The dynamic ToF-SIMS depth profiling also indicated that the signals of representative species from permeate were immediately increased once the ions reached the detective area (Supplementary Fig. 27). On the other hand, the *in situ* liquid ToF-SIMS has been demonstrated to be a surprisingly soft technique for investigation of water-water interactions[3–6]. During the determination, hydrates fragmentation in liquid SIMS analysis could be alleviated due to the continuously updated liquid surface around the punched pore via diffusion as well as the flexible liquid environments, which is favored for the formation of intact water clusters. In addition, once the SiN_x film was perforated, the pulse width was immediately reduced to 100 ns, which reduces the intensity of the beam current from 0.27 pA to 0.15 pA. This can mitigate the impact on the hydrated ions from ion irradiation. Furthermore, the ionized hydrates can go through the time-of-flight tube and be detected in an extremely short time (duration ~10-200 μs)[4]—greatly minimizing the possibility of collisions and additional evaporation. As such, the original structure of the water clusters is well maintained after water clusters leave the nanochannel, which can represent the operando changes in the ion hydration structure during transport through nanochannels.

We collected the mass spectrum data of $(H_2O)_nH^+$ at the moment of SiN_x perforation from the detection area (see Supplementary Fig. 28). The mass spectrum peaks of $(H_2O)_nH^+$ were assigned according to their mass-to-charge ratio (m/z), and normalized the intensities of individual water clusters to the total intensities of $(H_2O)_nH^+$ to present the hydration distribution ($h_{\mathrm{H_2O}}$),

$$X_n = \frac{I_{(\mathrm{H_2O})_n\mathrm{H^+}}}{\sum_{n=1}^{n=6} I_{(\mathrm{H_2O})_n\mathrm{H^+}}} \tag{1}$$

Therefore, X_n represents the proportion of $(H_2O)_nH^+$ with different bound waters (n=1-6) to $(H_2O)_nH^+$ (X_n=0-1). To better describe the hydration distribution, we used the weighted average to mathematical comparison, i.e.,

$$h_{\mathrm{avg}} = \frac{\sum nX_n}{\sum X_n} \tag{2}$$

The hydration number measured by ToF-SIMS was in good agreement with the strongly-bound water molecules of the hydrates obtained by theoretical extractions based on bulk properties, which has more important implications regarding ion transport behaviour[7–9]. Overall, the hydration numbers determined by ToF-SIMS give a reasonable indication of the variation in hydration numbers when water clusters are transported between different membranes.

Estimated pore size distributions of polyamide membranes via neutral solute transport method

The pore size distribution (PSD) of all membranes were obtained by means of the neutral solute transport method[10–12]. The PSD of PIP-TMC and QBTS-1membranes can be described as the probability density function:

$$\frac{dR_{\mathrm{T}}(r_{\mathrm{p}})}{dr_{\mathrm{p}}} = \frac{1}{\ln\sigma_{\mathrm{p}}\sqrt{2\pi}} \cdot \frac{1}{r_{\mathrm{p}}} \cdot \exp\left[-\frac{(\ln r_{\mathrm{p}} - \ln\mu_{\mathrm{p}})^2}{2(\ln\sigma_{\mathrm{p}})^2}\right] \tag{3}$$

in which, R_{T} is the solute rejection, r_{p} is the effective pore radius of the membrane, μ_{p} is the mean effective pore radius, and σ_{p} is the standard deviation of the effective pore radius.

By neglecting the effects of spatial obstruction and solute-pore hydrodynamic interactions on solute rejection, μ_{p} and σ_{p} can be regarded the same as the geometric mean (μ_{s}) and standard deviation (σ_{s}) of the solute radius, severally. In the meanwhile, μ_{s} is the solute radius (r_{s}) at R_{T}=50%, and σ_{s} is the ratio of r_{s} at R_{T}=84.13% to r_{s} at R_{T}=50%. R_{T} vs. r_{s} can be described as a linear relation of log-normal probability:

$$f(R_{\mathrm{T}}) = A + B(\ln r_{\mathrm{s}}) \tag{4}$$

Where A is the intercept and B is the slope of the fitted line.

We prepared a set of 200 ppm neutral organic solutions to measure solute rejection with different membranes, including glycol, glycerol, glucose and sucrose. The solute

radius (r_s, nm) can be obtained from the equation of the Stokes radius and molecular weight (MW, Da) of the solute:

$$\log r_s = -1.5575 + 0.4911 \log MW \qquad (5)$$

Positron annihilation-Doppler Broadening Energy Spectroscopy (DBES) measurement

The microstructure of membrane is characterized by positron annihilation coupled DBES (Institute of High Energy Physics, Beijing, China). A ^{22}Na radioactive source was employed to generate positrons with incident energies ranging from 0 to 10 keV. Doppler broadening energy spectroscopy was used to detect the γ-photons resulting from positron annihilation, utilizing a high-purity germanium detector. The annihilation characteristics were quantified using two key parameters, i.e., *S* and *W*. The *S* parameter primarily reflects the free volume within the polymer matrix, while the *W* parameter provides insight into the chemical environment and defect types[13]. The total energy window of the collected γ spectrum was 499.5-522.5 keV. The S parameter was defined as the ratio of counts within the 510.2-511.8 keV energy range to the total counts in the full peak range (499.5-522.5 keV). Similarly, the *W* parameter was calculated as the sum of counts in the 513.6-516.9 keV and 505.1-508.4 keV ranges, normalized by the total peak counts.

The average depth of incidence of positrons in the membrane can be calculated by the following equation:

$$Z = \left(\frac{40}{\rho}\right) E^{1.6} \qquad (6)$$

where *Z* is the average implantation depth in the membrane (nm). ρ is the density of the sample (1.25 g cm^{-3} for PIP-TMC and QBTS-1, approximately) and *E* represents the energy of the incident positron.

Determination of energy barrier for water transmembrane transport

According to the solution-diffusion model[14], the intrinsic permeability of water can be calculated by following equation:

$$P_i = \frac{J_i}{\Delta P - \Delta\pi} \cdot \frac{RT}{C_i v_i} \quad (7)$$

where P_i is the intrinsic permeability of water (L m^{-2} h^{-1}), J_i is the water flux (L m^{-2} h^{-1}), ΔP is the pressure gradient across the film (bar), $\Delta\pi$ is the osmotic pressure gradient across the film (bar), T is thermodynamic temperature (K), R is the gas constant, c_i is the molar concentration of water (mol mol^{-1}), and v_i is the molar volume of water (L mol^{-1}).

According to the Eyring's transition-state theory (TST)[15,16], the intrinsic water permeability can be also described with the entropic ($-T\Delta S^{\ddagger}$) and enthalpic ($\Delta H^{\ddagger}$) component of water transport through the nanofiltration membrane:

$$P_i = \lambda^2 \frac{k_B T}{hd} \exp\left(\frac{\Delta S^{\ddagger}}{R}\right) \exp\left(-\frac{\Delta H^{\ddagger}}{RT}\right) \quad (8)$$

where λ is the distance between two equilibrium positions in the membrane, k_B is the Boltzmann constant, h is Planck's constant, R is the gas constant, d is the membrane thickness, $\Delta S^{\ddagger}$ is the entropy of activation, $\Delta H^{\ddagger}$ is the enthalpic component. We measured P_i at different temperature of 298.15, 303.15, 308.15, and 313.15 K (i.e., 25, 30, 35, 40°C). The $-T\Delta S^{\ddagger}$ and $\Delta H^{\ddagger}$thus can be obtained by:

$$\ln\left(\frac{P_i \delta h}{\lambda^2 kT}\right) = \frac{\Delta S^{\dagger}}{R} - \frac{\Delta H^{\dagger}}{RT} \quad (9)$$

The value of λ was taken as an average of 0.5 nm[16,17], and membrane thickness d was measured by SEM method.

We also used the Arrhenius-type equation to evaluate the effective activated energy barrier (E_a) and pre-exponential factor (P_0) of water during transmembrane transport[17,18]:

$$P_i = P_0 \exp\left(-\frac{E_a}{RT}\right) \quad (10)$$

$$\ln P_i = -\frac{E_a}{R} \times \frac{1}{T} + \ln P_0 \quad (11)$$

Mechanical performance test of membranes

The mechanical properties of the membranes were evaluated using an Instron model 3369 tester and in order to obtain reliable results, at least three membrane samples (length × width: 5 cm × 1 cm) per membrane were analyzed. The Young's modulus

can be obtained by:

$$E = \frac{F \cdot L}{A \cdot \Delta L} \quad (12)$$

where E is the Young's modulus (Pa), F is the tensile force applied to both sides of the membrane (N), L is the length of the membrane (m), A is the cross-sectional area of the membrane (m^2), ΔL is the elongation amount of the membrane (m).

Meanwhile, the tensile strength of the membrane can be calculated by:

$$P = \frac{F_{\max}}{A} \quad (13)$$

where P is the tensile strength of the membrane (Pa), $F_{\max}$ is the force exerted at the moment of membrane rupture (N), A is the cross-sectional area of the membrane (m^2).

Antifouling performance test

The antifouling performance of the nanofiltration membranes was evaluated using bovine serum albumin (BSA) and sodium alginate (SA) as model foulants at a high concentration of 1000 ppm. The tests were conducted in dead-end filtration mode under a constant operating pressure of 12 bar and a solution temperature of 25°C. Prior to fouling tests, each membrane was stabilized with deionized water for 1 h at 12 bar. The initial pure water flux (J_0) was then measured by sampling every 20 min over 1 h. Thereafter, the feed was switched to the foulant solution, and the flux was monitored for 4 h (sampled hourly), constituting one fouling cycle. After the first cycle, the membrane was cleaned by switching to deionized water and rinsing in cross-flow mode for 1 h to remove the surface fouling layer. A second fouling cycle was then repeated under the same conditions. Following the two cycles, the recovered pure water flux (J_i) was measured again by sampling every 20 min. The flux recovery ratio (FRR) was calculated as:

$$FRR = (J_i/J_0) \times 100\% \quad (14)$$

Antibacterial performance test

The antibacterial performance of the QBTS-1 and commercial NF270 nanofiltration membranes was evaluated using *Escherichia coli* (ATCC25922, Gram-negative) and

Staphylococcus aureus (ATCC29213, Gram-positive) as model bacteria. The detailed procedure is described below.

For the preparation of culture media, Luria-Bertani (LB) liquid medium was prepared by dissolving 2.5 g of LB broth in 100 mL of distilled water and autoclaving at 121°C for 15 min. LB solid medium was prepared by dissolving 2.5 g of LB broth and 1.5 g of agar in 100 mL of distilled water, autoclaving at 121°C for 15 min, and pouring into sterile Petri dishes (15 mL per dish) after cooling to 40–50°C. A 1/500 NB aqueous solution was prepared by dissolving 1.8 g of nutrient broth in 100 mL of deionized water, diluting it 500-fold, and then sterilizing at 115°C for 30 min. For bacterial suspension preparation, single colonies of *S. aureus* and *E. coli* were separately inoculated into 3 mL of LB liquid medium in 12-mL culture tubes and incubated overnight (15 h) at 37°C with shaking at 200 rpm, while a tube without inoculation served as the blank control. For sample treatment and contact culture, membrane samples were cut into 2 cm × 2 cm squares and sterilized under UV light for 30 min; cover films (1.5 cm × 1.5 cm) were soaked in alcohol for 1 h and air-dried.

The overnight bacterial culture was diluted with 1/500 NB solution to 10^5 CFU mL^{-1}, and then 64 µL of the diluted suspension was evenly dropped onto each membrane surface. A sterilized cover film was placed over the droplet to ensure uniform contact without overflow, and samples were incubated statically at 37°C for 24 h. For elution, plating, and counting, after incubation the bacteria were eluted with 6.4 mL of sterile PBS, serially diluted 10-fold with sterile PBS, and 100 µL of appropriate dilutions were spread on LB solid plates; after incubation at 37°C for 18 h, colonies were photographed and counted.

The antibacterial rate of the membrane was calculated as:

$$R = \left(1 - \frac{M_i}{M_0}\right) \times 100\% \qquad (15)$$

where M_i is the number of bacterial colonies (CFU) recovered from the QBTS-1 or NF270 membrane, and M_0 is the number of colonies from the membrane-free control group.

Stability tests of the nanofiltration membrane

The separation performance and long-term stability of the QBTS-1 nanofiltration membrane were evaluated under different pressures and salt concentrations. Prior to each test, the membrane was pre-stabilized at 8 bar and 25°C for 1 h. The specific test conditions were as follows: Pressure-dependent test: Conducted using a 1000 ppm Na_2SO_4 solution at pressures of 2.5, 5, 6, 7.5, and 10 bar. Concentration-dependent test: Performed with Na_2SO_4 solutions at concentrations of 1, 3, 5, 7, and 10 g L^{-1} under an operating pressure of 6 bar. Long-term stability test: Carried out continuously for 48 h at 6 bar and 25°C, with water permeability recorded every 2 h.

Water permeability (*J*) was determined using Equation 1:

$$J = \frac{m}{A \cdot \Delta t \cdot \Delta p \cdot \rho_{\mathrm{w}}} \qquad (16)$$

where J is the water permeability (L m^{-2} h^{-1} bar^{-1}), m (kg) is the mass of the collected permeate over the filtration time Δt (h), ρ_w (1 g cm^{-3}) is the density of the permeate, A (m^2) is the effective membrane area, and Δp (bar) is the transmembrane pressure.

The salt rejection rate (*R*) was calculated using Equation 2:

$$R = \frac{C_0 - C_{\mathrm{p}}}{C_0} \times 100\% \qquad (17)$$

Where R (%) represents the salt rejection rate, c_0 and c_p are the concentrations of the feed and permeate solutions, respectively, salt concentrations are measured with the conductivity meter.

Density functional theory (DFT) calculations

We used Ab initio DFT to calculate the interaction of functional groups with the dehydration process of water molecules and water clusters. DFT calculations were performed using the Gaussian 16 program[19]. At the theoretical level of B3LYP/6-31G(d)[20] at 298.15 K and 1 atm[21,22], all molecular geometries were optimized using SMD[23] as the Grimme's D3 dispersion correction for the solvation mode in water. Harmonic vibration analysis verifies that the optimized minimum value has no virtual frequency. In order to improve the calculated energy, single-point calculations with a large base set of 6-311 G(d,p) were performed using the same B3LYP-D3BJ functional group as the SMD as the solvation mode in water.

Molecular Dynamics Simulations

In this study, we did not use exactly the same force field across three independent and distinct MD simulations, namely: i) Hydration competition between functional groups and water molecules; ii) Confined transport of water molecules in different Nanochannels; and iii) calculation of H-bond lifetimes near QBTS. Because the specific sub-questions addressed by these three MD simulations are different and the compositions of their models are not identical, we did not enforce a uniform force field across all three simulations. Instead, we selected a more appropriate force field for each specific system—as has been reported in many other studies[24–29]. Specifically, for the two similar simulation systems based on functionalized graphene, namely i) and iii), we used a consistent OPLS-AA force field, whereas for the polyamide nanochannel simulation system, i.e., ii), we chose the PCFF-INTERFACE force field.

Hydration competition between functional groups and water molecules. In order to investigate the hydration competition mechanism of functional groups, we constructed four pores with a pore size of 0.6 nm on the surface of monolayer graphene. On the inner surfaces of these pores, we functionalized -H, -OH, $-COO^-$, and $-R_3N^+$ groups, respectively. Such a simplified perforated graphene serves as a "elementary model" of the mechanism. Compared with the complex pore size distribution and structural heterogeneity of a polyamide network, functionalized perforated graphene enables a clean analysis of the effects of functional groups on water cluster structure (e.g., DBs, RDF), following approaches widely used in the literature[30–32]. A standard molecular mechanics potential model was used with the following functional form:

$$u(r^N) = \sum_{bonds}\frac{k_i}{2}\left(l_i - l_{i,0}\right)^2 + \sum_{angles}\frac{k_i}{2}\left(\theta_i - \theta_{i,0}\right)^2 + \sum_{torsions}\frac{V_n}{2}(1+\cos(n\omega - \gamma)) + \sum_{i=1}^{N}\sum_{j=i+1}^{N}\left(4\varepsilon_{ij}\left[\left(\frac{\sigma_{ij}}{r_{ij}}\right)^{12} - \left(\frac{\sigma_{ij}}{r_{ij}}\right)^{6}\right] + \frac{q_i q_j}{r_{ij}}\right) \quad (18)$$

where the first three terms are the bonded interactions, including bond, angle, and torsion interactions, and the second terms are nonbonded interactions, including van der Waals (vdW) and Coulombic interactions. For different kinds of atoms, the Lorentz-Berthelot mix rules were adopted for vdW interactions, which is following the equation:

$$\sigma_{ij} = \frac{1}{2}\left(\sigma_{ii} + \sigma_{jj}\right);\ \varepsilon_{ij} = (\varepsilon_{ii} * \varepsilon_{jj})^{1/2} \quad (19)$$

According to the experimental conditions, several systems were constructed. The initial

configurations of the systems were constructed using the Packmol software[33]. The simulations were performed using GROMACS package (version 2023.3)[34–37] with the all-atom OPLS force field[38]. The OPLS-AA force field has been successfully used to simulate similar complex interface systems, proving its suitability for the "hydration competition" mechanism we investigate[30–32]. For each system, the steep descent method was used to minimize the energy of the system. After minimization, molecular dynamics simulations under NVT ensemble were performed for 50 ns for each system. LINCS algorithm[39] was applied to constrain the bond lengths of other components. Periodic boundary conditions were applied in all three directions. The temperature was maintained using the V-rescale thermostat algorithm[40]. The pressure control was changed to the Parrinello-Rahman method in the production run. The cut-off distance for the Lennard-Jones and electrostatic interactions was 1.2 nm. Particle mesh Ewald method[41] was used to calculate the long-range electrostatic interactions. Configurations were visualized using Visual Molecular Dynamics software[42].

In this work, H-bonds were determined according to geometric criteria: an O-H···O bond was defined as a H-bond only when the distance between the donor O-H and the acceptor O was less than 2.5 Å and the angle was greater than 90°. O-H···O bonds that did not meet these criteria were classified as dangling bonds (DBs). It is worth noting that this criterion is broader compared with those reported in other literature[43–46]; accordingly, the criterion for DBs is relatively stricter, and the resulting DB statistics in our study can more accurately reflect the influence of functional groups on the structure of water clusters. Based on this, in the MD simulations, our calculated DBs are defined as a water molecule (mol1) within a specified distance from the pore center (i.e., radial position) that does not form an H-bond with surrounding water molecules (mol2)[47–50]. For an H atom in *mol1*, if it does not satisfy the H-bonding criteria with any of the surrounding water molecules, that H atom is counted as having one DB. The final DB value is calculated as the total number of DBs divided by the total number of water molecules, which corresponds to the metric of "Number of dangling bonds per H_2O" reported in Fig. 2.

Confined transport of water molecules in different Nanochannels. In order to study

the transport behavior of $(H_2O)_nH^+$ and ion in two types of polyamide nanochannels, two simulated nanochannel models were constructed, namely PIP-TMC and QBTS (Supplementary Fig. 24). It is worth noting that both nanochannels are composed of polyamide units, thus retaining the basic physicochemical characteristics of the polyamide nanofiltration membrane prepared in this experiment[1,2,51,52]. Both models have dimensions of 45.0×45.0×120.0 (X×Y×Z) $Å^3$. In both models, the original layer was stacked from duplicate polyamide units with a thickness of 6.2 nm and embedded in the center of the membrane with a cylindrical nanopore of a radius of about 0.3 nm along the Z-axis. In PIP-TMC, the top surface of the membrane is ionized by replacing the exposed -COOH group with a $-COO^-$ group. In QBTS, the top surface of the membrane was first grafted with seven QBTS molecules by esterification. To maintain charge neutrality in the simulation system and to mimic the actual nanofiltration process in our study, we randomly placed different ions in the water box, as detailed below: the aqueous solution in PIP-TMC contained 15 Na^+, 1 Cl^-, 1 SO_4^{2-}, and 4329 H_2O molecules. The aqueous phase in QBTS included 15 Na^+, 1 Cl^-, 7 Br^-, 1 SO_4^{2-}, and 4218 H_2O molecules. Meanwhile, some of the carboxyl and hydroxyl groups in PIP-TMC and QBTS were ionized to ensure charge neutrality of the system. The species of interest (hydronium hydrate ions or Cl^-) was initially placed above the top surface of the membrane, located at the central axis of the cylindrical nanopore (Supplementary Fig. 24).

A 3D periodic condition was applied to both models. In order to eliminate spatial overlap or inappropriate geometry, the energy of the two models was first minimized. After that, a 0.5 ns simulation of the molecular dynamics of the NVT ensemble was performed at 300 K. During these relaxation processes, both the polyamide unit and the hydronium hydrate ions of the membranes of both models remained fixed. In the official run, the hydronium hydrate ions are de-immobilized and their centroid was attached to a virtual spring with an external force constant of 50 pN, which is fixed in a plane 1.0 nm from the bottom surface of the membrane. A 1000 ps guided molecular dynamics simulation was then performed to induce the transport of hydronium ions through the nanopores. Data collection was performed in 1.0 ps time steps to allow for subsequent statistical analysis.

For the umbrella sampling production runs, the ion of interest was released from its fixed position. A total of 86 umbrella sampling windows were deployed along the Z-axis, spanning from $z = 102.7$ Å to $z = 17.7$ Å with a uniform spacing of 1.0 Å between

adjacent windows, thereby covering the entire membrane thickness and portions of the bulk solution on both sides. For each window, the simulation proceeded in three sequential stages: (i) the ion was first steered to the target position by applying a harmonic biasing potential with a force constant of 5.0 kcal mol^{-1} $Å^{-2}$) tethered to the nanochannel central axis in the X and Y directions and to the target Z coordinate, and a 30 ps pulling simulation was conducted; (ii) the restraints in the X and Y directions were then released, retaining only the Z-direction harmonic restraint at the same force constant, and a 200 ps relaxation was performed to allow the ion and surrounding solvent molecules to equilibrate at the target Z position; (iii) a 500 ps production trajectory was then collected for data analysis, during which the center-of-mass position of the ion along the Z-axis was recorded every 100 fs. The polyamide membrane atoms were kept fixed throughout all simulations. The same umbrella sampling protocol, including the identical initial and final positions, window spacing, and spring constant, was applied to both hydronium hydrate ions and Cl^- ions in both PIP-TMC and QBTS. The PMF profiles were reconstructed from the biased umbrella sampling trajectories using the weighted histogram analysis method (WHAM)[53].

The position-dependent diffusion coefficients were extracted from the same umbrella sampling production trajectories using the Woolf–Roux method[54,55]. The membrane permeability coefficient was then determined based on the inhomogeneous solubility–diffusion model[56]. This enables a direct comparison of the transport properties through the two membrane models.

Packmol[33] was used to build both model systems. LAMMPS[57] and PCFF-INTERFACE force field[58] were used to perform the molecular simulations. The PCFF INTERFACE force field can accurately describe both organic and inorganic components simultaneously, eliminating systematic biases in interfacial simulations that occur with traditional force fields. It is highly suitable for interfacial simulations, as reported in the literature[58–60]. The integration time step was fixed at 1.0 fs using the Verlet velocity algorithm, and the temperature was controlled by the Nosé-Hoover thermostat. The cut-offs for all the non-bonded interactions were 1.2 nm, and the PPPM method with a relative precision of 10^{-5} was used to account for the long-range electrostatic interactions.

Hydrogen bond characteristic simulation between functional groups and water

molecules. Molecular dynamics simulations were performed using the GROMACS software[34–37]. The force field was chosen to be OPLS-AA, consistent with the simulation system for functionalized graphene nanopores. The simulation box was filled with water using the TIP3P water model. Cl^- ions were added to neutralize the system charge, ensuring overall charge neutrality. Prior to the production simulations, energy minimization was performed to eliminate unreasonable contacts in the initial conformation. Subsequently, 100 ps of pre-equilibration simulation in the NVT ensemble and 100 ps of pre-equilibration simulation in the NPT ensemble were carried out. After the system was fully relaxed, production simulations were performed. The production simulations employed the leap-frog algorithm to integrate Newton's equations of motion, with a time step of 0.002 ps and a total of 50,000,000 steps, resulting in a total simulation time of 100.0 ns. Due to the use of a cutoff distance for short-range interactions, long-range electrostatic interactions were corrected using the PME method. For long-range van der Waals interactions, a dispersion correction was applied. Periodic boundary conditions were applied in all three directions.

Method for estimating the entropic penalty at the pore entrance

Following the method proposed by Jeffrey C. Grossman[64], we estimated the change in the entropic penalty for water molecules at the entrances of the PIP-TMC and QBTS-1 pores. Specifically, under the assumption that water transport through the nanopores follows Arrhenius model, the transport rate of water molecules can be written as follows:

$$k = A_0 \exp\left[-\frac{\Delta E + T\Delta S - \Omega P}{RT}\right] \quad (20)$$

Here, k denotes the transport rate of water molecules, which was estimated from the water displacement–time curves shown in Fig. 5g, with the region from 92 to 80 Å defined as the pore-entrance vicinity. A_0 is the pre-exponential factor. Ω represents the effective molecular volume, and P is the applied pressure; the product ΩP is regarded as the driving force for species transport through the pore. ΔE is the enthalpic barrier for species translocation through the pore, ΔS is the entropic barrier for species translocation through the pore, R is the ideal gas constant, and T is the absolute temperature in Kelvin.

By taking the ratio of the transport rates of water molecules through the two pore entrances, the ratio of the transport rates through the two pore entrances can be simplified as:

$$RT\ln\frac{k_{QBTS}}{k_{PIP-TMC}} = -T\left(\Delta S_{QBTS} - \Delta S_{PIP-TMC}\right) \quad (21)$$

where k_{QBTS} and $k_{\mathrm{PIP\text{-}TMC}}$ denote the transport rates of water molecules in the vicinity of the pore entrances of the QBTS and PIP-TMC channels, respectively. ΔS_{QBTS} and $\Delta S_{\mathrm{PIP\text{-}TMC}}$ represent the entropic barriers for water molecules passing through the pore-entrance regions of the QBTS and PIP-TMC channels, respectively. $-T(\Delta S_{\mathrm{QBTS}}-\Delta S_{\mathrm{PIP\text{-}TMC}})$ corresponds to the reduction in the entropic barrier at the QBTS pore entrance relative to that of the PIP-TMC channel.

The results show that compared with the PIP-TMC pore, the effective entropic penalty near the pore entrance of the QBTS pore is reduced by approximately 0.799 kcal mol^{-1}. This value is comparable to the entropic barrier change calculated using the Eyring equation from transition state theory, indicating that QBTS plays a significant entropic modulation role in the local water entry process at the pore entrance. This result is also consistent with our hypothesis that the polyhydroxy structure can provide more orientations for water molecules during transmembrane transport, thereby helping to reduce the entropic penalty of the water cluster transmembrane process and consequently promoting water transport.

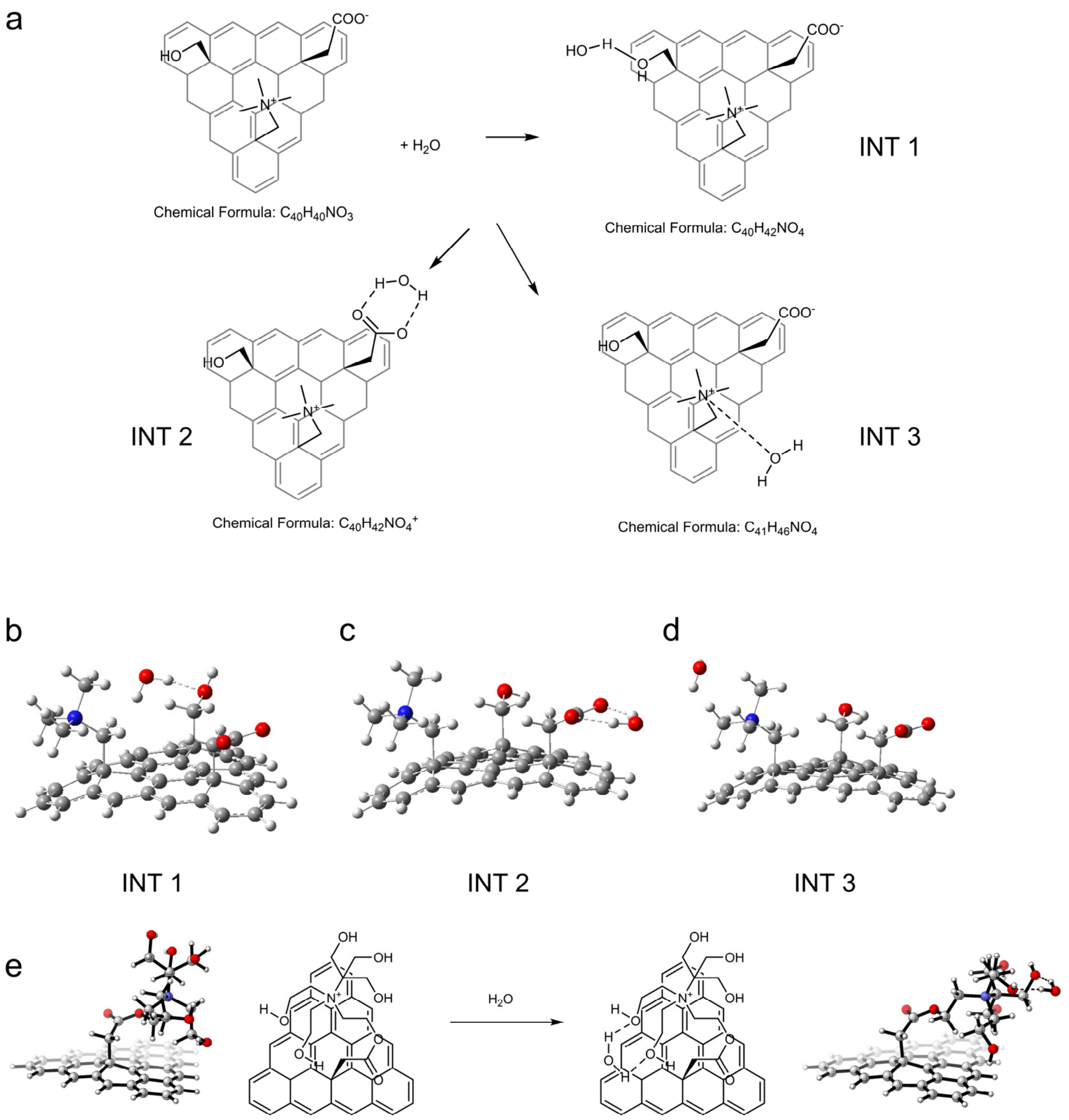

Supplementary Fig. 1. Interaction mode between the studied functional groups and water molecules for calculating the interaction energy. (a) Schematic diagram of the interactions between water molecules and different functional groups. Ball-and-stick representation showing interaction between water molecule and (b) -OH, (c) $-COO^-$ and (d) $-R_3N^+$. (e) Schematic diagram of the interaction between polyhydroxy quaternary ammonium functional group and water molecule. The illustration on the right shows the double-sites interaction between QBTS and water molecule. We grafted three functional groups onto an inert plane composed of nine benzene rings to investigate the interaction energies between the functional groups and water molecules. Colors of atoms: O, red; H, white; N, blue; C, grey.

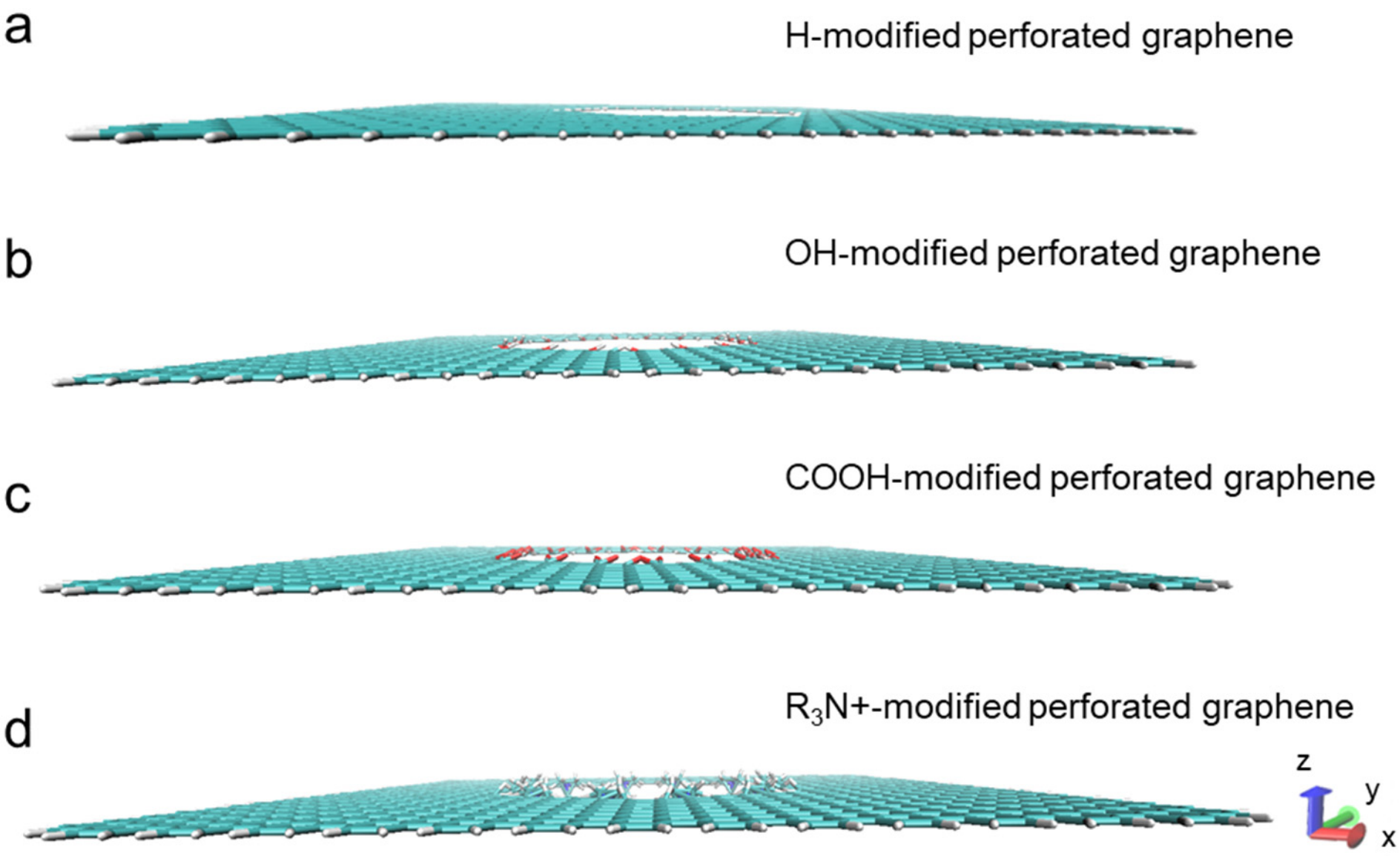

Supplementary Fig. 2. Front view of perforated graphene pore modified by (a) -H. (b) -OH. (c) -COOH and (d) -R_3N^+. We can see that the functional groups are not entirely located “inside the pore” but rather near the edge of the pore entrance. Colors of atoms: O, red; H, white; N, blue; C, cyan.

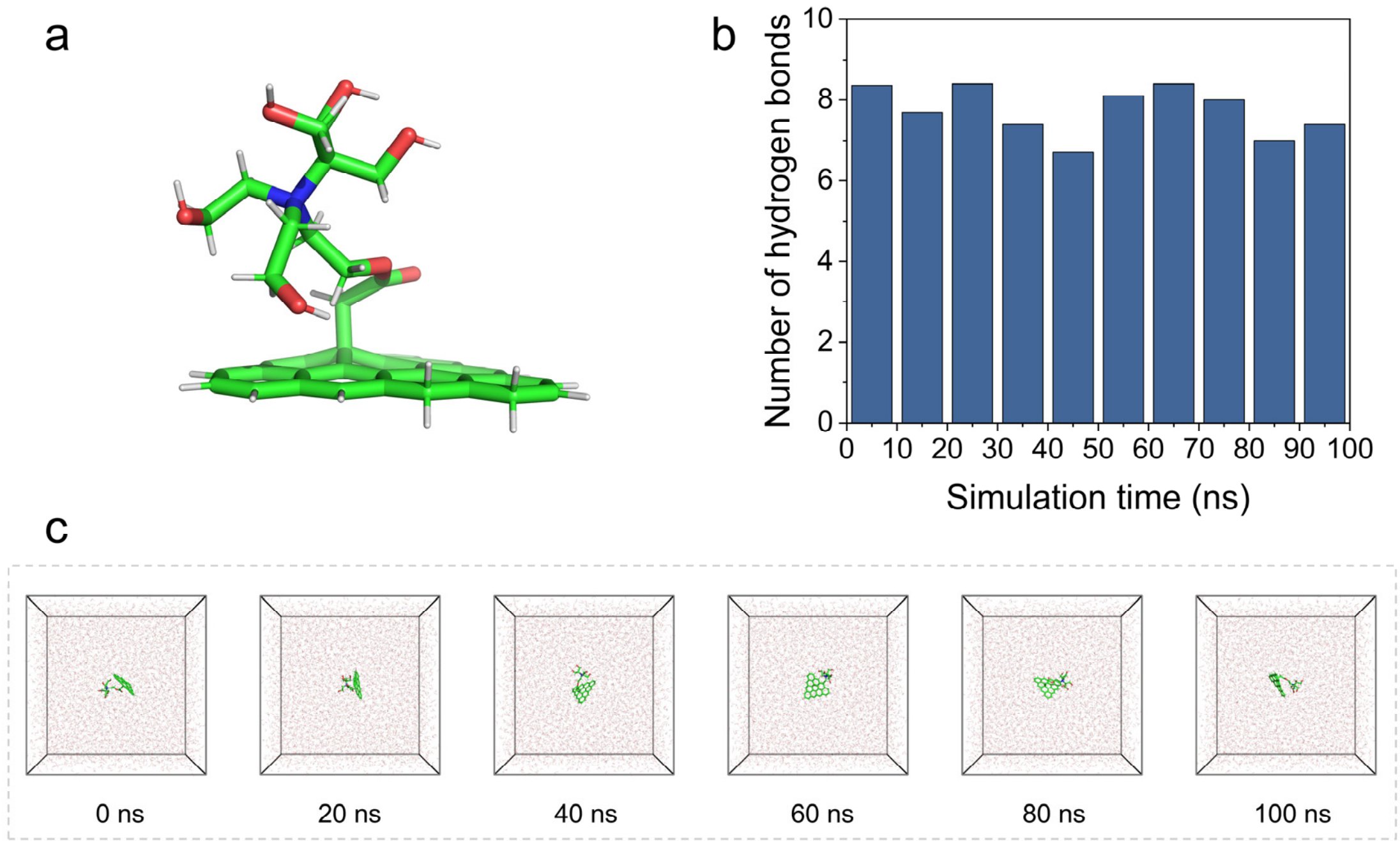

Supplementary Fig. 3. Simulation details for calculating H-bonds lifetime. (a) A graphene grafted by QBTS unit. (b) The variation of H-bonds number between QBTS and nearby water molecules as the simulation time increased. (c) The simulation aqueous box at different simulation time. Colors of atoms: O, red; H, white; N, blue; C, green.

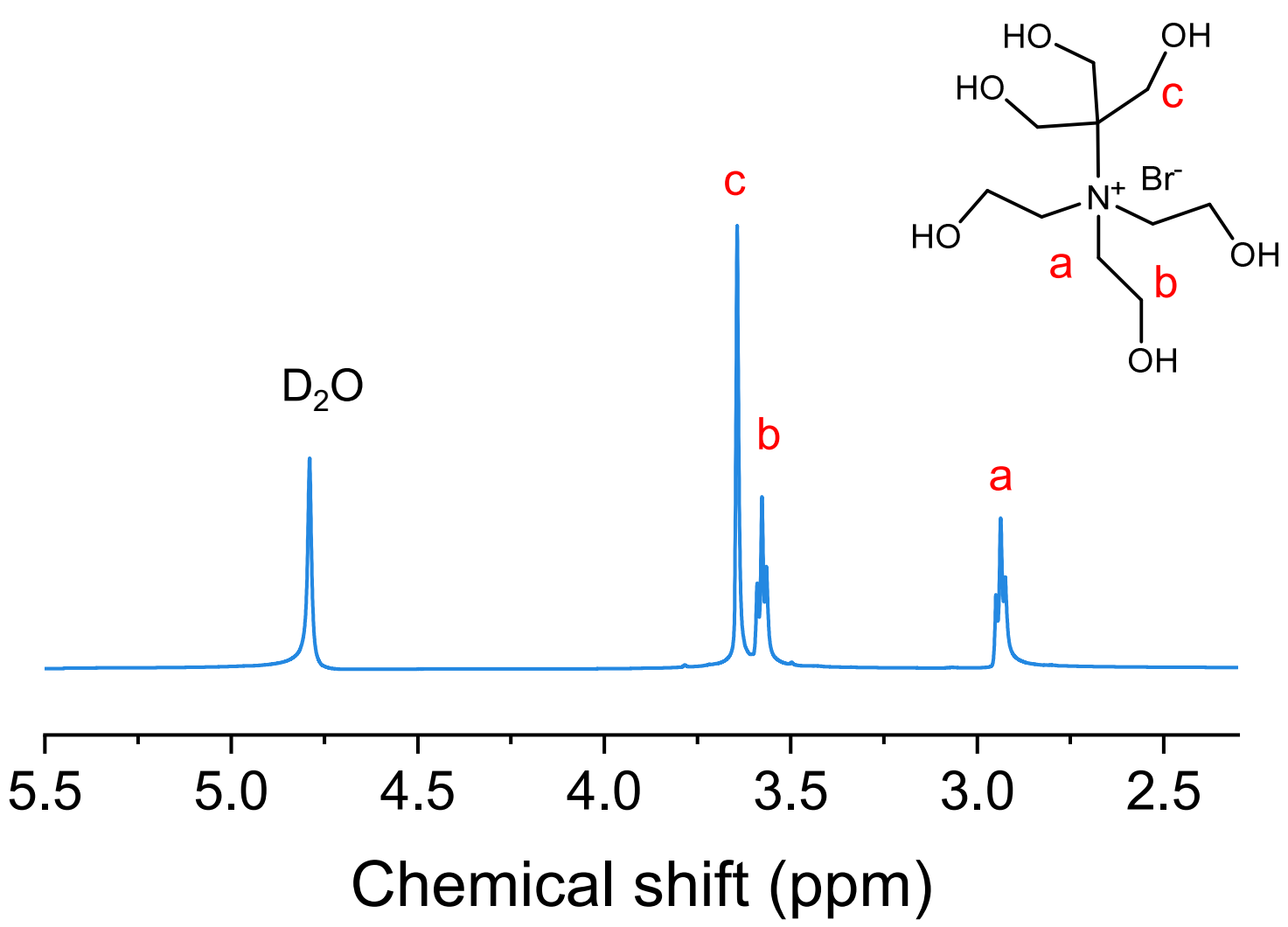

Supplementary Fig. 4. ^{1}H-NMR of QBTS molecules. Using D_2O as the solvent, the QBTS was dissolved in D_2O, and the NMR peaks of the three Hs corresponding to the QBTS structure are shown in the figure. Note: The H in the -OH can not be shown in D_2O.

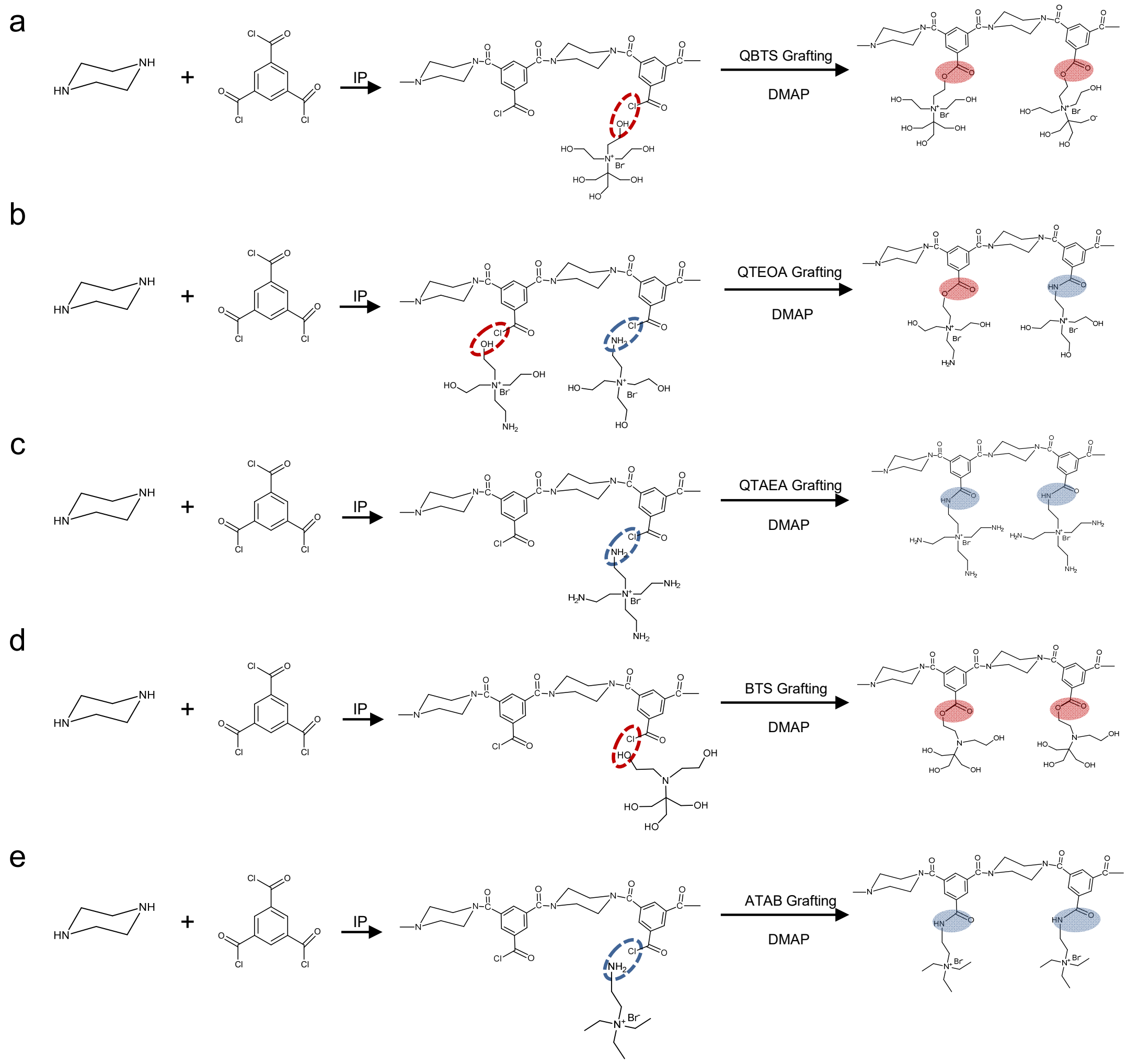

Supplementary Fig. 5. schematic illustration of the principle of membrane fabrication. (a)-(e) The main reaction process of interfacial polymerization (IP) and surface modification by QBTS, QTEOA, QTAEA, BTS, and ATAB, respectively. In this study, QBTS was grafted onto the membrane surface via an esterification reaction between its hydroxyl groups and the residual acyl chloride groups of TMC within the freshly formed polyamide active layer. This grafting strategy is widely employed for anchoring hydroxyl-containing functional groups onto polyamide membranes[65–68]. The process begins with the interfacial polymerization of PIP and TMC to generate a nascent polyamide active layer. Subsequently, under the catalysis of 4-dimethylaminopyridine (DMAP)—a nucleophilic acyl transfer catalyst[69,70]—the remaining acyl chloride groups of TMC undergo esterification with the hydroxyl groups of QBTS, thereby covalently attaching QBTS to the polyamide network. (b)-(e) show the reaction

principles of other monomers contain hydroxyl or primary amine groups for surface modification. The hydroxyl groups react with acyl chloride groups to form ester bonds, while the primary amine groups react with acyl chloride groups to form amide bonds[71–73]. The red and blue dashed lines indicate the functional groups involved in esterification and amidation reactions, respectively. The red and blue shaded regions denote the resulting ester and amide bonds, respectively.

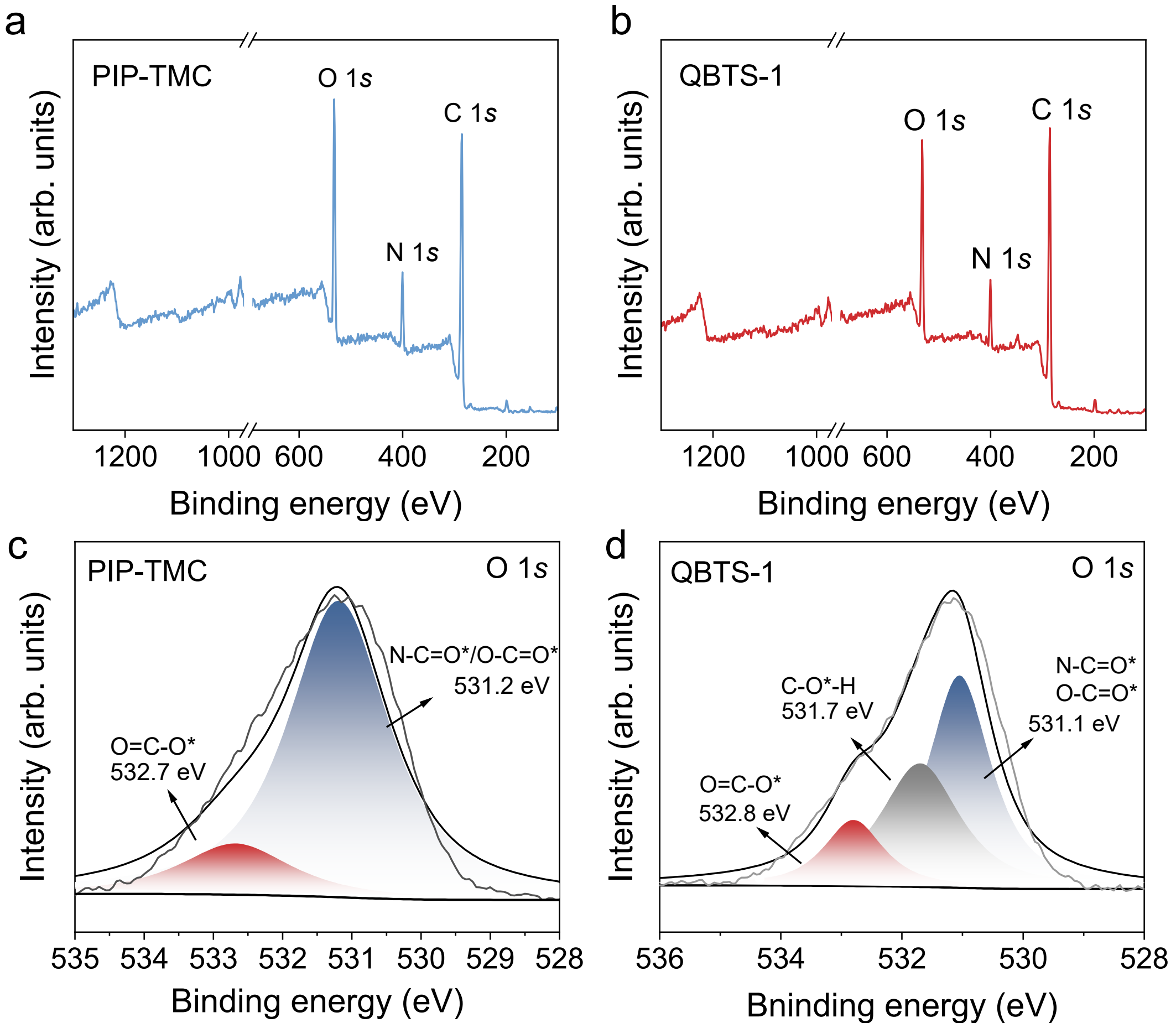

Supplementary Fig. 6. X-ray photoelectron spectra (XPS) of PIP-TMC and QBTS-1 membranes. (a)-(b) Survey XPS spectra of PIP-TMC and QBTS-1 membranes, respectively. (c)-(d) XPS O 1*s* analysis of PIP-TMC and QBTS-1 membranes, respectively. We found the characteristic polyamide structure, i.e., O=C-O*, N-C=O* and O-C=O*[65], in both the PIP-TMC and QBTS membranes. In addition, the new peak at 531.7 eV observed in the QBTS membrane corresponds to C-O*-H[74], which is a typical chemical feature of QBTS monomer.

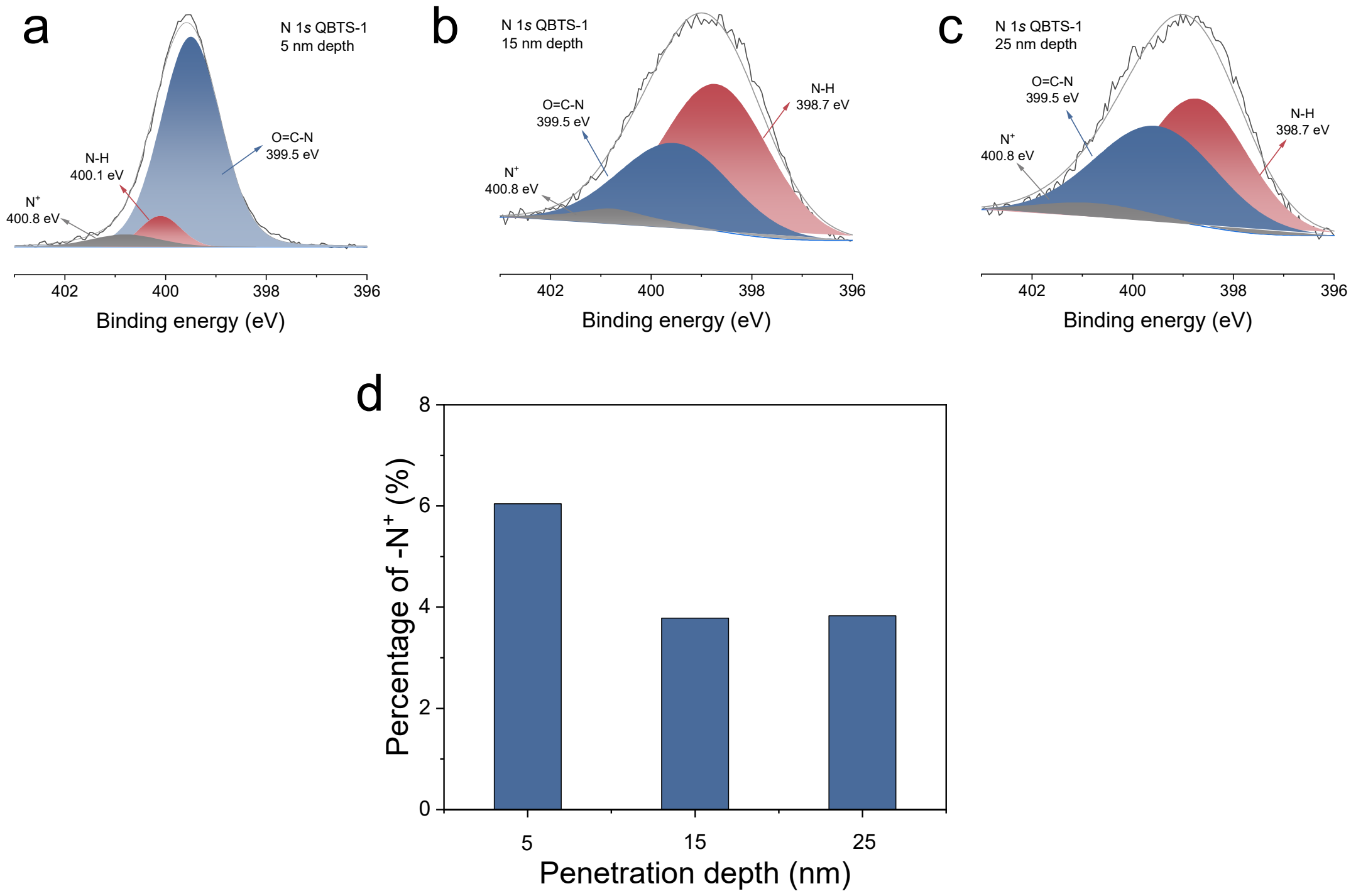

Supplementary Fig. 7. XPS depth profiling analysis of QBTS-1 membranes. (a)–(c) N 1*s* X-ray photoelectron spectra of QBTS-1 membranes at depths of 5 nm, 15 nm, and 25 nm, respectively. (d) Content of -N^+ species in the N 1*s* X-ray photoelectron spectra of QBTS at the corresponding depths. A certain amount of quaternary-N^+ was still observed at a depth of about 25 nm inside the pore. Based on this, it can be inferred that QBTS is distributed on the membrane surface and in the pores near the surface. Therefore, edge-functionalized perforated graphene model adopted in our study (Fig. 2) captures the main pore characteristics of the QBTS-modified membrane.

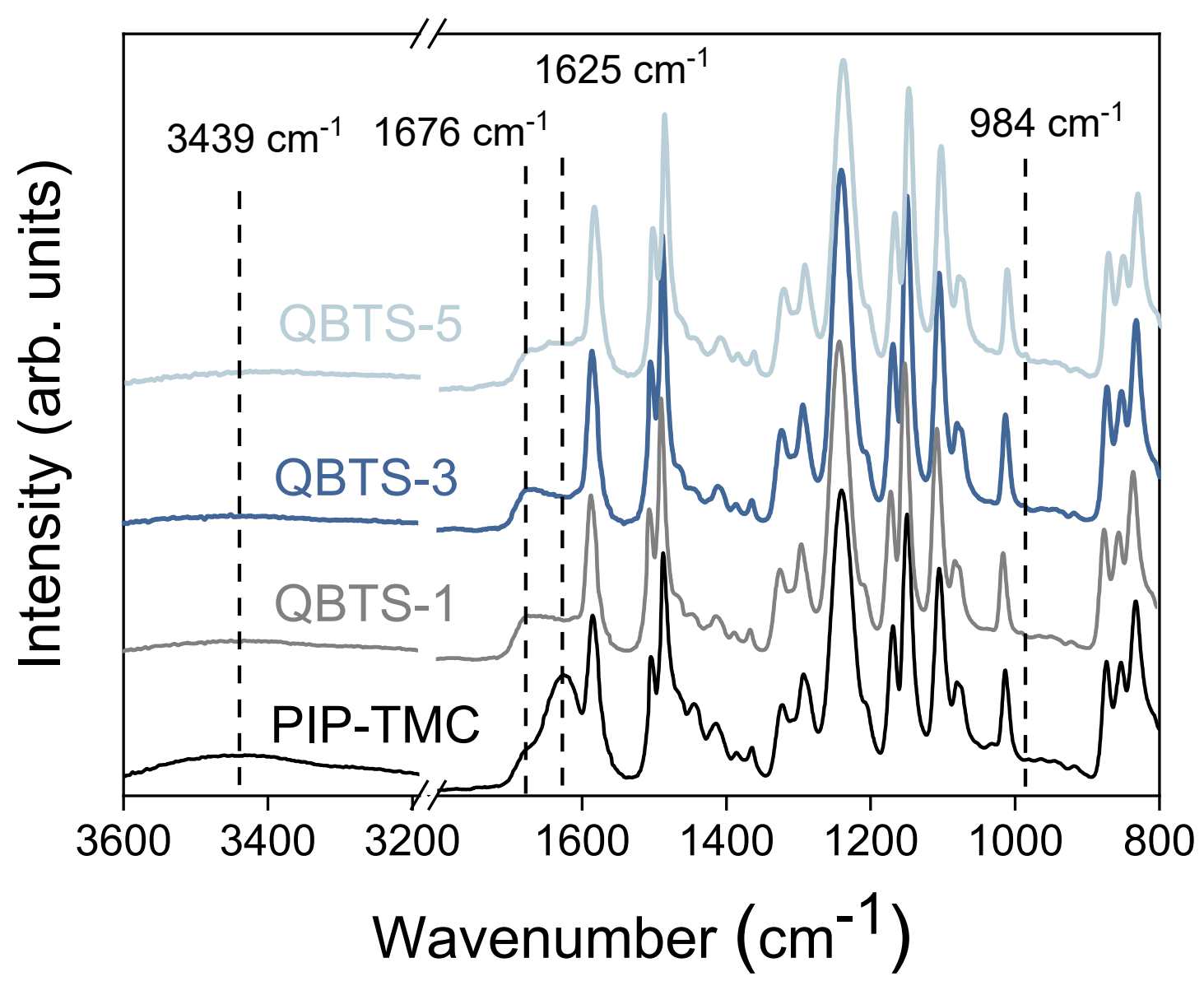

Supplementary Fig. 8. FTIR spectrum of PIP-TMC and QBTS modified membranes with different monomer concentrations. The new peak appeared in ~984 cm^{-1} is ascribed to N^+-$(CH_3)_4$[75,76]. The absorption peak at 1625 cm^{-1} corresponds to the C=O stretching vibration of the amide group[77], the absorption peak at 1676 cm-1 is the characteristic peak of the ester group[78], and the adsorption peak at 3439 cm^{-1} is attributed to the N-H bending vibration of the amide group[79]. The presence of N^+-$(CH_3)_4$ and ester groups proves the successful modification of PIP-TMC by QBTS monomers.

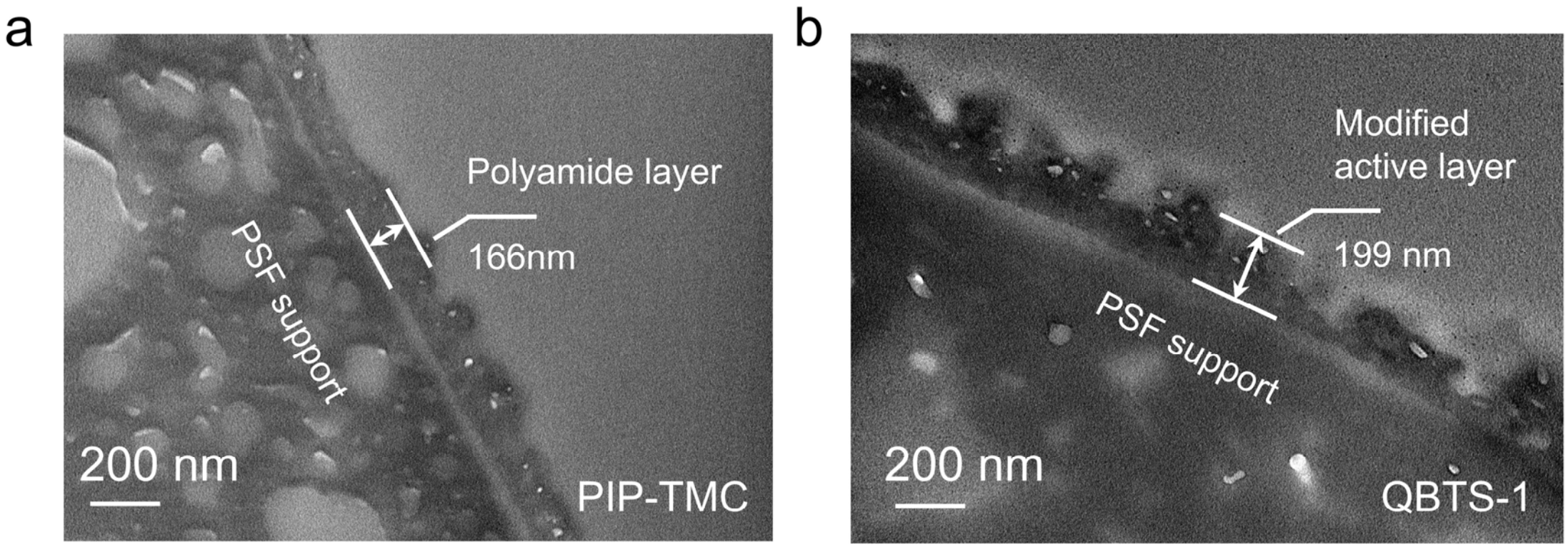

Supplementary Fig. 9. Transmission Electron Microscope (TEM) images of the cross section of PIP-TMC (a) and QBTS-1 (b). We found that the thickness of the PIP-TMC membrane was approximately 166 nm. After modification with QBTS, the thickness of modified active layer increased to approximately 199 nm. This observation is consistent with the results obtained from our SEM analysis (Fig. 2g).

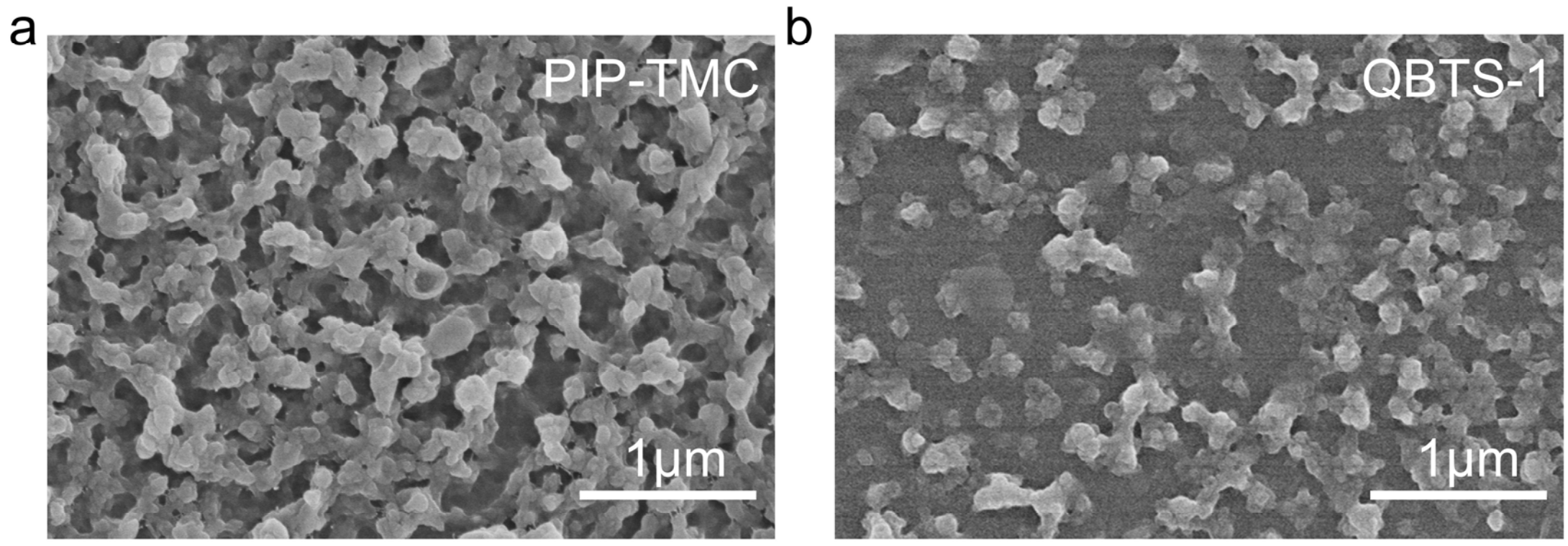

Supplementary Fig. 10. Scanning electron microscopy (SEM) images of surface morphologies of PIP-TMC (a) and QBTS-1 (b). As shown in the Supplementary Fig. 4, nodular structures can be observed on the membrane surface, which typically result from diffusion-driven instability during the interfacial polymerization reaction and generating nanoscale local inhomogeneities due to locally intense and heterogeneous reactions during the interfacial polymerization process[80]. Such surface morphology helps increase the water transport pathways and thereby facilitates water permeation. Since QBTS molecules are relatively small in size, the modified membrane exhibits nodular structures similar to that of the PIP-TMC membrane[81]. In addition, due to the grafting reaction between the QBTS molecules and the nascent polyamide layer, the edges of these nodules appear relatively smoother in QBTS-1[82].

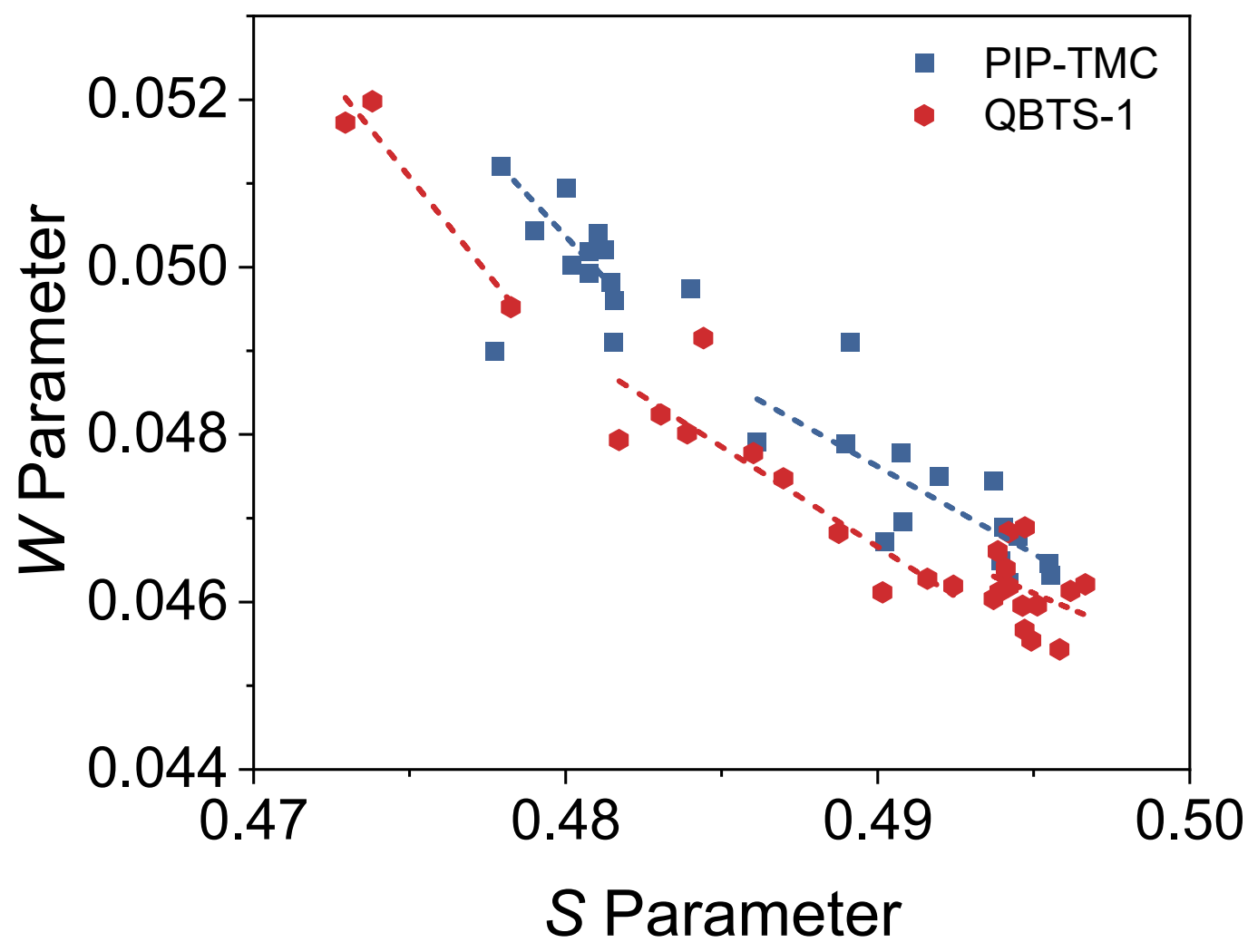

Supplementary Fig. 11. The *S*-*W* plot of PIP-TMC and QBTS-1 membranes. The lines were from linear regressions fitting the data into different regions and the intercept of the line indicates a significant transition from one layer to the other. For the PIP-TMC, i.e., blue lines from left to right, it shows the transition from the PA layer to the PSF supporting layer. For the QBTS-1, i.e., red lines, it shows the transition from QBTS modified layer to PA layer and then to the PSF supporting layer.

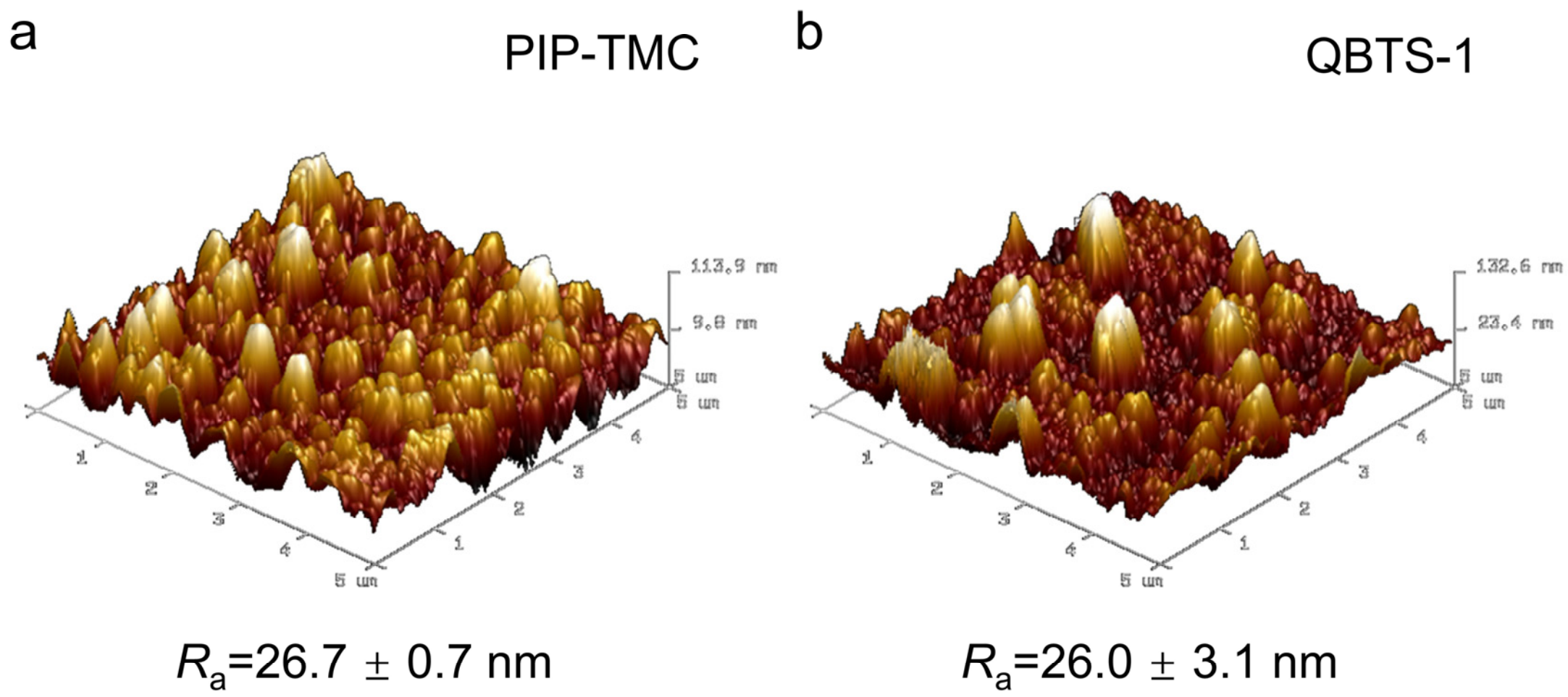

Supplementary Fig. 12. AFM surface morphologies of (a) PIP-TMC membranes and (b) QBTS-1 membranes. The surface roughness of PIP-TMC and QBTS-1 membranes are determined to be 26.7 ± 0.7 nm and 26.0 ± 3.1 nm, respectively, showing comparable values.

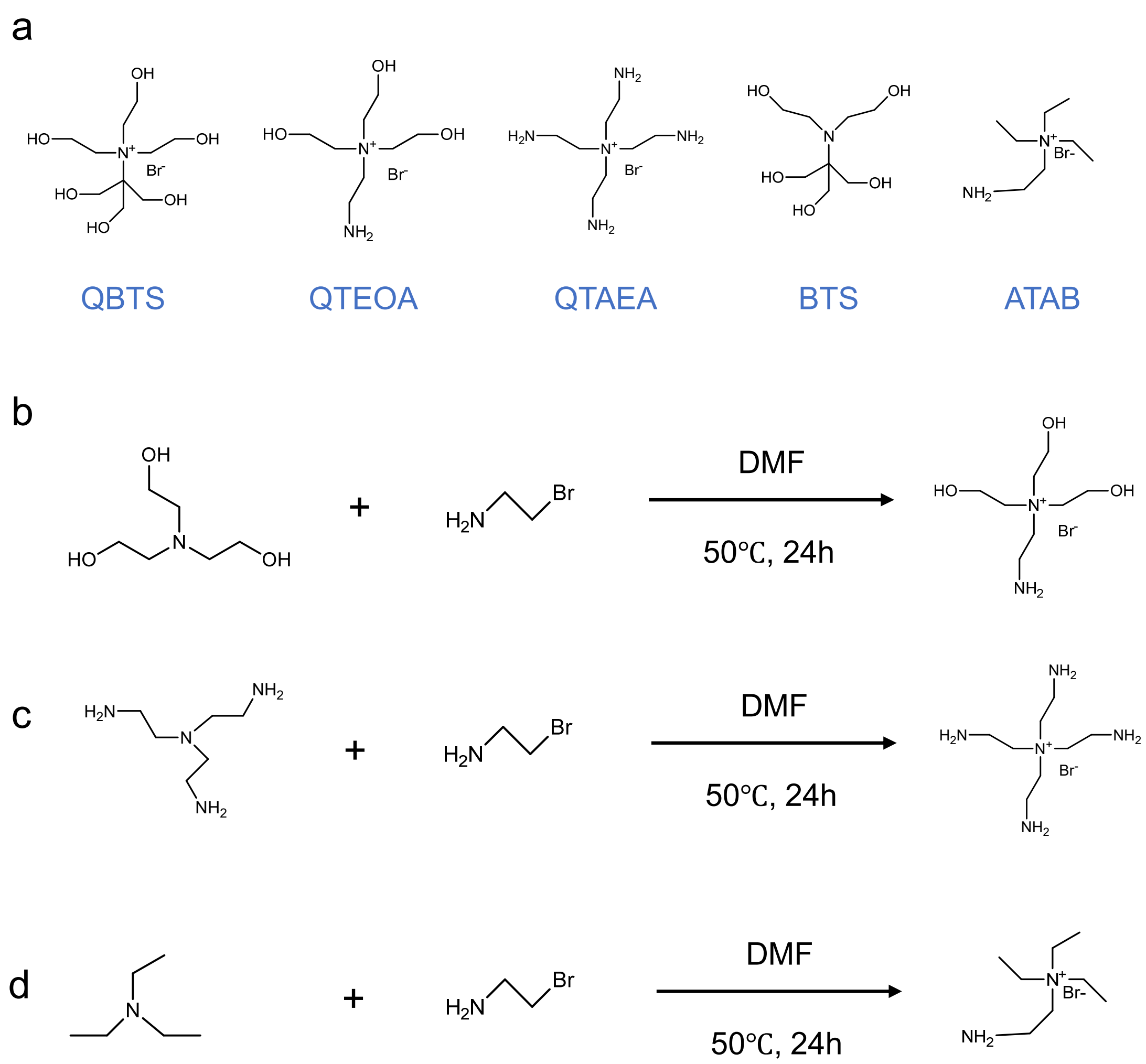

Supplementary Fig. 13. Schematic diagram of modified monomer structure and synthesis process. (a) Chemical structure of monomers used to modify the surface of the PIP-TMC membranes. (b)-(d) Synthesis reaction of the QTEOA, QTAEA, and ATAB monomers.

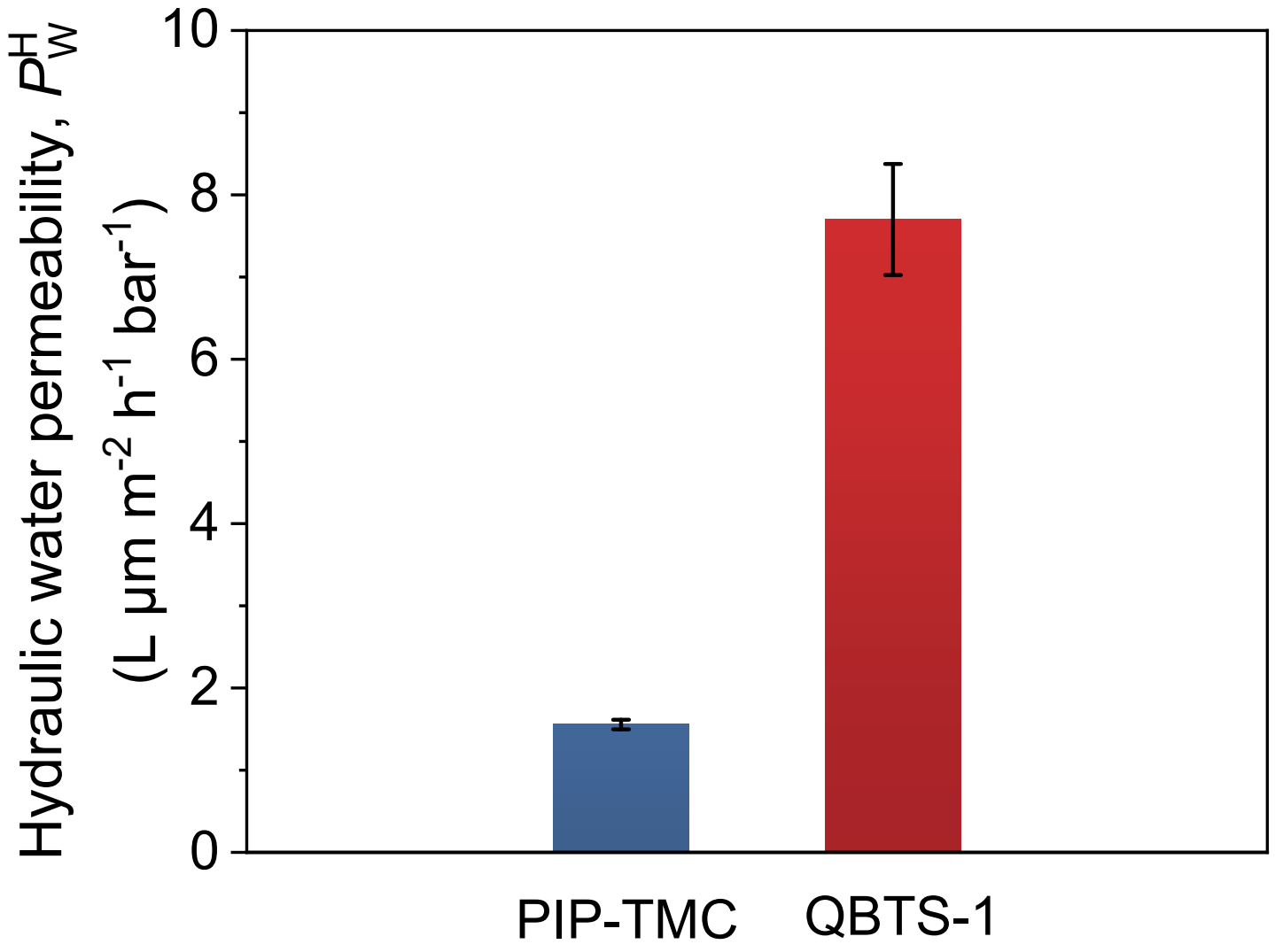

Supplementary Fig. 14. The hydraulic water permeabilities P_w^H of the PIP-TMC and QBTS membranes. P_w^H is a thickness-independent property that is governed by the physical confinement and chemical interactions experienced by water during transmembrane transport[83]. The P_w^H can be calculated by：

$$P_w^H = \frac{J_w \times \delta}{(\Delta P - \Delta \pi)}$$

where J_w is the water permeance, ΔP is the driven pressure, $\Delta \pi$ is theosmotic pressure. The P_w^H for QBTS (7.7 L μm·m^{-2}·h^{-1}·bar^{-1}) is much higher than that for the unmodified PIP-TMC (1.6 L μm·m^{-2}·h^{-1}·bar^{-1}). Given that key parameters such as pore size distribution, specific surface area, and surface roughness remain comparable between PIP-TMC and QBTS (Fig. 3d, 3e, 3g and 3h), this indicates that the increase in water flux of QBTS is mainly contributed by the chemical characteristics of the membrane surface. For data presented with error bars, data are presented as mean ± standard deviation (SD) from at least three replicate measurements.

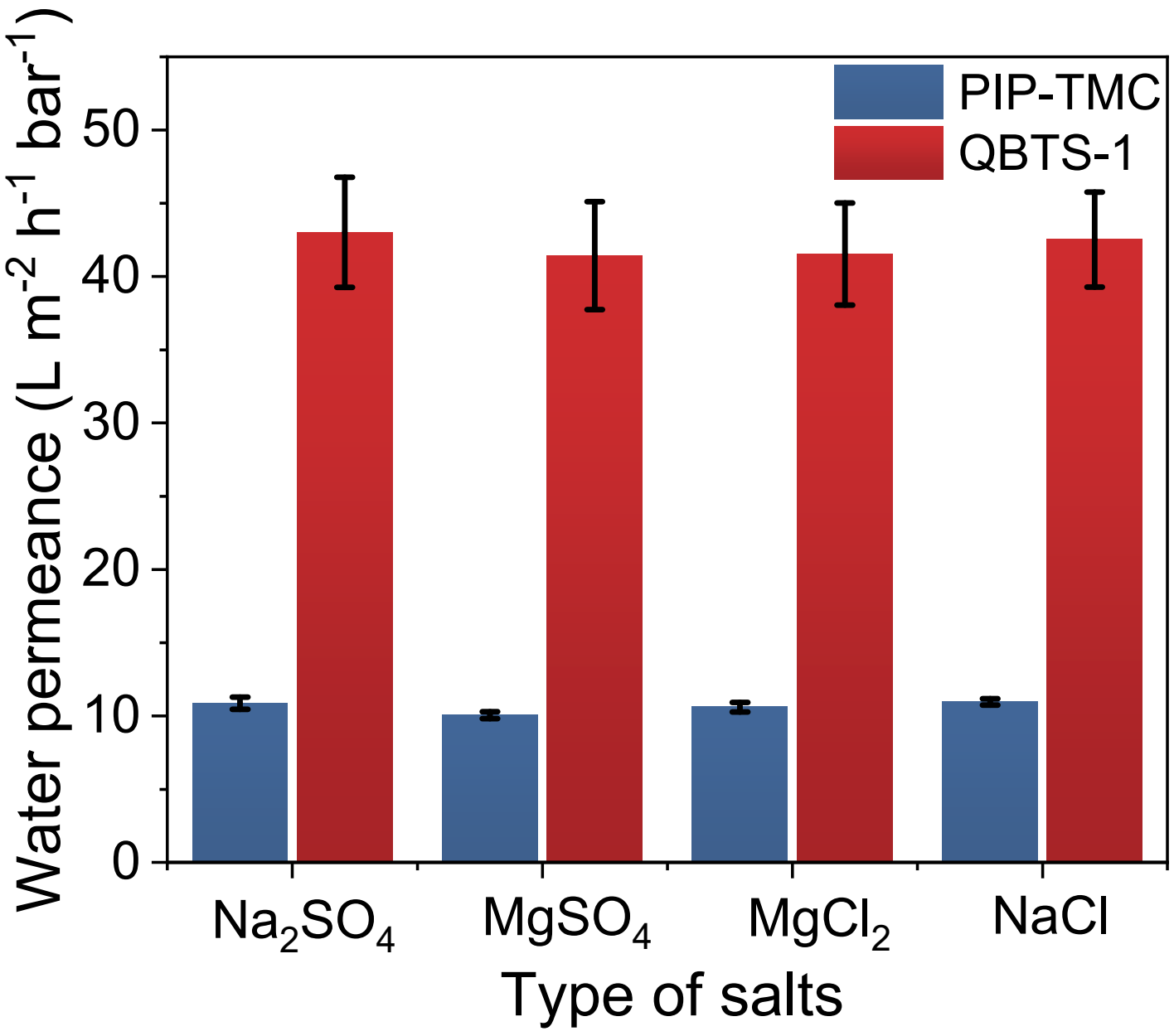

Supplementary Fig. 15. Water permeance of Na_2SO_4, $MgSO_4$, $MgCl_2$, and NaCl by PIP-TMC and QBTS-1 membranes (Salt concentration, 1000 ppm; applied pressure, 6 bar; temperature, 25 °C). For data presented with error bars, data are presented as mean ± standard deviation (SD) from at least three replicate measurements.

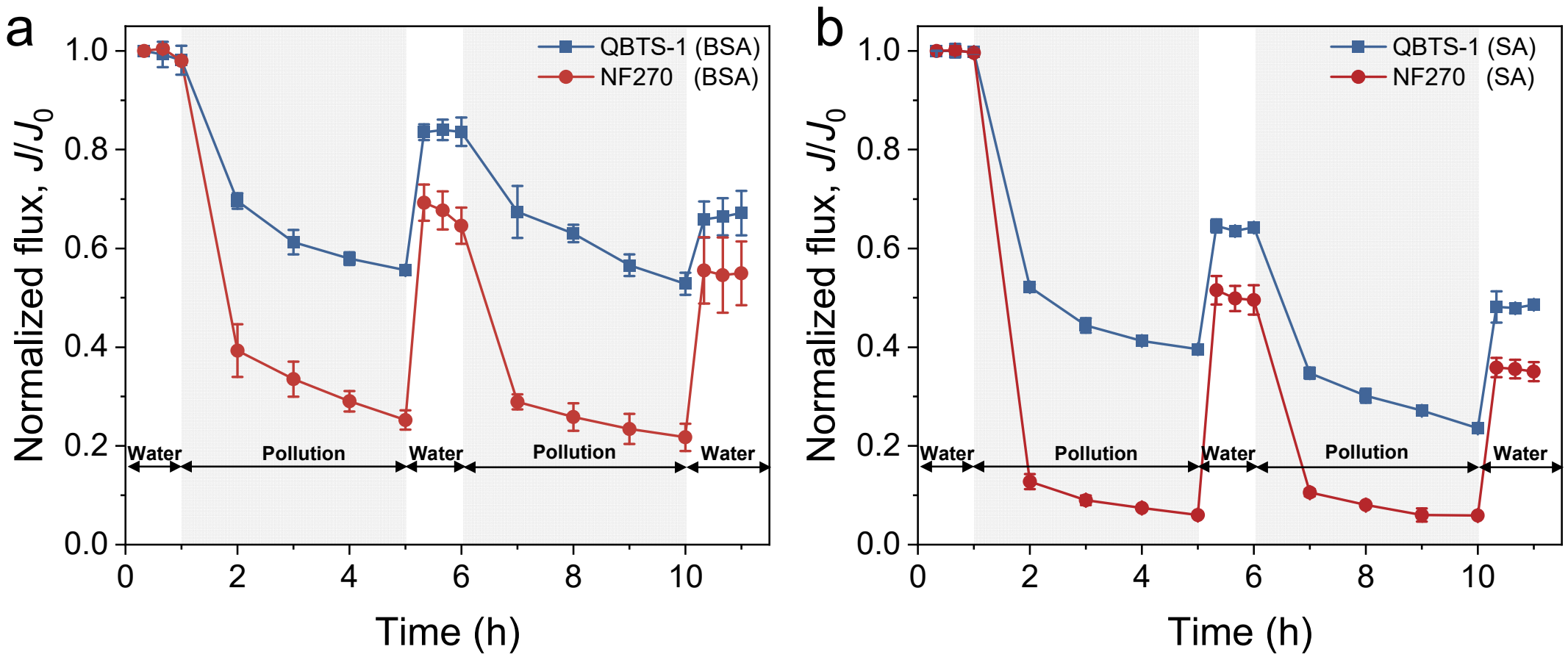

Supplementary Fig. 16. Analysis of the antifouling performance of QBTS compared with the commercial NF membrane NF 270. (a) Foulant: bovine serum albumin (BSA). (b) Foulant: sodium alginate (SA). The grey shaded regions indicate the membrane fouling process. For data presented with error bars, data are presented as mean ± standard deviation (SD) from at least three replicate measurements.

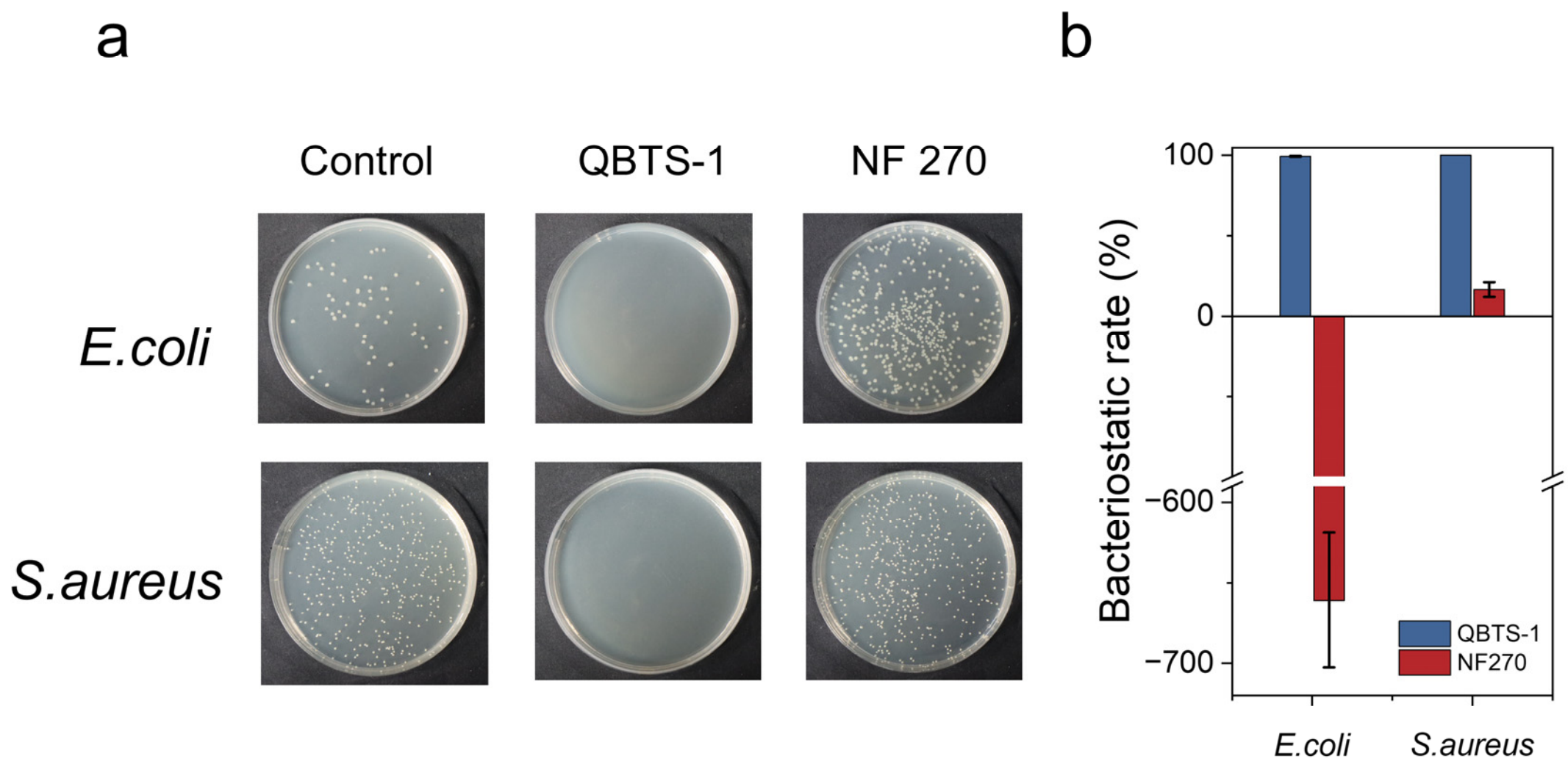

Supplementary Fig. 17. Comparison of the antibacterial performance of the two nanofiltration membranes. (a) Optical images of *E. coli* and *S. aureus* after 18 h of cultivation (left: membrane-free control; middle: QBTS-1 membrane; right: NF270 membrane). (b) Antibacterial rates of the QBTS-1 and NF270 membranes against the two bacterial strains relative to the membrane-free control. For data presented with error bars, data are presented as mean ± standard deviation (SD) from at least three replicate measurements.

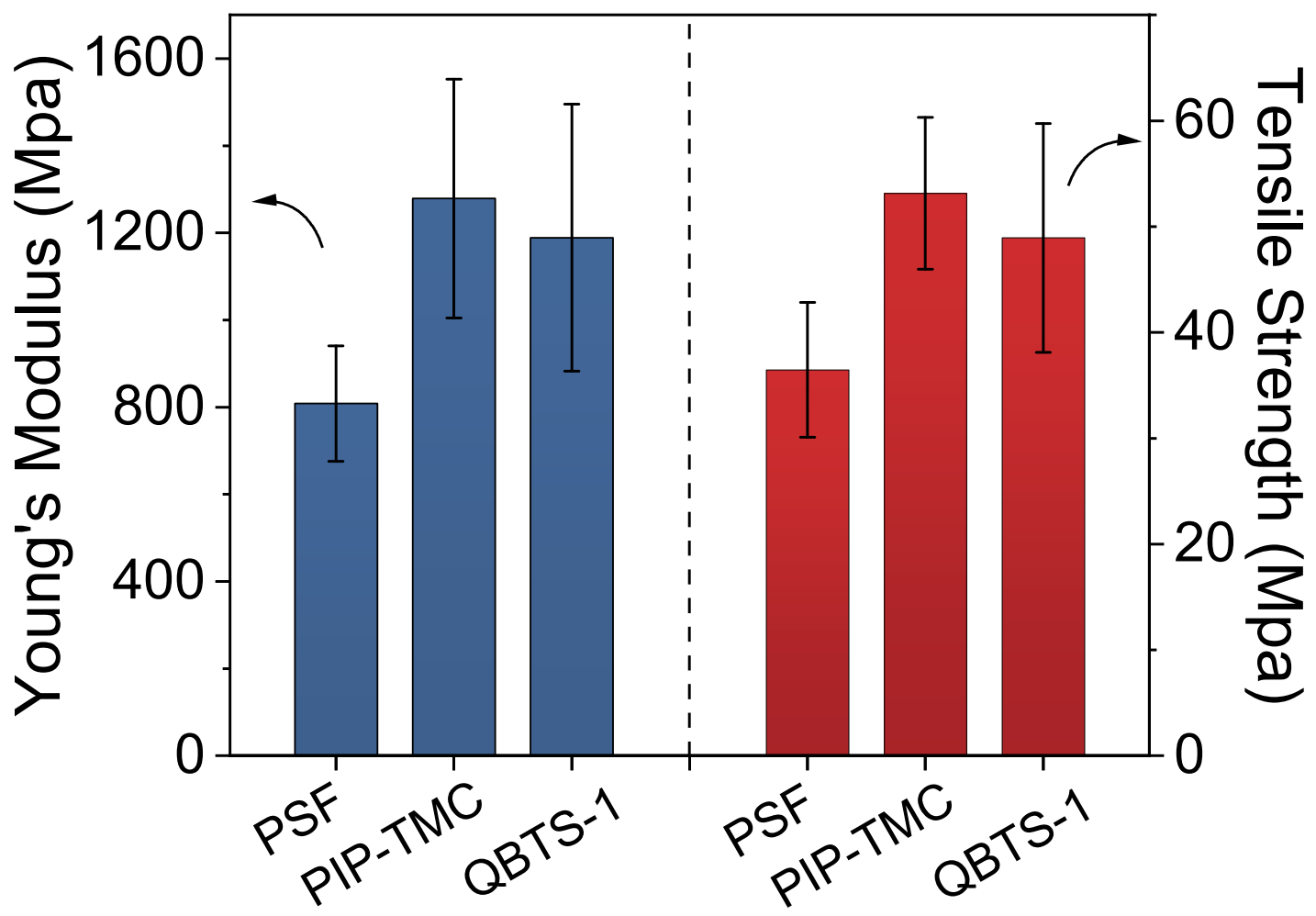

Supplementary Fig. 18. Mechanical performance of PSF, PIP-TMC and QBTS-1 membranes. Compared to the PSF supporting membrane, the mechanical strength of PIP-TMC and QBTS-1 membranes increase, which can be attributed to the formation of the dense nanofiltration layer above the PSF supporting membrane. In the meanwhile, the two types of nanofiltration membranes have similar mechanical performance. For data presented with error bars, data are presented as mean ± standard deviation (SD) from at least three replicate measurements.

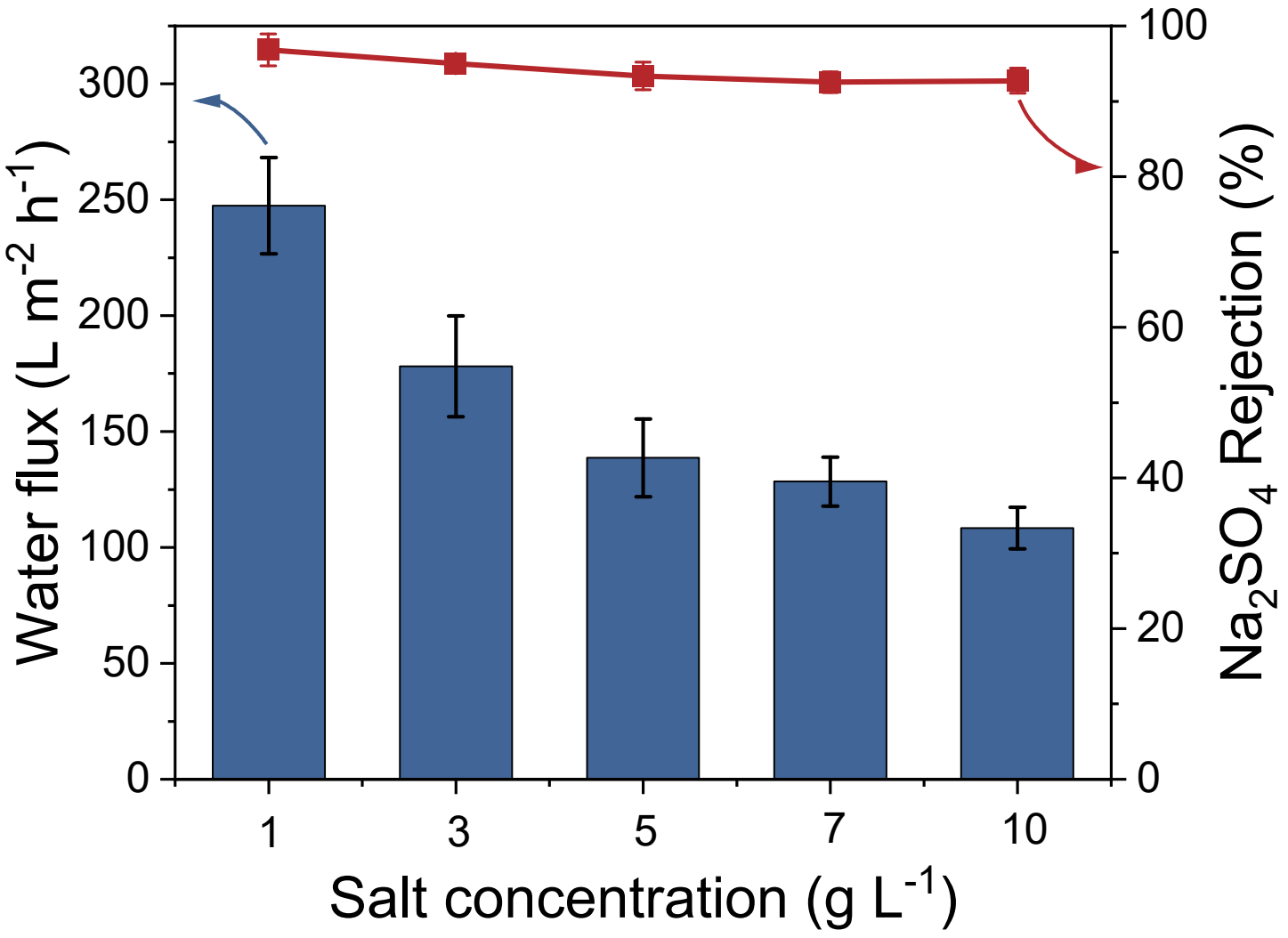

Supplementary Fig. 19. The filtration performance of QBTS-1 under different salt concentration. For data presented with error bars, data are presented as mean ± standard deviation (SD) from at least three replicate measurements.

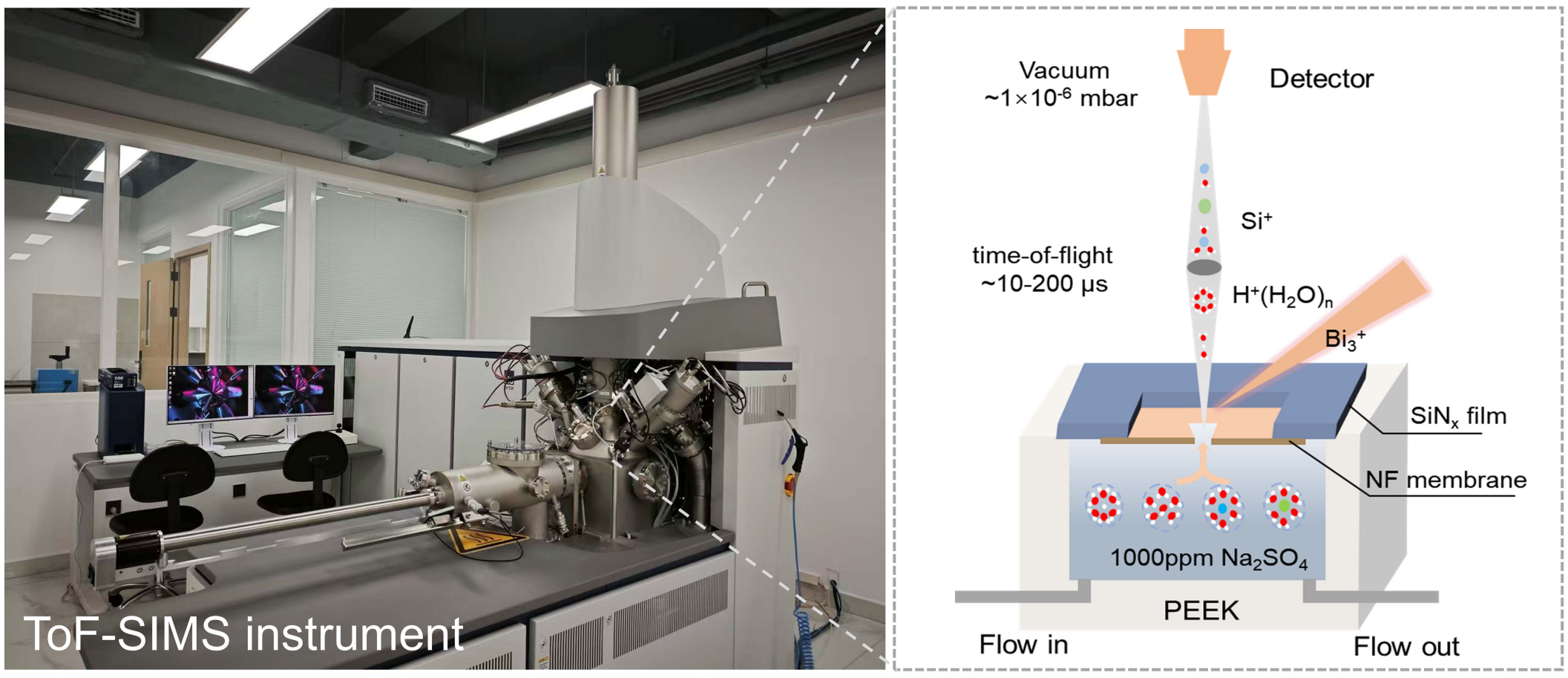

Supplementary Fig. 20. Schematic diagram of liquid ToF-SIMS. Left: ToF-SIMS instrument; Right: microfluidic filtration device placed in the ToF-SIMS vacuum chamber. PEEK denotes polyether ether ketone. Colors: O, red; H, white; ions, blue and green.

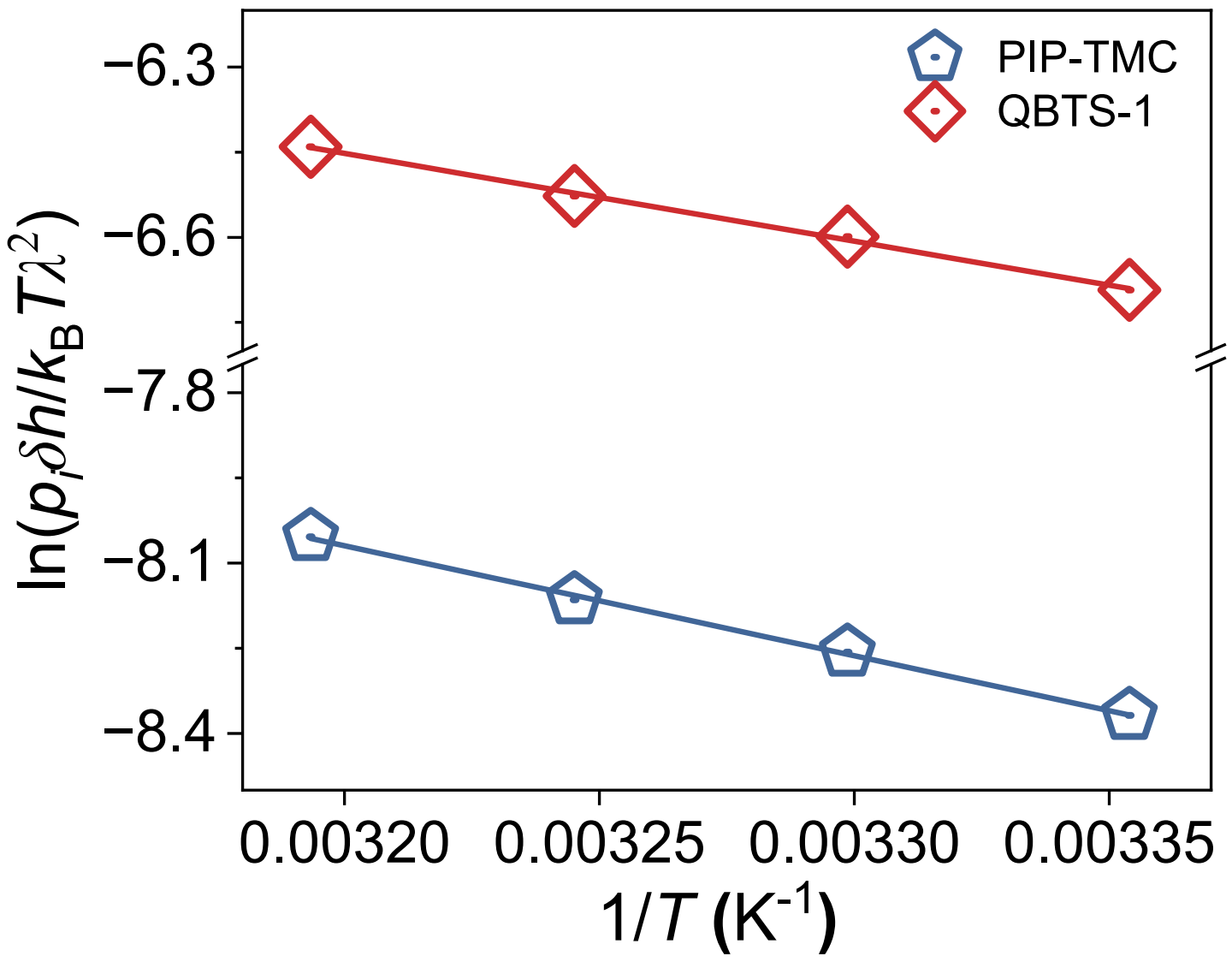

Supplementary Fig. 21. Fitting curves for water transport through PIP-TMC/QBTS-1 membranes according to Eyring-type equation. The fitted line in the graph represents one set of experiments in the parallel experiment.

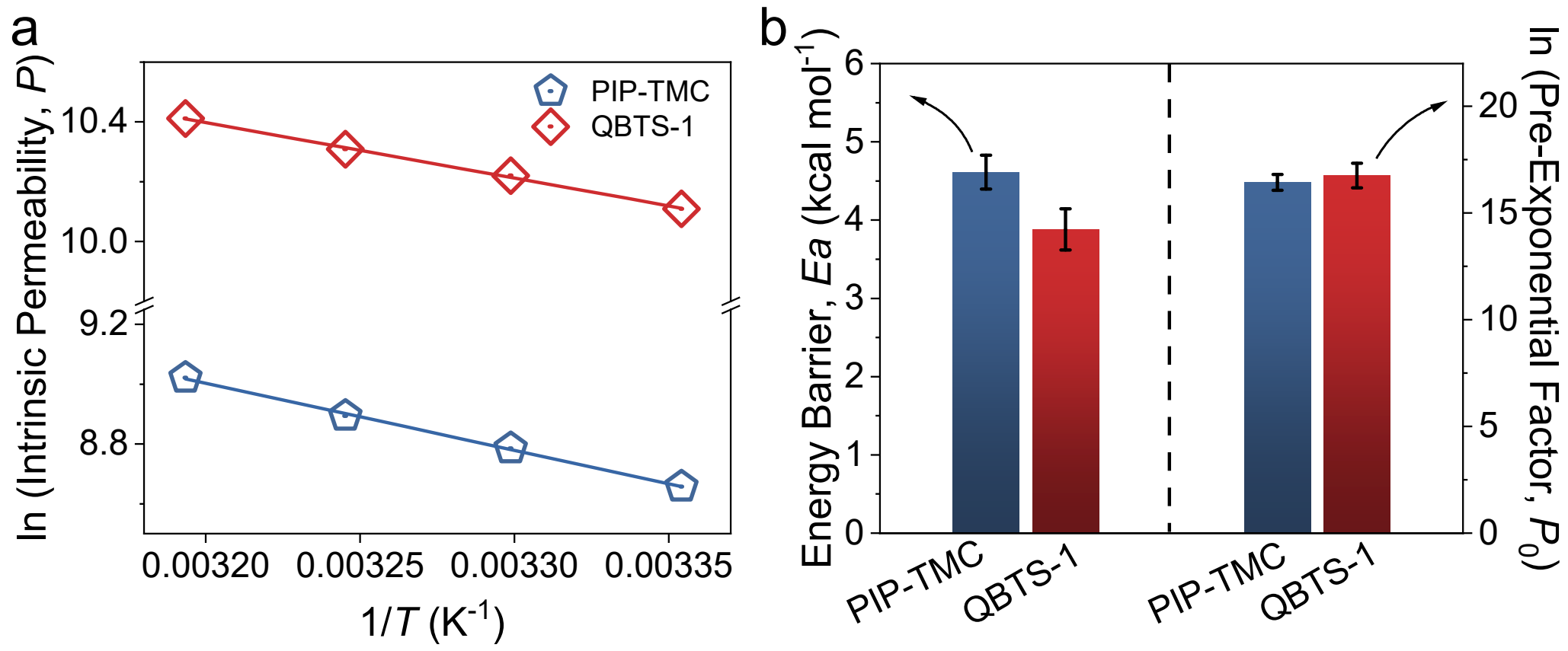

Supplementary Fig. 22. Energy barrier of water transmembrane transport determining using Arrhenius equation. (a) Arrhenius plots of the water permeabilities for 25, 30, 35, and 40°C. The effective energy barriers (E_a) and pre-exponential factor (P_0) were obtained from the slope and intercept of the curves, respectively. (b) Effective energy barriers and Pre-exponential factors for water transmembrane transport (Feed solution: 1000 ppm Na_2SO_4 at 6bar). For data presented with error bars, data are presented as mean ± standard deviation (SD) from at least three replicate measurements.

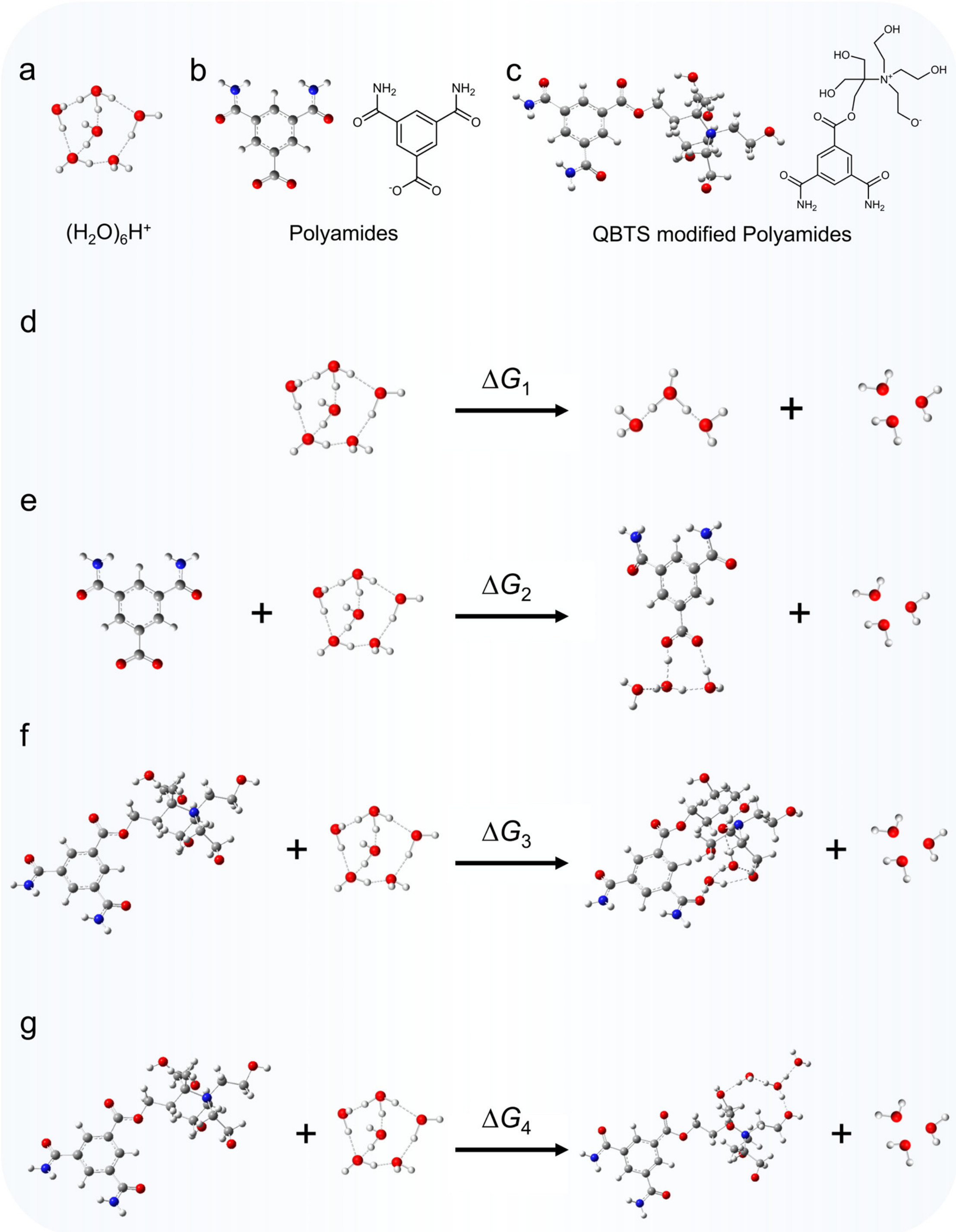

Supplementary Fig. 23. Schematic of the dehydration process from $(H_2O)_6H^+$ to $(H_2O)_3H^+$ in different pathways. Schematic of the representative molecules involved in the dehydration process, i.e., (a) water clusters $(H_2O)_6H^+$, (b) representative polyamide segment, and (c) representative QBTS modified polyamide segment. (d) Dehydration process from $(H_2O)_6H^+$ to $(H_2O)_3H^+$ without the participation of membrane segments, ΔG_1. (e) Dehydration process from $(H_2O)_6H^+$ to $(H_2O)_3H^+$ with the participation of

representative polyamide segment, ΔG_2. (f) Dehydration process from $(H_2O)_6H^+$ to $(H_2O)_3H^+$ with the participation of representative QBTS modified polyamide segment, ΔG_3, water clusters interact with both the polyamide and QBTS segments. (g) Dehydration process from $(H_2O)_6H^+$ to $(H_2O)_3H^+$ with the participation of representative QBTS modified polyamide segment, ΔG_4, water clusters interact exclusively with QBTS segments. Colors of atoms: O, red; H, white; N, blue; C, grey.

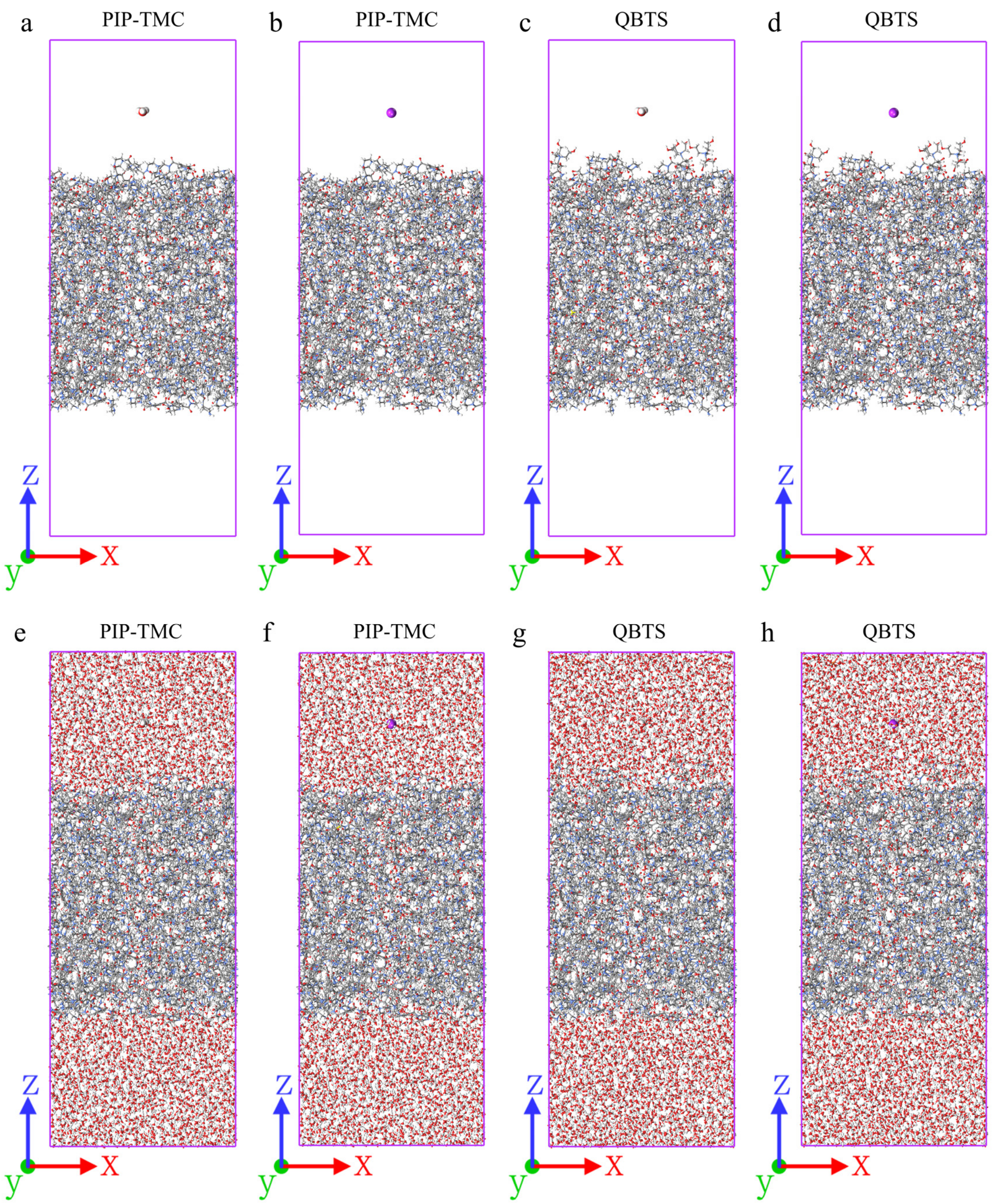

Supplementary Fig. 24. Molecular dynamics (MD) simulation platform of the polyamide nanochannels for investigating water and ion transport. Initial configurations showing (a)-(b) polyamide simulated nanochannels (PIP-TMC) for investigating hydronium ion and Cl^-, respectively. (c)-(d) QBTS modified polyamide simulated nanochannels for investigating hydronium ion and Cl^-, respectively. The highly cross-linked and porous polymeric membrane was simplified to a nanochannel composed of polyamide units. The cross-sectional illustration of the simulation platform showing (e)-(f) PIP-TMC nanochannel and (g)-(h) QBTS modified polyamide simulated

nanochannels immersed in containers filled with water molecules to investigate hydration number, water diffusion velocities, and PMF during transmembrane transport. Colors: O, red; C, grey; H, white; N, blue; Cl, purple; QBTS was grafted in pore entrance. As shown in Fig. 4e, Fig. 4f, Fig. 5f, Fig. 5g and Supplementary Fig. 25, we define the entrance of the nanochannels located ~92-80 Å along Z direction. This statistical range was used for analyzing the hydrogen bond/hydration number, diffusion coefficient and PMF.

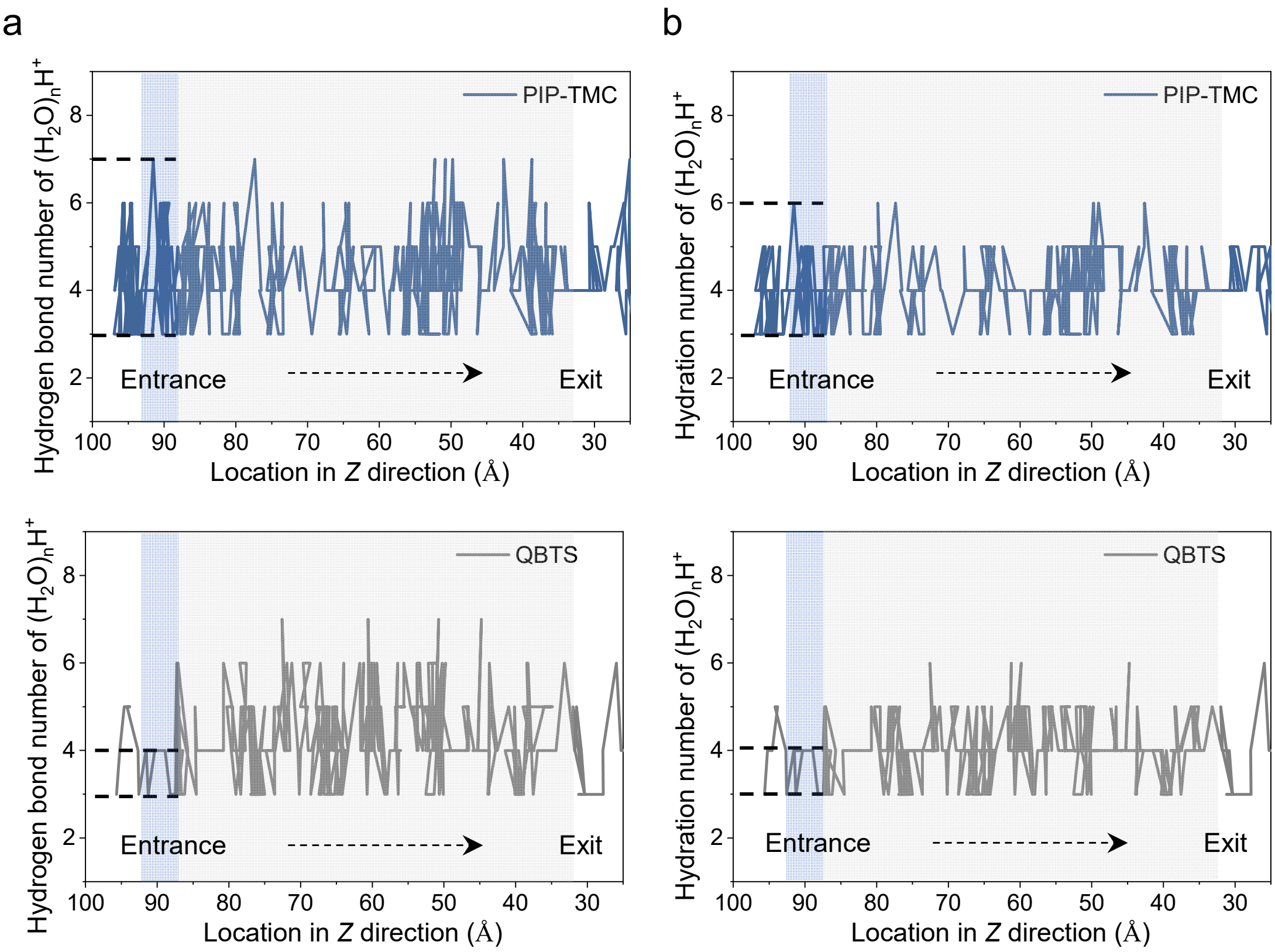

Supplementary Fig. 25. The change in hydration state of water clusters during transmembrane transport through the PIP-TMC and QBTS grafted nanochannels. The hydrogen bond number (a) and hydration number (b) of $(H_2O)_nH^+$. The blue shaded region indicates the pore entrance, while the blue and grey shaded regions together represent the polyamide nanochannel region. The dashed boxes highlight the corresponding values at the entrance region.

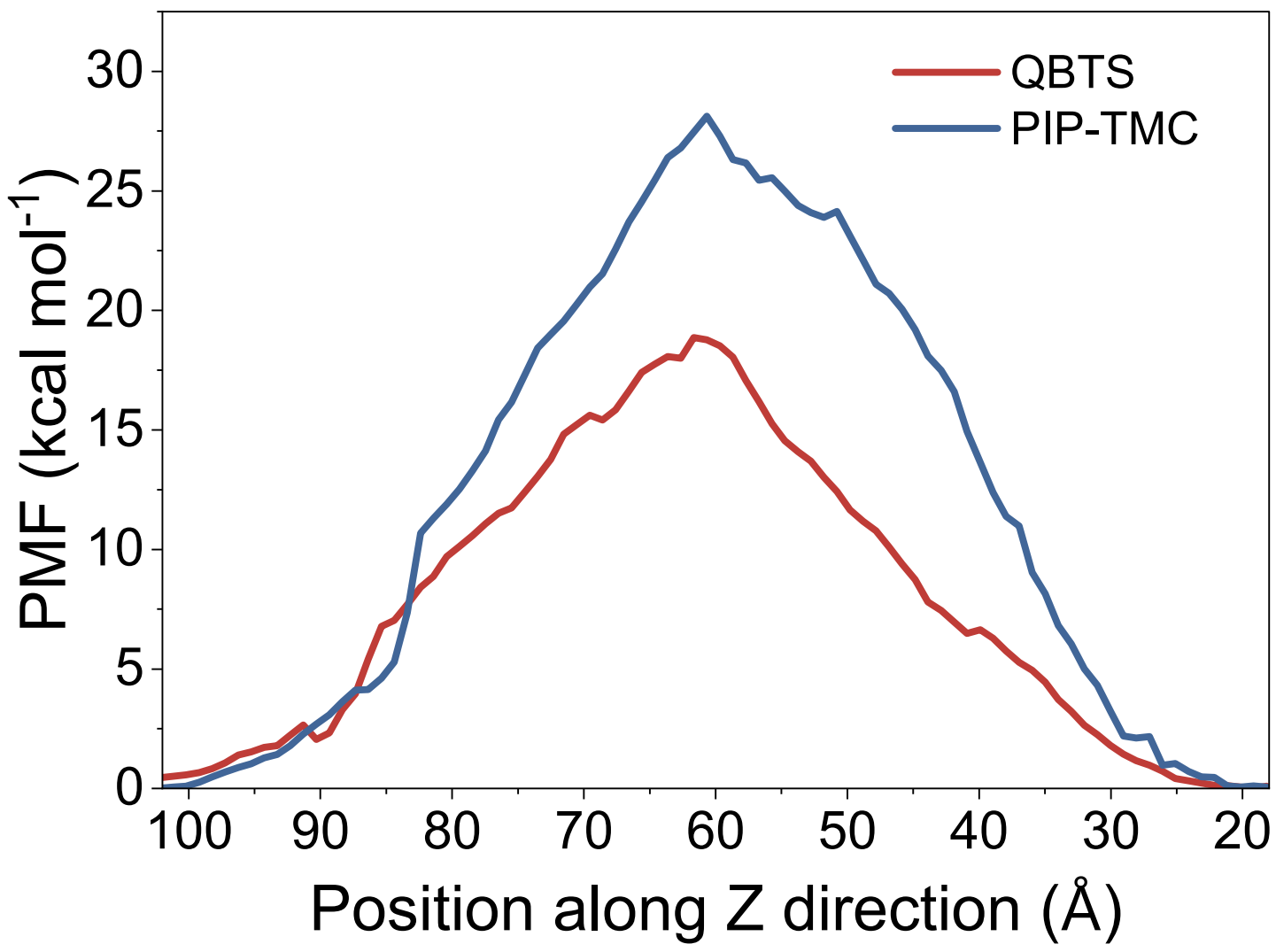

Supplementary Fig. 26. The potential of mean force (PMF) of H_2O along the diffusion coordinate in PIP TMC and QBTS simulated nanochannels.

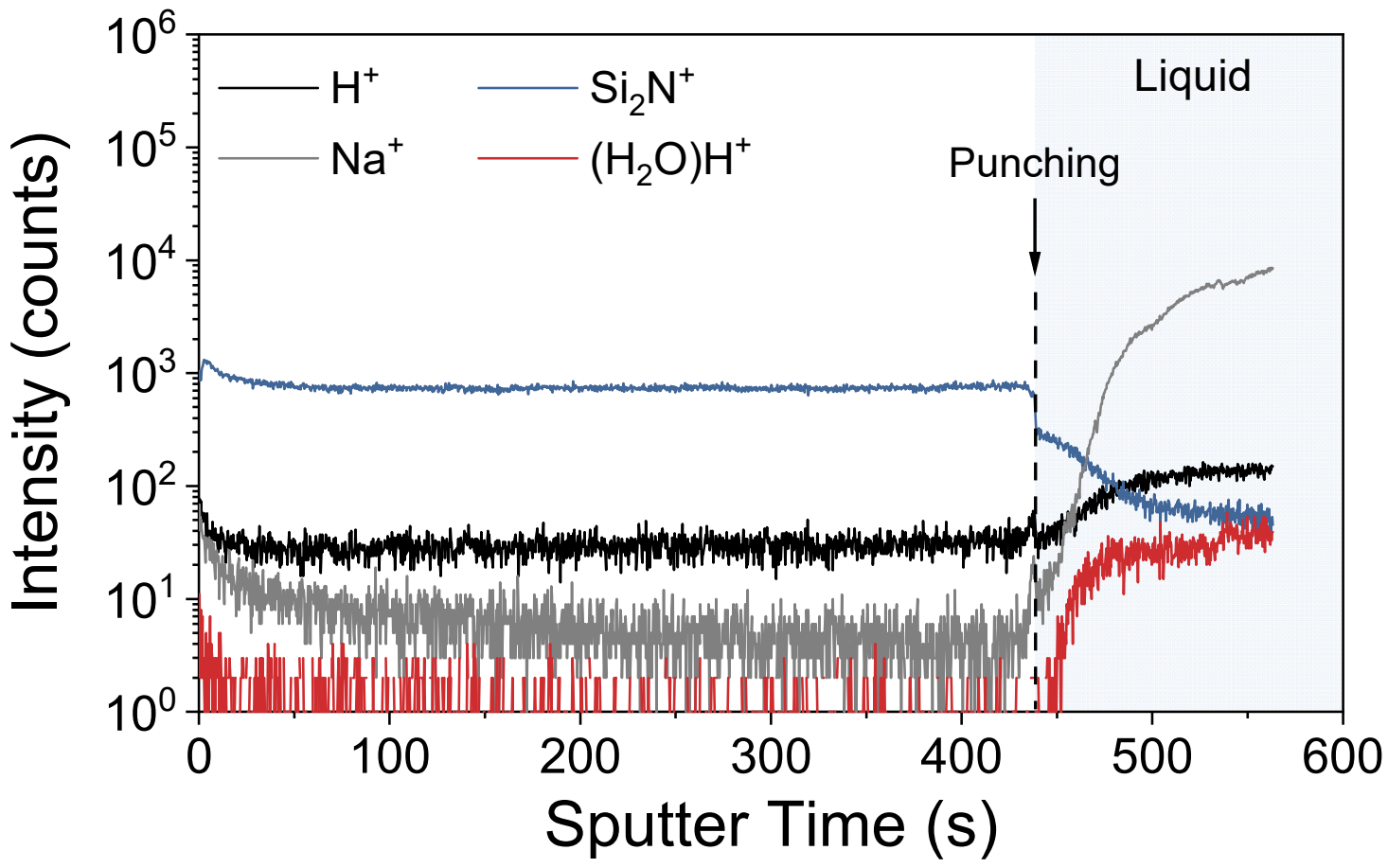

Supplementary Fig. 27. Representative ToF-SIMS depth profiling during determination of hydration distributions. The Si_2N^+ was from the SiN_x film and $(H_2O)H^+$, H^+ and Na^+ were from 1000 ppm Na_2SO_4 solution. The SiN_x film was punched through at around 440 s, allowing Na_2SO_4 solution to permeate through the polyamide membrane. We thus observed that the Si_2N^+ signals were decreased and $(H_2O)H^+$, H^+ and Na^+ signals were increased.

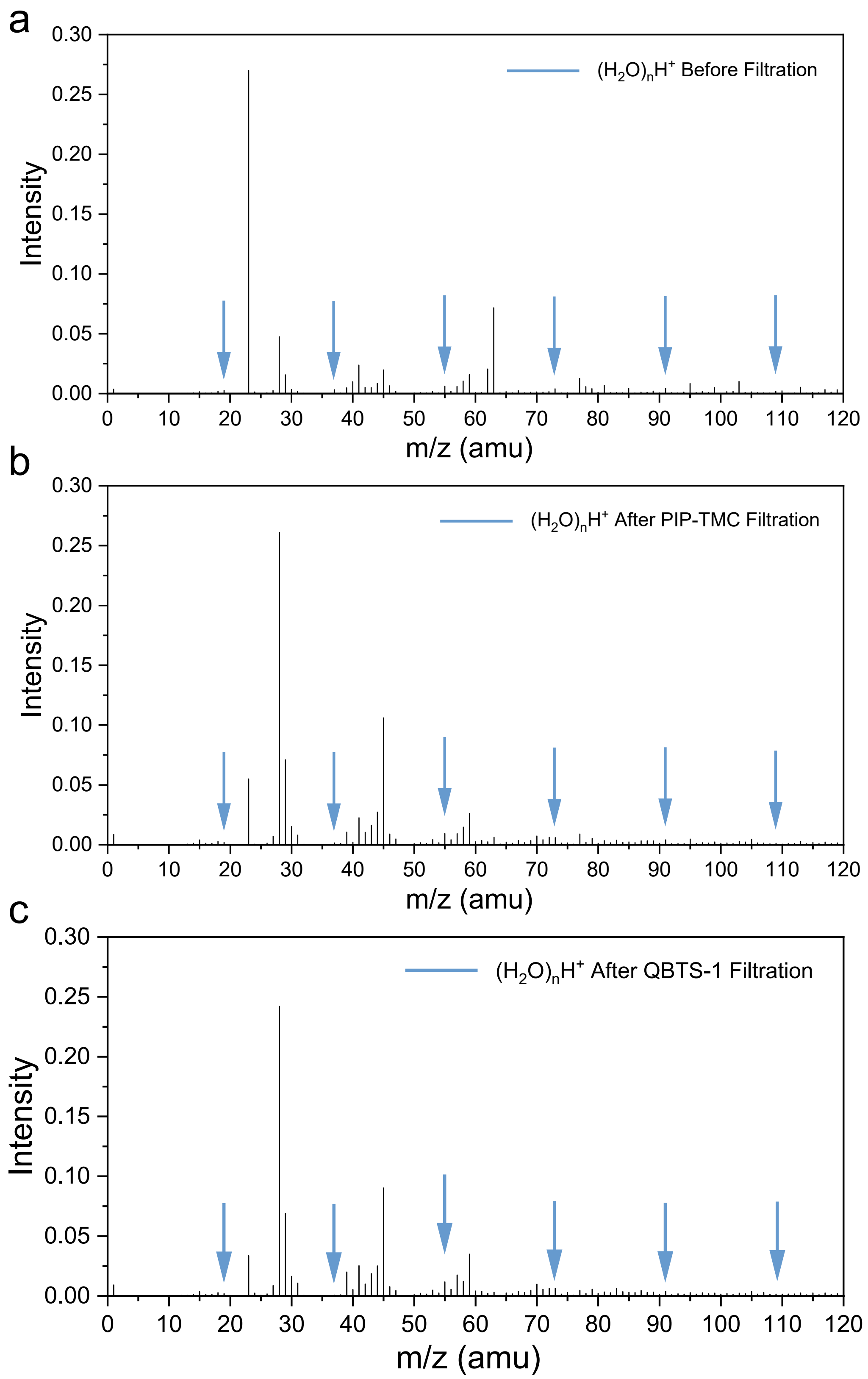

Supplementary Fig. 28. Representative ToF-SIMS spectra of Na_2SO_4 solution before and after filtration by nanofiltration membranes in positive mode. The $(H_2O)_nH^+$ were marked with blue arrows.

Supplementary Table 1. Self-diffusion coefficients of water clusters with different inside H-bonds number.

Type of nanochannel	Number of inside H-bonds	Self-diffusion coefficients (10^{-5} cm^{-2} s^{-1})	Literature
Carbon Nanotubes (CNTs), *r*=0.5 nm	2.22	0.0298	[84]
	2.07	0.0322	
	1.78	1.108	
	1.61	2.475	
	1.37	5.065	
	1.15	5.125	
Carbon Nanotubes (CNTs), *r*=1 nm	2.77	0.956	[84]
	2.5	1.896	
	2.2	3.225	
	2.02	4.462	
	1.69	7.226	
	1.42	10.795	
	1.21	14.52	
Carbon Nanotubes (CNTs), *r*=0.61 nm	3.335	0.162	[85]
	3.281	0.298	
	3.016	0.867	
Carbon Nanotubes (CNTs), *r*=0.815 nm	3.258	2.348	[85]
	3.372	1.393	
	3.435	1.076	
Carbon Nanotubes (CNTs)	1.799	11.3	[86]
	3.279	3.9	
Pure Graphene (2D channel)	2.676	2.107	[87]
	2.589	2.115	
	2.557	2.092	
	2.929	1.366	
	3.244	0.877	
	2.635	1.789	

	2.741	1.678	
BN	2.608	1.929	[87]
(2D channel)	3.054	1.085	
	3.247	0.833	
	2.437	2.649	
	2.47	2.426	
MoS2	2.467	2.507	[87]
(2D channel)	2.942	1.151	
	3.062	0.907	
	1.263	1.499	
Pure Graphene	1.378	2.018	[88]
(2D channel)	1.708	1.02	
	4.424	0.028	
	1.197	1.079	
3% B-doped	1.188	1.059	[88]
Graphene	1.65	0.028	
(2D channel)	4.4	0.007	
	1.016	0.926	
3% Si-doped	1.321	0.862	[88]
Graphene	1.684	0.028	
(2D channel)	4.375	0.012	

Supplementary Table 2. Comparison of permeance and rejection of Na_2SO_4.

	Permeance ($L\ m^{-2}\ h^{-1}\ bar^{-1}$)	Rejection (%)	Literature
	43.0	97.6	Our work
	32.9	99.4	[89]
	19.5	97.5	[90]
	7.52	99.9	[91]
	9.28	96.7	[92]
	6.53	96.5	[93]
	16	97.8	[94]
	5.75	99.1	[95]
	28.6	96.1	[96]
	11.4	98.3	[97]
	10.9	99	[98]
	22.5	95	[99]
	10.4	99.7	[100]
	5.87	97.7	[101]
	7.67	95.75	[102]
Reported	33.4	94.7	[103]
	38.2	92.2	[104]
	28.5	93.9	[104]
	15.9	98.1	[105]
	13.9	94.3	[106]
	28.5	97.1	[107]
	8.8	98.3	[108]
	25.0	94.2	[109]
	17.0	98.5	[110]
	8.2	93.0	[111]
	17.0	98.2	[74]
	18.8	97.9	[112]
	35.2	98.7	[113]
	14.2	97.4	[114]
	7.63	97.2	[115]
	11.98	99.2	[116]
	20.8	95.8	[117]
	13.8	99.4	[118]

Supplementary Table 3. Characteristic parameters of the probability density functions for membranes used herein.

**Membranes**	**Fitted equation**	**Adj.R^2**	**μ_p (nm)**	**σ_p**
PIP-TMC	$f(R_T)=4.86+3.887\ln r_s$	0.9934	0.29	1.29
QBTS-1	$f(R_T)=4.47+3.92\ln r_s$	0.97633	0.32	1.29

Supplementary References

1. Lu, C. *et al. In Situ* Characterization of Dehydration during Ion Transport in Polymeric Nanochannels. *J. Am. Chem. Soc.* **143**, 14242–14252 (2021).
2. Lu, C. *et al.* Dehydration-enhanced ion-pore interactions dominate anion transport and selectivity in nanochannels. *Science Advances* **9**, eadf8412 (2023).
3. Liu, Y.-Y. *et al.* Ion-Specific Effects on Hydrogen Bond Network at a Submicropore Confined Liquid-Vacuum Interface: An in Situ Liquid ToF-SIMS Study. *J. Phys. Chem. Lett.* **10**, 4935–4941 (2019).
4. Zhang, Y. *et al.* In Situ Liquid Secondary Ion Mass Spectrometry: A Surprisingly Soft Ionization Process for Investigation of Halide Ion Hydration. *Anal. Chem.* **91**, 7039–7046 (2019).
5. Liu, Y.-Y., Ying, Y.-L., Hua, X. & Long, Y.-T. In-situ discrimination of the water cluster size distribution in aqueous solution by ToF-SIMS. *Sci. China Chem.* **61**, 159–163 (2018).
6. Lu, C. *et al.* Response of Ionic Hydration Structure and Selective Transport Behavior to Aqueous Solution Chemistry during Nanofiltration. *Environ. Sci. Technol.* **58**, 11791–11801 (2024).
7. Guo, L. *et al.* Formate-Anion-Induced Water Network Reshaping Enables Concurrent Hydrogen and Magnesium Hydroxide Production From Seawater. *Angewandte Chemie International Edition* **n/a**, e6937994.
8. Bockris, J. O. & Saluja, P. P. S. Ionic solvation numbers from compressibilities and ionic vibration potentials measurements. *J. Phys. Chem.* **76**, 2140–2151 (1972).
9. Marcus, Y. The Standard Partial Molar Volumes of Ions in Solution. Part 4. Ionic Volumes in Water at 0−100 °C. *J. Phys. Chem. B* **113**, 10285–10291 (2009).
10. Wang, K. Y. & Chung, T.-S. Fabrication of polybenzimidazole (PBI) nanofiltration hollow fiber membranes for removal of chromate. *Journal of Membrane Science* **281**, 307–315 (2006).
11. Aimar, P., Meireles, M. & Sanchez, V. A contribution to the translation of retention curves into pore size distributions for sieving membranes. *Journal of Membrane Science* **54**, 321–338 (1990).

12. Bowen, W. R. & Mohammad, A. W. Characterization and Prediction of Nanofiltration Membrane Performance—A General Assessment. *Chemical Engineering Research and Design* **76**, 885–893 (1998).

13. Chen, H. *et al.* Free-Volume Depth Profile of Polymeric Membranes Studied by Positron Annihilation Spectroscopy: Layer Structure from Interfacial Polymerization. *Macromolecules* **40**, 7542–7557 (2007).

14. Pavluchkov, V., Shefer, I., Peer-Haim, O., Blotevogel, J. & Epsztein, R. Indications of ion dehydration in diffusion-only and pressure-driven nanofiltration. *Journal of Membrane Science* **648**, 120358 (2022).

15. Shefer, I., Lopez, K., Straub, A. P. & Epsztein, R. Applying Transition-State Theory to Explore Transport and Selectivity in Salt-Rejecting Membranes: A Critical Review. *Environ. Sci. Technol.* **56**, 7467–7483 (2022).

16. Rickman, M., Davis, R. H. & Pellegrino, J. Temperature-variation study of neutral solute and electrolyte fractionation through cellulose acetate and polyamide membranes. *Journal of Membrane Science* **461**, 114–122 (2014).

17. Zwolinski, B. J., Eyring, H. & Reese, C. E. Diffusion and Membrane Permeability. *J. Phys. Chem.* **53**, 1426–1453 (1949).

18. Epsztein, R., DuChanois, R. M., Ritt, C. L., Noy, A. & Elimelech, M. Towards single-species selectivity of membranes with subnanometre pores. *Nat. Nanotechnol.* **15**, 426–436 (2020).

19. Gaussian 16, Revision C.01, M. J. Frisch, G. W. Trucks, H. B. Schlegel, G. E. Scuseria, M. A. Robb, J. R. Cheeseman, G. Scalmani, V. Barone, G. A. Petersson, H. Nakatsuji, X. Li, M. Caricato, A. V. Marenich, J. Bloino, B. G. Janesko, R. Gomperts, B. Mennucci, H. P. Hratchian, J. V. Ortiz, A. F. Izmaylov, J. L. Sonnenberg, D. Williams-Young, F. Ding, F. Lipparini, F. Egidi, J. Goings, B. Peng, A. Petrone, T. Henderson, D. Ranasinghe, V. G. Zakrzewski, J. Gao, N. Rega, G. Zheng, W. Liang, M. Hada, M. Ehara, K. Toyota, R. Fukuda, J. Hasegawa, M. Ishida, T. Nakajima, Y. Honda, O. Kitao, H. Nakai, T. Vreven, K. Throssell, J. A. Montgomery, Jr., J. E. Peralta, F. Ogliaro, M. J. Bearpark, J. J. Heyd, E. N. Brothers, K. N. Kudin, V. N. Staroverov, T. A. Keith, R. Kobayashi, J. Normand, K. Raghavachari, A. P. Rendell,

J. C. Burant, S. S. Iyengar, J. Tomasi, M. Cossi, J. M. Millam, M. Klene, C. Adamo, R. Cammi, J. W. Ochterski, R. L. Martin, K. Morokuma, O. Farkas, J. B. Foresman, and D. J. Fox, Gaussian, Inc., Wallingford CT, 2019.

20. Hariharan, P. C. & Pople, J. A. The influence of polarization functions on molecular orbital hydrogenation energies. *Theoret. Chim. Acta* **28**, 213–222 (1973).

21. Lee, C., Yang, W. & Parr, R. G. Development of the Colle-Salvetti correlation-energy formula into a functional of the electron density. *Phys. Rev. B* **37**, 785–789 (1988).

22. Vosko, S. H., Wilk, L. & Nusair, M. Accurate spin-dependent electron liquid correlation energies for local spin density calculations: a critical analysis. *Can. J. Phys.* **58**, 1200–1211 (1980).

23. Marenich, A. V., Cramer, C. J. & Truhlar, D. G. Universal Solvation Model Based on Solute Electron Density and on a Continuum Model of the Solvent Defined by the Bulk Dielectric Constant and Atomic Surface Tensions. *J. Phys. Chem. B* **113**, 6378–6396 (2009).

24. Wu, H., Xu, Y., Tang, Q., Wu, B. & Wu, P. Microstructure engineering of polyamide membranes for ultrafast polar and non-polar solvent transport. *Nat Commun* **16**, 8414 (2025).

25. Liao, S. *et al.* Theoretical framework for confined ion transport in two-dimensional nanochannels. *Nat Commun* **16**, 6675 (2025).

26. Pan, Z. *et al.* Molecular manipulation of polyamide nanostructures reconciles the permeance-selectivity threshold for precise ion separation. *Nat Commun* **16**, 7171 (2025).

27. Han, S. *et al.* Microporous organic nanotube assisted design of high performance nanofiltration membranes. *Nat Commun* **13**, 7954 (2022).

28. Lin, J. *et al.* Shielding effect enables fast ion transfer through nanoporous membrane for highly energy-efficient electrodialysis. *Nat Water* **1**, 725–735 (2023).

29. Jia, Y. *et al.* Voltage-controlled organic solvent nanofiltration using conjugated microporous polymer membranes. *Nat Commun* **17**, 236 (2025).

30. Ouyang, J., Sheng, Y. & Wang, W. Water Transport Dynamics in Layered

Nanoporous Graphene: An In-Depth Molecular Dynamics Study. *J. Phys. Chem. B* **129**, 8521–8540 (2025).

31. Fang, C., Yu, Z. & Qiao, R. Impact of Surface Ionization on Water Transport and Salt Leakage through Graphene Oxide Membranes. *J. Phys. Chem. C* **121**, 13412–13420 (2017).
32. Chen, B., Jiang, H., Liu, X. & Hu, X. Observation and Analysis of Water Transport through Graphene Oxide Interlamination. *J. Phys. Chem. C* **121**, 1321–1328 (2017).
33. Martínez, L., Andrade, R., Birgin, E. G. & Martínez, J. M. PACKMOL: A package for building initial configurations for molecular dynamics simulations. *Journal of Computational Chemistry* **30**, 2157–2164 (2009).
34. Abraham, M. J. *et al.* GROMACS: High performance molecular simulations through multi-level parallelism from laptops to supercomputers. *SoftwareX* **1–2**, 19–25 (2015).
35. Szilárd, P., Abraham, M. J., Kutzner, C., Hess, B. & Lindahl, E. Tackling Exascale Software Challenges in Molecular Dynamics Simulations with GROMACS. in vol. 8759 3–27 (2015).
36. Pronk, S. *et al.* GROMACS 4.5: a high-throughput and highly parallel open source molecular simulation toolkit. *Bioinformatics* **29**, 845–854 (2013).
37. Hess, B., Kutzner, C., van der Spoel, D. & Lindahl, E. GROMACS 4: Algorithms for Highly Efficient, Load-Balanced, and Scalable Molecular Simulation. *J. Chem. Theory Comput.* **4**, 435–447 (2008).
38. Jorgensen, W. L., Maxwell, D. S. & Tirado-Rives, J. Development and Testing of the OPLS All-Atom Force Field on Conformational Energetics and Properties of Organic Liquids. *J. Am. Chem. Soc.* **118**, 11225–11236 (1996).
39. Hess, B., Bekker, H., Berendsen, H. J. C. & Fraaije, J. G. E. M. LINCS: A linear constraint solver for molecular simulations. *Journal of Computational Chemistry* **18**, 1463–1472 (1997).
40. Bussi, G., Donadio, D. & Parrinello, M. Canonical sampling through velocity rescaling. *J. Chem. Phys.* **126**, 014101 (2007).
41. Essmann, U. *et al.* A smooth particle mesh Ewald method. *J. Chem. Phys.* **103**,

8577–8593 (1995).

42. Humphrey, W., Dalke, A. & Schulten, K. VMD: Visual molecular dynamics. *Journal of Molecular Graphics* **14**, 33–38 (1996).

43. Ishiyama, T. & Kitanaka, K. Asymmetric Hydrogen-Bonding Structure at a Water/Ice Interface. *J. Phys. Chem. C* **124**, 23287–23294 (2020).

44. Otsuki, Y. *et al.* Unveiling subsurface hydrogen-bond structure of hexagonal water ice. *Phys. Rev. B* **96**, 115405 (2017).

45. de Dios, A. C. 17O Chemical Shifts in Water. *J. Phys. Chem. A* **129**, 11203–11212 (2025).

46. Ozkanlar, A., Zhou, T. & Clark, A. E. Towards a unified description of the hydrogen bond network of liquid water: A dynamics based approach. *J. Chem. Phys.* **141**, (2014).

47. Robalo, J. R. *et al.* Hydrophobic but Water-Friendly: Favorable Water–Perfluoromethyl Interactions Promote Hydration Shell Defects. *J. Am. Chem. Soc.* **141**, 15856–15868 (2019).

48. Itoh, Y. *et al.* Ultrafast water permeation through nanochannels with a densely fluorous interior surface. (2022).

49. Perera, P. N. *et al.* Observation of water dangling OH bonds around dissolved nonpolar groups. *Proceedings of the National Academy of Sciences* **106**, 12230–12234 (2009).

50. Roy, S., Biswas, B., Ghosh, N., Singh, P. C. & Mondal, J. A. Hydrophobic Hydration of Fluoroalkyl (C–F) is Distinctly Different from That of Its Hydrogenated Counterpart (C–H), as Observed by Raman Difference with Simultaneous Curve Fitting Analysis. *J. Phys. Chem. C* **123**, 27012–27019 (2019).

51. Wang, P. *et al.* Ultrafast ion sieving using nanoporous polymeric membranes. *Nat Commun* **9**, 569 (2018).

52. Cruz-Chu, E. R., Ritz, T., Siwy, Z. S. & Schulten, K. Molecular control of ionic conduction in polymer nanopores. *Faraday Discuss.* **143**, 47–62 (2009).

53. Kumar, S., Rosenberg, J. M., Bouzida, D., Swendsen, R. H. & Kollman, P. A. THE weighted histogram analysis method for free-energy calculations on biomolecules. I.

The method. *Journal of Computational Chemistry* **13**, 1011–1021 (1992).

54. Woolf, T. B. & Roux, B. Molecular dynamics simulation of the gramicidin channel in a phospholipid bilayer. *Proceedings of the National Academy of Sciences* **91**, 11631–11635 (1994).
55. Hummer, G. Position-dependent diffusion coefficients and free energies from Bayesian analysis of equilibrium and replica molecular dynamics simulations. *New J. Phys.* **7**, 34 (2005).
56. Marrink, S.-J. & Berendsen, H. J. C. Simulation of water transport through a lipid membrane. *J. Phys. Chem.* **98**, 4155–4168 (1994).
57. Plimpton, S. Fast Parallel Algorithms for Short-Range Molecular Dynamics. *Journal of Computational Physics* **117**, 1–19 (1995).
58. Heinz, H., Lin, T.-J., Kishore Mishra, R. & Emami, F. S. Thermodynamically Consistent Force Fields for the Assembly of Inorganic, Organic, and Biological Nanostructures: The INTERFACE Force Field. *Langmuir* **29**, 1754–1765 (2013).
59. Li, X., Zhang, G., Liu, H., Lan, H. & Qu, J. Sequential Demulsification through the Hydrophobic-Hydrophilic-Hydrophobic Filtration Layer toward High-Performing Oil Recovery. *Environ. Sci. Technol.* **57**, 12083–12093 (2023).
60. Yan, X., Meng, Q., Ammami, M.-T. & Wei, L. Effect of PAM on Surface Hydrophobicity of Montmorillonite and Difference of Interface Adsorption: An Experimental and Simulation Study. *ACS Omega* **9**, 15818–15832 (2024).
61. Hossen, M. J., Nematipour, A. & Bilodeau, C. Molecular Scale Hydrophobicity and Adsorption Thermodynamics on Hydrophobic-Charged Surfaces. *ACS Nano* **20**, 6970–6982 (2026).
62. Vassetti, D., Pagliai, M. & Procacci, P. Assessment of GAFF2 and OPLS-AA General Force Fields in Combination with the Water Models TIP3P, SPCE, and OPC3 for the Solvation Free Energy of Druglike Organic Molecules. *J. Chem. Theory Comput.* **15**, 1983–1995 (2019).
63. Lim, V. T., Bayly, C. I., Fusti-Molnar, L. & Mobley, D. L. Assessing the Conformational Equilibrium of Carboxylic Acid via Quantum Mechanical and Molecular Dynamics Studies on Acetic Acid. *J. Chem. Inf. Model.* **59**, 1957–1964

(2019).

64. Cohen-Tanugi, D. & Grossman, J. C. Water Desalination across Nanoporous Graphene. *Nano Lett.* **12**, 3602–3608 (2012).

65. Peng, H. *et al.* Phosphonium Modification Leads to Ultrapermeable Antibacterial Polyamide Composite Membranes with Unreduced Thickness. *Adv. Mater.* **32**, 2001383 (2020).

66. Han, R. *et al.* Piperazine-modulated bis-tris-based polyester-amide nanofilm for high-performance nanofiltration membrane. *Advanced Membranes* **8**, 100220 (2026).

67. Li, N. *et al.* Tuning composite nanofiltration membranes with γ-cyclodextrin for improved Mg^{2+}/Li^{+} selectivity. *Separation and Purification Technology* **330**, 125206 (2024).

68. Li, J., Peng, H., Liu, K. & Zhao, Q. Polyester Nanofiltration Membranes for Efficient Cations Separation. *Advanced Materials* **36**, 2309406 (2024).

69. Cheng, J. *et al.* A novel polyester composite nanofiltration membrane prepared by interfacial polymerization catalysed by 4-dimethylaminopyridine: Enhanced the water permeability and anti-fouling ability. *Polymer* **153**, 24–32 (2018).

70. Zhang, R. *et al.* A novel polyesteramide thin film composite nanofiltration membrane prepared by interfacial polymerization of serinol and trimesoyl chloride (TMC) catalyzed by 4-dimethylaminopyridine (DMAP). *Journal of Membrane Science* **542**, 68–80 (2017).

71. Baig, U. & Waheed, A. Exploiting interfacial polymerization to fabricate hyper-cross-linked nanofiltration membrane with a constituent linear aliphatic amine for freshwater production. *npj Clean Water* **5**, 46 (2022).

72. Wanjiya, M., Jin, C.-G., Shen, Y., Yin, M.-J. & An, Q.-F. Conversion of membrane surface charge property via post-crosslinking for heavy metal ions removal. *Separation and Purification Technology* **362**, 131613 (2025).

73. Yu, D. *et al.* Polyamide composite nanofiltration membrane via a combination of polydopamine grafting and reverse interfacial polymerization for high Mg^{2+}/Li^{+} separation capacity. *Separation and Purification Technology* **355**, 129746 (2025).

74. Yan, F. *et al.* Improving the water permeability and antifouling property of thin-

film composite polyamide nanofiltration membrane by modifying the active layer with triethanolamine. *Journal of Membrane Science* **513**, 108–116 (2016).

75. Soyekwo, F., Wen, H., Liao, D. & Liu, C. Fouling-resistant ionic graft-polyamide nanofiltration membrane with improved permeance for lithium separation from MgCl2/LiCl mixtures. *Journal of Membrane Science* **659**, 120773 (2022).
76. Li, X. *et al.* Surface modification of polyamide nanofiltration membrane by grafting zwitterionic polymers to improve the antifouling property. *Journal of Applied Polymer Science* **131**, (2014).
77. Li, Y. *et al.* Surface fluorination of polyamide nanofiltration membrane for enhanced antifouling property. *Journal of Membrane Science* **455**, 15–23 (2014).
78. Ren, X. *et al.* Grafting modification of thin-film composite membrane with quaternary ammonium polyelectrolyte for Mg^{2+}/Li^{+} separation. *Journal of Environmental Chemical Engineering* **12**, 112223 (2024).
79. Li, H., Shi, W., Su, Y., Zhang, H. & Qin, X. Preparation and characterization of carboxylated multiwalled carbon nanotube/polyamide composite nanofiltration membranes with improved performance. *Journal of Applied Polymer Science* **134**, 45268 (2017).
80. Tan, Z., Chen, S., Peng, X., Zhang, L. & Gao, C. Polyamide membranes with nanoscale Turing structures for water purification. *Science* **360**, 518–521 (2018).
81. Li, Q. *et al.* High performance Li^{+}/Mg^{2+} separation membrane by grafted short chain amino-rich monomers. *Journal of Membrane Science* **677**, 121634 (2023).
82. Chen, G., Li, X., Li, X., Song, Z. & Liu, B. Active-passive synergistic alleviation of membrane scaling from groundwater by grafting charged positive monomers on the surface of nanofiltration membranes. *Desalination* **599**, 118436 (2025).
83. Ritt, C. L. *et al.* Ionization behavior of nanoporous polyamide membranes. *Proc. Natl. Acad. Sci. U.S.A.* **117**, 30191–30200 (2020).
84. Srivastava, A., Hassan, J. & Homouz, D. Hydrogen Bond Dynamics and Phase Transitions of Water inside Carbon Nanotubes. *Nanomaterials* **13**, 284 (2023).
85. Mendonça, B. H. S., Ternes, P., Salcedo, E., de Oliveira, A. B. & Barbosa, M. C. Water diffusion in rough carbon nanotubes. *The Journal of Chemical Physics* **152**,

024708 (2020).
86. Tunuguntla, R. H. *et al.* Enhanced water permeability and tunable ion selectivity in subnanometer carbon nanotube porins. *Science* **357**, 792–796 (2017).
87. Zhang, Q., Wang, X., Li, J., Lu, S. & Lu, D. How pressure affects confine water inside different nanoslits. *RSC Adv.* **9**, 19086–19094 (2019).
88. Abbaspour, M., Akbarzadeh, H. & Zaeifi, S. Thermodynamics, Structure, and Dynamic Properties of Nanostructured Water Confined into B-, N-, and Si-Doped Graphene Surfaces and Carbon Nanotubes. *Ind. Eng. Chem. Res.* **59**, 9642–9654 (2020).
89. Zhang, X. *et al.* In Situ Chemical Modification with Zwitterionic Copolymers of Nanofiltration Membranes: Cure for the Trade-Off between Filtration and Antifouling Performance. *ACS Appl. Mater. Interfaces* **14**, 28842–28853 (2022).
90. Zhan, Z.-M. *et al.* Coupling heat curing and surface modification for the fabrication of high permselectivity polyamide nanofiltration membranes. *Journal of Membrane Science* **623**, 119073 (2021).
91. Zhu, X. *et al.* Supramolecular-Based Regenerable Coating Layer of a Thin-Film Composite Nanofiltration Membrane for Simultaneously Enhanced Desalination and Antifouling Properties. *ACS Appl. Mater. Interfaces* **11**, 21137–21149 (2019).
92. Li, A. *et al.* Polyhydroxy Group Functionalized Zwitterion for a Polyamide Nanofiltration Membrane with High Water Permeation and Antifouling Performance. *ACS Appl. Polym. Mater.* **2**, 3850–3858 (2020).
93. Deng, L. *et al.* Fabrication of antifouling thin-film composite nanofiltration membrane via surface grafting of polyethyleneimine followed by zwitterionic modification. *Journal of Membrane Science* **619**, 118564 (2021).
94. Peng, H., Tang, Q., Tang, S., Gong, J. & Zhao, Q. Surface modified polyamide nanofiltration membranes with high permeability and stability. *Journal of Membrane Science* **592**, 117386 (2019).
95. Zhu, X. *et al.* Improving chlorine resistance and separation performance of thin-film composite nanofiltration membranes with in-situ grafted melamine. *Desalination* **489**, 114539 (2020).

96. Pu, L. *et al.* Tailored nanofiltration membranes with enhanced permeability and antifouling performance towards leachate treatment. *Journal of Membrane Science* **658**, 120730 (2022).

97. Zhang, H.-L., Gao, Y.-B. & Gai, J.-G. Guanidinium-functionalized nanofiltration membranes integrating anti-fouling and antimicrobial effects. *J. Mater. Chem. A* **6**, 6442–6454 (2018).

98. Zhou, B., Huang, F., Gao, C. & Xue, L. The role of ring opening reaction chemistry of sultones/lactones in the direct zwitterionization of polyamide nano-filtration membranes. *Journal of Membrane Science* **641**, 119918 (2022).

99. He, B., Peng, H., Chen, Y. & Zhao, Q. High performance polyamide nanofiltration membranes enabled by surface modification of imidazolium ionic liquid. *Journal of Membrane Science* **608**, 118202 (2020).

100. Chen, Y., Wu, G., Xue, H., Ren, Y. & Duan, X. Engineering nanofiltration membranes based on a two-step modification process with enhanced antifouling and salt separation performance. *Separation and Purification Technology* **330**, 125546 (2024).

101. Vaishnavi, P. S. V. *et al.* Surface modification of thin film composite nanofiltration membrane with graphene oxide by varying amine linkers: Synthesis, characterization, and applications. *Journal of Membrane Science* **687**, 122021 (2023).

102. Ang, M. B. M. Y. *et al.* Modifying the surface of active polyamide layer in thin-film composite tubular membranes with polyethylene glycol for improved separation and antifouling. *Journal of the Taiwan Institute of Chemical Engineers* **131**, 104157 (2022).

103. Guo, X. *et al.* High flux nanofiltration membrane via surface modification using spirocyclic quaternary ammonium diamine for efficient antibiotics/salt separation. *Separation and Purification Technology* **325**, 124736 (2023).

104. Guo, X. *et al.* Tailored nanofiltration membrane for enhanced antibiotic desalination by surface modification using branched quaternary triethanolamine assembly. *Journal of Membrane Science* **713**, 123364 (2025).

105. Zhan, Z.-M. *et al.* Polyamide Nanofiltration Membranes with Enhanced Desalination and Antifouling Performance Enabled by Surface Grafting Polyquaternium-7. *Ind. Eng. Chem. Res.* **60**, 14297–14306 (2021).

106. Jashni, E. *et al.* Polyamide thin film nanocomposite membranes modified with cationic nanogel for efficient Li^+/Mg^{2+} separation. *Desalination* **616**, 119336 (2025).

107. Fan, K. *et al.* Esterified Chlorine-Resistant Nanofiltration Membranes with Enhanced Removal of Disinfection Byproducts for Efficient Water Purification. *Environ. Sci. Technol.* **59**, 2875–2885 (2025).

108. Xu, P. *et al.* Charge-Sign-Independent Separation of Mono- and Divalent Ions With Nanofiltration Membranes. *Advanced Functional Materials* **35**, 2416458 (2025).

109. Zhang, J. *et al.* Surface grafted zwitterions modulate the microstructure of polyamide separation layer to achieve efficient antibiotic desalination. *Journal of Water Process Engineering* **63**, 105495 (2024).

110. Liu, M. *et al.* High efficient removal of dyes from aqueous solution through nanofiltration using diethanolamine-modified polyamide thin-film composite membrane. *Separation and Purification Technology* **173**, 135–143 (2017).

111. Cao, X.-L. *et al.* High-permeability and anti-fouling nanofiltration membranes decorated by asymmetric organic phosphate. *Journal of Membrane Science* **617**, 118667 (2021).

112. Huang, B.-Q. *et al.* Dually charged polyamide nanofiltration membranes fabricated by microwave-assisted grafting for heavy metals removal. *Journal of Membrane Science* **640**, 119834 (2021).

113. Ren, L. *et al.* Cucurbit[n]uril-rotaxanes functionalized membranes with heterogeneous channel and regenerable surface for efficient and sustainable nanofiltration. *Journal of Membrane Science* **659**, 120765 (2022).

114. Song, Q. *et al.* Ultrathin surface-amination-modified membrane with ion-charge shielding effect for ultraselective nanofiltration. *Desalination* **597**, 118325 (2025).

115. Li, Y., Li, J., Zhu, D., Qian, G. & Tang, H. Facile dual-functionalization of NF membranes with excellent chlorine resistance and good antifouling property by in-

situ grafting of zwitterions. *Separation and Purification Technology* **315**, 123660 (2023).

116. Liu, Y. *et al.* A nanofiltration membrane with positively and negatively charged groups by grafted p-aminosalicylic acid-Fe(III) chelation for Li^{+}/Mg^{2+} efficient separation. *Separation and Purification Technology* **308**, 122968 (2023).

117. Guo, X. *et al.* Surface modification of nanofiltration membrane using quaternary diethanolamine for efficient antibiotics/salt separation. *Journal of Membrane Science* **701**, 122740 (2024).

118. Hu, H., Qi, Z., Chen, C., Zhou, Z. & Shen, L. Mechanistic study on distinct roles of antibiotics in constructing high-performance antifouling nanofiltration membranes *via in-situ* and surface modifications. *Journal of Membrane Science* **734**, 124491 (2025).
