# Supplementary material for: Dissociating water clusters via polyhydroxy quaternized interface for enhanced water permeation in nanochannels
Source: Nat Commun. 2026 Aug 5;17:9435. doi: 10.1038/s41467-026-76291-z (PMC13538467; doi:10.1038/s41467-026-76291-z)
Supplement: Supplementary file 2 [file 41467_2026_76291_MOESM2_ESM.pdf]

nature portfolio

Peer Review File

Dissociating Water Clusters via Polyhydroxy Quaternized Interface for Enhanced Water Permeation in Nanochannels

Corresponding Author: Professor Chengzhi Hu

This file contains all reviewer reports in order by version, followed by all author rebuttals in order by version.

Attachments originally included by the reviewers as part of their assessment can be found at the end of this file.

Version 0:

Reviewer comments:

Reviewer #1

(Remarks to the Author)
This paper presents a high-flux nanofiltration membrane based on quaternary ammonium salt surface functionalization. The proposed mechanism, which involves the dissociation of water clusters through hydration competition between functional groups and water molecules to further enhance water permeability, is important. The authors employed in situ liquid time-of-flight secondary ion mass spectrometry (ToF-SIMS) and molecular dynamics simulations to provide experimental evidence and molecular-level analysis of water cluster dissociation. Overall, this is an innovative study, and the proposed mechanism offers valuable insights for various water-related interfacial processes. I believe this work is suitable for publication in Nature Communications. Some specific comments include:
1. Quaternary ammonium salt modification is widely used for antifouling treatment in nanofiltration membranes. Do the membranes prepared in this study possess antifouling or antibacterial properties?
2. Furthermore, the authors should demonstrate the stability of the fabricated nanofiltration membranes, for example, by testing membrane performance under different pressures, salt concentrations, and filtration durations.
3. Could the authors further investigate the influence of the functional groups on hydrogen bond lifetime to provide additional evidence supporting their role in water cluster dissociation?
4. The authors conducted extensive simulation analyses to explain the molecular mechanism of water cluster dissociation. Please further elaborate on the rationale for selecting different models (e.g., graphene pores, polyamide models).
5. The authors observed a significant decrease in the rejection of $MgCl_2$ by the QBTS-1 membrane and attributed this to the promotion of ion dehydration by -OH and $-R_3N^+$. Is this claim supported by evidence? This point needs further discussion and insight.
6. In practice, the functional groups discussed by the authors are quite common in membrane separation processes. Is the observed phenomenon universal? Does this effect also exist in other high-flux nanofiltration membranes?
7. According to the mechanism proposed in this paper, water clusters dissociate when they approach the functional groups. However, given that the number of these functional group sites is finite, could the "active sites" become occupied, leading to the eventual failure of this effect?
8. In this study, the modified nanofiltration membrane exhibits improved hydrophilicity and zeta potential, both of which are known to contribute to enhanced water flux. How do the authors attribute the significant increase in flux primarily to the water cluster dissociation mechanism, rather than to these conventional and well-known effects of hydrophilicity and electrical properties?
9. In the ToF-SIMS tests, the authors used a 30 keV Bi_3^+ beam to bombard the solution. Could this energy level cause intrinsic dissociation of the water clusters, thereby affecting the accuracy of the method? Additionally, the authors should provide more details regarding the ToF-SIMS testing procedure to explain how the hydration distributions were accurately obtained.
10. Furthermore, I noted that during the ToF-SIMS measurements, the opposite side of the membrane was under vacuum. Could this condition potentially introduce discrepancies between the tested results and the actual operating system?

Reviewer #2

(Remarks to the Author)
Focusing on the core demand of improving the water permeability of nanofiltration (NF) membranes, this study proposed an

innovative strategy based on polyhydroxy quaternized interfaces to dissociate water clusters. By combining experimental methods with molecular simulations, it reveals the mechanism by which the hydration competition of functional groups regulates the structure of water clusters and thereby optimizes water transport. The developed QBTS-functionalized NF membrane exhibits outstanding performance in balancing water flux and salt rejection. There are aspects requiring improvement in terms of detailed expression, mechanism integrity, and consistency between experiments and simulations, which need further revisions to enhance the persuasiveness of the study.

1. The abscissa of Figure 1b is labeled "Number of H-Bonds for water clusters", but it is not clear whether it refers to "the number of hydrogen bonds formed by a single water molecule in a water cluster" or "the total number of hydrogen bonds in a single water cluster".
2. The functional group -R_3N^+ is repeatedly mentioned in Figure 2 and the main text, but the specific structure of R is not clearly defined.
3. In lines 163-164, the "number of dangling bonds (DBs)" is mentioned , but the specific meaning of "dangling bonds" (as well as the statistical method) is not explained.
4. QBTS-1, QBTS-3, and QBTS-5 are not clearly defined when first mentioned or in the experimental methods section. It is suggested to add the explanation that "QBTS-1, QBTS-3, and QBTS-5 respectively refer to NF membranes modified with 1 wt%, 3 wt%, and 5 wt% QBTS aqueous solutions" either in the "Nanofiltration Membrane Preparation" chapter or when QBTS-1 is first mentioned.
5. In lines 118-215, an inert graphene pore model with functional groups (-H, -OH, -COO^-, -R_3N^+) modified on the pore edges is used to explore the mechanism. However, the functional groups on the active layer of the modified membrane in subsequent experiments are polyhydroxy quaternary ammonium structures (-$N(OH)_n$), resulting in differences in the type and distribution of functional groups between the two systems. It is suggested to supplement the DFT calculation of the interaction energy between -NOH groups and water molecules, following the method used for Figure 1c.
6. In Lines 118-215, the author modifies the functional groups inside the pores of perforated graphene, while in subsequent experimental studies, the modification is conducted on the membrane surface, leading to inconsistency between the two approaches.
7. During the simulation, the force field should be consistent.
8. In Lines 305-306, the conclusion that "the water flux of the membrane increases with the increase of hydroxyl content" is open to question.
9. Figure 4c fails to demonstrate the competitive process between "the hydrogen bonding interaction between -OH/-R_3N^+ and water molecules" . Consequently, it cannot well explain why the performance of BTS is inferior to that of QTEOA.
10. Figure 5b, the water clusters in the bulk solution are mainly composed of 3-5 water molecules. After filtration through both PIP-TMC and QBTS-1 membranes, the water clusters are mainly composed of 3 water molecules, indicating that the pore structure itself can reduce the number of water molecules in the clusters. However, there is a difference in salt rejection between the two membranes. Is the reason merely the reduction in the number of water molecules in the clusters caused by hydration competition? Has the influence of pore size been considered?
11. In Lines 338-341 , it is proposed that "the poorest $MgCl_2$ rejection performance of QBTS-1 is due to hydrogen bond destruction", but this cannot explain "why the $MgSO_4$ rejection performance remains good".
12. In the experiments, solutions containing Na_2SO_4, $MgCl_2$, etc., are used as research objects. The hydration of ions will form "hydration competition" with the functional groups on the membrane surface (e.g., the coordination between ions and water molecules may weaken the dissociation effect of functional groups on water clusters). However, the existing simulations do not include the ion system.
13. It is suggested to analyze the ion rejection behavior through molecular dynamics simulations and add simulation details, such as the statistical range inside or at the entrance of the pores.

Reviewer #3

(Remarks to the Author)

This manuscript presents interesting experimental and computational results on rapid water transport in functionalized NF membranes. The method is scalable, and the combination of experiments, performance tests, and simulation provides useful mechanistic insight and context. I recommend the article be published after several key revisions. I have provided specific comments below that the authors should address:

• Lines 137-155, Figure 1: The data seem to suggest that water prefers to interact with -R3N+, -OH, and -COO- over itself. I am skeptical of that so this point should be discussed more. If this is indeed the claim, then the authors should comment on and cite what has been observed in prior studies. There must be prior computational studies on this topic.
• Lines 220-223: The authors should provide citations for the interfacial polymerization used to fabricate membranes. For their QBTS NF membranes, the authors should clearly explain how the QBTS is bound to the polymer network based on their methodology, as it is unclear based on current description.
• Figure 4 (a, b, e): Should the Y-axis here actually be water permeance? It is labeled as water flux currently but has units of permeance.
• Figure 4 c and Lines 330-343: It should be made clear here (and perhaps elsewhere), that this is based on a proposed mechanism. The performance data is quite interesting, though the connection to mechanisms is somewhat speculative. It is reasonable to suggest these statements, though it should be clear these are proposed ideas based on observations.
• Lines 346-366 and Figure 5 b: The section was very interesting but challenging to parse through. For example, it took me multiple reads to understand “after filtration” was a solution phase measurement. Is the argument that the reader is meant to walk away with that if the hydration distribution shifts, it means that a particular structure of water is more likely to permeate through the membrane? More clarity and few additional sentences in the discussion would be helpful.
• Lines 382-383: The authors should mention the temperatures probed here and reference the SI.
• Lines 386-389: I do not necessarily agree that stronger interactions relate to lower diffusion energy barriers. Stronger

interactions have been shown to increase the energy barrier in many simulation studies. Can the authors provide direct evidence that suggests that stronger interactions reduce the energy barrier? Moreover, the authors should cite a broader set of literature in these discussions.

Version 1:

Reviewer comments:

Reviewer #1

(Remarks to the Author)
The authors have addressed all my comments in the previous review. The manuscript is good for publication.

Reviewer #2

(Remarks to the Author)
The authors have addressed most of the comments. Here are some minor suggestions:
For the molecular dynamics simulations, the choice of force field parameters exerts a significant influence on the simulation results and may even lead to completely opposite conclusions. In this work, two different force fields, OPLS AA and GAFF2, were separately employed for simulations involving the graphene system, which is insufficiently rigorous to ensure the consistency, comparability, and reliability of the simulation methodology. For the same type of system (e.g., the functionalized graphene pore system in this study), it is recommended to use a unified force field to avoid uncontrollable errors arising from force field discrepancies. Although the authors argue that the force field mainly affects quantitative details rather than the overall physical trends and theoretical conclusions, this viewpoint still requires further validation. Therefore, I suggest that the authors repeat the validation of key conclusions using at least one additional independent force field to confirm the robustness of the findings. Alternatively, the rationality and accuracy of the selected force fields should be demonstrated by quantitative/qualitative consistency between simulation results and experimental data.

Reviewer #3

(Remarks to the Author)

Version 2:

Reviewer comments:

Reviewer #1

(Remarks to the Author)
The authors have addressed all my comments. The manuscript is fine for publication.

Reviewer #2

(Remarks to the Author)
All the comments were well addressed by the authors, therefore I recommend the acceptance of this manuscript.

Reviewer #3

(Remarks to the Author)
The authors have satisfactorily revised the manuscript based on my comments from the original submission.

Open Access This Peer Review File is licensed under a Creative Commons Attribution 4.0 International License, which permits use, sharing, adaptation, distribution and reproduction in any medium or format, as long as you give appropriate credit to the original author(s) and the source, provide a link to the Creative Commons license, and indicate if changes were made.
In cases where reviewers are anonymous, credit should be given to 'Anonymous Referee' and the source.
The images or other third party material in this Peer Review File are included in the article's Creative Commons license, unless indicated otherwise in a credit line to the material. If material is not included in the article's Creative Commons license and your intended use is not permitted by statutory regulation or exceeds the permitted use, you will need to obtain permission directly from the copyright holder.
To view a copy of this license, visit https://creativecommons.org/licenses/by/4.0/

RESPONSE TO REVIEWERS' COMMENTS

Reviewer #1:

<u>Reviewer</u>: *This paper presents a high-flux nanofiltration membrane based on quaternary ammonium salt surface functionalization. The proposed mechanism, which involves the dissociation of water clusters through hydration competition between functional groups and water molecules to further enhance water permeance, is important. The authors employed in situ liquid time-of-flight secondary ion mass spectrometry (ToF-SIMS) and molecular dynamics simulations to provide experimental evidence and molecular-level analysis of water cluster dissociation. Overall, this is an innovative study, and the proposed mechanism offers valuable insights for various water-related interfacial processes. I believe this work is suitable for publication in Nature Communications. Some specific comments include:*

Our Response: We thank the reviewer for their thorough analysis of our work and greatly appreciate the positive feedback. We would like to kindly remind that in the point-by-point response, we have marked newly added text in blue, deleted text in red with a strikethrough, and placed all revision marks within grey text boxes. In the text, MS-Line refers to the line numbers in the Marked Manuscript, and SI-Line refers to the line numbers in the Marked Supplementary Information.

<u>Reviewer (Comment 1)</u>: *Quaternary ammonium salt modification is widely used for antifouling treatment in nanofiltration membranes. Do the membranes prepared in this study possess antifouling or antibacterial properties?*

Our Response: We thank the reviewer for the comment. Indeed, the more hydrophilic surface, the more negatively charged membrane surface, and the quaternary-N^+ of QBTS-1 suggest certain antifouling properties and antibacterial characteristics.

First, we evaluated the antifouling performance of QBTS in comparison with the widely used NF 270 commercial NF membrane using dead-end filtration (experimental temperature of 25 °C, constant operating pressure of 12 bar), with bovine serum albumin (BSA) and sodium alginate (SA) as two model foulants. As shown in Fig. S16, the stabilized membrane normalized flux (J/J_0) of QBTS-1 was higher than that of NF 270 for both BSA and SA fouling. After two fouling cycles, with membrane cleaning using pure water at 5 h and 10 h, the flux recovery rates of QBTS-1 for BSA and SA reached 67.9% and 48.6%, respectively, which were

significantly higher than those of NF270 (54.9% and 35.1%). These results indicate that QBTS-1 exhibits superior antifouling performance compared to the commercial NF270 membrane, demonstrating good antifouling properties.

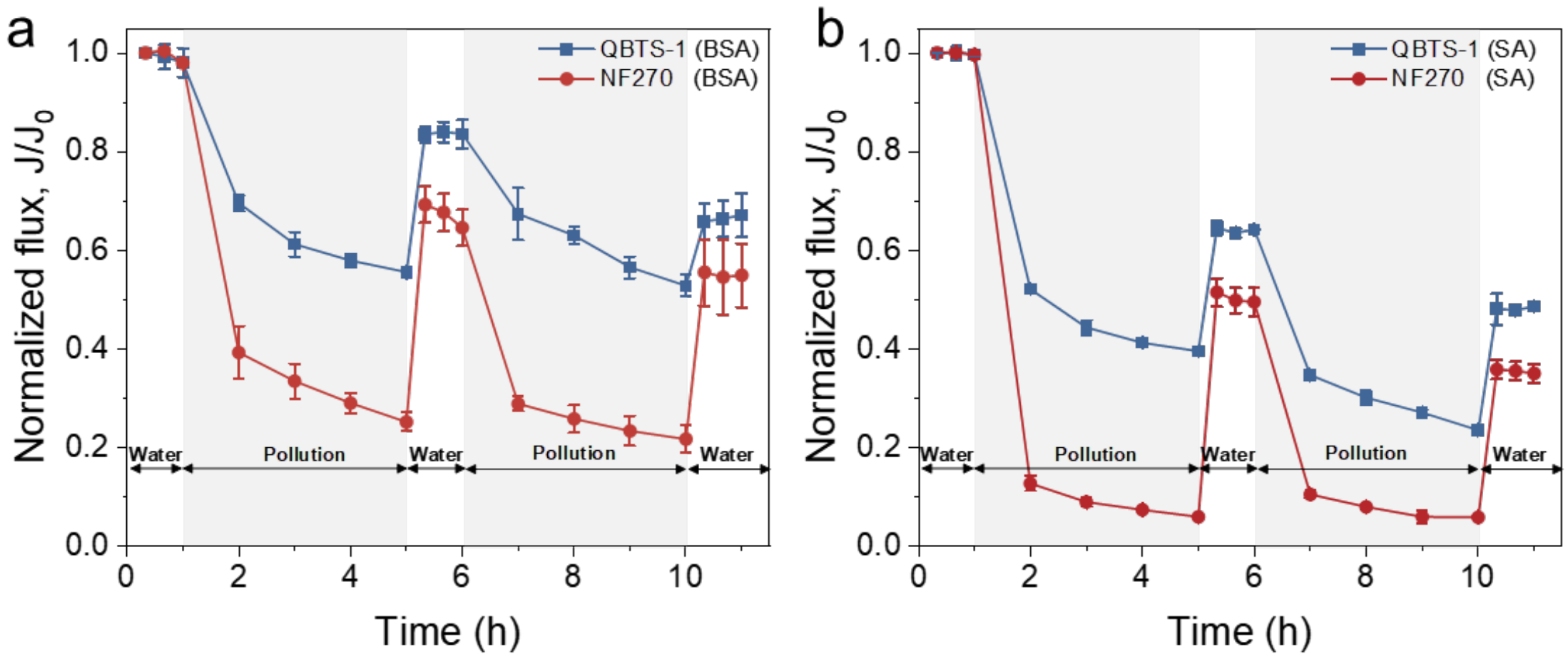

Fig. S16 Analysis of the antifouling performance of QBTS compared with the commercial NF membrane NF 270. (a) Foulant: bovine serum albumin (BSA). (b) Foulant: sodium alginate (SA).

To further investigate the antibacterial properties of the QBTS-1, we selected Gram-negative bacterium *Escherichia coli* (ATCC25922) and Gram-positive bacterium *Staphylococcus aureus* (ATCC29213) as model bacteria for antibacterial tests. As shown in Fig. S17, compared with the control group without a membrane and NF 270, QBTS-1 exhibited antibacterial rates exceeding 99% against both *E. coli* and *S. aureus*, demonstrating significantly better antibacterial performance than NF270. These results indicate that the QBTS-1 membrane prepared in this study possesses excellent antibacterial capability, which is advantageous for its practical application in water treatment scenarios.

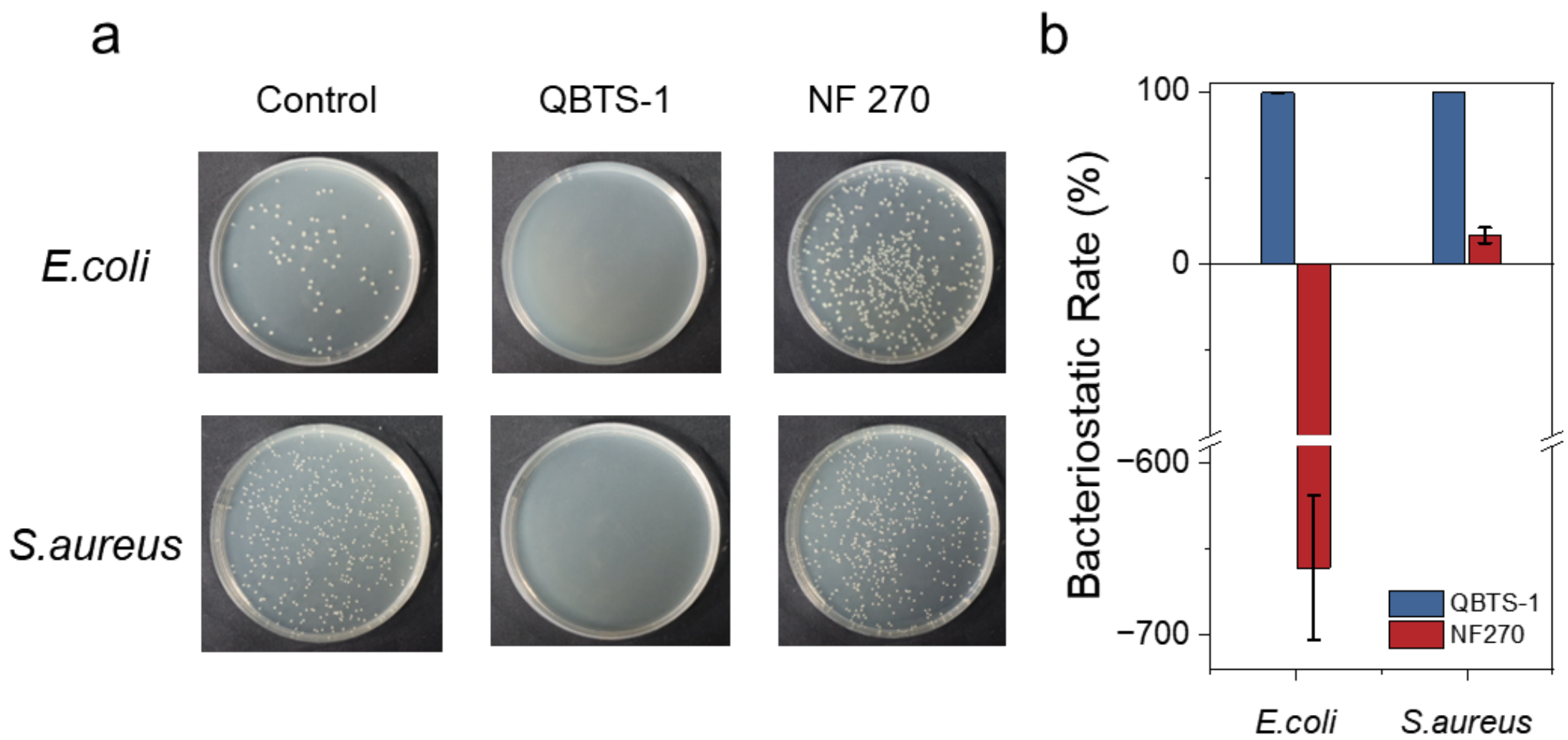

Fig. S17. Comparison of the antibacterial performance of the two nanofiltration membranes. (a) Optical images of *E. coli* and *S. aureus* after 18 h of cultivation (left: membrane-free control; middle: QBTS-1 membrane; right: NF270 membrane). (b) Antibacterial rates of the QBTS-1 and NF270 membranes against the two bacterial strains relative to the membrane-free control.

We have revised the manuscript as follow:

MS-Line 453 Meanwhile, QBTS-1 demonstrated satisfactory flux of other salts (Fig. 4e). As a polyhydroxy quaternary ammonium salt, QBTS also endows the modified membrane surface with good antifouling and antibacterial properties (Fig.S16 and S17). After QBTS interfacial modification, the overall mechanical strength of QBTS-1 remained excellent.

SI-Line 210 Antifouling performance test

The antifouling performance of the nanofiltration membranes was evaluated using bovine serum albumin (BSA) and sodium alginate (SA) as model foulants at a high concentration of 1000 ppm. The tests were conducted in dead-end filtration mode under a constant operating pressure of 12 bar and a solution temperature of 25 °C. Prior to fouling tests, each membrane was stabilized with deionized water for 1 h at 12 bar. The initial pure water flux (J_0) was then measured by sampling every 20 min over 1 h. Thereafter, the feed was switched to the foulant solution, and the flux was monitored for 4 h (sampled hourly), constituting one fouling cycle. After the first cycle, the membrane was cleaned by switching to deionized water and rinsing in cross-flow mode for 1 h to remove the surface fouling layer. A second fouling cycle was then repeated under the same conditions. Following the two cycles, the recovered pure water flux (J_i) was measured again by sampling every 20 min. The flux recovery ratio (FRR) was calculated as $FRR = (J_i / J_0) \times 100\%$.

SI-Line 226 Antibacterial performance test

The antibacterial performance of the QBTS-1 and commercial NF270 nanofiltration membranes was evaluated using *Escherichia coli* (ATCC25922, Gram-negative) and *Staphylococcus aureus* (ATCC29213, Gram-positive) as model bacteria. The detailed procedure is described below.

For the preparation of culture media, Luria-Bertani (LB) liquid medium was prepared by dissolving 2.5 g of LB broth in 100 mL of distilled water and autoclaving at 121 °C for 15 min. LB solid medium was prepared by dissolving 2.5 g of LB broth and 1.5 g of agar in 100 mL of distilled water, autoclaving at 121 °C for 15 min, and pouring into sterile Petri dishes (15 mL per dish) after cooling to 40–50 °C. A 1/500 NB aqueous solution was prepared by dissolving 1.8 g of nutrient broth in 100 mL of deionized water, diluting it 500-fold, and then sterilizing at 115 °C for 30 min. For bacterial suspension preparation, single colonies of *S. aureus* and *E. coli* were separately inoculated into 3 mL of LB liquid medium in 12-mL culture tubes and incubated overnight (15 h) at 37 °C with shaking at 200 rpm, while a tube without inoculation served as the blank control. For sample treatment and contact culture, membrane samples were cut into 2 cm × 2 cm squares and sterilized under UV light for 30 min; cover films (1.5 cm × 1.5 cm) were soaked in alcohol for 1 h and air-dried.

The overnight bacterial culture was diluted with 1/500 NB solution to 10^5 CFU/mL, and then 64 μL of the diluted suspension was evenly dropped onto each membrane surface. A sterilized cover film was placed over the droplet to ensure uniform contact without overflow, and samples were incubated statically at 37 °C for 24 h. For elution, plating, and counting, after incubation the bacteria were eluted with 6.4 mL of sterile PBS, serially diluted 10-fold with sterile PBS, and 100 μL of appropriate dilutions were spread on LB solid plates; after incubation at 37 °C for 18 h, colonies were photographed and counted.

The antibacterial rate of the membrane was calculated as

$$R = \left(1 - \frac{M_i}{M_0}\right) \times 100\%$$

where M_i is the number of bacterial colonies (CFU) recovered from the QBTS-1 or NF270 membrane, and M_0 is the number of colonies from the membrane-free control group.

Reviewer (Comment 2): *Furthermore, the authors should demonstrate the stability of the fabricated nanofiltration membranes, for example, by testing membrane performance under different pressures, salt concentrations, and filtration durations.*

Our Response: Thanks for the comments. Based on the reviewer's suggestion, we conducted a detailed investigation of the stability of the QBTS NF membrane. First, we evaluated the NF performance of QBTS-1 under different pressures. As shown in Fig. 4g, as the pressure increased, the membrane flux increased in a stepwise manner and maintained a good linear relationship ($R^2 > 0.99$). Meanwhile, QBTS-1 maintained a high Na_2SO_4 rejection rate (1000 ppm, 25 °C) under various pressures, indicating good structural stability. Notably, under low pressure (2.5 bar), QBTS still exhibited high flux and salt rejection, making it suitable for low-pressure operation, which helps save energy while maintaining efficient filtration.

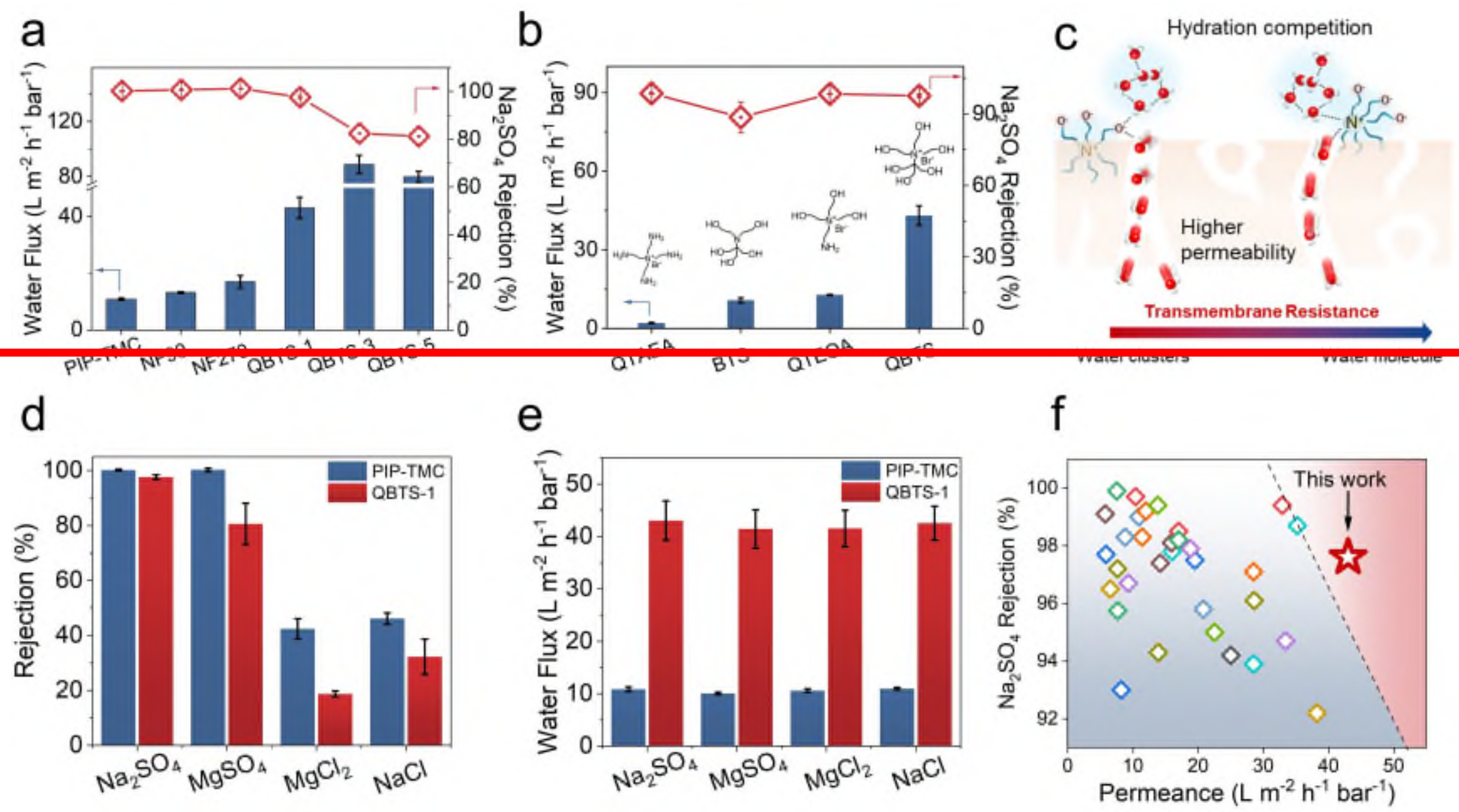

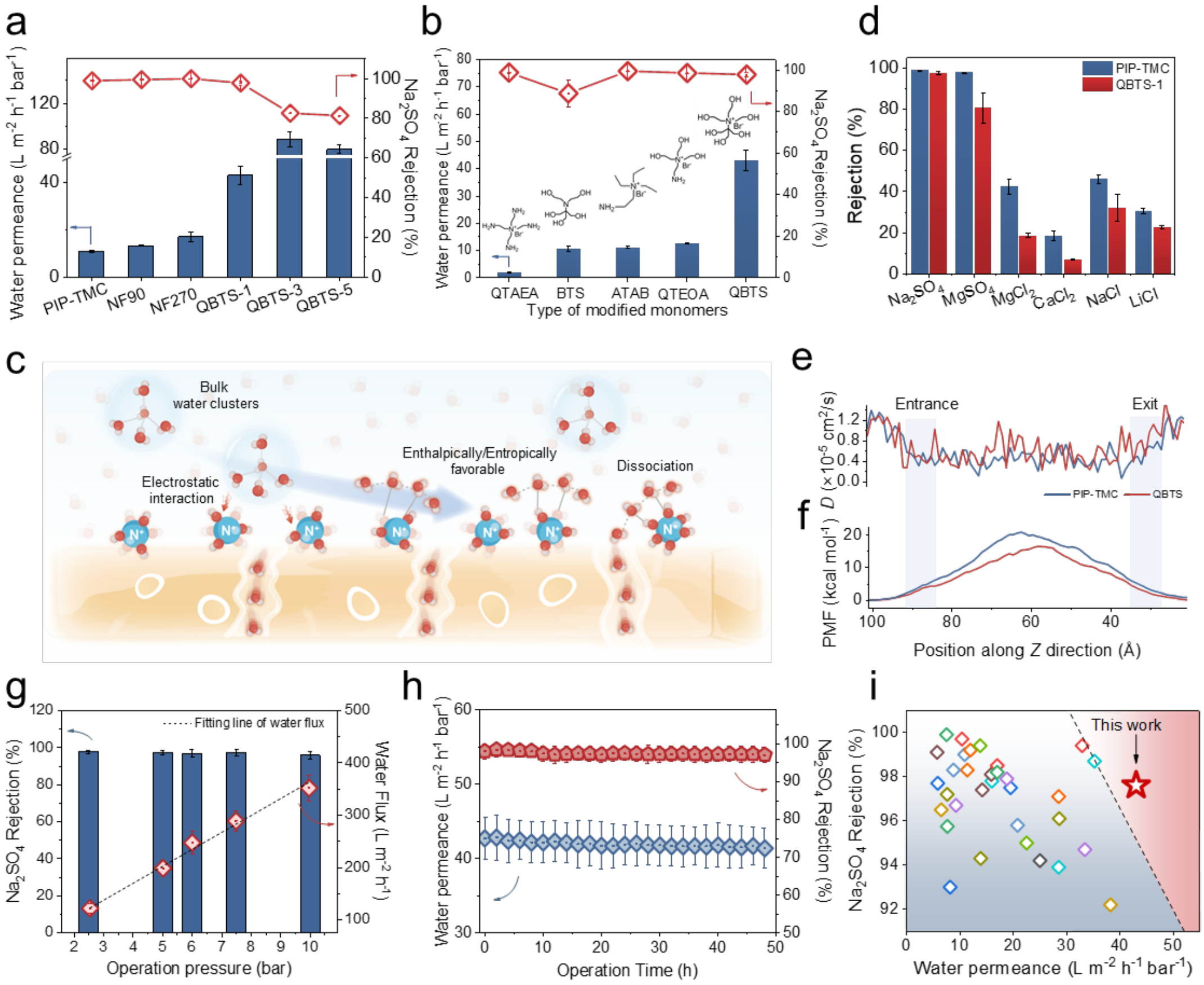

Fig. 4 Filtration performance of QBTS functionalized NF membranes. (a) Effect of QBTS monomer concentration on the separation performance of NF membranes (feed: 1000 ppm Na_2SO_4, 6 bar pressure). (b) Filtration performance of NF membranes modified with different ~~amine~~ monomers (feed: 1000 ppm Na_2SO_4, 6 bar pressure). (c) Schematic diagram of the suppositional mechanism of functional groups promoting the dissociation of water clusters. The dashed lines between water molecules indicate that the original water-water hydrogen bonds are weakened by the polyhydroxy structure of QBTS. (d) Salts rejection ~~and (e) water flux~~ of Na_2SO_4, $MgSO_4$, $MgCl_2$, $CaCl_2$, LiCl and NaCl by PIP-TMC and QBTS-1 membranes (Salt concentration, 1000 ppm; applied pressure, 6 bar~~; temperature, 25 °C~~). (e) The potential of mean force (PMF) and (f) diffusion coefficients (*D*) of Cl^- along the diffusion coordinate in PIP TMC and QBTS simulated nanochannels. The filtration performance of QBTS-1 under different (g) operation pressure and (h) operation Time (feed: 1000 ppm Na_2SO_4, 6 bar pressure). ~~(f)~~ (i) Performance comparison of QBTS-1 membrane with the state-of-the-art NF membranes using surface modification in reported literature (details in Table. S2).

We further investigated the filtration performance of QBTS-1 under different salt concentrations at an operating pressure of 6 bar and temperature of 25 °C. As shown in Fig. S19, as the salt concentration increased, both the membrane flux and salt rejection gradually decreased. The decline in water flux can be attributed to the increased osmotic pressure, which reduced the apparent water flux. The gradual decrease in salt rejection is attributed to the compression of the electrical double layer on the membrane surface under high-salt conditions, which weakens the

Donnan effect and consequently reduces the salt rejection capability of the membrane. Nevertheless, even under a high salt concentration of 10 g/L, the membrane still exhibited a high flux of 108 L m^{-2} h^{-1} and a high salt rejection rate of >90%, indicating that the QBTS-1 nanofiltration membrane maintains good applicability even under high-salinity conditions.

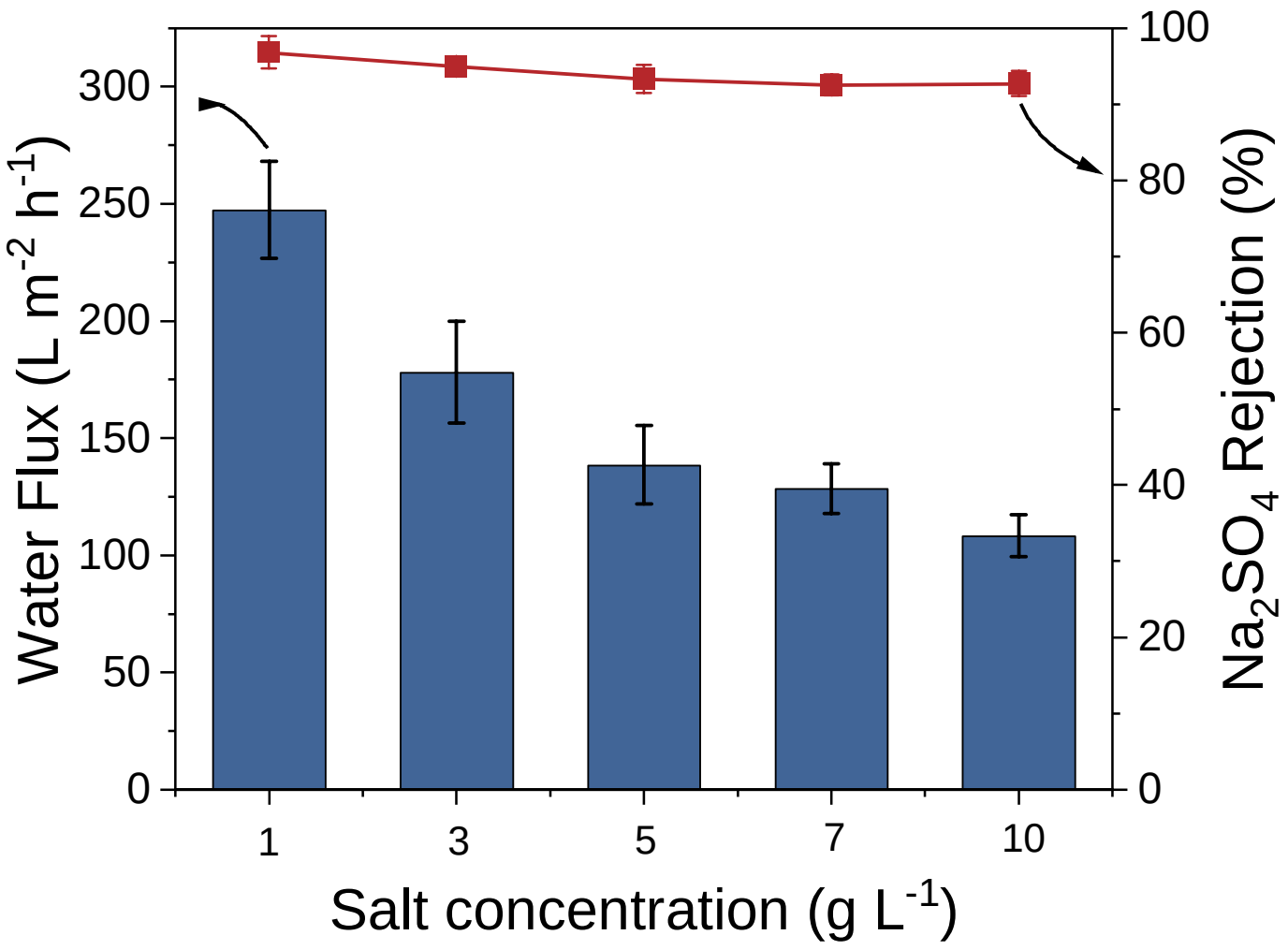

Fig. S19 The filtration performance of QBTS-1 under different salt concentration.

We also analyzed the long-term operational stability of QBTS. As shown in Fig.4h, after 48 h of filtration, both the Na_2SO_4 rejection (for 1000 ppm) and water flux of QBTS-1 remained essentially unchanged, demonstrating that QBTS-1 exhibits excellent stability during long-term operation, which is of great significance for its application in practical water treatment processes.

Accordingly, the manuscript has been revised as below.

MS-Line 110 Under optimal conditions, the functionalized membrane shows great permeability with a membrane flux of 43.01 L m^{-2} h^{-1} bar^{-1}, while maintaining over 97.63% Na_2SO_4 retention performance and ~~mechanical properties~~ satisfied operational stability.

MS-Line 457 After QBTS interfacial modification, the overall mechanical strength of QBTS-1 remained excellent (Fig. S10). In addition, we evaluated the stability of the QBTS membrane performance under different operating pressures, various salt concentrations, and long-term operation. As shown in Fig. 4g, QBTS-1 maintained good rejection and permeation performance within the operating pressure range of 2.5-10 bar. Even under high-salt conditions of 10 g/L, it still exhibited good salt rejection (Fig. S19). Furthermore, it also demonstrated long-term operational stability (Fig. 4h).

Reviewer (Comment 3)*: *Could the authors further investigate the influence of the functional groups on H-bond lifetime to provide additional evidence supporting their role in water cluster dissociation?

Our Response: We thank the reviewer's comment. As shown in Fig. 2e, we compared the H-bond lifetime of water-water and that between water molecules and the polyhydroxy quaternary ammonium functional group of QBTS in an aqueous box (the specific method is provided later). As the simulation time increased, the number of H-bonds between QBTS and nearby water molecules gradually increased Fig. S3), indicating that the interaction between QBTS and water molecules gradually strengthened. Meanwhile, the H-bond lifetime between QBTS and water molecules was 9.69 ps (Fig. 2f), which is longer than that between water molecules, suggesting that QBTS can preferentially "capture" water molecules from water-water networks. Numerous computational and even experimental studies have confirmed that the preferential interaction between functional group such -OH with water molecules within water clusters can disrupt or even reconstructing the water cluster structure[1, 2, 3, 4, 5]. Combined with the interaction energy data in Fig. 1c, we speculate that the polyhydroxy quaternary ammonium structure may facilitate the dissociation of water clusters.

We have added additional statement to clarify the influence of the functional groups on H-bond lifetime to provide additional evidence supporting their role in water cluster dissociation.

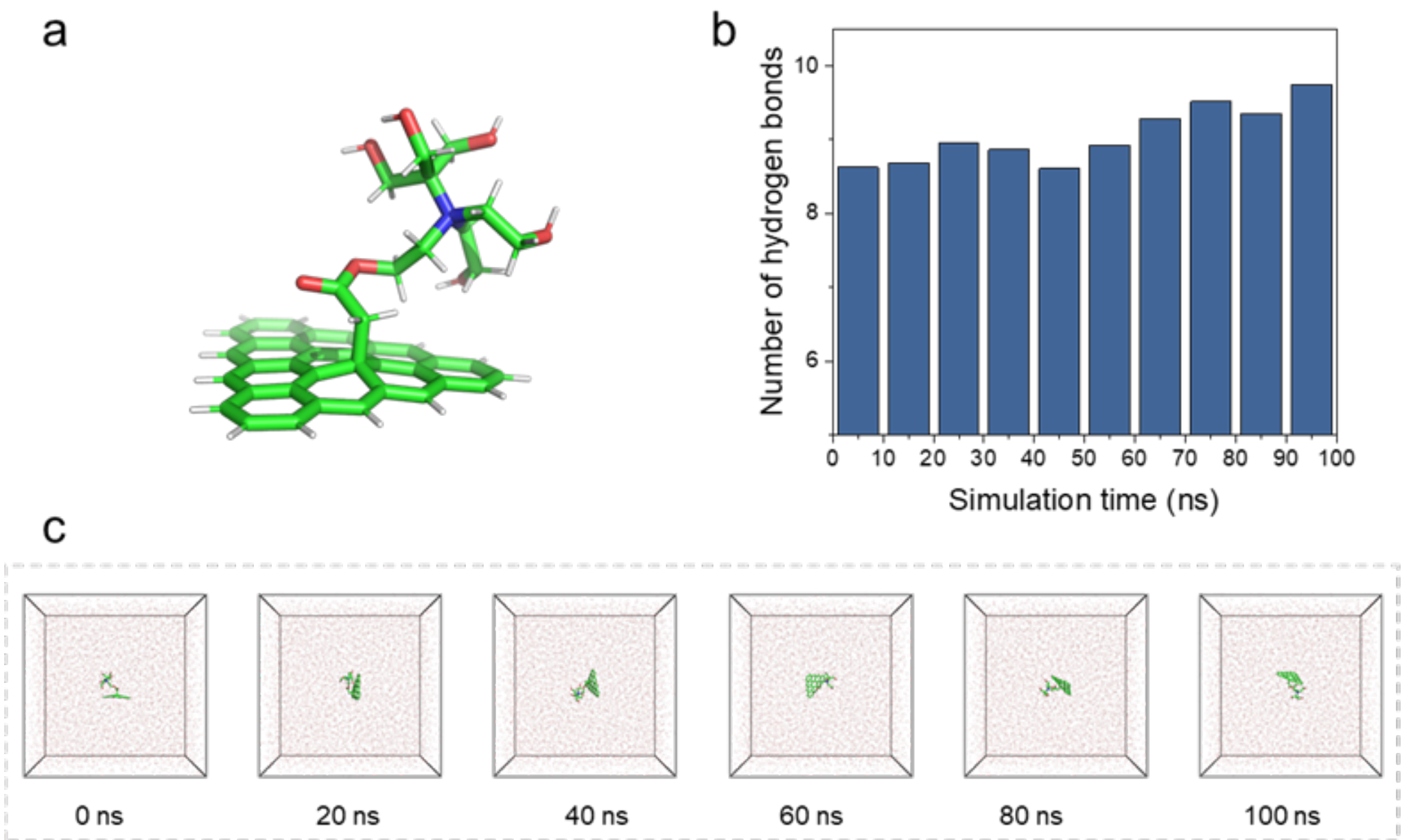

Fig. S3 Simulation details for calculating H-bonds lifetime. (a) A graphene grafted by QBTS unit. (b) The variation of H-bonds number between QBTS and nearby water molecules as the simulation time increased. (c) The simulation aqueous box at different

simulation time.

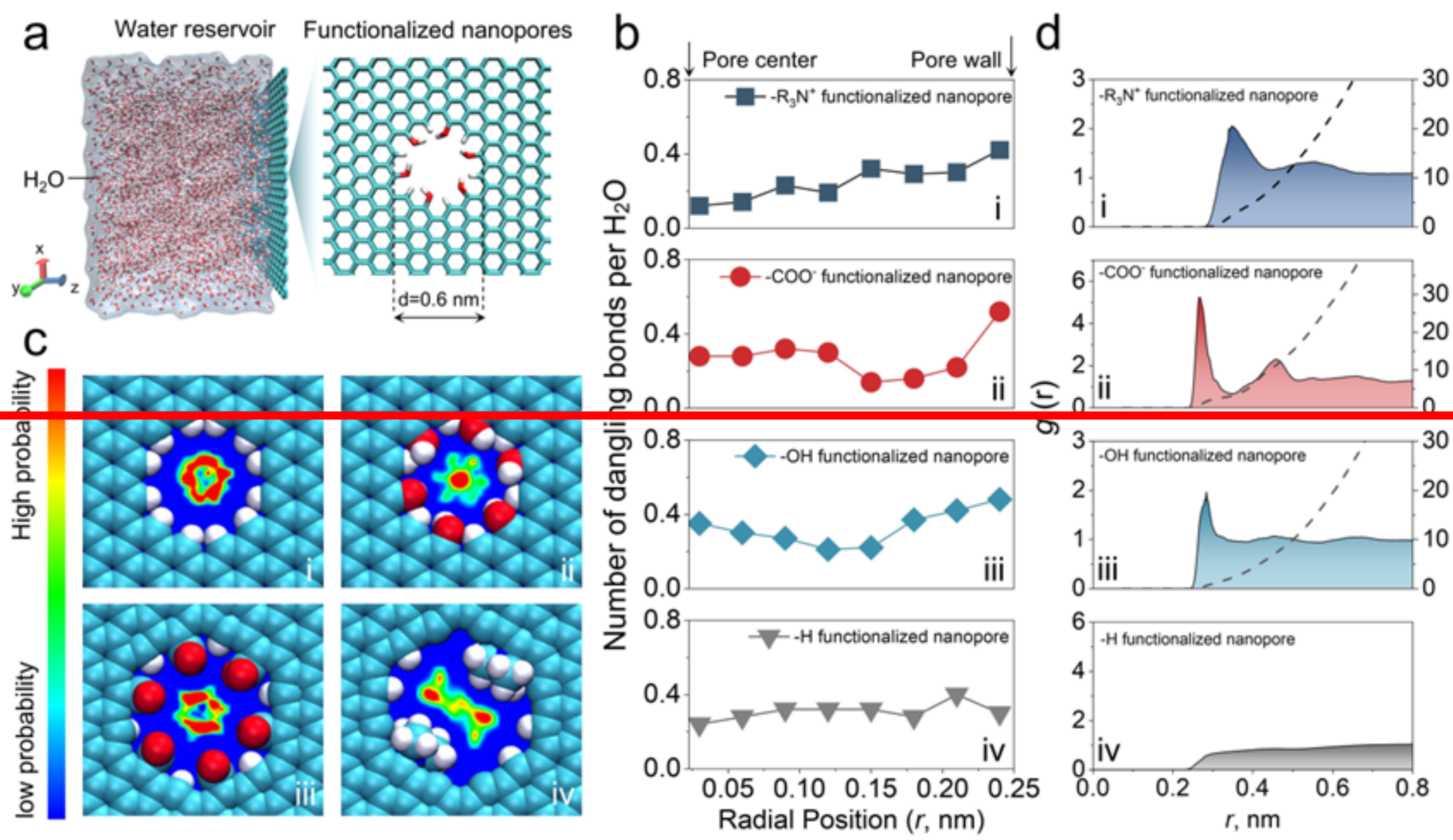

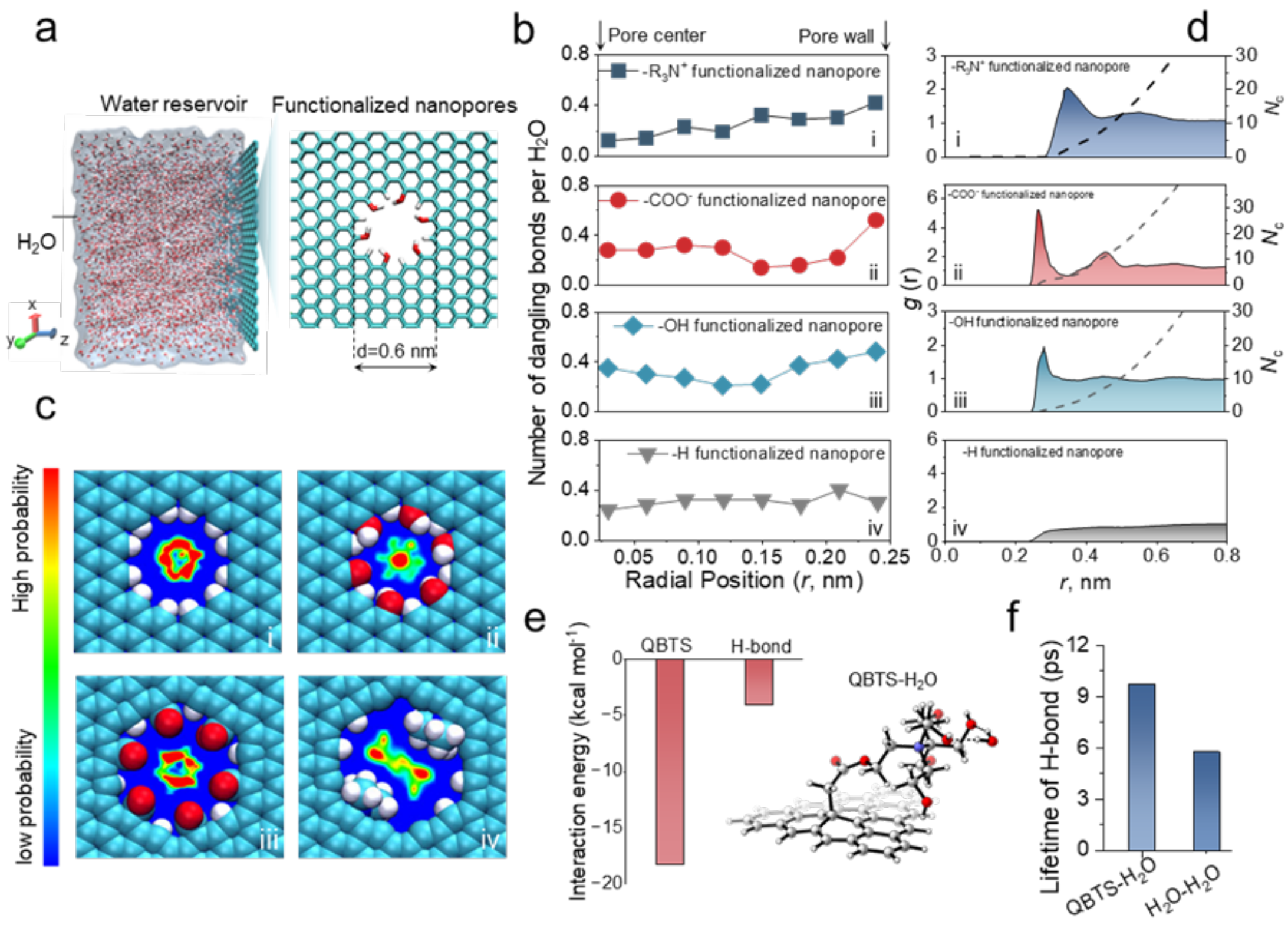

Fig. 2 Mechanisms of water cluster-dissociation from hydration competition by functional groups. (a) Side view of the MD simulation system investigated in this work. (b) Dangling bond radial distribution of water clusters in different functionalized pores. (c) Oxygen density maps in different functionalized pores, i-iv represents -H, -OH, $-COO^-$ and $-R_3N^+$ functionalized pores, respectively. (d) RDFs between water molecule and the functional groups, and the corresponding coordination numbers (N_c), i.e., N-O_w, O-H_w, and H-O_w for $-R_3N^+$, $-COO^-$ and -OH,

respectively. (e) The interaction energy between a single water molecule and the QBTS functional group. The inset shows the double-sites interaction between QBTS and water molecule. (f) The H-bond lifetime of QBTS-water and water-water calculating by MD simulation.

MS-Line 235 In contrast, we propose that the polyhydroxy quaternary ammonium structure could further facilitate the dissociation of water clusters—the positively charged ~~R_3N^+~~ quaternary-N^+ center attracts water clusters to the active sites via electrostatic adsorption, and the polyhydroxy network with rich hydrogen-bond donating sites, can more effectively promote cluster dissociation. DFT calculation results show that the polyhydroxy quaternary ammonium structure indeed exhibits much stronger functional group-water interaction energies (-18.3 kcal mol^{-1}) than carboxyl and hydroxyl groups (Fig. 1c and Fig. 2e). Meanwhile, H-bonds with longer lifetimes relative to the water-water network can form near QBTS (Fig. 2f and Fig. S3), indicating that compared with water itself, water molecules prefer to interact with the polyhydroxy structure. Combining these results, we speculate that the polyhydroxy quaternary ammonium structure may facilitate the dissociation of water clusters, showing potential to facile water transport.

SI-Line 412 Hydrogen bond characteristic simulation between functional groups and water molecules.

RESP charges were fitted using MultiWFN, and GAFF2 force field parameters for the corresponding molecules were generated using Sobtop. GAFF2 is an improved version of GAFF (General Amber Force Field), specifically designed to cover a broad range of organic chemical space, including well-parameterized oxygen-containing functional groups, nitrogen-containing groups, and aromatic systems. Molecular dynamics simulations were performed using the Gromacs 2021 software. Gromacs is a widely used open-source molecular dynamics simulation software, primarily employed for simulating biomolecular systems such as proteins, lipids, nucleic acids, etc., as well as various molecular systems in materials science. The force field used in this simulation was GAFF2, and the water model was TIP3P. A simulation box of 6 × 6 × 6 nm^3 was constructed, with the multi-hydroxy quaternary ammonium group-modified graphene molecule placed in the center of the box, and the box was filled with water. Na^+ and Cl^- were used to balance the system charge, rendering the overall system electrically neutral. Prior to simulation, energy minimization was first performed to eliminate unreasonable contacts in the initial conformation. This was followed by a 100 ps NVT pre-equilibration simulation. The NVT ensemble refers to a thermodynamic ensemble that maintains constant particle number (N), volume (V), and temperature (T) during the simulation, used to raise the temperature to the target simulation temperature. Subsequently, a 100 ps NPT pre-equilibration simulation was executed. The NPT ensemble refers to a thermodynamic ensemble that maintains constant particle number (N), pressure (P), and temperature (T) during the simulation, used to adjust the system density to the equilibrium value at the target pressure. After system relaxation, the production simulation was carried out. The production simulation used the leap-frog algorithm to integrate Newton's equations of motion, with an integration time step of 2 fs and a total of 50,000,000 integration steps, resulting in a total simulation time of 100.0 ns. The production simulation employed the V-rescale temperature coupling method, with a simulation temperature of 298.15 K. The production simulation used the Parrinello-Rahman pressure coupling method, with a simulation pressure of 1.0 bar. Due to the use of a cutoff distance method, the Particle Mesh Ewald (PME) method was applied to correct for long-range electrostatic interactions. Also due to the use of a cutoff distance method, dispersion correction was applied for long-range van der Waals interactions. Periodic boundary conditions were applied in all three directions throughout the simulation.

References:

1. Shin S, Willard AP. Water's Interfacial Hydrogen Bonding Structure Reveals the Effective Strength of Surface-Water Interactions. *J Phys Chem B* **122**, 6781-6789 (2018).

2. Zhou L, *et al.* Suppressing Hydrogen Evolution in Aqueous Lithium-Ion Batteries with Double-Site Hydrogen Bonding. *ACS Energy Letters* **8**, 40-47 (2022).

3. Ng LS, *et al.* Chaotropic Nanoelectrocatalysis: Chemically Disrupting Water Intermolecular Network at the Point-of-Catalysis to Boost Green Hydrogen Electrosynthesis. *Angew Chem Int Ed Engl* **63**, e202317751 (2024).

4. Zhu Y, Chen Q, Hao J, Jiao Y. Breaking hydrogen-bonds in aqueous electrolytes towards highly reversible zinc-ion batteries. *Journal of Materials Chemistry A* **12**, 20097-20106 (2024).

5. Guo L, *et al.* Formate-Anion-Induced Water Network Reshaping Enables Concurrent Hydrogen and Magnesium Hydroxide Production From Seawater. *Angew Chem Int Ed Engl*, e6937994 (2026).

Reviewer (Comment 4): *The authors conducted extensive simulation analyses to explain the molecular mechanism of water cluster dissociation. Please further elaborate on the rationale for selecting different models (e.g., graphene pores, polyamide models).*

Our Response: We thank the reviewer's comment. In our study, different models were used to address different scientific questions. Specifically, as shown in Fig. 2a and Fig. S2, to evaluate the effect of functional groups on the structure of water clusters, we constructed a functionally modified perforated graphene model with a simplified model. By contrast, to explore the microscopic mechanism by which the QBTS-modified nanofiltration membrane affects water transport/permeability, we retained the characteristics of polyamide and constructed a QBTS-modified polyamide nanopore. The rationale for choosing these models is as follows:

i) For the question of how functional groups influence water cluster structure, perforated graphene is relatively inert, has a well-defined pore boundary, and allows convenient variation of only the functional group type at the pore orifice (i.e., -H, -OH, -COOH, and -R_3N^+). This serves as a "elementary model" of the mechanism. Compared with the complex pore size distribution and structural heterogeneity of a polyamide network, functionalized perforated graphene enables a clean analysis of the effects of functional groups on water cluster structure (e.g., DBs, RDF), following approaches widely used in the literature [1, 2, 3, 4, 5, 6]. This helps us focus specifically on the intrinsic influence of the functional groups themselves on water clusters.

ii) For the mechanism by which the QBTS-modified membrane affects water transport/permeability, it is necessary to retain the features of a polyamide nanofiltration membrane. Therefore, we constructed a polymeric nanochannel using polyamide building blocks and grafted QBTS at the pore orifice, preserving the essential characteristics of the QBTS nanofiltration membrane prepared in this experiment, as has been done in many previous studies[7, 8, 9, 10]. Through this nanopore, we simulated the water cluster transport process to fully understand the microscopic events (e.g., H-bond distribution, MSD) that occur when water clusters pass through a "realistic" nanopore.

Therefore, these two models are complementary rather than redundant: the former

is used for mechanistic isolation, and the latter for mechanistic validation in a membrane-relevant transport scenario. We will revise the manuscript to clarify this rationale.

MS-Line 179 As illustrated in Fig 2a, a series of inert graphene pores functionalized at the edges with -H, -OH, -COO^-, and -R_3N^+ were constructed to investigate the influence of functional groups on ~~water molecule transport~~ structural changes of water clusters during transport.

SI-Line 299 In order to investigate the hydration competition mechanism of functional groups, we constructed four pores with a pore size of 0.6 nm on the surface of monolayer graphene. On the inner surfaces of these pores, we functionalized -H, -OH, -COO^-, and -R_3N^+ groups, respectively. Such a simplified perforated graphene serves as a "elementary model" of the mechanism. Compared with the complex pore size distribution and structural heterogeneity of a polyamide network, functionalized perforated graphene enables a clean analysis of the effects of functional groups on water cluster structure (e.g., DBs, RDF), following approaches widely used in the literature.

SI-Line 347 In order to study the transport behavior of $(H_2O)_nH^+$ in two types of polyamide nanochannels, two simulated nanochannel models were constructed, namely PIP-TMC and QBTS (Fig. S24). It is worth noting that this nanochannel is composed of polyamide units, thus retaining the basic physicochemical characteristics of the polyamide nanofiltration membrane prepared in this experiment.

References:

1. Cohen-Tanugi D, Grossman JC. Water Desalination across Nanoporous Graphene. *Nano Letters* **12**, 3602-3608 (2012).

2. Shi Q, He Z, Gupta KM, Wang Y, Lu R. Efficient ethanol/water separation via functionalized nanoporous graphene membranes: insights from molecular dynamics study. *Journal of Materials Science* **52**, 173-184 (2016).

3. Fang C, Yu Z, Qiao R. Impact of Surface Ionization on Water Transport and Salt Leakage through Graphene Oxide Membranes. *The Journal of Physical Chemistry C* **121**, 13412-13420 (2017).

4. Yu T, Xu Z, Liu S, Liu H, Yang X. Enhanced hydrophilicity and water-permeating of functionalized graphene-oxide nanopores: Molecular dynamics simulations. *Journal of Membrane Science* **550**, 510-517 (2018).

5. Ouyang J, Sheng Y, Wang W. Water Transport Dynamics in Layered Nanoporous Graphene: An In-Depth Molecular Dynamics Study. *The Journal of Physical Chemistry B* **129**, 8521-8540 (2025).

6. He Z, Zhou J, Lu X, Corry B. Bioinspired graphene nanopores with voltage-tunable ion

selectivity for Na(+) and K(+). *ACS Nano* **7**, 10148-10157 (2013).

7. Lu C, *et al.* In Situ Characterization of Dehydration during Ion Transport in Polymeric Nanochannels. *J Am Chem Soc* **143**, 14242-14252 (2021).

8. Wang P, *et al.* Ultrafast ion sieving using nanoporous polymeric membranes. *Nature Communications* **9**, 569 (2018).

9. Cruz-Chu ER, Ritz T, Siwy ZS, Schulten K. Molecular control of ionic conduction in polymer nanopores. *Faraday Discussions* **143**, 47-62 (2009).

10. Lu C, *et al.* Dehydration-enhanced ion-pore interactions dominate anion transport and selectivity in nanochannels. **9**, eadf8412 (2023).

Reviewer (Comment 5): *The authors observed a significant decrease in the rejection of $MgCl_2$ by the QBTS-1 membrane and attributed this to the promotion of ion dehydration by -OH and -R_3N^+. Is this claim supported by evidence? This point needs further discussion and insight.*

Our Response: Thanks for the comments. Indeed, it is not sufficiently comprehensive and detailed to attribute the reduction in $MgCl_2$ rejection solely to the "promotion of ion dehydration by -OH and -R_3N^+". According to the widely accepted nanofiltration separation mechanism[1, 2, 3, 4], salt rejection is typically determined by the ion with lower permeability[5, 6]. Factors affecting ion permeability include membrane surface charge (Donnan effect)[6], membrane pore size (size sieving)[6], and ion-membrane interactions (adsorption)[7, 8], among others. As shown in Fig. R1, previous studies have demonstrated that the rejection of different salt types responds distinctly to membrane surface charge [1, 5].

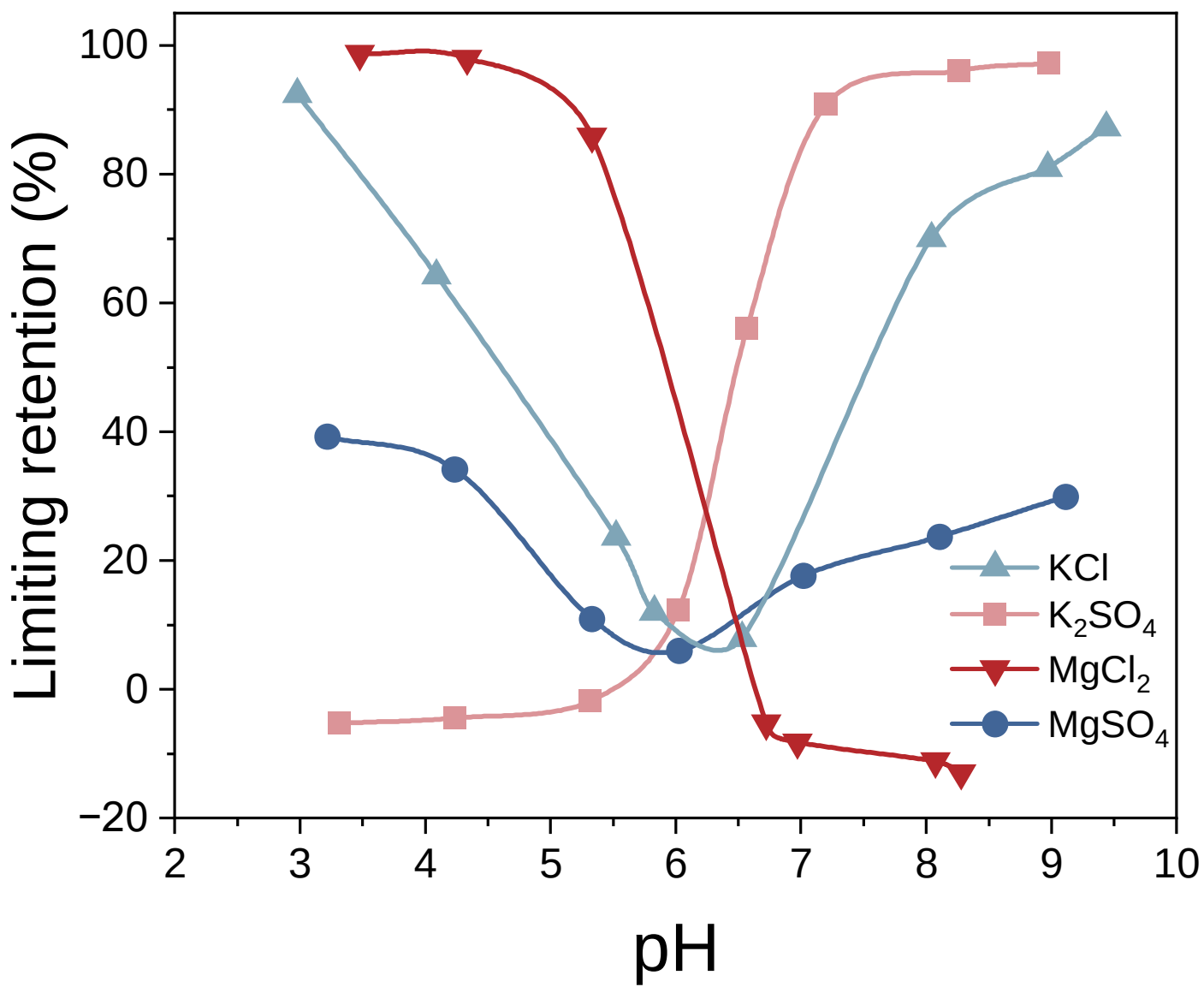

Fig. R1 Limiting retention of single solutions as a function of pH at fixed concentration of 1 mmol L^{-1}. Figure adapted from [1, 5].

For $MgCl_2$, the salt rejection is determined by the Mg^{2+}. Since the pore size distributions of QBTS-1 and PIP-TMC were almost identical (Fig. 3d), we speculate that this reduction in salt rejection originates from the unique surface characteristics of QBTS. First, the lower Zeta potential of QBTS facilitates the adsorption of cations onto the membrane surface (Fig. 3j). Second, the polyhydroxy quaternary ammonium structure can complex with cations[9, 10, 11, 12], particularly Mg^{2+}, further enhancing the affinity between the cations and the membrane. These Mg^{2+} ions adsorbed onto the membrane surface further induce charge neutralization[7, 8, 13], thereby weakening the repulsion of the membrane surface negative charge against co-ions.

Finally, as shown in Fig. 4e and Fig. 4f, we calculated the potential of mean force (PMF) and diffusion coefficients of Cl^- along the diffusion coordinate in PIP-TMC and QBTS simulated nanochannels. Unexpectedly, the PMF of Cl^- in QBTS was lower, decreasing from 20.75 to 16.39 kcal mol^{-1} (Fig. 4f). Meanwhile, as shown in Fig.4e, during transport through the QBTS pore, Cl^- exhibited several favorable transport regions, such as near the pore entrance (~90 Å) and at the pore center (~60 Å), showing higher diffusion coefficients. Using the inhomogeneous solubility-diffusion model, the calculated permeability coefficient across the entire membrane increased correspondingly from 1.10×10^{-13} to 1.20×10^{-10} cm s^{-1}, indicating that Cl^- is more easily transported in QBTS. This phenomenon seems counterintuitive for QBTS, which has a more negative zeta potential. However, similar observations have been reported in many nanopores with -OH-modified entrances[14, 15, 16]. Based these previous studies, we speculate two possible reasons for the reduction of Cl^- rejection: i) the polyhydroxy structure in QBTS can interact

with Cl^- through a "dehydration compensation" mechanism[14, 17, 18], replacing some of the bound water lost from the hydrated Cl^-, thereby stabilizing the dehydration process of Cl^- and lowering the dehydration energy barrier; ii) the quaternary-N^+ near the pore entrance may help direct Cl^- toward the pore by electrostatic adsorption, making Cl^- transport more favorable.

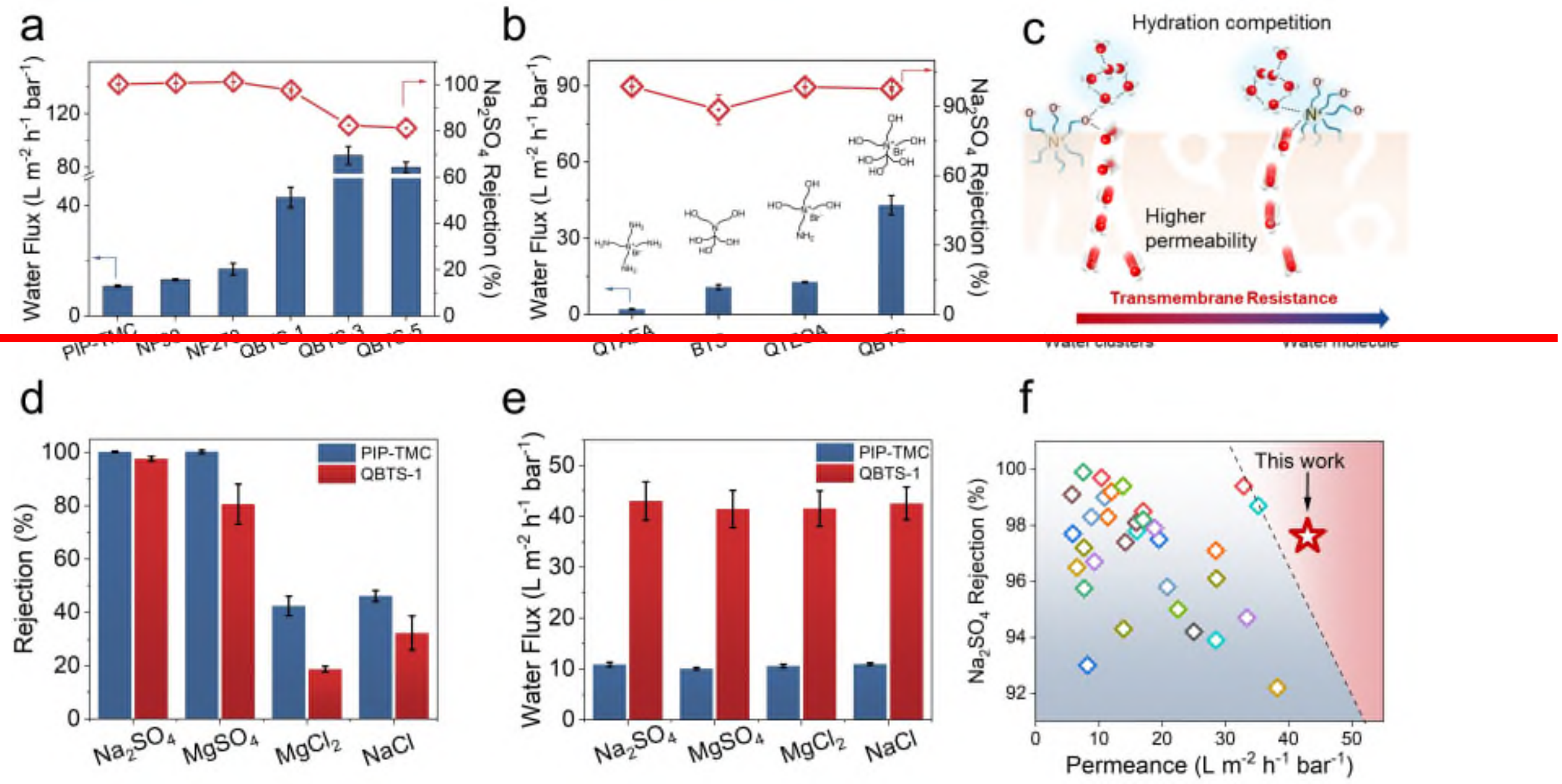

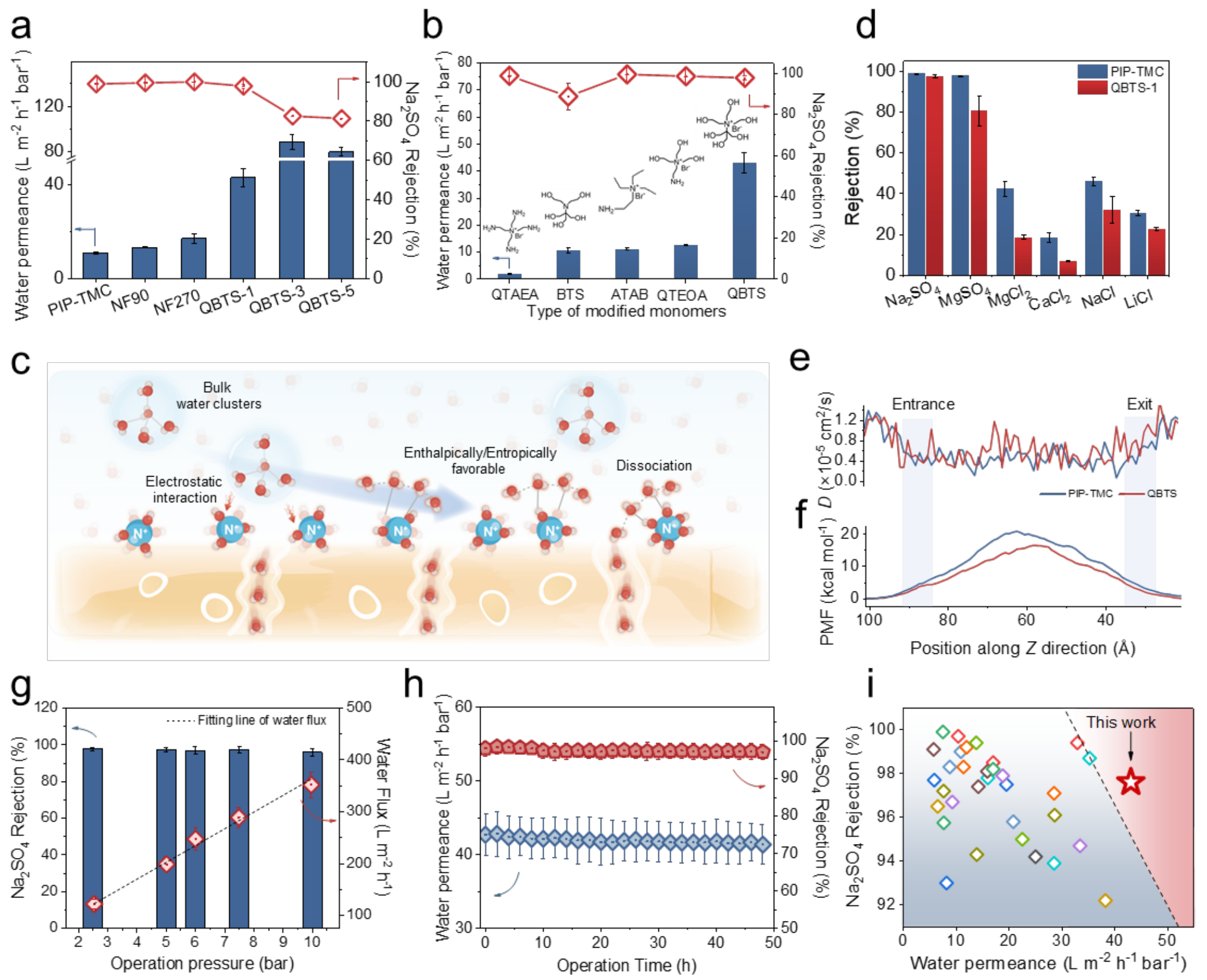

Fig. 4 Filtration performance of QBTS functionalized NF membranes. (a) Effect of QBTS monomer concentration on the separation performance of NF

membranes (feed: 1000 ppm Na_2SO_4, 6 bar pressure). (b) Filtration performance of NF membranes modified with different amine monomers (feed: 1000 ppm Na_2SO_4, 6 bar pressure). (c) Schematic diagram of the suppositional mechanism of functional groups promoting the dissociation of water clusters. The dashed lines between water molecules indicate that the original water-water hydrogen bonds are weakened by the polyhydroxy structure of QBTS. (d) Salts rejection and (e) water flux of Na_2SO_4, $MgSO_4$, $MgCl_2$, $CaCl_2$, LiCl and NaCl by PIP-TMC and QBTS-1 membranes (Salt concentration, 1000 ppm; applied pressure, 6 bar; temperature, 25 °C). (e) The potential of mean force (PMF) and (f) diffusion coefficients (*D*) of Cl^- along the diffusion coordinate in PIP TMC and QBTS simulated nanochannels. The filtration performance of QBTS-1 under different (g) operation pressure and (h) operation Time (feed: 1000 ppm Na_2SO_4, 6 bar pressure). (f) (i) Performance comparison of QBTS-1 membrane with the state-of-the-art NF membranes using surface modification in reported literature (details in Table. S2).

These effects synergistically enhance salt partitioning and thus the permeability, thereby leading to a decrease in salt rejection. Compared with SO_4^{2-}, Cl^- has a smaller hydrated size and less negative charge. Therefore, $MgCl_2$ is more susceptible to the aforementioned factors, resulting in a lower rejection. We have also revised the relevant state to better clarify the reasons for the change in QBTS rejection.

MS-Line 423 Simultaneously, we observed that QBTS-1 slightly reduced the retention of the four salts, particularly for salts containing monovalent Cl^-, i.e., $MgCl_2$, CaCl, LiCl and NaCl, which is reasonably attributed to that -OH and -R_3N^+ on the membrane surface breaking the hydrogen bonds within the water molecule clusters while assisting in the dehydration of hydrated ions and facilitating the transport of ions. We thus used molecular dynamics to investigate ion rejection behavior in simulated polyamide nanochannels (details in *Supplementary information*). As shown in Fig. 4f, we calculated the potential of mean force (PMF) and diffusion coefficients of Cl^- along the diffusion coordinate in PIP TMC and QBTS simulated nanochannels. Unexpectedly, the PMF of Cl^- in QBTS was lower, decreasing from 20.75 to 16.39 kcal mol^{-1}. Meanwhile, as shown in Fig. 4e, during transport through the QBTS pore, Cl^- exhibited several favorable transport regions, such as near the pore entrance (~90 Å), showing higher diffusion coefficients. Using the inhomogeneous solubility-diffusion model, the calculated permeability coefficient across the entire membrane increased correspondingly from 1.10×10^{-13} to 1.20×10^{-10} cm s^{-1}, indicating that Cl^- is more easily transported in QBTS. This phenomenon seems counterintuitive for QBTS, which has a more negative zeta potential. However, similar observations have been reported in many nanopores with -OH-modified entrances. Based these previous studies, we speculate two possible reasons for the reduction of Cl^- rejection: i) the polyhydroxy structure in QBTS can interact with Cl^- through a “dehydration compensation” mechanism, replacing some of the bound water lost from the hydrated Cl^-, thereby stabilizing the dehydration process of Cl^- and lowering the dehydration energy barrier; ii) the quaternary-N^+ near the pore entrance may help direct Cl^- toward the pore by electrostatic adsorption, making Cl^- transport more favorable. In addition, the polyhydroxy quaternary structure of QBTS can complex with cations, particularly Mg^{2+}, further enhancing the affinity between the cations and the membrane. These Mg^{2+} adsorbed onto the membrane surface further induce charge neutralization, thereby weakening the repulsion of the membrane surface negative charge against co-ions. These effects together result in a more pronounced decrease in rejection of $MgCl_2$, NaCl, LiCl, and $CaCl_2$ after QBTS modification.

References:

1. Luo J, Wan Y. Effects of pH and salt on nanofiltration—a critical review. *Journal of Membrane Science* **438**, 18-28 (2013).

2. Shefer I, Lopez K, Straub AP, Epsztein R. Applying transition-state theory to explore transport and selectivity in salt-rejecting membranes: a critical review. *Environmental Science & Technology*, (2022).

3. Wijmans JG, Baker RW. The Solution-Diffusion Model - a Review. *Journal of Membrane Science* **107**, 1-21 (1995).

4. Epsztein R, Shaulsky E, Dizge N, Warsinger DM, Elimelech M. Role of ionic charge density in donnan exclusion of monovalent anions by nanofiltration. *Environmental Science & Technology* **52**, 4108-4116 (2018).

5. Szabolcs Szoke GP, Laszlo Weiser. Characteristics of thin-film nanofiltration membranes at

various pH-values. *Desalination* **151**, 123-129 (2002).

6. Cevallos-Cueva N, Rahman MM, Hailu Kinfu H, Abetz V. Mass transport mechanism of nitrate selective nanofiltration membranes on the basis of the Donnan steric pore model with dielectric exclusion (DSPM-DE). *Chemical Engineering Journal* **493**, (2024).

7. Guo H-y, Gao X-q, Yu K-c, Wang X-m, Liu S-m. Ion adsorption on nanofiltration membrane surface and its effect on rejection of charged solutes: A zeta potential approach. *Separation and Purification Technology* **326**, 124830 (2023).

8. Roth RS, Birnhack L, Avidar M, Hjelvik EA, Straub AP, Epsztein R. Effect of solution ions on the charge and performance of nanofiltration membranes. *npj Clean Water* **7**, (2024).

9. Al-Sogair FM, Operschall BP, Sigel A, Sigel H, Schnabl J, Sigel RKOJCr. Probing the metal-ion-binding strength of the hydroxyl group. **111 8**, 4964-5003 (2011).

10. Gyurcsik B, Nagy L. Carbohydrates as ligands: coordination equilibria and structure of the metal complexes. *Coordination Chemistry Reviews* **203**, 81-149 (2000).

11. Allscher T, Klüfers P, Mayer P. Carbohydrate-Metal ComplexesCarbohydrate–metal complexes: Structural Chemistry of Stable Solution Species. In: *Glycoscience: Chemistry and Chemical Biology* (eds Fraser-Reid BO, Tatsuta K, Thiem J). Springer Berlin Heidelberg (2008).

12. Tajmir-Riahi HA. Magnesium-sugar interaction: Synthesis, spectroscopic and structural characterization of Mg-sugar complexes containing β-d-fructose. *Biophysical Chemistry* **23**, 223-228 (1986).

13. Schaep J, Vandecasteele C, Mohammad AW, Bowen WR. Modelling the retention of ionic components for different nanofiltration membranes. *Separation and Purification Technology* **22-3**, 169-179 (2001).

14. Cohen-Tanugi D, Grossman JC. Water Desalination across Nanoporous Graphene. *Nano Letters* **12**, 3602-3608 (2012).

15. Corry B. Water and ion transport through functionalised carbon nanotubes: implications for desalination technology. *Energy & Environmental Science* **4**, (2011).

16. Guerrero-Aviles R, Orellana W. Energetics and diffusion of liquid water and hydrated ions through nanopores in graphene: ab initio molecular dynamics simulation. *Phys Chem Chem Phys* **19**, 20551-20558 (2017).

17. Wang M, *et al.* A coupled effect of dehydration and electrostatic interactions on selective ion transport through charged nanochannels. *Nanoscale* **10**, 18821-18828 (2018).

18. He Z, Zhou J, Lu X, Corry B. Bioinspired graphene nanopores with voltage-tunable ion selectivity for Na^+ and K^+. *ACS Nano* **7**, 10148-10157 (2013).

Reviewer (Comment 6): *In practice, the functional groups discussed by the authors are quite common in membrane separation processes. Is the observed phenomenon universal? Does this effect also exist in other high-flux nanofiltration membranes?*

Our Response: We thank the reviewer for the insightful comments. We would first like to make two points:

i) The structure of water clusters in nanochannels has been demonstrated to be closely related to water transport rate/water flux[1, 2, 3, 4]. In highly confined polyamide membranes, water is transported in the form of clusters smaller than those in the bulk phase[1, 2], which requires energy consumption[1];

ii) A large body of literature has confirmed that functional groups can indeed alter the hydrogen-bond network of water through strong interactions[5, 6, 7, 8, 9, 10, 11]. For example, surface hydroxyl groups can form new interfacial H-bonds with adjacent water molecules, weakening the water-water H-bonds within water clusters and creating a weakly hydrogen-bonded interface that reduces water activity[5]. The introduction of caprolactam, which contains both hydrogen-bond donors and acceptors, effectively confines water molecules in a dual-site anchoring configuration, enhancing hydrogen-bond interactions, and disrupting the original H-bonds among water molecules[6]. Pentaerythritol, with its unique symmetric structure bearing four hydroxyl groups (-OH), significantly strengthens its interaction with water molecules and alters the proportions of different H-bond types among water molecules (strong H-bonds decrease while weak H-bonds increase)[10]. In summary, functional groups, especially hydroxyl or carboxyl groups, can "break" the water cluster structure and destabilize water through mechanisms such as hydration competition, oriented locking of water molecules, and local electric field construction[5, 6, 7, 8, 9, 10, 11]. Since the influence of the above-mentioned functional groups on the structure of water clusters has been observed in various scenarios—such as membrane separation, electrode interfaces, and nanofluidics—and these functional groups are commonly present in membrane separation processes, we infer that the effect of functional groups on water cluster structure is universal.

In separation processes using polymeric membrane, spatial confinement effects and various functional groups that can serve as hydrogen bond donors/acceptors (e.g., hydroxyl, carboxyl, amide groups) coexist. Therefore, this phenomenon of water cluster dissociation does occur. Our results in Fig. 5b also demonstrate that even in the unmodified PIP-TMC membrane, this phenomenon exists. However, although several high-flux membrane materials have established a connection between water

permeance and the structural transformation of water clusters, for example:

i) -OH modified perforated graphene increases the cross-sectional area for water clusters transport and provides more water molecular orientations, reducing the entropic loss during transmembrane transport, thereby enhancing water flux[12];

ii) In carboxylated MXene 2D nanochannels, the carboxyl groups orient the water molecules, promoting orderly water transport along the channel direction and reducing ineffective collisions between water molecules and the channel walls[13];

iii) Nanofiltration membranes full of graphene capillaries, through extreme confinement, induce water molecules to form a highly compressed ice-like structure. The water molecule chains transfer momentum efficiently from one molecule to the next via cooperative rearrangement of hydrogen bonds, achieving ultrafast water transport with ultralow resistance[14];

iv) Moreover, as we mentioned in Fig. 1b, in CNTs, doped graphene, BN channels, MoS_2 channels, etc., a link has been established between the number of hydrogen bonds in water clusters and the water diffusion coefficient.

In the field of nanofiltration membrane research, particularly for conventional polyamide nanofiltration membranes, the structural transformation of water clusters and its relationship with water flux remain poorly understood. Our study demonstrates that the polyhydroxy quaternary ammonium salt at the pore entrance can indeed promote the dissociation of water clusters and reduce the energetic penalty of this process, thereby enhancing water flux. This finding provides an important complementary perspective for the mechanistic understanding of nanofluidic systems, especially membrane separation processes. We have further added supplementary remarks to discuss the generalizability of this mechanism.

MS-Line 617 Water clusters, as the fundamental units of transmembrane water transport, inherently influence the fluid characteristics within confined channels~~, thus significantly determining membrane permeability. Developing high-performance membrane materials based on the structural transformation mechanisms of water clusters within sub-nanometer pores represents a novel design framework.~~ Although previous studies have observed that the extreme confinement environment of dense polyamide membranes forces water clusters to reduce their hydration size to complete transport at the cost of energy consumption, and have established a fundamental understanding of the relationship between water cluster structure and water flux, how to precisely design membrane structures to regulate the confined transport structure of water clusters and thereby enhance flux remains an open question.

MS-Line 634 From the practical application viewpoint, this modified method has the potential for large-scale production due to the minor deviation from traditional interface polymerization. In conclusion, the results presented in our study provide novel insights into the mechanisms governing water permeance regarding the regulation of interfacial water cluster structures. More importantly, in other scenarios such as electrode interfaces process and nanofluidics, carboxyl and hydroxyl groups have also been observed to disrupt water cluster structures through hydration competition, oriented locking of water molecules, and local electric field construction, thereby affecting water-related interfacial processes. This indicates that the dissociation of water clusters by functional groups observed in our study is referenceable. The ~~the~~ ultrafast transport mechanisms of water molecules based on the dissociation of water clusters thus can be extended to other interfacial water-related applications, including energy technology, bio-medicine, water treatment and catalysis, etc.

1. Ding M, Szymczyk A, Goujon F, Soldera A, Ghoufi A. Structure and dynamics of water confined in a polyamide reverse-osmosis membrane: A molecular-simulation study. *Journal of Membrane Science* **458**, 236-244 (2014).

2. Li Wang JH, Mohammad Heiranian, Hanqing Fan, Lianfa Song, Ying Li, Menachem Elimelech. Water transport in reverse osmosis membranes is governed by pore flow, not a solution-diffusion mechanism. *Science advances* **9**, eadf8488 (2023).

3. Itoh Y, *et al.* Ultrafast water permeation through nanochannels with a densely fluorous interior surface. *Science* **376**, 738-743 (2022).

4. Tunuguntla RH, Henley RY, Yao Y-C, Pham TA, Wanunu M, Noy A. Enhanced water permeability and tunable ion selectivity in subnanometer carbon nanotube porins. *Science* **357**, 792-796 (2017).

5. Wang S, *et al.* Weak H-Bond Interface Environment for Stable Aqueous Zinc Batteries. *ACS Nano* **19**, 4484-4495 (2025).

6. Zhou L, *et al.* Suppressing Hydrogen Evolution in Aqueous Lithium-Ion Batteries with Double-Site Hydrogen Bonding. *ACS Energy Letters* **8**, 40-47 (2022).

7. Guo L, *et al.* Formate-Anion-Induced Water Network Reshaping Enables Concurrent Hydrogen and Magnesium Hydroxide Production From Seawater. *Angew Chem Int Ed Engl*, e6937994 (2026).

8. Ng LS, *et al.* Chaotropic Nanoelectrocatalysis: Chemically Disrupting Water Intermolecular Network at the Point-of-Catalysis to Boost Green Hydrogen Electrosynthesis. *Angew Chem Int Ed Engl* **63**, e202317751 (2024).

9. Gallina ME, Sassi P, Paolantoni M, Morresi A, Cataliotti RS. Vibrational analysis of molecular

interactions in aqueous glucose solutions. Temperature and concentration effects. *J Phys Chem B* **110**, 8856-8864 (2006).

10. Zhu Y, Chen Q, Hao J, Jiao Y. Breaking hydrogen-bonds in aqueous electrolytes towards highly reversible zinc-ion batteries. *Journal of Materials Chemistry A* **12**, 20097-20106 (2024).

11. Hanikel N, *et al.* Evolution of water structures in metal-organic frameworks for improved atmospheric water harvesting. *Science* **374**, 454-459 (2021).

12. Cohen-Tanugi D, Grossman JC. Water Desalination across Nanoporous Graphene. *Nano Letters* **12**, 3602-3608 (2012).

13. Zhang S, *et al.* Hydrogen-bonding-engineered 2D MXene nanochannels with reduced mass transfer resistance for high-permeability water purification. *Journal of Membrane Science* **730**, (2025).

14. Zhang Q, *et al.* Anomalous water molecular gating from atomic-scale graphene capillaries for precise and ultrafast molecular sieving. *Nat Commun* **14**, 6615 (2023).

Reviewer (Comment 7): *According to the mechanism proposed in this paper, water clusters dissociate when they approach the functional groups. However, given that the number of these functional group sites is finite, could the "active sites" become occupied, leading to the eventual failure of this effect?*

Our Response: Thank you for your valuable comments. First, it should be clarified that even in the absence of QBTS, the size of water clusters can still be reduced within a dense polyamide network due to extreme confinement—as demonstrated in studies by Wang et al[1]. Using MD simulations, they observed that water molecules are transported through dense polyamide nanochannels in the form of clusters, and the cluster size (coordination number) inside the nanochannels is significantly lower than that in the bulk phase, consistent with our observations for PIP-TMC (Fig. 5b). This process itself incurs a substantial energetic penalty [2].

Returning to the present study, as shown in Fig. 5g, when QBTS participates in the dissociation of water clusters, the Gibbs free energy becomes more negative, indicating that cluster dissociation occurs more readily. The results in Fig. 1c show that the functional groups have a stronger interaction with water clusters, which may provide two benefits:

i) the stronger interaction can compensate for the "structural destabilization" caused by water-cluster dissociation, thereby offsetting the energetic penalty associated with dissociation—similar to the "dehydration compensation" widely reported for partially dehydrated ions in biological channels [3, 4, 5], this effect directly lowers the energy barrier for transmembrane water transport. Indeed, we observed

this reduced energy barrier using TST (Fig. 5c). We have also provided a schematic diagram of this process (Fig. R2);

ii) this stronger interaction further promotes the dissociation of water clusters, generating more free water molecules (Fig. 5b) and expanding the effective water transport area (Fig. 2c). Consequently, we observed a higher water flux for the QBTS nanofiltration membrane.

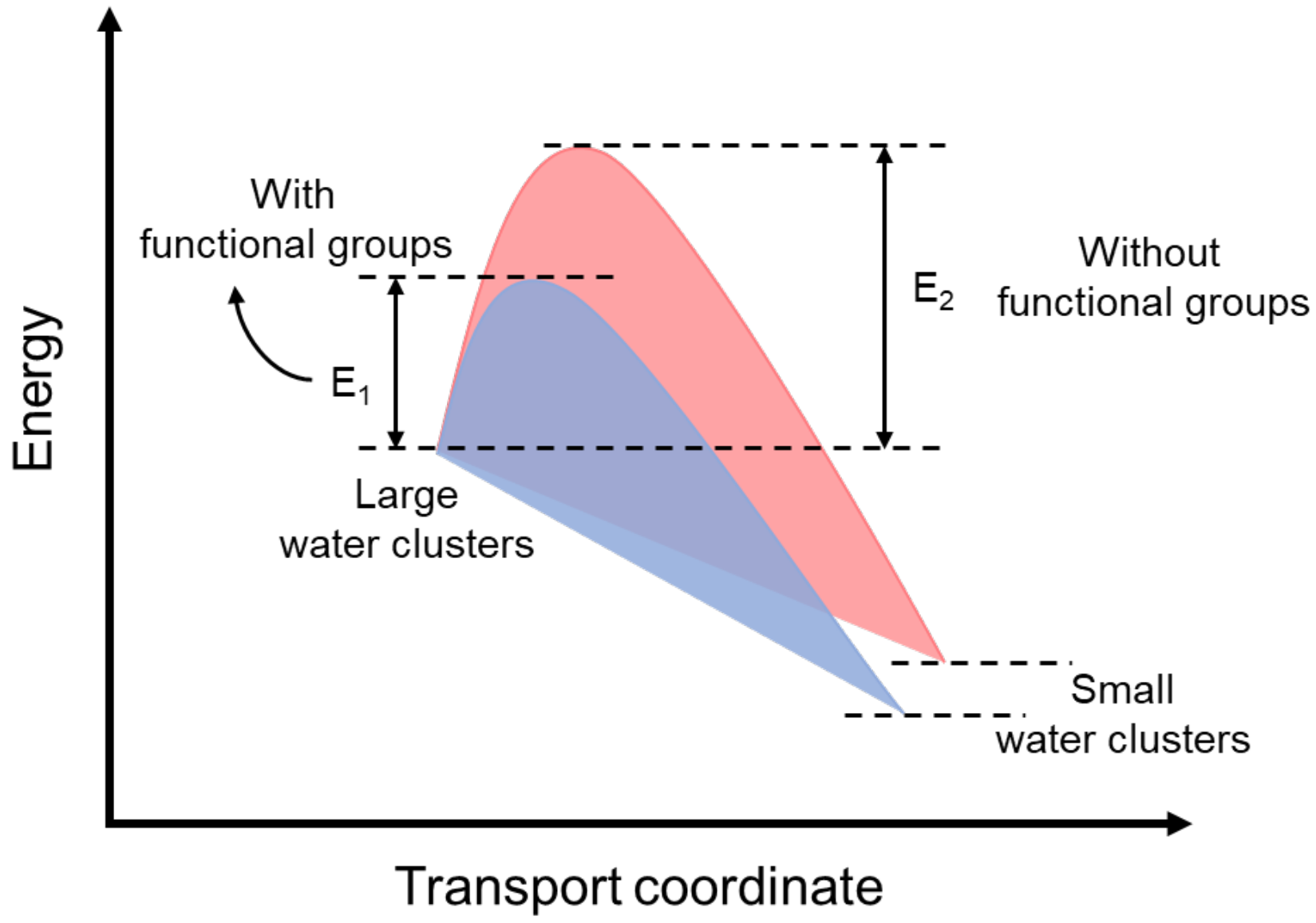

Fig R2. Schematic diagram of the energy changes during the dissociation process of water clusters with and without the involvement of functional groups. Functional groups compensate the energy required for this process through interactions with partially dissociated water clusters—acting as a catalyst—thereby making the dissociation of water clusters energetically favorable.

In summary, the interaction between QBTS and water clusters is fundamentally different from conventional adsorption processes. It acts more like a “catalyst” for water-cluster dissociation, both facilitating dissociation and generating more free water molecules. When this process occurs at the membrane-water interface, the pressure-driven convective flow refreshes the functional group-water interactions in the vicinity of QBTS, preventing “deactivation” due to occupation of active sites. In addition, as shown in Fig. 4i, long-term experiments demonstrate that even after 48 h, QBTS maintains both high water flux and good salt rejection.

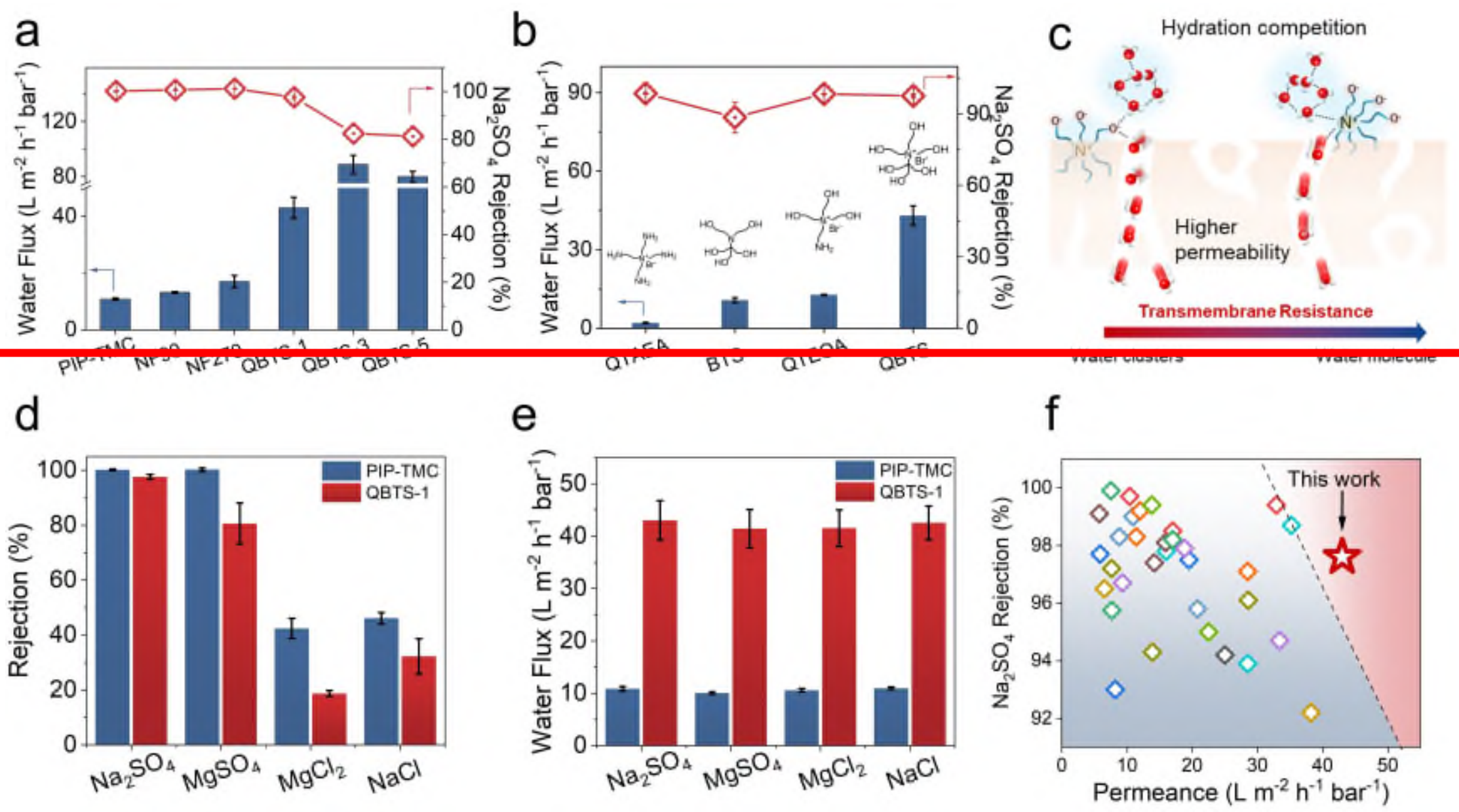

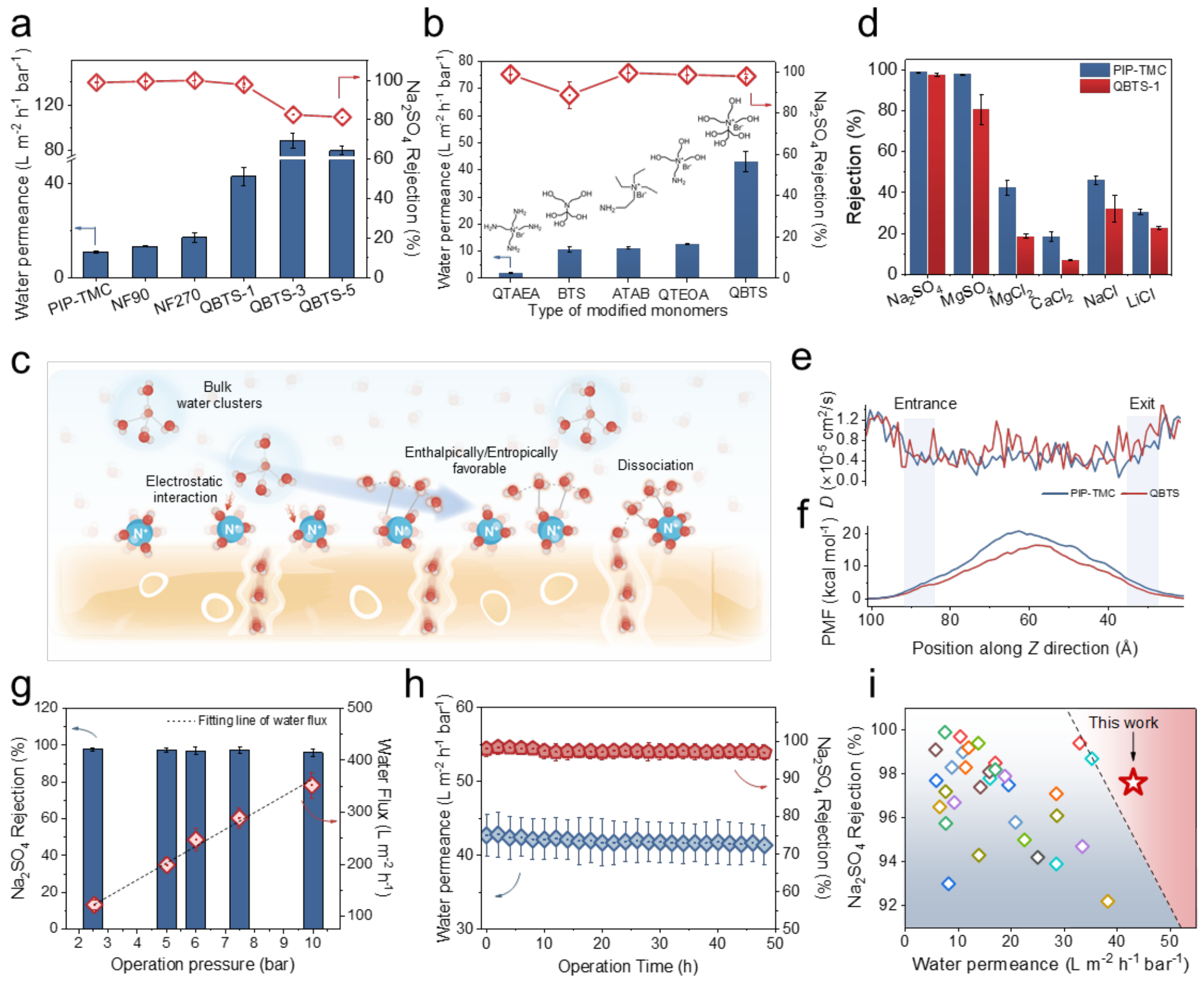

Fig. 4 Filtration performance of QBTS functionalized NF membranes. (a) Effect of QBTS monomer concentration on the separation performance of NF membranes (feed: 1000 ppm Na_2SO_4, 6 bar pressure). (b) Filtration performance of NF membranes modified with different ~~amine~~ monomers (feed: 1000 ppm Na_2SO_4, 6 bar pressure). (c) Schematic diagram of the suppositional mechanism of functional groups promoting the dissociation of water clusters. The dashed lines between water molecules indicate that the original water-water hydrogen bonds are weakened by the polyhydroxy structure of QBTS. (d) Salts rejection ~~and (e) water flux~~ of Na_2SO_4, $MgSO_4$, $MgCl_2$, $CaCl_2$, LiCl and NaCl by PIP-TMC and QBTS-1

membranes (Salt concentration, 1000 ppm; applied pressure, 6 bar; temperature, 25 °C). (e) The potential of mean force (PMF) and (f) diffusion coefficients (*D*) of Cl^- along the diffusion coordinate in PIP TMC and QBTS simulated nanochannels. The filtration performance of QBTS-1 under different (g) operation pressure and (h) operation Time (feed: 1000 ppm Na_2SO_4, 6 bar pressure). (f) (i) Performance comparison of QBTS-1 membrane with the state-of-the-art NF membranes using surface modification in reported literature (details in Table. S2).

MS-Line 463 Furthermore, it also demonstrated long-term operational stability (Fig. 4h), indicating that the promoting effect of QBTS on water transport does not fail after long-term operation. Overall, compared with other reported performances of advanced membranes using surface modification, the fabricated QBTS-functionalized NF membrane showed excellent water permeability while maintaining satisfactory salt rejection performance, which can be attributed to the dissociation effect of QBTS active sites on the water clusters.

References:

1. Li Wang JH, Mohammad Heiranian, Hanqing Fan, Lianfa Song, Ying Li, Menachem Elimelech. Water transport in reverse osmosis membranes is governed by pore flow, not a solution-diffusion mechanism. *Science advances* **9**, eadf8488 (2023).

2. Ding M, Szymczyk A, Goujon F, Soldera A, Ghoufi A. Structure and dynamics of water confined in a polyamide reverse-osmosis membrane: A molecular-simulation study. *Journal of Membrane Science* **458**, 236-244 (2014).

3. Wang M, *et al.* A coupled effect of dehydration and electrostatic interactions on selective ion transport through charged nanochannels. *Nanoscale* **10**, 18821-18828 (2018).

4. He Z, Zhou J, Lu X, Corry B. Bioinspired graphene nanopores with voltage-tunable ion selectivity for Na^+ and K^+. *ACS Nano* **7**, 10148-10157 (2013).

5. Zhang Y, Zhou K, Su S, Gao J, Liu J, Jiang L. Congener-welded crystalline carbon nitride membrane for robust and highly selective Li/Mg separation. *Science Advances* **10**, eadm9620 (2024).

<u>Reviewer (Comment 8)</u>: *In this study, the modified nanofiltration membrane exhibits improved hydrophilicity and zeta potential, both of which are known to contribute to enhanced water flux. How do the authors attribute the significant increase in flux primarily to the water cluster dissociation mechanism, rather than to these conventional and well-known effects of hydrophilicity and electrical properties?*

Our Response: We greatly appreciate the reviewer's insightful comment. Indeed, a wide range of studies on high-flux nanofiltration membranes have reported that membrane surface hydrophilicity/charge is crucial for enhancing water permeance, as they facilitate water partitioning into the membrane[1, 2, 3, 4, 5, 6]. However, after carefully analyzing the data and comparing it with other studies, we believe that hydrophilicity/zeta potential alone cannot fully explain the water flux enhancement observed in this study.

In our study, the water contact angle decreased from 65.0° for PIP-TMC to 44.5° for QBTS-1, the zeta potential (at pH 7) decreased from -21.69 to -34.04 mV, and the water flux increased from 10.87 to 43.01 LMH/bar (~4-fold). For comparison, Xiao et al. reported that an amino acid ionic liquid-modified biomimetic membrane exhibited a sharp decrease in water contact angle from 37.8° to 11.4°, with water flux increasing from 7.5 to 12.2 LMH bar^{-1} (a 63% increase)[3]. Mi et al. prepared a zwitterionic surface membrane with a contact angle decrease from 57.8° to 39.1°, and the flux increased from 30 to 77 LMH (~2.6-fold)[4].

Although direct comparison of water flux across different systems without considering factors other than contact angle, such as salt rejection and pore size, is not sufficiently rigorous, the literature comparison suggests that the reduction in contact angle observed in this study is insufficient to support such a large increase in water flux—especially considering that after QBTS modification, the membrane thickness increased from 143 nm to 179 nm, which is expected to increase additional water transport resistance. Furthermore, we observed that other polyhydroxy monomer-modified membranes, such as BTS and QTEOA, which theoretically also possess hydrophilic -OH groups, did not show a substantial increase in water flux in our experiments. This suggests that mechanisms beyond hydrophilicity contribute to the higher permeability.

More importantly, the enhancement of water flux by hydrophilicity/zeta potential is not independent of the structural transformation of water clusters at the membrane-water interface; on the contrary, the two are closely intertwined. For a membrane, the essence of hydrophilicity is precisely that the interaction between the membrane surface functional groups and water molecules is stronger than the water-water interactions within water clusters[7, 8]. Typical hydrophilic functional groups, such as -OH and -COOH, cause local distortion of water clusters through strong interactions[7], prolong the rotational relaxation time of water molecules[9], and orient water molecules in an ordered and restricted manner[8, 9]. These effects

collectively cause water molecules to spread along the membrane surface rather than aggregating into clusters. This finding is consistent with our observations in Figs. 2b and 2c, indicating that QBTS indeed is expected to facilitate the partitioning of water clusters into the membrane phase.

Furthermore, with respect to Zeta potential, an appropriate membrane surface charge can disrupt the hydrogen-bond network between water molecules through electrostatic interactions and reduce the entropic penalty, thereby lowering the energy barrier (PMF) for water molecules entering the nanochannels and enhancing water flux[2]. This principle is essentially similar to the mechanism of QBTS (Figs. 2b, 5b, and 5c). Therefore, these results link macroscopic properties, i.e., hydrophilicity/zeta potential with the phenomenon of water cluster dissociation induced by QBTS, providing deeper insight into the relationship between membrane surface physicochemical characteristics and water permeance. We have added corresponding remarks in the relevant sections of the manuscript to clarify the connection between hydrophilicity/zeta potential and the dissociation effect of QBTS.

MS-Line 317 In addition, the QBTS membrane exhibited a more hydrophilic surface (Fig. 3i) and a more negative zeta potential (Fig. 3j)~~,~~. According to previous studies, higher hydrophilicity may originate from the stronger interaction between the polyhydroxy structure of QBTS near the membrane pores and water molecules, which helps distort the local network of water clusters and spread along the membrane surface rather than aggregating into clusters. Meanwhile, the richer local negative charge near the membrane pores could help disrupt the H-bond network between water molecules through electrostatic interactions and reduce the entropic penalty.~~ which were~~ Therefore, QBTS on membrane surface was expected to promote the partition of water molecules from aqueous into the membrane thereby enhancing water flux [43].

References:

1. Xu X, *et al.* Nanofiltration membranes with ultra-high negative charge density for enhanced anion sieving and removal of organic micropollutants. *Nature Water* **3**, 704-713 (2025).

2. Xu X, *et al.* Water desalination through charged graphene nanoslits: Role of edge chirality and electrostatic effects. *Colloids and Surfaces A: Physicochemical and Engineering Aspects* **725**, (2025).

3. Xiao H-F, *et al.* Amphibian-inspired amino acid ionic liquid functionalized nanofiltration membranes with high water permeability and ion selectivity for pigment wastewater treatment. *Journal of Membrane Science* **586**, 44-52 (2019).

4. Mi Y-F, Zhao F-Y, Guo Y-S, Weng X-D, Ye C-C, An Q-F. Constructing zwitterionic surface of nanofiltration membrane for high flux and antifouling performance. *Journal of Membrane Science* **541**, 29-38 (2017).

5. Peng H, Zhao Q. A Nano-Heterogeneous Membrane for Efficient Separation of Lithium from High Magnesium/Lithium Ratio Brine. *Advanced Functional Materials* **31**, (2021).

6. Cao X-L, *et al.* High-permeability and anti-fouling nanofiltration membranes decorated by asymmetric organic phosphate. *Journal of Membrane Science* **617**, (2021).

7. Shin S, Willard AP. Water's Interfacial Hydrogen Bonding Structure Reveals the Effective Strength of Surface-Water Interactions. *J Phys Chem B* **122**, 6781-6789 (2018).

8. Li E, Du Z, Yuan S. Properties of a water layer on hydrophilic and hydrophobic self-assembled monolayer surfaces: A molecular dynamics study. *Science China Chemistry* **56**, 773-781 (2013).

9. Zhang H, Sundaresan S, Webb MA. Molecular Dynamics Investigation of Nanoscale Hydrophobicity of Polymer Surfaces: What Makes Water Wet? *J Phys Chem B* **127**, 5115-5127 (2023).

Reviewer (Comment 9): *In the ToF-SIMS tests, the authors used a 30 keV Bi_3^+ beam to bombard the solution. Could this energy level cause intrinsic dissociation of the water clusters, thereby affecting the accuracy of the method? Additionally, the authors should provide more details regarding the ToF-SIMS testing procedure to explain how the hydration distributions were accurately obtained.*

Our Response: Thanks for the valuable comments. Firstly, we would like to explain in a bit more detail how the *in situ* liquid ToF-SIMS works [1, 2]. In the ToF-SIMS characterization utilizing the microfluidic device (Fig. S20), a pulsed Bi_3^+ primary ion beam was consistently directed toward the SiN_x detective window. The SiN_x film was punched through approximately at ~440 s (Fig. S27), as evidenced by a significant decrease in the signal intensity of Si_2N^+, coinciding with a simultaneous increase in the signals of $(H_2O)H^+$ from the feed solution. Subsequently, the hydrates from the Na_2SO_4 solution were exposed to the Bi_3^+ primary ion beam, promptly ionizing them into secondary ions. The secondary ions will fly through the time-of-fly tube to be detected in an extremely short time (duration ~10-200 μs), forming the instantaneous mass spectrum of interested hydrates based on their mass-to-charge ratio (m/z) (Fig. S28). Finally, we normalized the mass spectrum peak intensities of individual hydrate [$I_{(\mathrm{H_2O})_n\mathrm{H}^+}$] to the total mass spectrum intensities of all hydrates [$\sum I_{(\mathrm{H_2O})_n\mathrm{H}^+}$] to obtain the ion hydration distribution ($h_{\mathrm{H_2O}}$).

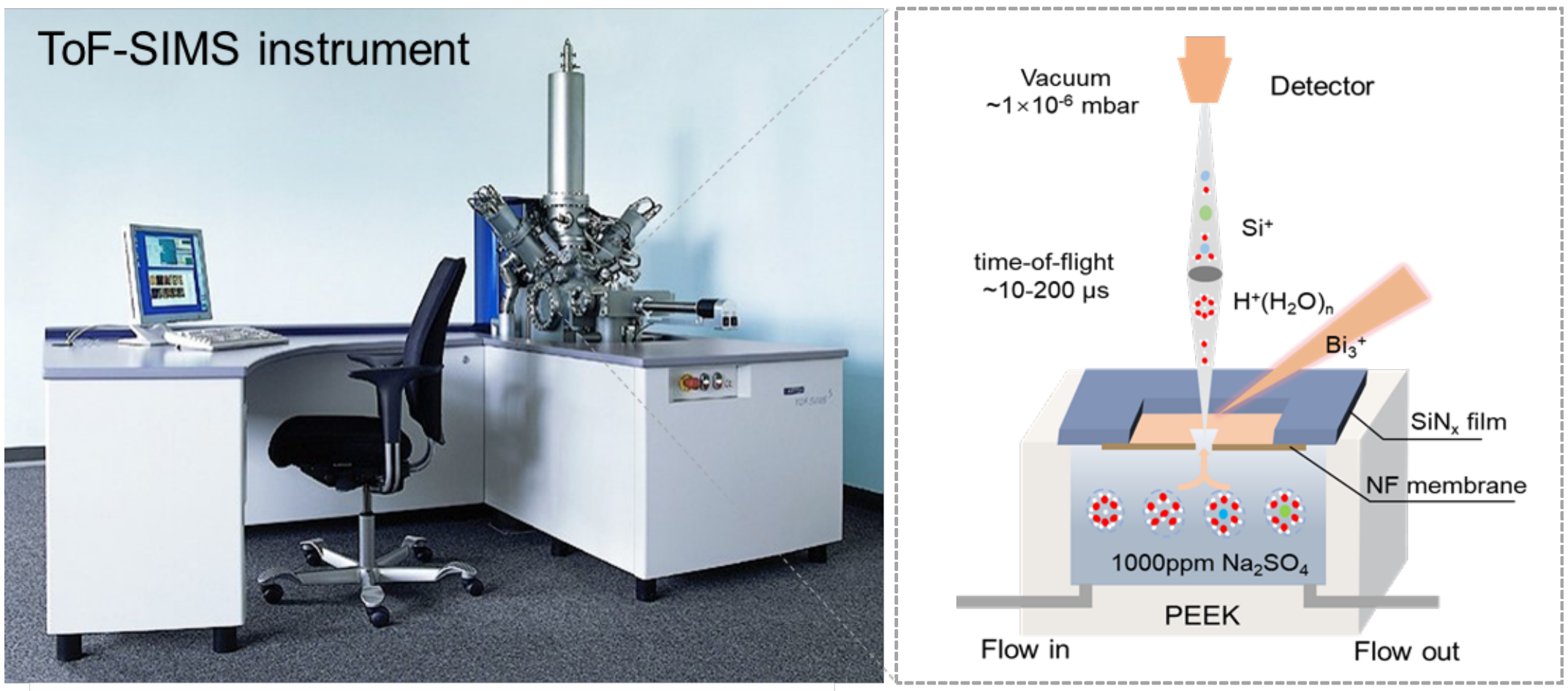

Fig. S20 Schematic diagram of liquid ToF-SIMS. Left: ToF-SIMS instrument; Right: microfluidic filtration device placed in the ToF-SIMS vacuum chamber.

In our previous study, we demonstrated that the *in situ* liquid ToF-SIMS is a surprisingly soft ionization process for investigation of ion-water and water-water interactions[2, 3, 4, 5]. During the determination, hydrates fragmentation in liquid SIMS analysis could be alleviated due to the continuously updated liquid surface around the punched pore via diffusion as well as the flexible liquid environments, which is favored for the formation of intact hydrates such as water clusters. In addition, once the SiN_x film was perforated, the pulse width was immediately reduced to 100 ns, which reduced the intensity of the beam current from 0.27 pA to 0.15 pA. This can mitigate the impact on the hydrated ions from ion irradiation. Furthermore, the ionized hydrated ions can go through the time-of-flight tube and be detected in an extremely short time (duration ~10-200 μs)[4]—greatly minimizing the possibility of collisions and additional evaporation. Overall, the *in situ* liquid ToF-SIMS can maintain the relatively intact hydrated structure of water clusters, which is thus suitable for detecting solvation structures and weak water-water interactions. Corresponding statements about the determination details have therefore been added to our manuscript.

MS-Line 472 The *operando* characterization of the dissociation of water clusters, i.e., $(H_2O)_nH^+$ as the indicators, during transmembrane transport was conducted on an *in situ* liquid ToF-SIMS microfluidic filtration platform with pristine and functionalized NF membranes (Fig. 5a). Previous studies have demonstrated that *in situ* liquid ToF-SIMS is applicable for detecting solvation structures and weak water-water interactions within the solution phase and even during hydrates transport through the polymeric membrane (details in Supplementary information).

MS-Line 712 Notably, the hydrates were ionized directly upon exiting the nanochannels and detected within ~~~100 μs~~ ~10-200 μs via time-of-flight analysis, effectively preserving their pristine hydration structures during transmembrane transport.

SI-Line 87 Thus, the $(H_2O)_nH^+$ in feed solution were consequently exposed to the Bi_3^+ primary ion beam and were ~~immediately~~ ionized to secondary ions, forming the instantaneous mass spectrum of interested $(H_2O)_nH^+$. It should be particularly noted that the water clusters from permeate will be ionized immediately once they exit the nanochannel. In other words, the water clusters were ionized immediately in the aqueous phase rather than in the gas phase, which helps to ensure that the detected SIMS signals maintain the hydration properties of ions from permeate. The dynamic ToF-SIMS depth profiling also indicated that the signals of representative species from permeate were immediately increased once the ions reached the detective area. On the other hand, the *in situ* liquid ToF-SIMS has been demonstrated to be a surprisingly soft technique for investigation of water-water interactions. During the determination, hydrates fragmentation in liquid SIMS analysis could be alleviated due to the continuously updated liquid surface around the punched pore via diffusion as well as the flexible liquid environments, which is favored for the formation of intact water clusters. In addition, once the SiN_x film was perforated, the pulse width was immediately reduced to 100 ns, which reduces the intensity of the beam current from 0.27 pA to 0.15 pA. This can mitigate the impact on the hydrated ions from ion irradiation. Furthermore, the ionized hydrates can go through the time-of-flight tube and be detected in an extremely short time (duration ~10-200 μs)—greatly minimizing the possibility of collisions and additional evaporation. As such, the original structure of the water clusters is well maintained after water clusters leave the nanochannel, which can represent the operando changes in the ion hydration structure during transport through nanochannels.

References:

1. Lu C, *et al.* In Situ Characterization of Dehydration during Ion Transport in Polymeric Nanochannels. *J Am Chem Soc* **143**, 14242-14252 (2021).

2. Liu YY, Zhang SZ, Ying YL, Xia HL, Hua X, Long YT. Ion-Specific Effects on Hydrogen Bond Network at a Submicropore Confined Liquid-Vacuum Interface: An in Situ Liquid ToF-SIMS Study. *J Phys Chem Lett* **10**, 4935-4941 (2019).

3. Liu Y-Y, Ying Y-L, Hua X, Long Y-T. In-situ discrimination of the water cluster size distribution in aqueous solution by ToF-SIMS. *Science China Chemistry* **61**, 159-163 (2017).

4. Zhang Y, *et al.* In situ liquid secondary ion mass spectrometry: A surprisingly soft ionization process for investigation of halide ion hydration. *Anal Chem* **91**, 7039-7046 (2019).

5. Lu C, *et al.* Response of Ionic Hydration Structure and Selective Transport Behavior to Aqueous Solution Chemistry during Nanofiltration. *Environ Sci Technol* **58**, 11791-11801 (2024).

Reviewer (Comment 10): *Furthermore, I noted that during the ToF-SIMS measurements, the opposite side of the membrane was under vacuum. Could this condition potentially introduce discrepancies between the tested results and the actual operating system?*

Our Response: We thank the reviewers for their comments. We would like to explain in a bit more detail how the *in situ* liquid ToF-SIMS works. As shown in Fig. S20, in the ToF-SIMS characterization based on the microfluidic filtration device, aqueous solution enters the nanochannels of the membrane and transports to the other side driven by the transmembrane pressure provided by the vacuum applied in the permeate side. When the aqueous solution with water clusters exits the membrane nanochannels, it is exposed to the Bi_3^+ that is continuously applied to permeate side of the membrane[1, 2, 3, 4]. Therefore, the water clusters from permeate will be ionized immediately once they exit the nanochannel. In other words, the water clusters were ionized immediately in the aqueous phase rather than in the gas phase, which helps to ensure that the detected SIMS signals maintain the hydration properties of ions from permeate. The dynamic ToF-SIMS depth profiling also indicated that the signals of representative species from permeate were immediately increased once the ions reached the detective area (Fig. S20).

On the other hand, the *in situ* liquid ToF-SIMS has been demonstrated to be a surprisingly soft technique[4]. Hence, it is suitable for detecting solvation structures and weak ion-water interactions during in situ analysis [2, 4]. In addition, once the hydrates are ionized to secondary ions and leave the aqueous phase, they require only a short time (~10-200 μs) to reach the detector[4]—greatly minimizing the possibility of collisions and additional evaporation. As such, the original structure of the water clusters is well maintained after water clusters leave the nanochannel, which can represent the operando changes in the ion hydration structure during transport through nanochannels.

In addition, the hydration number measured by ToF-SIMS was in good agreement with the strongly-bound water molecules of the hydrates obtained by theoretical extractions based on bulk properties[5, 6, 7, 8], which has more important implications regarding ion transport behaviour. Overall, the hydration numbers determined by ToF-SIMS give a reasonable indication of the variation in hydration numbers when water clusters are transported between different membranes.

Collectively, we agree with the reviewer that the conditions on the measurement side of the membrane are very important. The discrepancy between the experimental environment and the actual physicochemical environment of the ion might cause concern that the measured hydration number deviates from the "real" hydration number. However, as we mentioned before, the hydrates were ionized in the liquid phase rather than in the gas phase, and the duration time of ionized secondary ions in the vacuum was quite short, which allows the detected hydrated

ions to retain its properties of the aqueous phase. In addition, the hydration number measured by ToF-SIMS was in good agreement with the hydration number obtained from theoretical extractions based on bulk properties. Previous studies have also demonstrated that *in situ* liquid ToF-SIMS is applicable for detecting solvation structures and weak water-water interactions within the solution phase[2, 4, 9] and even during hydrates transport through the polymeric membrane[1, 3]. Lastly, we adjusted the parameters of the ToF-SIMS instrument to obtain consistent operating conditions as much as possible before performing each experiment so that the variables of the experiment were mainly from different membranes. This ensures that the observed relative changes in hydration distribution induced by different membranes were convincing. We have also added additional explanation in the manuscript to clarify the feasibility of the liquid TOF-SIMS measurement.

MS-Line 472 The *operando* characterization of the dissociation of water clusters, i.e., $(H_2O)_nH^+$ as the indicators, during transmembrane transport was conducted on an *in situ* liquid ToF-SIMS microfluidic filtration platform with pristine and functionalized NF membranes (Fig. 5a). Previous studies have demonstrated that *in situ* liquid ToF-SIMS is applicable for detecting solvation structures and weak water-water interactions within the solution phase and even during hydrates transport through the polymeric membrane (details in Supplementary information).

SI-Line 87 Thus, the $(H_2O)_nH^+$ in feed solution were consequently exposed to the Bi_3^+ primary ion beam and were ~~immediately~~ ionized to secondary ions, forming the instantaneous mass spectrum of interested $(H_2O)_nH^+$. It should be particularly noted that the water clusters from permeate will be ionized immediately once they exit the nanochannel. In other words, the water clusters were ionized immediately in the aqueous phase rather than in the gas phase, which helps to ensure that the detected SIMS signals maintain the hydration properties of ions from permeate. The dynamic ToF-SIMS depth profiling also indicated that the signals of representative species from permeate were immediately increased once the ions reached the detective area. On the other hand, the *in situ* liquid ToF-SIMS has been demonstrated to be a surprisingly soft technique for investigation of water-water interactions. During the determination, hydrates fragmentation in liquid SIMS analysis could be alleviated due to the continuously updated liquid surface around the punched pore via diffusion as well as the flexible liquid environments, which is favored for the formation of intact water clusters. In addition, once the SiN_x film was perforated, the pulse width was immediately reduced to 100 ns, which reduces the intensity of the beam current from 0.27 pA to 0.15 pA. This can mitigate the impact on the hydrated ions from ion irradiation. Furthermore, the ionized hydrates can go through the time-of-flight tube and be detected in an extremely short time (duration ~10-200 μs)—greatly minimizing the possibility of collisions and additional evaporation. As such, the original structure of the water clusters is well maintained after water clusters leave the nanochannel, which can represent the operando changes in the ion hydration structure during transport through nanochannels.

SI-Line 118 The hydration number measured by ToF-SIMS was in good agreement with the strongly-bound water molecules of the hydrates obtained by theoretical extractions based on bulk properties, which has more important implications regarding ion transport behaviour. Overall, the hydration numbers determined by ToF-SIMS give a reasonable indication of the variation in hydration numbers when water clusters are transported between different membranes.

References:

1. Lu C, *et al.* In Situ Characterization of Dehydration during Ion Transport in Polymeric Nanochannels. *J Am Chem Soc* **143**, 14242-14252 (2021).

2. Liu YY, Zhang SZ, Ying YL, Xia HL, Hua X, Long YT. Ion-Specific Effects on Hydrogen Bond Network at a Submicropore Confined Liquid-Vacuum Interface: An in Situ Liquid ToF-SIMS Study. *J Phys Chem Lett* **10**, 4935-4941 (2019).

3. Lu C, *et al.* Dehydration-enhanced ion-pore interactions dominate anion transport and selectivity in nanochannels. **9**, eadf8412 (2023).

4. Zhang Y, *et al.* In situ liquid secondary ion mass spectrometry: A surprisingly soft ionization process for investigation of halide ion hydration. *Anal Chem* **91**, 7039-7046 (2019).

5. Marcus Y. *Ions in solution and their solvation*. John Wiley & Sons, Inc. (2015).

6. Marcus Y. Electrostriction, ion solvation, and solvent release on ion pairing. *J Phys Chem B* **109**, 18541-18549 (2005).

7. Bockris JOM, Saluja PPS. Ionic Solvation Numbers from Compressibilities and Ionic Vibration Potentials Measurements. *The Journal of Physical Chemistry* **76**, 2140-2151 (1972).

8. Marcus Y. The Standard Partial Molar Volumes of Ions in Solution. Part 4. Ionic Volumes in Water at 0-100 °C. *J Phys Chem B* **113**, 10285-10291 (2009).

9. Liu Y-Y, Ying Y-L, Hua X, Long Y-T. In-situ discrimination of the water cluster size distribution in aqueous solution by ToF-SIMS. *Science China Chemistry* **61**, 159-163 (2017).

Reviewer #2:

Reviewer: *Focusing on the core demand of improving the water permeance of nanofiltration (NF) membranes, this study proposed an innovative strategy based on polyhydroxy quaternized interfaces to dissociate water clusters. By combining experimental methods with molecular simulations, it reveals the mechanism by which the hydration competition of functional groups regulates the structure of water clusters and thereby optimizes water transport. The developed QBTS-functionalized NF membrane exhibits outstanding performance in balancing water flux and salt rejection. There are aspects requiring improvement in terms of detailed expression, mechanism integrity, and consistency between experiments and simulations, which need further revisions to enhance the persuasiveness of the study.*

Our Response: Thank you for the thorough analysis of our work. We believe this work will be of great interest to the broad field of nanofluidics. We would like to kindly remind that in the point-by-point response, we have marked newly added text in blue, deleted text in red with a strikethrough, and placed all revision marks within grey text boxes. In the text, MS-Line refers to the line numbers in the Marked Manuscript, and SI-Line refers to the line numbers in the Marked Supplementary Information.

Reviewer (Comment 1): *The abscissa of Figure 1b is labeled "Number of H-Bonds for water clusters", but it is not clear whether it refers to "the number of hydrogen bonds formed by a single water molecule in a water cluster" or "the total number of hydrogen bonds in a single water cluster".*

Our Response: We thank the reviewer for the suggestion. To be precise, the data shown in Fig. 1b represent the average number of hydrogen bonds per water molecule in water clusters for the different nanochannels, which typically reflects the stability of the H-bond network of water clusters inside the nanochannels. Accordingly, we have revised the abscissa of Fig. 1b and the corresponding text in the manuscript.

MS-Line 125 As shown in Fig. 1b, we have summarized the relationship between the ~~average number of H-bonds in water clusters~~ average number of H-bonds per water molecule in water clusters and their diffusion coefficients within different nanochannels, e.g., CNTPs (details in supplementary information, Table. S1).

MS-Line 159 (b) The relationship between the ~~number of H-bonds and the diffusion rate of water molecules in different nanochannels~~ average number of H-bonds per water molecule in water clusters and their diffusion coefficients within different nanochannels (details in *supplementary information*).

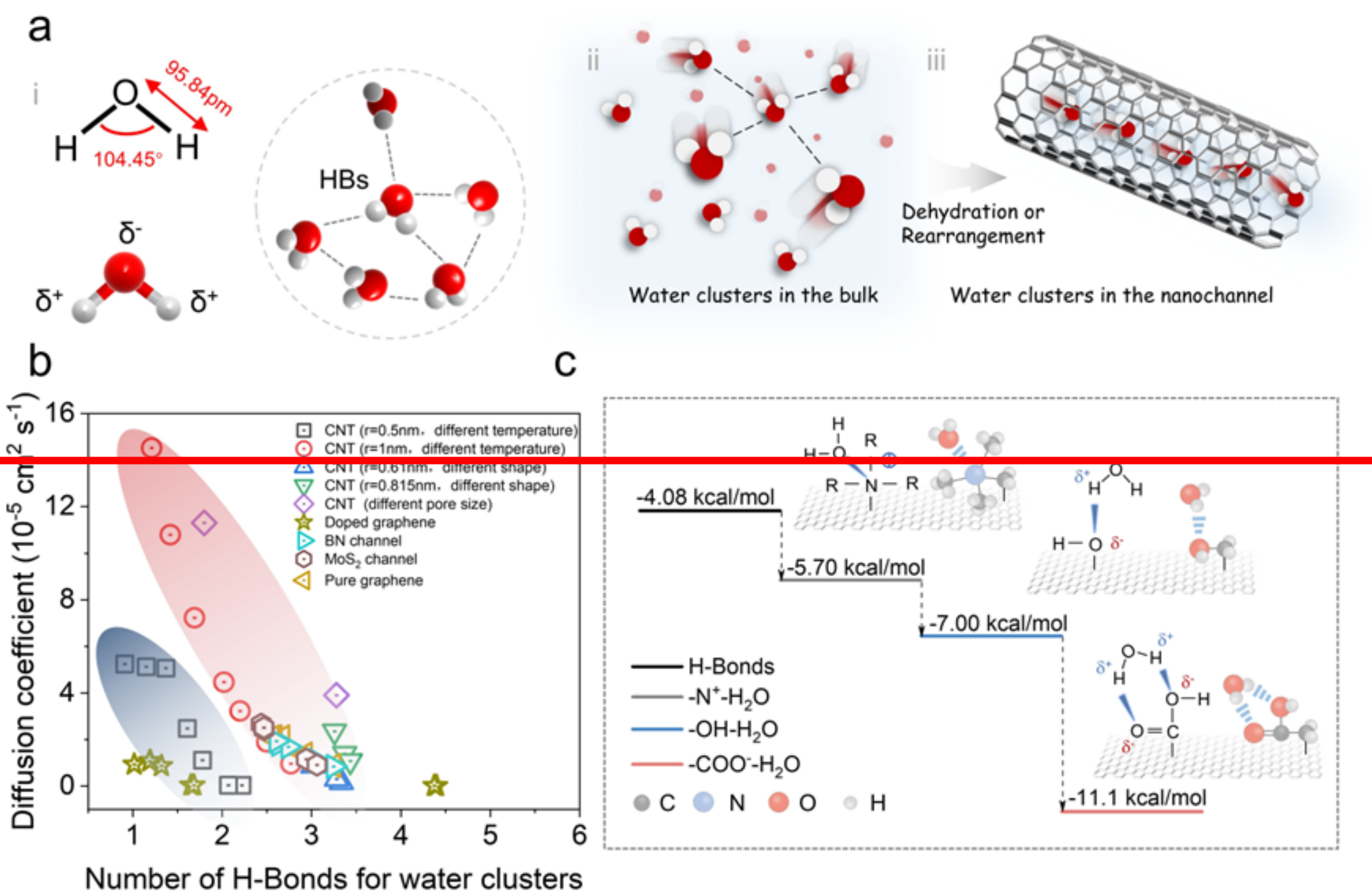
a
i
95.84pm
104.45°
H
O
H
δ-
δ+
δ+
HBs
ii
iii
Dehydration or Rearrangement
Water clusters in the bulk
Water clusters in the nanochannel
b
c
Diffusion coefficient (10^-5 cm^2 s^-1)
Number of H-Bonds for water clusters
CNT (r=0.5nm, different temperature)
CNT (r=1nm, different temperature)
CNT (r=0.61nm, different shape)
CNT (r=0.815nm, different shape)
CNT (different pore size)
Doped graphene
BN channel
MoS2 channel
Pure graphene
-4.08 kcal/mol
-5.70 kcal/mol
-7.00 kcal/mol
-11.1 kcal/mol
H-Bonds
-N+-H2O
-OH-H2O
-COO−-H2O
C
N
O
H

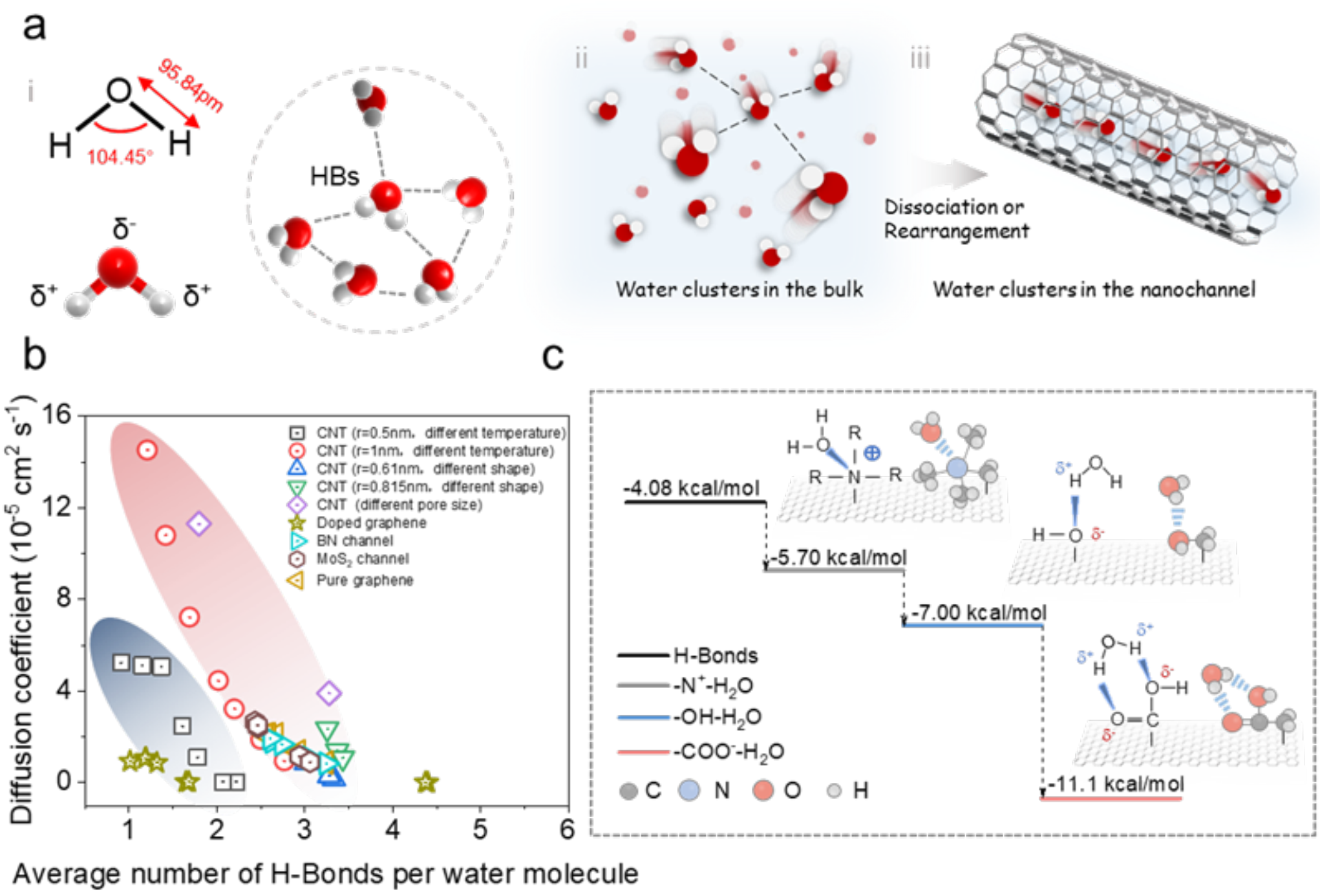
a
i
95.84pm
104.45°
H
O
H
δ-
δ+
δ+
HBs
ii
iii
Dissociation or Rearrangement
Water clusters in the bulk
Water clusters in the nanochannel
b
c
Diffusion coefficient (10^-5 cm^2 s^-1)
Average number of H-Bonds per water molecule
CNT (r=0.5nm, different temperature)
CNT (r=1nm, different temperature)
CNT (r=0.61nm, different shape)
CNT (r=0.815nm, different shape)
CNT (different pore size)
Doped graphene
BN channel
MoS2 channel
Pure graphene
-4.08 kcal/mol
-5.70 kcal/mol
-7.00 kcal/mol
-11.1 kcal/mol
H-Bonds
-N+-H2O
-OH-H2O
-COO−-H2O
C
N
O
H

<u>Reviewer (Comment 2)</u>: *The functional group -R_3N^+ is repeatedly mentioned in Figure 2 and the main text, but the specific structure of R is not clearly defined.*

Our Response: We thank the reviewer for the careful suggestion. In this study, we investigated the effect of the quaternary ammonium N^+ cation on the structure of water clusters. In the first part of the manuscript (Lines 116-208), -R_3N^+ was used for DFT calculation, where R represents a methyl group (-CH_3). For the QBTS monomer, however, the actual structure of "-R_3N^+" is $(CH_2CH_2OH)_3(C(CH_2OH)_3)$ N^+. To avoid confusion, we have specified that for QBTS, the quaternary-N^+ was used to represent the $(CH_2CH_2OH)_3(C(CH_2OH)_3)$ N^+, whereas the -R_3N^+ represents the -$(CH_3)_3N^+$. The relevant text in the manuscript has been revised as follows:

MS-Line 26 The results, observed by *in situ* liquid time-of-flight secondary ion mass spectrometry in combination with molecular dynamics simulation, reveal that the functional groups (-OH and ~~-R_3N^+~~ quaternary-N^+) of membrane pore entrances can dissociate water clusters

MS-Line 99 we confirmed that ~~-R_3N^+ (R represents Methyl)~~ quaternary-N^+, -OH, and -COO^-, which act as hydrogen bond acceptors/donors, can dissociate water clusters via the strong interactions between functional group and water molecule.

MS-Line 105 we found that the QBTS active sites, i.e., -OH and ~~-R_3N^+~~ quaternary-N^+ dissociated the water clusters with larger sizes.

MS-Line 167 To verify this, we selected three typical functional groups modified onto membranes in NF processes—carboxyl (-COO^-), hydroxyl (-OH), and quaternary ammonium (-R_3N^+, R represents Methyl) to explore their interactions with water molecules (Fig. 1c and Fig. S1) using DFT calculations.

MS-Line 235 In contrast, we propose that the polyhydroxy quaternary ammonium structure could further facilitate the dissociation of water clusters—the positively charged ~~-R_3N^+~~ quaternary-N^+ center attracts water clusters to the active sites via electrostatic adsorption, and the polyhydroxy network with rich hydrogen-bond donating sites, can more effectively promote cluster dissociation.

MS-Line 263 A new XPS peak at 400.8 eV in N 1s core level spectra is seen from QBTS-1 (Fig. 3c and Fig. S3), which is assigned to ~~-R_3N^+~~ quaternary-N^+ due to the incorporation of QBTS[35], confirming the successful modification of PIP-TMC by the QBTS monomers. Additionally, FTIR analysis of the membrane modified with QBTS monomers reveals new absorption peaks at 984 cm^{-1} and 1676 cm^{-1}, corresponding to ~~-R_3N^+~~ quaternary-N^+ and ester groups[36], further validating the successful modification of PIP-TMC by QBTS monomers (Fig. S4).

MS-Line 371 Based on the binding energy data in Fig. 1c, we hypothesize that there may be a synergistic effect between the -OH and ~~R_3N^+~~ quaternary-N^+ on the dissociation of water clusters to facilitate the transport of water molecules. Specifically, as shown in Fig. 4c, during the partitioning process where water clusters from the aqueous phase go into the membrane phase driven by pressure, larger water clusters approach the active sites through the electrostatic adsorption by ~~R_3N^+~~ quaternary-N^+ in QBTS and undergo dissociation due to the hydration competition of ~~R_3N^+~~ quaternary-N^+ and -OH with H-bond donor/acceptor.

MS-Line 423 Simultaneously, we observed that QBTS-1 slightly reduced the retention of the four salts, particularly for $MgCl_2$, which is reasonably attributed to that -OH and ~~R_3N^+~~ quaternary-N^+ on the membrane surface breaking the hydrogen bonds within the water molecule clusters while assisting in the dehydration of hydrated ions and facilitating the transport of ions.

MS-Line 516 Therefore, we can deduce that the hydration competition at the active sites of QBTS, i.e., -OH and ~~R_3N^+~~ quaternary-N^+ might further promote the water clusters dissociation, facilitating more favorable transmembrane transport of water molecules.

MS-Line 565 In contrast, the involvement of functional groups ($-COO^-$, -OH, and ~~R_3N^+~~ quaternary-N^+) in the dehydration process (details in Fig. S13) led to a negative ΔG (Fig. 5d), indicating that the hydration competition is favorable for water clusters to spontaneous dissociation to transform as smaller water species.

MS-Line 627 In this study, using *in situ* liquid ToF-SIMS in combination with MD simulations, we demonstrated that the NF membrane with polyhydroxy quaternized interface can dissociate water clusters through the hydration competition effect of -OH and ~~R_3N^+~~ quaternary-N^+, thereby reducing water transport resistance and further enhancing membrane flux.

Reviewer (Comment 3): *In lines 163-164, the "number of dangling bonds (DBs)" is mentioned , but the specific meaning of "dangling bonds" (as well as the statistical method) is not explained.*

Our Response: We thank the reviewer's valuable comments. In our study, any H atom that was not H-bonded to other water molecules was defined as a dangling band (DB) [1, 2, 3, 4]. Therefore, the DB represents a free water molecule that has "escaped" from the water cluster; the more DBs there are, the less favorable the formation of water clusters [2, 5, 6].

In this work, H-bonds were determined according to geometric criteria: an O-H···O bond was defined as a H-bond only when the distance between the donor O-H and the acceptor O was less than 2.5 Å and the angle was greater than 90°. O-H···O bonds that did not meet these criteria were classified as DBs. It is worth noting that this criterion is broader compared with those reported in other literature[7, 8, 9, 10]; accordingly, the criterion for DBs is relatively stricter, and the resulting DB statistics in our study can more accurately reflect the influence of functional groups on the structure of water clusters.

Based on this, in the MD simulations, our calculated DBs are defined as a water molecule (*mol1*) within a specified distance from the pore center (i.e., radial position) that does not form an H-bond with surrounding water molecules (*mol2*). For an H atom in mol1, if it does not satisfy the H-bonding criteria with any of the surrounding water molecules, that H atom is counted as having one DB. The final DB value is calculated as the total number of DBs divided by the total number of water molecules, which corresponds to the metric of "Number of dangling bonds per H_2O" reported in Fig. 2b. We have also added a supplemental statement to illustrate the definition of DBs in the manuscript.

MS-Line 179 As illustrated in Fig 2a, a series of inert graphene pores functionalized at the edges with -H, -OH, $-COO^-$, and $-R_3N^+$ were constructed to investigate the influence of functional groups on water molecule transport. We first evaluated the radial distribution of the number of dangling bonds (DBs) around different functional groups in the pore rim of perforated graphene. In our study, any H atom that was not hydrogen bonded to other water molecules was defined as a dangling band. Therefore, the DB represents a free water molecule that has "escaped" from the water cluster; the more DBs there are, the less favorable the formation of water clusters. In the control case of the H-functionalized pore, the fluctuation in ~~dangling bonds (~~DBs~~)~~ of the water cluster along the radial direction was minimal, indicating limited structural perturbation of water clusters by the inert pore edges

SI-Line 331 In this work, H-bonds were determined according to geometric criteria: an O-H···O bond was defined as a H-bond only when the distance between the donor O-H and the acceptor O was less than 2.5 Å and the angle was greater than 90° . O-H···O bonds that did not meet these criteria were classified as dangling bonds (DBs). It is worth noting that this criterion is broader compared with those reported in other literature; accordingly, the criterion for DBs is relatively stricter, and the resulting DB statistics in our study can more accurately reflect the influence of functional groups on the structure of water clusters. Based on this, in the MD simulations, our calculated DBs are defined as a water molecule (*mol1*) within a specified distance from the pore center (i.e., radial position) that does not form an H-bond with surrounding water molecules (*mol2*). For an H atom in *mol1*, if it does not satisfy the H-bonding criteria with any of the surrounding water molecules, that H atom is counted as having one DB. The final DB value is calculated as the total number of DBs divided by the total number of water molecules, which corresponds to the metric of "Number of dangling bonds per H_2O" reported in Fig. 2.

References:

1. Robalo JR, Streacker LM, Mendes de Oliveira D, Imhof P, Ben-Amotz D, Verde AV. Hydrophobic but Water-Friendly: Favorable Water–Perfluoromethyl Interactions Promote Hydration Shell Defects. *Journal of the American Chemical Society* **141**, 15856-15868 (2019).

2. Itoh Y, *et al.* Ultrafast water permeation through nanochannels with a densely fluorous interior surface. *Science* **376**, 738-743 (2022).

3. Perera PN, Fega KR, Lawrence C, Sundstrom EJ, Tomlinson-Phillips J, Ben-Amotz D. Observation of water dangling OH bonds around dissolved nonpolar groups. *Proceedings of the National Academy of Sciences* **106**, 12230-12234 (2009).

4. Roy S, Biswas B, Ghosh N, Singh PC, Mondal JA. Hydrophobic Hydration of Fluoroalkyl (C–F) is Distinctly Different from That of Its Hydrogenated Counterpart (C–H), as Observed by Raman Difference with Simultaneous Curve Fitting Analysis. *The Journal of Physical Chemistry C* **123**, 27012-27019 (2019).

5. Miyazaki M, Fujii A, Ebata T, Mikami N. Infrared Spectroscopic Evidence for Protonated Water Clusters Forming Nanoscale Cages. *Science* **304**, 1134-1137 (2004).

6. Servage KA, Silveira JA, Fort KL, Russell DH. Evolution of Hydrogen-Bond Networks in Protonated Water Clusters H+(H2O)n (n = 1 to 120) Studied by Cryogenic Ion Mobility-Mass Spectrometry. *The Journal of Physical Chemistry Letters* **5**, 1825-1830 (2014).

7. Ishiyama T, Kitanaka K. Asymmetric Hydrogen-Bonding Structure at a Water/Ice Interface. *The Journal of Physical Chemistry C* **124**, 23287-23294 (2020).

8. Otsuki Y, Sugimoto T, Ishiyama T, Morita A, Watanabe K, Matsumoto Y. Unveiling subsurface hydrogen-bond structure of hexagonal water ice. *Physical Review B* **96**, 115405 (2017).

9. de Dios AC. 17O Chemical Shifts in Water. *The Journal of Physical Chemistry A* **129**, 11203-11212 (2025).

10. Ozkanlar A, Zhou T, Clark AE. Towards a unified description of the hydrogen bond network of liquid water: A dynamics based approach. *The Journal of Chemical Physics* **141**, 214107 (2014).

Reviewer (Comment 4): *QBTS-1, QBTS-3, and QBTS-5 are not clearly defined when first mentioned or in the experimental methods section. It is suggested to add the explanation that "QBTS-1, QBTS-3, and QBTS-5 respectively refer to NF membranes modified with 1 wt%, 3 wt%, and 5 wt% QBTS aqueous solutions" either in the "Nanofiltration Membrane Preparation" chapter or when QBTS-1 is first mentioned.*

Our Response: Thanks for the comments. Based on the reviewer's suggestion, we have provided further detailed explanations for QBTS-1, QBTS-3, and QBTS-5 in the manuscript, specifically in the "Nanofiltration Membrane Preparation" section and where QBTS-1 is first mentioned.

MS-Line 328 We first evaluated the water flux of the functionalized membranes with different QBTS monomer concentrations, i.e., QBTS-1 (1 wt%), QBTS-3 (3 wt%), and QBTS-5 (5 wt%) (Fig. 4a).

MS-Line 679 The PIP-TMC NF membranes were modified ~~with 1 wt%, 3 wt%, and 5 wt% QBTS solutions~~ with QBTS solutions at different mass fractions of 1 wt%, 3 wt%, and 5 wt%. The resulting NF membranes are denoted ~~were designated~~ as QBTS-1, QBTS-3, and QBTS-5, respectively.

Reviewer (Comment 5): *In lines 118-215, an inert graphene pore model with functional groups (-H, -OH, -COO$^-$, -R_3N^+) modified on the pore edges is used to explore the mechanism. However, the functional groups on the active layer of the modified membrane in subsequent experiments are polyhydroxy quaternary ammonium structures (-$N(OH)_n$), resulting in differences in the type and distribution of functional groups between the two systems. It is suggested to supplement the DFT calculation of the interaction energy between -NOH groups and water molecules, following the method used for Figure 1c.*

Our Response: Thanks for the insightful comments. We calculated the interaction energy between the characteristic functional group of QBTS, namely $(CH_2CH_2OH)_3(C(CH_2OH)_3)N^+$, and a single water molecule using the same conditions as those employed for calculating the interaction energy between functional groups and water molecules in Fig. 1c. The equilibration process of the water molecule with the QBTS structure is shown in Fig. R2. As expected, the interaction energy between the polyhydroxy quaternary ammonium functional group and a single water molecule is -18.3 kcal mol^{-1}, which is much lower than that between the three functional groups (-OH, -COO$^-$, -R_3N^+) and water molecules, indicating that the polyhydroxy quaternary ammonium functional group has a stronger ability to disrupt the H-bond networks of water clusters.

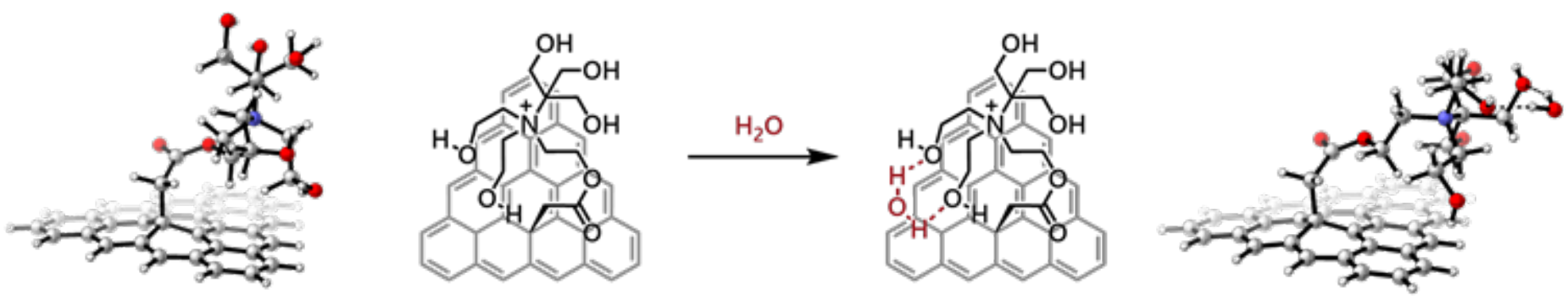

Fig. R2. Schematic diagram of the interaction between polyhydroxy quaternary ammonium functional group and water molecule.

To ensure a more logical structure in the manuscript and avoid an abrupt introduction of the polyhydroxy quaternary ammonium functional group in the first part of the article, we only discuss the interactions of -OH, -COOH, and -R_3N^+ with water molecules in Fig. 1c. The above data regarding the polyhydroxy quaternary ammonium functional group are presented in the last of this section. A discussion on the interaction between the polyhydroxy quaternary ammonium functional group and water molecules has been added to the manuscript as follow.

MS-Line 235 In contrast, we propose that the polyhydroxy quaternary ammonium structure could further facilitate the dissociation of water clusters—the positively charged ~~R_3N^+~~ quaternary-N^+ center attracts water clusters to the active sites via electrostatic adsorption, and the polyhydroxy network with rich hydrogen-bond donating sites, can more effectively promote cluster dissociation. DFT calculation results show that the polyhydroxy quaternary ammonium structure indeed exhibits much stronger functional group-water interaction energies (-18.3 kcal mol^{-1}) than carboxyl and hydroxyl groups (Fig. 1c and Fig. 2e). Meanwhile, hydrogen bonds with longer lifetimes relative to the water-water network can form near QBTS, indicating that compared with water itself, water molecules prefer to interact with the polyhydroxy structure. Combining these results, we speculate that the polyhydroxy quaternary ammonium structure may facilitate the dissociation of water clusters, showing potential to facile water transport. Based on the above results, in the next section, we will introduce the polyhydroxy quaternary ammonium salt (QBTS) into the active layer of the NF membrane, which is expected to promote the transmembrane transport of water molecules via the water cluster dissociation mechanism.

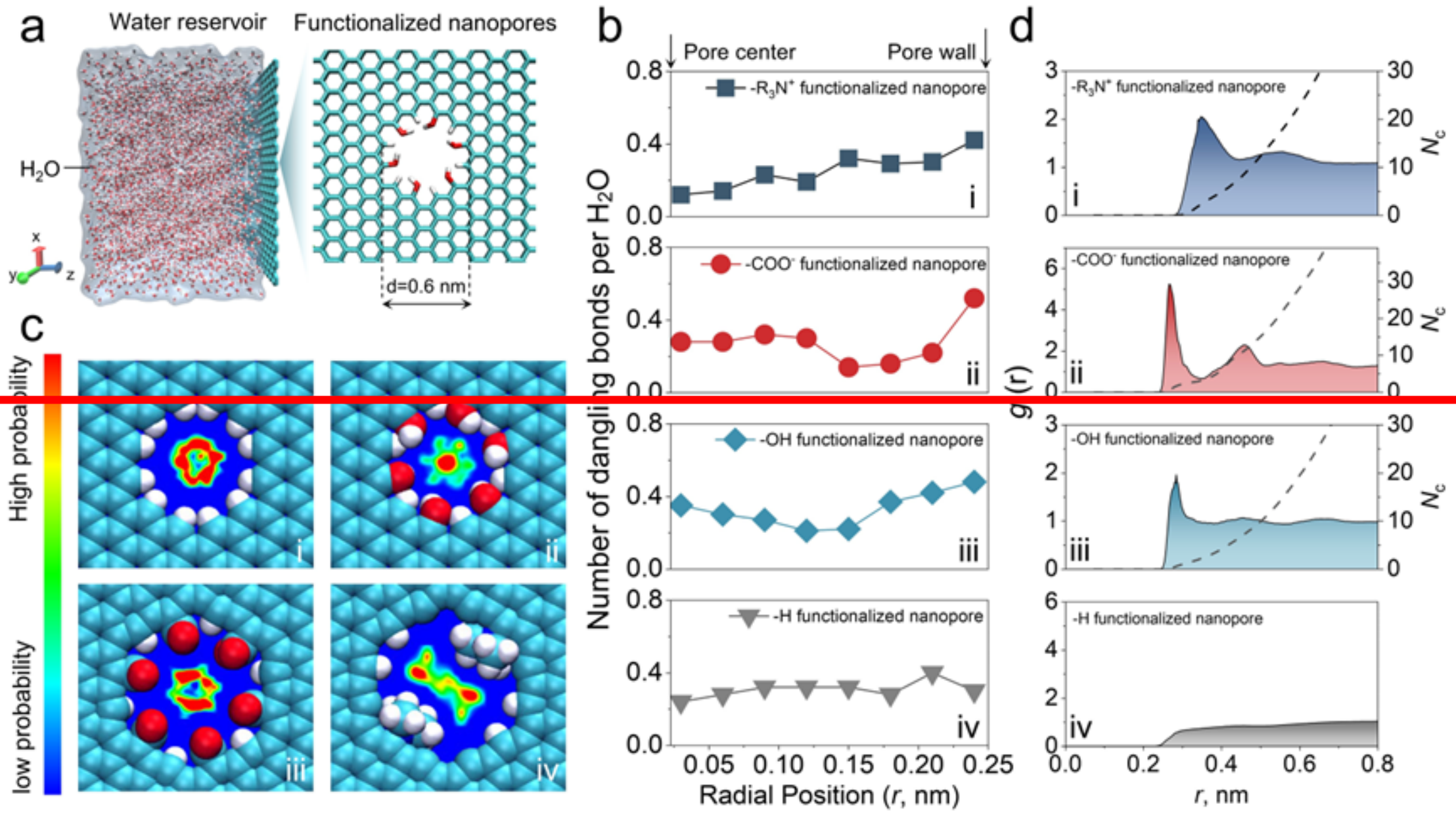

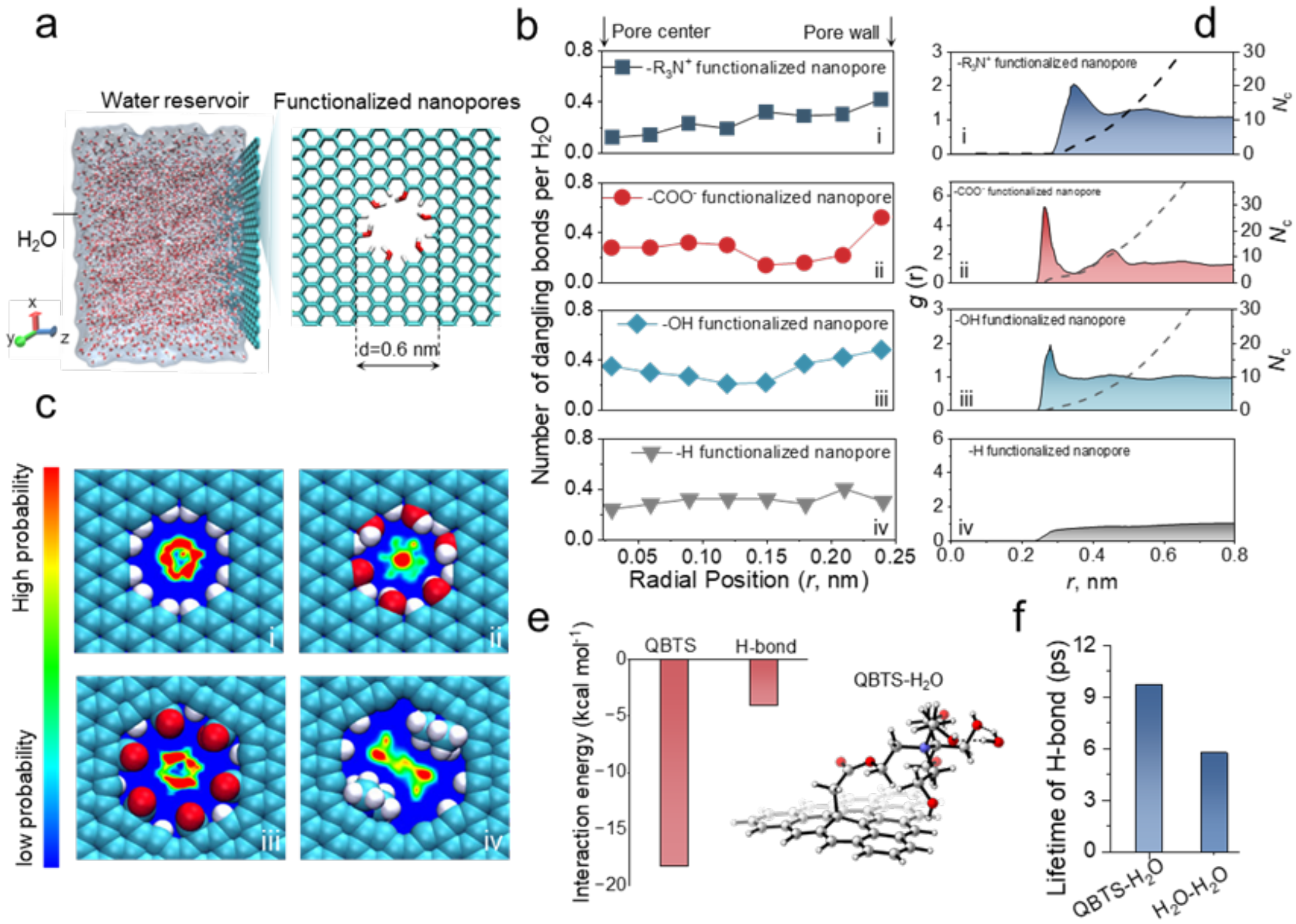

Fig. 2 Mechanisms of water cluster-dissociation from hydration competition by functional groups. (a) Side view of the MD simulation system investigated in this work. (b) Dangling bond radial distribution of water clusters in different functionalized pores. (c) Oxygen density maps in different functionalized pores, i-iv represents -H, -OH, -COO^- and -R_3N^+ functionalized pores, respectively. (d) RDFs between water molecule and the functional groups, and the corresponding coordination numbers (N_c), i.e., N-O_w, O-H_w, and H-O_w for -R_3N^+, -COO^- and -OH, respectively. (e) The interaction energy between a single water molecule and the QBTS functional group. The inset shows the double-sites interaction between QBTS and water molecule. (f) The H-bond lifetime of QBTS-water and water-water calculating by MD simulation.

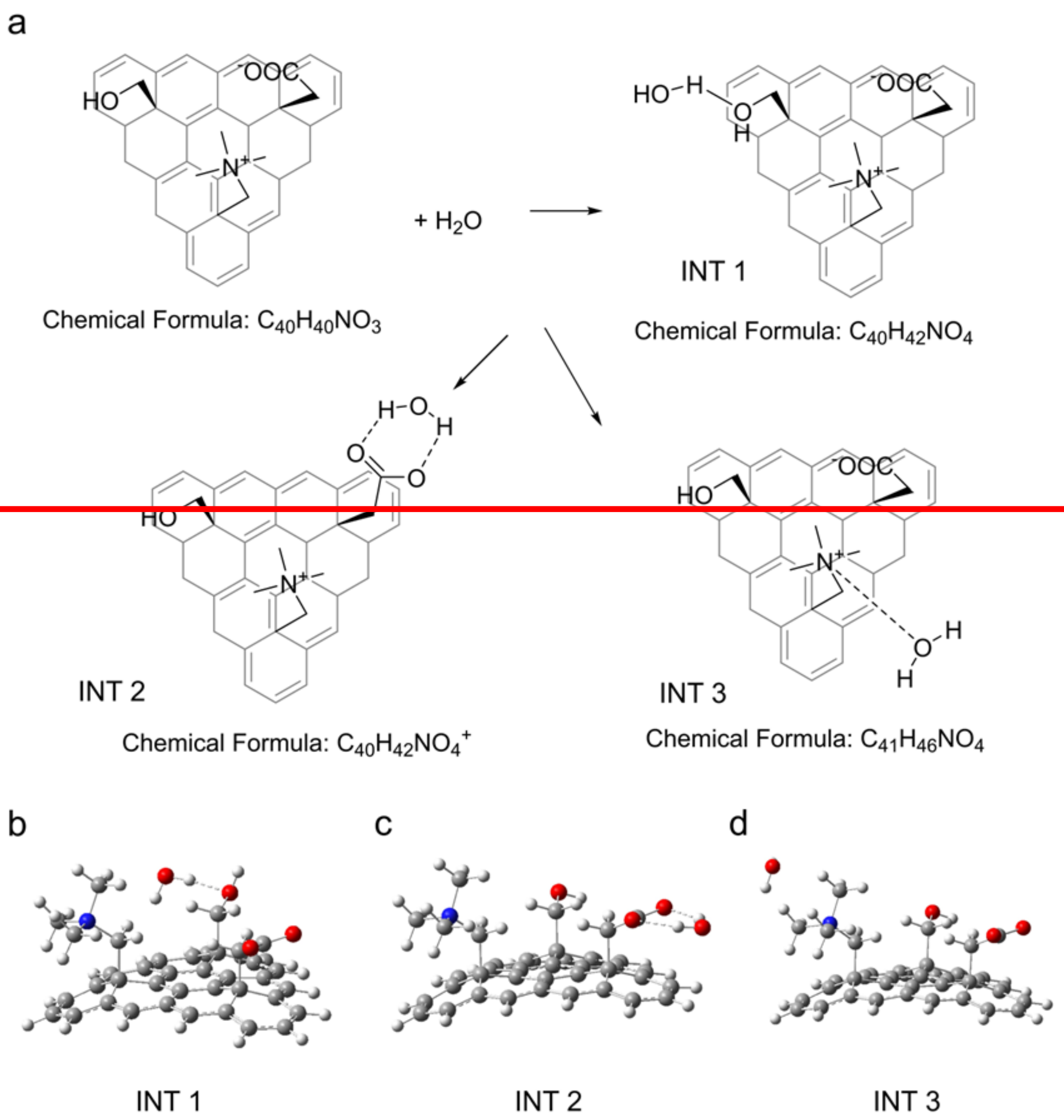

a
HO
-OOC
N+
+ H2O
HO-H
O
H
INT 1
Chemical Formula: C40H40NO3
Chemical Formula: C40H42NO4
H-O
H
O
O
INT 2
INT 3
O
H
H
Chemical Formula: C40H42NO4+
Chemical Formula: C41H46NO4
b
c
d
INT 1
INT 2
INT 3

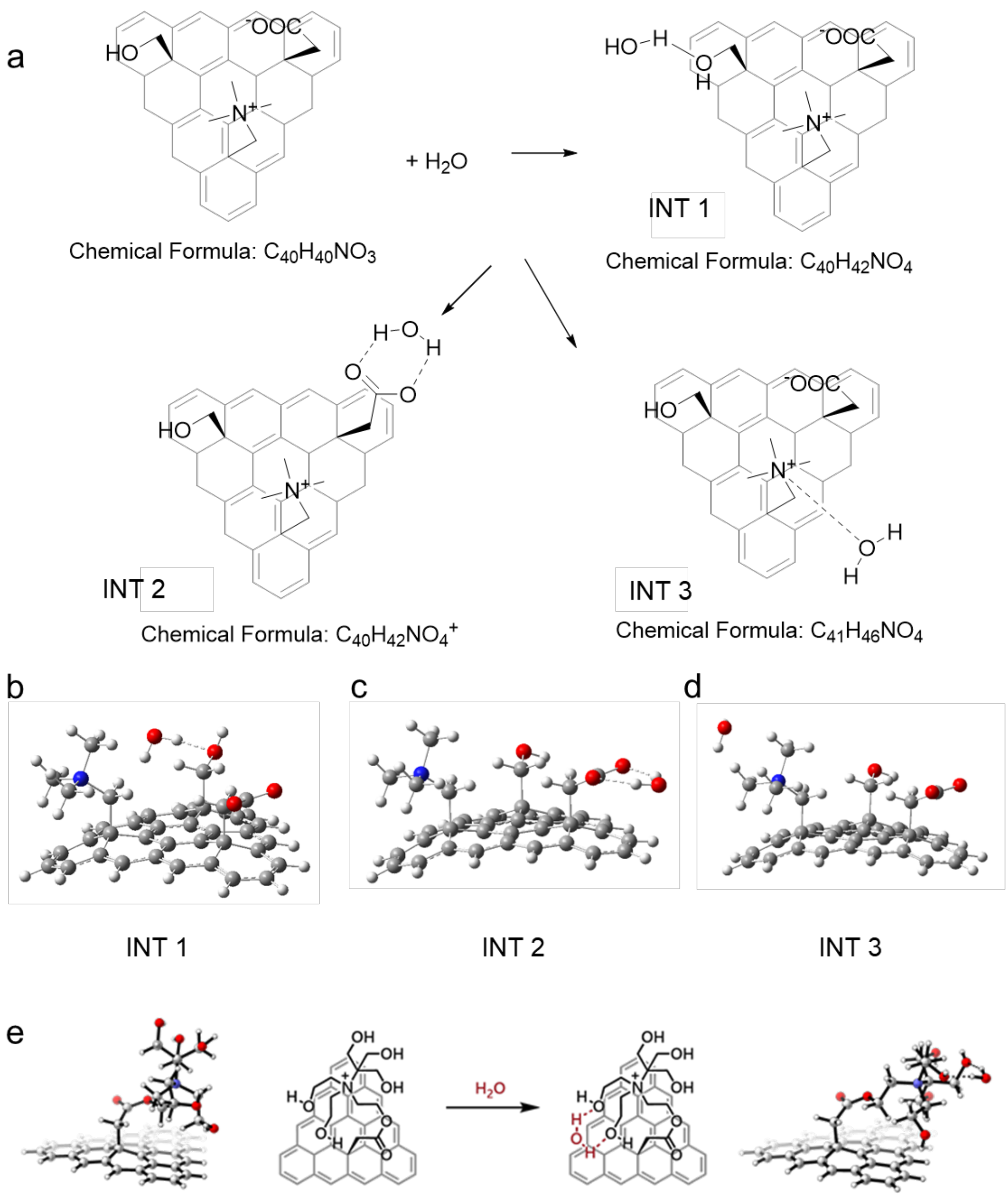

Fig S1. Interaction mode between the studied functional groups and water molecules for calculating the interaction energy. (a) Schematic diagram of the interactions between water molecules and different functional groups. Ball-and-stick representation showing interaction between water molecule and (b) -OH, (c) $-COO^-$ and (d) $-R_3N^+$. (e) Schematic diagram of the interaction between polyhydroxy quaternary ammonium functional group and water molecule. The illustration on the right shows the double-sites interaction between QBTS and water molecule. We grafted the functional groups onto an inert plane composed of nine benzene rings to investigate the interaction energies between the functional groups and water molecules.

Reviewer (Comment 6): *In Lines 118-215, the author modifies the functional groups inside the pores of perforated graphene, while in subsequent experimental studies, the modification is conducted on the membrane surface, leading to inconsistency between the two approaches.*

Our Response: We thank the reviewer for the detailed comment. To simplify the QBTS membrane model, we chose perforated graphene as a substitute for the membrane pore and directly functionalized the pore edge with -H, -OH, -COOH, and $–R_3N^+$, as reported in many nanofluidic studies[1, 2, 3, 4, 5]. For -H and -OH, the functional groups cling tightly to the edge of the pore, appearing as if they are inside the pore. For the polyatomic groups -COOH and $-R_3N^+$, because their orientations are free, they extend outward from the pore edge. This can be observed more clearly in the side view of the model (Fig. S2). This indicates that, in our study, the functional groups are not entirely located "inside the pore" but rather near the edge of the pore entrance.

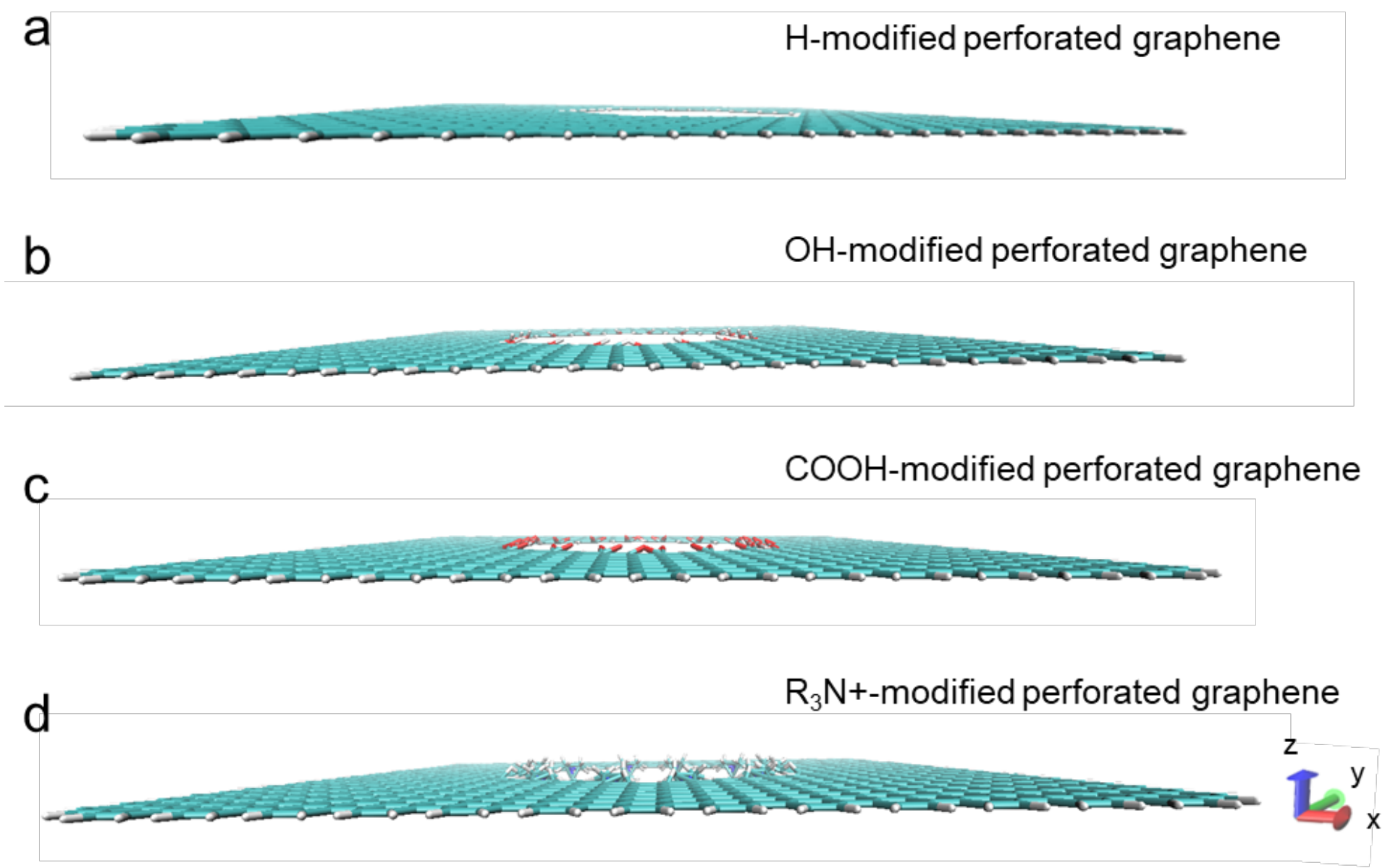

Fig. S2 Front view of perforated graphene pore modified by (a) -H. (b) -OH. (c) -COOH and (d) $-R_3N^+$. We can see that the functional groups are not entirely located "inside the pore" but rather near the edge of the pore entrance.

In this study, although we adopted a "surface modification" approach, since QBTS is fully immersed in the nascent polyamide membrane surface layer, the grafted polyhydroxy quaternary ammonium groups grow randomly near the surface layer and may even extend into the PA membrane pores, rather than being distributed exclusively on the surface. We further employed XPS depth profiling to analyze the distribution of quaternary-N^+ along the depth direction. Typically, XPS can probe chemical information up to about 5 nm from the surface [6, 7]. As shown in Fig. S7, the quaternary-N^+ information from the membrane surface to a depth of 25 nm was investigated. A certain amount of quaternary-N^+ was still observed at a depth of about 25 nm inside the pore. Based on this fact, it can be inferred that QBTS is

distributed on the membrane surface and inside the pores near the surface.

The edge-functionalized perforated graphene model adopted in our study captures the main pore characteristics of the QBTS-modified membrane. Therefore, the effect of functional groups on water cluster structure obtained with this model by MD simulation can reflect the mechanism by which QBTS functional groups influence the change of water clusters near the membrane pores. We have added supplementary explanations to clarify this issue.

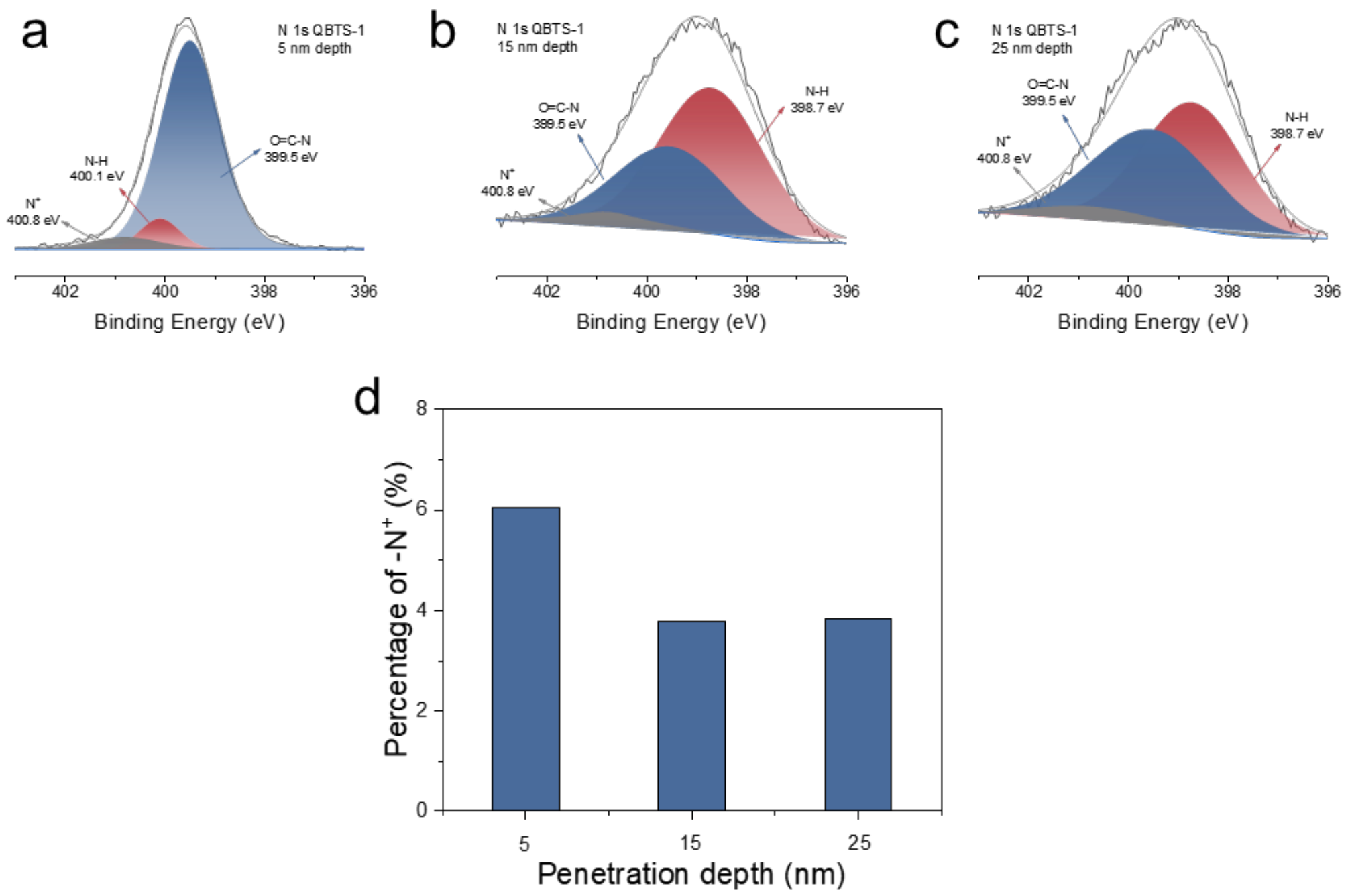

Fig. S7. XPS depth profiling analysis of QBTS-1 membranes. (a)–（c) N 1s X-ray photoelectron spectra of QBTS-1 membranes at depths of 5 nm, 15 nm, and 25 nm, respectively. (d) Content of $-N^{+}$ species in the N 1s X-ray photoelectron spectra of QBTS at the corresponding depths. A certain amount of quaternary-N^{+} was still observed at a depth of about 25 nm inside the pore. Based on this, it can be inferred that QBTS is distributed on the membrane surface and in the pores near the surface. Therefore, edge-functionalized perforated graphene model adopted in our study (Fig. 2a and Fig. S2) captures the main pore characteristics of the QBTS-modified membrane.

MS-Line 179 As illustrated in Fig 2a and Fig. S2, a series of inert graphene pores functionalized at the edges with -H, -OH, $-COO^{-}$, and $-R_3N^{+}$ were constructed to investigate the influence of functional groups on water molecule transport structural changes of water clusters during transport.

References:

1. Cohen-Tanugi D, Grossman JC. Water Desalination across Nanoporous Graphene. *Nano Letters* **12**, 3602-3608 (2012).

2. He Z, Zhou J, Lu X, Corry B. Bioinspired graphene nanopores with voltage-tunable ion selectivity for Na^+ and K^+. *ACS Nano* **7**, 10148-10157 (2013).

3. Fang C, Yu Z, Qiao R. Impact of Surface Ionization on Water Transport and Salt Leakage through Graphene Oxide Membranes. *The Journal of Physical Chemistry C* **121**, 13412-13420 (2017).

4. Guerrero-Aviles R, Orellana W. Energetics and diffusion of liquid water and hydrated ions through nanopores in graphene: ab initio molecular dynamics simulation. *Phys Chem Chem Phys* **19**, 20551-20558 (2017).

5. Yu T, Xu Z, Liu S, Liu H, Yang X. Enhanced hydrophilicity and water-permeating of functionalized graphene-oxide nanopores: Molecular dynamics simulations. *Journal of Membrane Science* **550**, 510-517 (2018).

6. Krishna DNG, Philip J. Review on surface-characterization applications of X-ray photoelectron spectroscopy (XPS): Recent developments and challenges. *Applied Surface Science Advances* **12**, 100332 (2022).

7. Powell CJ, Jablonski A. Surface sensitivity of X-ray photoelectron spectroscopy. *Nuclear Instruments and Methods in Physics Research Section A: Accelerators, Spectrometers, Detectors and Associated Equipment* **601**, 54-65 (2009).

Reviewer (Comment 7): *During the simulation, the force field should be consistent.*

Our Response: We thank the reviewer for this important comment. We agree that force field consistency is important for ensuring the reliability and comparability of MD results. In each independent MD simulation, we kept the force field consistent for all runs within that simulation to ensure comparability among different models in the same MD simulation. In the revised manuscript, we have clarified the simulation protocol to avoid possible misunderstanding. However, we must acknowledge that in this paper we did not use exactly the same force field across three independent and distinct MD simulations, namely: i) structural analysis of water near functionalized perforated graphene; ii) transport behavior of ions and water in polyamide nanochannels; and iii) calculation of H-bond lifetimes near QBTS.

Because the specific sub-questions addressed by these three MD simulations are different and the compositions of their models are not identical, we did not enforce a uniform force field across all three simulations. Instead, we selected a more appropriate force field for each specific system—as has been reported in many other

studies[1, 2, 3, 4, 5, 6], where different force fields are used within the same study to simulate and analyze different processes. More importantly, Liao et al.[2] employed several force fields, such as OPLS-AA, Merz, Netz and Williams FFs (the latter three are named by the authors who developed them) to test the robustness and generality of the ion-transport framework. Their results suggest that while force field choices may affect quantitative details, the main physical trend and theoretical conclusions remained unchanged across different force-field treatments. In addition, the data from the three independent MD simulations in this study were not directly compared quantitatively; rather, we emphasized the trend analysis of water/ion structure and transport in different models. Therefore, we believe that the conclusions derived from combining different MD simulations with experimental data in this study are reliable.

Below is the rationale for force-field selection in this paper:

i) <u>Structural analysis of water near functionalized perforated graphene.</u> Our system involves a graphene surface and various polar/charged functional groups such as -H, -OH, $-COO^-$, and $-R_3N^+$. The OPLS-AA force field has been successfully used to simulate similar complex interface systems, such as graphene-water interfaces, and the behavior of charged molecules in solution, proving its suitability for the “hydration competition” mechanism we investigate[7, 8, 9].

ii) <u>Transport behavior of ions and water in polyamide nanochannels.</u> The PCFF-INTERFACE force field was developed by Heinz et al. specifically for simulating inorganic-organic interfaces. It uses the same functional form as mainstream force fields such as PCFF and CHARMM, but through special parameterization strategies it can accurately describe both organic and inorganic components simultaneously, eliminating systematic biases in interfacial simulations that occur with traditional force fields. It is highly suitable for interfacial simulations, as reported in the literature[10, 11, 12].

iii) <u>Calculation of hydrogen-bond lifetimes near QBTS.</u> GAFF2 is an improved version of GAFF (General Amber Force Field), specifically designed to cover a broad range of organic chemical space, including well-parameterized oxygen-containing functional groups, nitrogen-containing groups, and aromatic systems. For simulating H-bond lifetimes near QBTS grafted onto a graphene surface, there is no dedicated force field. Therefore, we used DFT to optimize the structure and generated high-precision RESP charges along with the GAFF2 force field to achieve a better description[13, 14, 15].

We have refined the Methods section regarding the MD simulations to clarify this concern.

SI-Line 290 Molecular Dynamics Simulations

In this study, we did not use exactly the same force field across three independent and distinct MD simulations, namely: i) Hydration competition between functional groups and water molecules; ii) Confined transport of water molecules in different Nanochannels; and iii) calculation of H-bond lifetimes near QBTS. Because the specific sub-questions addressed by these three MD simulations are different and the compositions of their models are not identical, we did not enforce a uniform force field across all three simulations. Instead, we selected a more appropriate force field for each specific system—as has been reported in many other studies.

SI-Line 316 According to the experimental conditions, several systems were constructed. The initial configurations of the systems were constructed using the Packmol software. The simulations were performed using GROMACS package (version 2023.3) with the all-atom OPLS force field. The OPLS-AA force field has been successfully used to simulate similar complex interface systems, proving its suitability for the “hydration competition” mechanism we investigate.

SI-Line 402 Packmol was used to build both model systems. LAMMPS and PCFF-INTERFACE force field were used to perform the molecular simulations. The PCFF INTERFACE force field can accurately describe both organic and inorganic components simultaneously, eliminating systematic biases in interfacial simulations that occur with traditional force fields. It is highly suitable for interfacial simulations, as reported in the literature.

SI-Line 412 Hydrogen bond characteristic simulation between functional groups and water molecules.

RESP charges were fitted using MultiWFN, and GAFF2 force field parameters for the corresponding molecules were generated using Sobtop. GAFF2 is an improved version of GAFF (General Amber Force Field), specifically designed to cover a broad range of organic chemical space, including well-parameterized oxygen-containing functional groups, nitrogen-containing groups, and aromatic systems. Molecular dynamics simulations were performed using the Gromacs 2021 software. Gromacs is a widely used open-source molecular dynamics simulation software, primarily employed for simulating biomolecular systems such as proteins, lipids, nucleic acids, etc., as well as various molecular systems in materials science. The force field used in this simulation was GAFF2, and the water model was TIP3P. A simulation box of 6 × 6 × 6 nm^3 was constructed, with the multi-hydroxy quaternary ammonium group-modified graphene molecule placed in the center of the box, and the box was filled with water. Na^+ and Cl^- were used to balance the system charge, rendering the overall system electrically neutral. Prior to simulation, energy minimization was first performed to eliminate unreasonable contacts in the initial conformation. This was followed by a 100 ps NVT pre-equilibration simulation. The NVT ensemble refers to a thermodynamic ensemble that maintains constant particle number (N), volume (V), and temperature (T) during the simulation, used to raise the temperature to the target simulation temperature. Subsequently, a 100 ps NPT pre-equilibration simulation was executed. The NPT ensemble refers to a thermodynamic ensemble that maintains constant particle number (N), pressure (P), and temperature (T) during the simulation, used to adjust the system density to the equilibrium value at the target pressure. After system relaxation, the production simulation was carried out. The production simulation used the leap-frog algorithm to integrate Newton's equations of motion, with an integration time step of 2 fs and a total of 50,000,000 integration steps, resulting in a total simulation time of 100.0 ns. The production simulation employed the V-rescale temperature coupling method, with a simulation temperature of 298.15 K. The production simulation used the Parrinello-Rahman pressure coupling method, with a simulation pressure of 1.0 bar. Due to the use of a cutoff distance method, the Particle Mesh Ewald (PME) method was applied to correct for long-range electrostatic interactions. Also due to the use of a cutoff distance method, dispersion correction was applied for long-range van der Waals interactions. Periodic boundary conditions were applied in all three directions throughout the simulation.

References:

1. Wu H, Xu Y, Tang Q, Wu B, Wu P. Microstructure engineering of polyamide membranes for ultrafast polar and non-polar solvent transport. *Nature Communications* **16**, 8414 (2025).

2. Liao S, Liu Y, Li L, Ding L, Wei Y, Wang H. Theoretical framework for confined ion transport in two-dimensional nanochannels. *Nature Communications* **16**, 6675 (2025).

3. Pan Z, *et al.* Molecular manipulation of polyamide nanostructures reconciles the permeance-selectivity threshold for precise ion separation. *Nature Communications* **16**, 7171 (2025).

4. Han S, *et al.* Microporous organic nanotube assisted design of high performance nanofiltration

membranes. *Nature Communications* **13**, 7954 (2022).

5. Lin J*, et al.* Shielding effect enables fast ion transfer through nanoporous membrane for highly energy-efficient electrodialysis. *Nature Water* **1**, 725-735 (2023).

6. Jia Y, Du J, Lu Y, Fang C, Liu P, Zhang S. Voltage-controlled organic solvent nanofiltration using conjugated microporous polymer membranes. *Nat Commun* **17**, 236 (2025).

7. Fang C, Yu Z, Qiao R. Impact of Surface Ionization on Water Transport and Salt Leakage through Graphene Oxide Membranes. *The Journal of Physical Chemistry C* **121**, 13412-13420 (2017).

8. Chen B, Jiang H, Liu X, Hu X. Observation and Analysis of Water Transport through Graphene Oxide Interlamination. *The Journal of Physical Chemistry C* **121**, 1321-1328 (2017).

9. Ouyang J, Sheng Y, Wang W. Water Transport Dynamics in Layered Nanoporous Graphene: An In-Depth Molecular Dynamics Study. *The Journal of Physical Chemistry B* **129**, 8521-8540 (2025).

10. Li X, Zhang G, Liu H, Lan H, Qu J. Sequential Demulsification through the Hydrophobic-Hydrophilic-Hydrophobic Filtration Layer toward High-Performing Oil Recovery. *Environmental Science & Technology* **57**, 12083-12093 (2023).

11. Yan X, Meng Q, Ammami M-T, Wei L. Effect of PAM on Surface Hydrophobicity of Montmorillonite and Difference of Interface Adsorption: An Experimental and Simulation Study. *ACS Omega* **9**, 15818-15832 (2024).

12. Heinz H, Lin T-J, Kishore Mishra R, Emami FS. Thermodynamically Consistent Force Fields for the Assembly of Inorganic, Organic, and Biological Nanostructures: The INTERFACE Force Field. *Langmuir* **29**, 1754-1765 (2013).

13. Hossen MJ, Nematipour A, Bilodeau C. Molecular Scale Hydrophobicity and Adsorption Thermodynamics on Hydrophobic-Charged Surfaces. *ACS Nano* **20**, 6970-6982 (2026).

14. Vassetti D, Pagliai M, Procacci P. Assessment of GAFF2 and OPLS-AA General Force Fields in Combination with the Water Models TIP3P, SPCE, and OPC3 for the Solvation Free Energy of Druglike Organic Molecules. *Journal of Chemical Theory and Computation* **15**, 1983-1995 (2019).

15. Lim VT, Bayly CI, Fusti-Molnar L, Mobley DL. Assessing the Conformational Equilibrium of Carboxylic Acid via Quantum Mechanical and Molecular Dynamics Studies on Acetic Acid. *Journal of Chemical Information and Modeling* **59**, 1957-1964 (2019).

<u>Reviewer (Comment 8)</u>: *In Lines 305-306, the conclusion that "the water flux of the membrane increases with the increase of hydroxyl content" is open to question.*

Our Response: We thank the reviewer for the valuable comment. After carefully examining the experimental data and the characteristics of the different monomers, we strongly agree that the statement "*the water flux of the membrane increases with the increase of hydroxyl content*" is not rigorous enough, as the water flux does not monotonically increase with the number of hydroxyl groups of the monomers. On the one hand, the differences among the monomers QTAEA, BTS, QTEOA, and QBTS are not limited to hydroxyl content, but also include the quaternary ammonium center, the reactivity of side-chain groups, and the spatial configuration. On the other hand, because the reaction pathways of these monomers are not identical, their effects on the physicochemical properties of the membrane cannot be precisely controlled. Therefore, directly correlating the increase in membrane flux with hydroxyl content is also insufficiently rigorous. We also used the *N, N, N*-triethyl-N-(2-aminoethyl) ammonium bromide (ATAB) as an additional monomer with quaternary-N^+ but without -OH to investigate the role of quaternary-N^+ on water permeance (Details are shown below). Accordingly, we have revised the relevant text in the manuscript as follows:

MS-Line 343 To gain deeper insights into the mechanism by which surface functional groups on the active layer enhance water flux, we selected ~~four~~ five amine monomers with similar spatial structures but different functional groups to functionalize the TMC-PIP membrane, i.e., *N*, *N*, *N*, *N*-Tetra (2-aminoethyl) ammonium bromide (QTAEA), Bis-(2-Hydroxyethyl) amino-tris (Hydroxymethyl) methane (BTS), *N*, *N*, *N*-triethyl-N-(2-aminoethyl) ammonium bromide (ATAB), *N*, *N*, *N*-Tris (2-hydroxyethyl)-*N*-(2-aminoethyl) ammonium bromide (QTEOA) and QBTS (details in Fig. 4b and Fig. S9). ~~As the hydroxyl content in the monomer increased, the water flux of the functionalized membranes also improved, following the order: QTAEA < BTS < QTEOA < QBTS (Fig. 4b). We observed that the water flux of the membrane modified with QTAEA was even lower than that of the pristine PIP-TMC membrane. More importantly, the water flux of the membranes modified with BTS was lower compared to QTEOA and QBTS functionalized membranes, i.e., BTS < QTEOA < QBTS (Fig. 4b).~~ The water flux order of the functional membranes is as follows: QTAEA < BTS < ATAB < QTEOA < QBTS (Fig. 4b). In the absence of hydrophilic hydroxyl groups, the highly reactive -NH_2 of QTAEA undergo multi-point reactions with residual acyl chlorides, forming a locally dense structure that is unfavorable for water permeation; consequently, its water permeance is even lower than that of PIP-TMC. For BTS, although it contains a relatively high number of hydroxyl groups, it lacks a quaternary-N^+, showing a modest improvement in water flux. Previous studies have shown that the hydration free energy of quaternary ammonium cations is significantly more favorable than that of neutral amines, meaning that the quaternary-N^+ center itself is a good hydration site—as reflected by the slight increase in water flux observed for ATAB compared with PIP-TMC. QTEOA possesses both a polyhydroxy structure and a quaternary-N^+ center, but its high reactive -NH_2 likely leads to a relatively dense active layer. In contrast, QBTS is grafted via a less reactive esterification reaction and possesses both polyhydroxy groups and a quaternary-N^+ center, thus exhibiting a significantly enhanced water-transport effect compared with the other monomers. ~~Based on the binding energy data in Fig. 1c, we hypothesize that there may be a synergistic effect between the -OH and -R_3N^+ quaternary-N^+ on the dissociation of water clusters to facilitate the transport of water molecules.~~ Overall, we suppose that when a monomer contains both abundant polyhydroxy structures and a quaternary-N^+ center, it enhances the water permeance of the membrane.

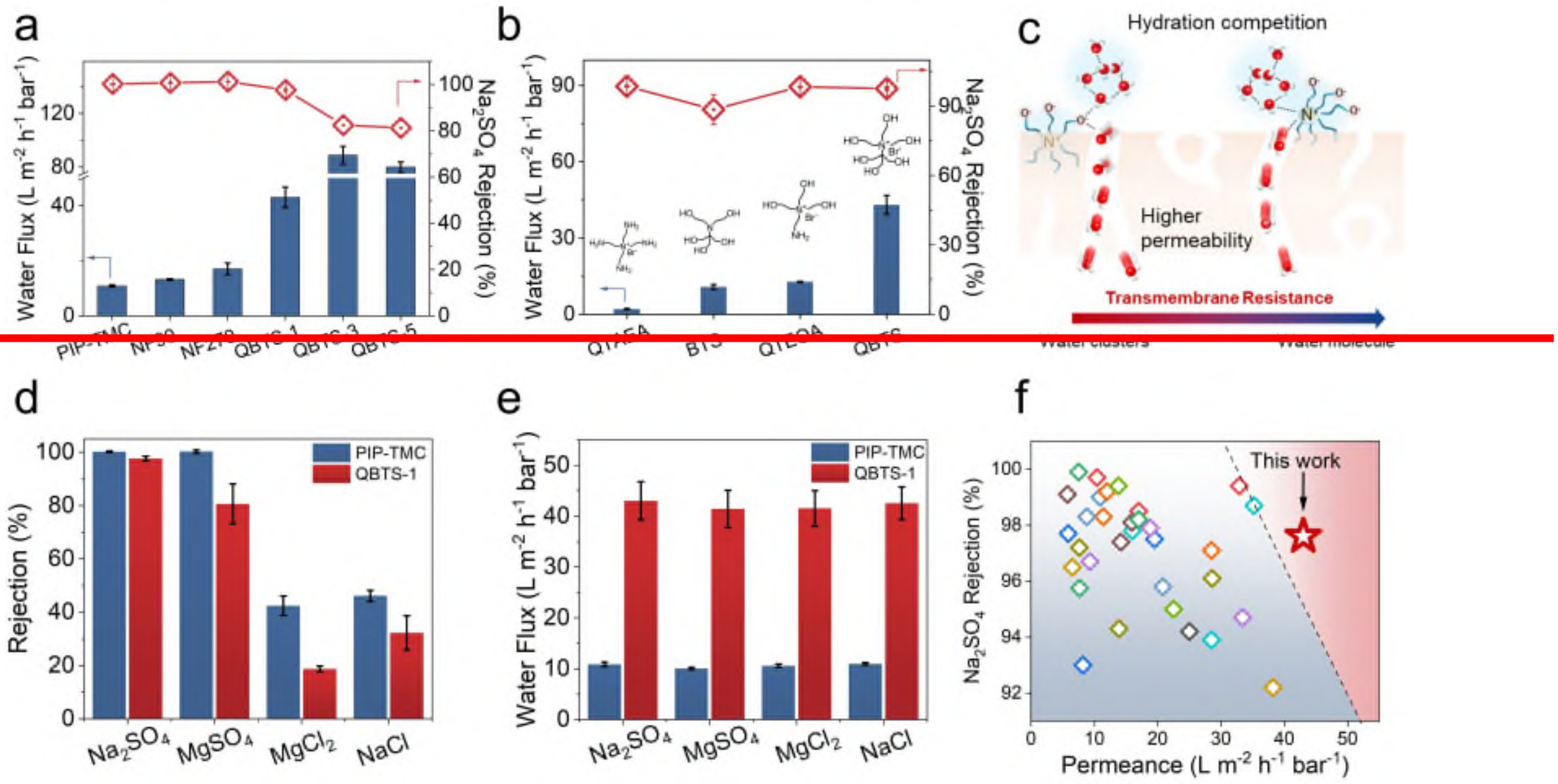

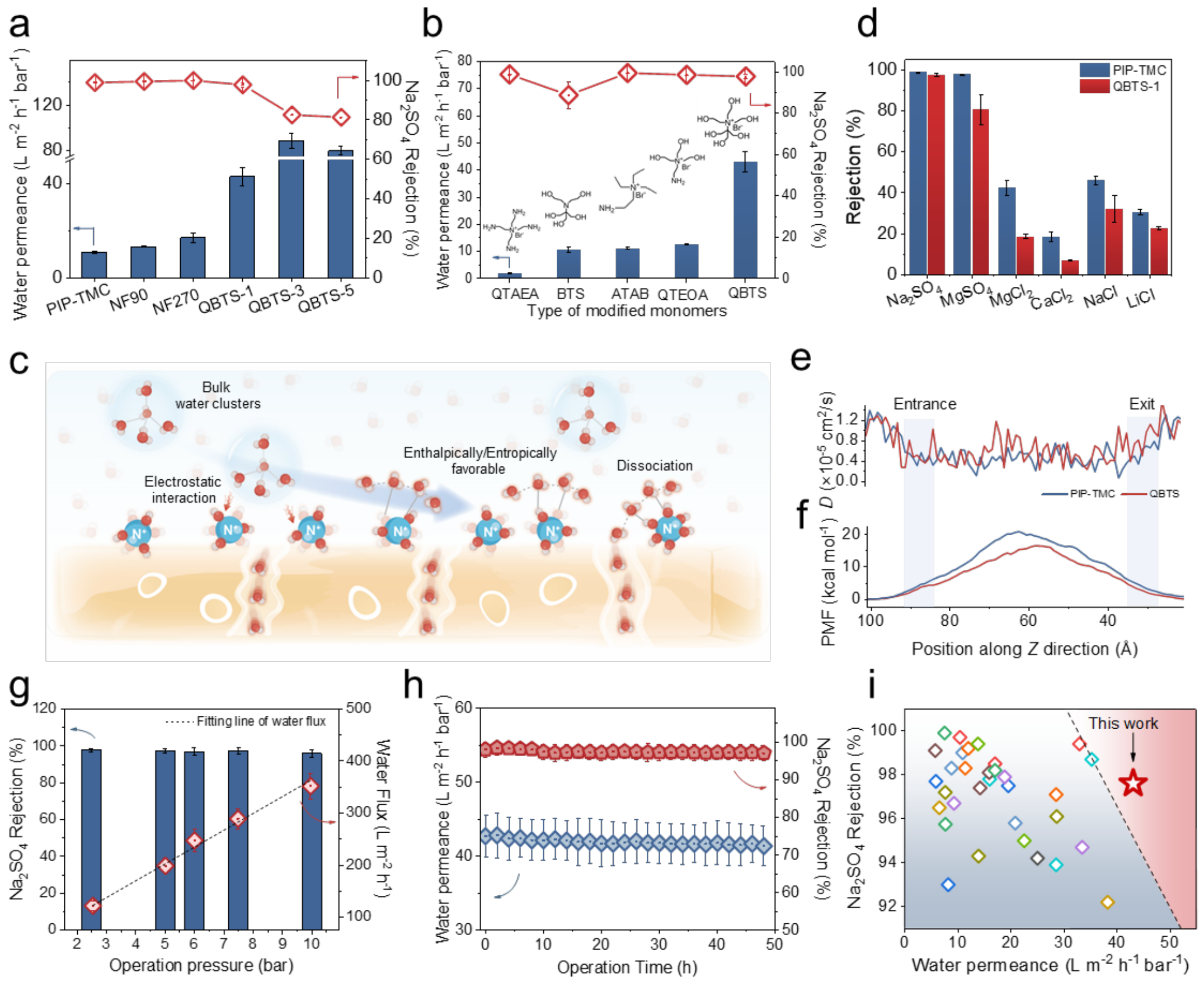

Fig. 4 Filtration performance of QBTS functionalized NF membranes. (a) Effect of QBTS monomer concentration on the separation performance of NF membranes (feed: 1000 ppm Na_2SO_4, 6 bar pressure). (b) Filtration performance of NF membranes modified with different ~~amine~~ monomers (feed: 1000 ppm Na_2SO_4, 6 bar pressure). (c) Schematic diagram of the suppositional mechanism of functional groups promoting the dissociation of water clusters. The dashed lines between water molecules indicate that the original water-water hydrogen bonds are weakened by the polyhydroxy structure of QBTS. (d) Salts rejection ~~and (e) water flux~~ of Na_2SO_4, $MgSO_4$, $MgCl_2$, $CaCl_2$, LiCl and NaCl by PIP-TMC and QBTS-1

membranes (Salt concentration, 1000 ppm; applied pressure, 6 bar; temperature, 25 °C). (e) The potential of mean force (PMF) and (f) diffusion coefficients (*D*) of Cl^- along the diffusion coordinate in PIP TMC and QBTS simulated nanochannels. The filtration performance of QBTS-1 under different (g) operation pressure and (h) operation Time (feed: 1000 ppm Na_2SO_4, 6 bar pressure). (f) (i) Performance comparison of QBTS-1 membrane with the state-of-the-art NF membranes using surface modification in reported literature (details in Table. S2).

Below is our specific analysis of the changes in water flux of the modified membranes after modification with different monomers. Specifically, for QTAEA, it contains multiple highly reactive primary amine groups ($-NH_2$), which are expected to undergo multi-point reactions with residual acyl chlorides[1, 2, 3]. In addition, its charged nature and relatively large steric volume make it prone to enrichment on the membrane surface [2, 3]. Therefore, after QTAEA surface treatment, a locally dense structure may form on the membrane surface [2, 3], corresponding to lower free volume and lower water permeance, which is unfavorable for water permeation. Thus, the significantly reduced flux of the QTAEA-modified membrane can be more reasonably attributed to surface densification. For BTS, it can also be grafted via esterification of its hydroxyl groups with residual acyl chlorides[4, 5]. The additional hydrophilic hydroxyl groups can improve water flux to some extent. Meanwhile, previous studies have confirmed that the hydration free energy of quaternary-N^+ is significantly more favorable than that of neutral amines[6], meaning that the quaternary-N^+ center itself is a stronger hydration site. The neutral tertiary amine backbone of BTS and its fewer hydroxyl groups result in only a modest increase in water flux. We also supplemented surface grafting modification using *N*,*N*,*N*-triethyl-N-(2-aminoethyl)ammonium bromide (ATAB) as a monomer (Fig. S5). We found that even without hydroxyl groups, the quaternary-N^+ center alone still brought about a slight increase in water flux. For QTEOA, it can be more effectively grafted onto the membrane surface through the reaction of its $-NH_2$ with residual acyl chlorides. The quaternary-N^+ center and polyhydroxy structure are beneficial for increasing water flux. However, the higher reactivity of the $-NH_2$ groups may simultaneously lead to a locally dense structure. These reasons result in a higher water flux after QTEOA modification compared with the other monomers, but still far lower than that of QBTS. In contrast, QBTS is grafted via a less reactive esterification reaction and possesses both polyhydroxy groups and a quaternary-N^+ center, thus exhibiting a significantly enhanced water-transport effect compared with the other monomers. Overall, we speculate that when a monomer contains both abundant polyhydroxy structures and a quaternary-N^+ center, it indeed enhances the water permeance of the membrane.

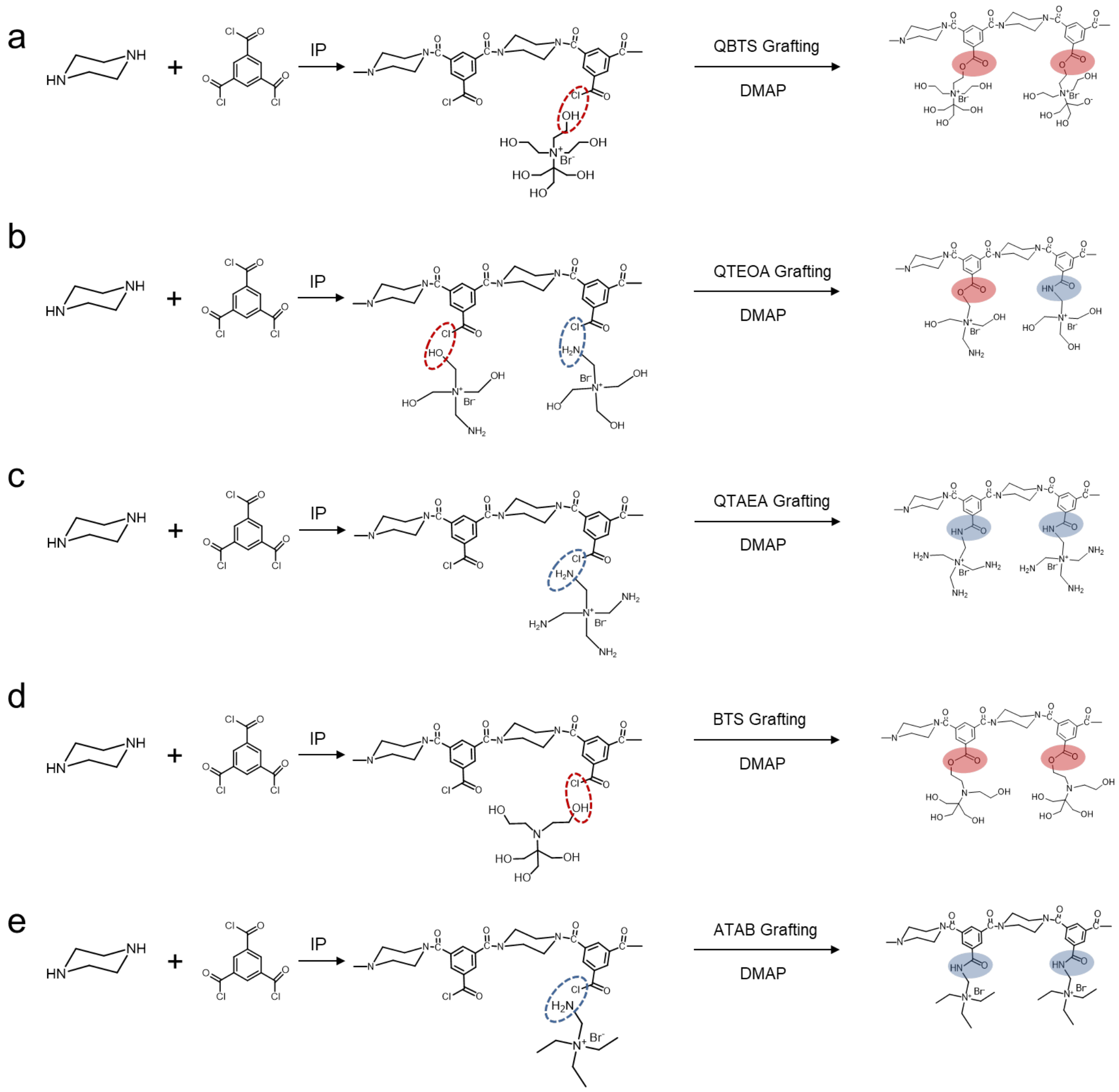

Fig. S5, schematic illustration of the principle of membrane fabrication. (a)-(e) The main reaction process of interfacial polymerization and surface modification by QBTS, QTEOA, QTAEA, BTS, and ATAB, respectively. In this study, QBTS was grafted onto the membrane surface via an esterification reaction between its hydroxyl groups and the residual acyl chloride groups of TMC within the freshly formed polyamide active layer. This grafting strategy is widely employed for anchoring hydroxyl-containing functional groups onto polyamide membranes. The process begins with the interfacial polymerization of PIP and TMC to generate a nascent polyamide active layer. Subsequently, under the catalysis of 4-dimethylaminopyridine (DMAP)—a nucleophilic acyl transfer catalyst—the remaining acyl chloride groups of TMC undergo esterification with the hydroxyl groups of QBTS, thereby covalently attaching QBTS to the polyamide network. (b)-(e) show the reaction principles of other monomers contain hydroxyl or primary amine groups for surface modification. The hydroxyl groups react with acyl chloride groups to form ester bonds, while the primary amine groups react with acyl chloride groups to form amide bonds.

References:

1. Baig U, Waheed A. Exploiting interfacial polymerization to fabricate hyper-cross-linked

nanofiltration membrane with a constituent linear aliphatic amine for freshwater production. *npj Clean Water* **5**, (2022).

2. Wanjiya M, Jin C-G, Shen Y, Yin M-J, An Q-F. Conversion of membrane surface charge property via post-crosslinking for heavy metal ions removal. *Separation and Purification Technology* **362**, (2025).

3. Yu D, Li M, Zhou H, Liang X, Shou Q, Liu H. Polyamide composite nanofiltration membrane via a combination of polydopamine grafting and reverse interfacial polymerization for high Mg2+/Li+ separation capacity. *Separation and Purification Technology* **355**, (2025).

4. Li N, *et al.* Tuning composite nanofiltration membranes with γ-cyclodextrin for improved Mg2+/Li+ selectivity. *Separation and Purification Technology* **330**, 125206 (2024).

5. Li J, Peng H, Liu K, Zhao Q. Polyester Nanofiltration Membranes for Efficient Cations Separation. *Advanced Materials* **36**, 2309406 (2024).

6. Singh H, Sharma S. Understanding the Hydration Thermodynamics of Cationic Quaternary Ammonium and Charge-Neutral Amine Surfactants. *J Phys Chem B* **126**, 9810-9820 (2022).

Reviewer (Comment 9): *Figure 4c fails to demonstrate the competitive process between "the hydrogen bonding interaction between -OH/-R_3N^+ and water molecules". Consequently, it cannot well explain why the performance of BTS is inferior to that of QTEOA.*

Our Response: We greatly appreciate the reviewer's comment. We would like to clarify that Fig.4c presents a hypothetical mechanism of how the polyhydroxy quaternary ammonium structure of QBTS may affect water cluster structure, which is proposed based on our experimental observations combined with previous studies on the influence of functional group–water interactions on water cluster structure.

Specifically, according to others studies, when functional groups possess H-bond acceptors/donors that can interact more strongly with water molecules, they tend to form stronger local interactions with water preferentially, thereby "capture" water molecules, creating oriented anchoring, cutting off or weakening the original water-water H-bond connectivity, reducing the thermodynamic benefit of maintaining the tetrahedral network, and transforming the continuous ordered water network into a more disordered, defect-rich, higher-entropy local structure[1, 2, 3, 4, 5].

Previous studies have indicated that such perturbation of water clusters by surface functional groups can generate smaller water clusters that are more favorable for transport[6, 7, 8], provide more water molecular orientations to reduce the entropic penalty[9],

and simultaneously lower the energy barrier of the dissociation process through a compensation effect[9, 10, 11]. This facilitates water partitioning into the membrane phase[9]. These observations provide a reasonable basis for inferring that the polyhydroxy quaternary ammonium structure of QBTS near the membrane pores enhances water flux by influencing water cluster structure.

Meanwhile, previous studies have confirmed that the hydration free energy of quaternary-N^+ is significantly more favorable than that of neutral amines[12], meaning that the quaternary-N^+ center itself may attract water clusters toward the membrane pore through electrostatic interactions between charge and water molecular dipole.

Thus, Fig. 4c can be described as follows: the polyhydroxy quaternary ammonium structure on the membrane surface can attract water clusters toward the membrane pore via electrostatic interactions. When water clusters interact with the polyhydroxy structure, dissociation of the water clusters occurs. This process has three effects: i) the formation of smaller water clusters; ii) a reduction in the enthalpic barrier required for dissociation through a compensation effect; and iii) providing more orientations for water molecules, thereby reducing the entropic penalty. Consequently, this process is entropically and enthalpically favorable. We must emphasize again, that Fig. 4c just presents a hypothetical mechanism based on partial experimental observations and previous studies, and this mechanism is further evidenced by the results shown in Fig. 1, Fig. 2, and Fig. 5.

Regarding the reasons for the performance differences among membranes modified with different monomers, we attribute this to three factors: i) differences in the density of the active layer caused by variations in the reactivity between the monomers and the nascent polyamide layer; ii) differences in the "dissociation" effect resulting from the varying number of hydroxyl groups; and iii) the presence or absence of quaternary-N^+ to provide a more easily hydrated membrane surface. We have discussed this in detail in Comment 8 and kindly refer the reviewer to the corresponding section. Accordingly, we have revised the manuscript as follows:

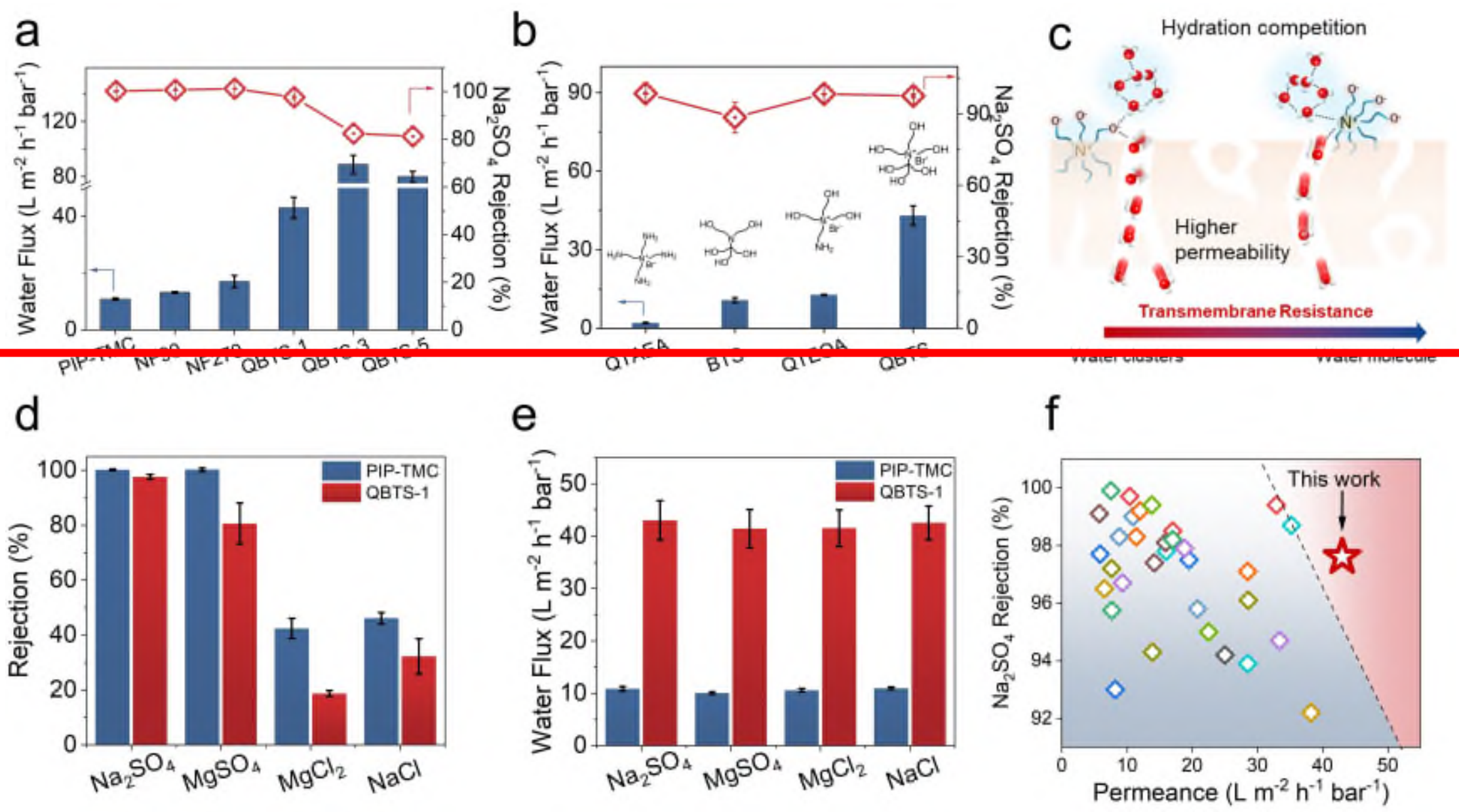

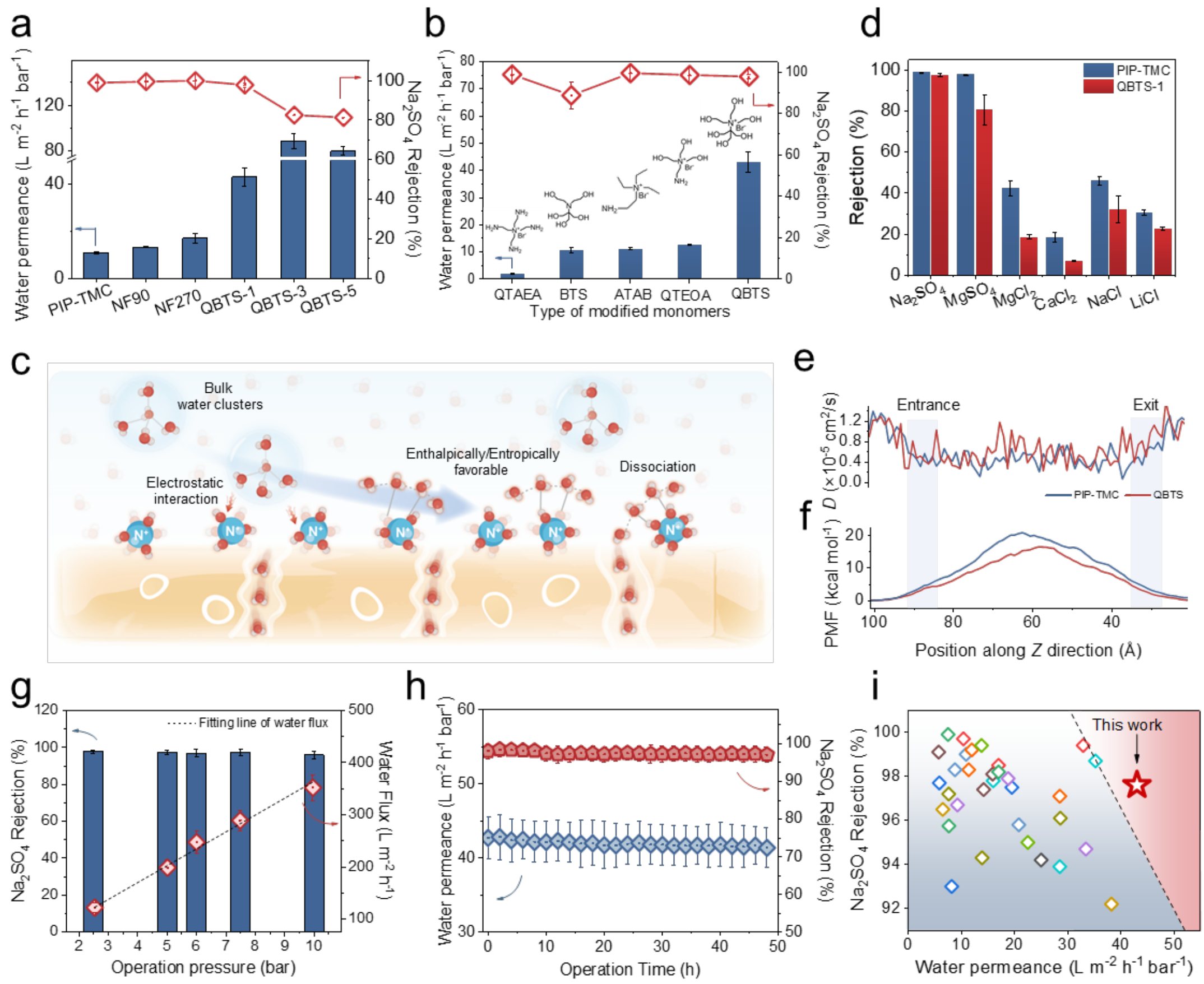

Fig. 4 Filtration performance of QBTS functionalized NF membranes. (a) Effect of QBTS monomer concentration on the separation performance of NF membranes (feed: 1000 ppm Na_2SO_4, 6 bar pressure). (b) Filtration performance of NF membranes modified with different ~~amine~~ monomers (feed: 1000 ppm Na_2SO_4, 6 bar pressure). (c) Schematic diagram of the suppositional mechanism of functional groups promoting the dissociation of water clusters. The dashed lines between water molecules indicate that the original water-water hydrogen bonds are weakened by the polyhydroxy structure of QBTS. (d) Salts rejection ~~and (e) water flux~~ of Na_2SO_4, $MgSO_4$, $MgCl_2$, $CaCl_2$, LiCl and NaCl by PIP-TMC and QBTS-1

membranes (Salt concentration, 1000 ppm; applied pressure, 6 bar; temperature, 25 °C). (e) The potential of mean force (PMF) and (f) diffusion coefficients (*D*) of Cl^- along the diffusion coordinate in PIP TMC and QBTS simulated nanochannels. The filtration performance of QBTS-1 under different (g) operation pressure and (h) operation Time (feed: 1000 ppm Na_2SO_4, 6 bar pressure). (f) (i) Performance comparison of QBTS-1 membrane with the state-of-the-art NF membranes using surface modification in reported literature (details in Table. S2).

MS-Line 371 Based on the binding energy data in Fig. 1c, we hypothesize that there may be a synergistic effect between the -OH and -R_3N^+ on the dissociation of water clusters to facilitate the transport of water molecules. Overall, we suppose that when a monomer contains both abundant polyhydroxy structures and a quaternary-N^+ center, it enhances the water permeance of the membrane.

The physicochemical characteristics and hydraulic permeability results of QBTS (Fig. S14) also indicate that the enhancement in water permeance mainly originates from the membrane surface chemistry. Based on the results shown in Fig. 2b-2c and 2e-2f, and previous reports, the polyhydroxy structure of QBTS is expected to exhibit stronger interactions with water molecules, thereby perturbing or even dissociating water clusters and forming a more disordered, defect-rich, higher-entropy local structure. Moreover, the positively charged quaternary-N^+ center has also been reported to attract water molecules, facilitating the formation of a hydration layer. Based on these facts, we speculate the following mechanism for the enhanced water permeance of QBTS. Specifically, as shown in Fig. 4c, during the partitioning process where water clusters from the aqueous phase go into the membrane phase driven by pressure, larger water clusters approach the active sites through the electrostatic adsorption by -R_3N^+ quaternary-N^+ in QBTS and undergo dissociation due to the hydration competition of -R_3N^+ and -OH with H-bond donor/acceptor. It allows water clusters to enter the nanochannels in polymeric membranes with smaller traversing species, thereby reducing the resistance to water transport for higher water flux.. Subsequently, the polyhydroxy network in QBTS preferentially interacts with water molecules, disrupting the original water-water network and forming a locally loose water structure. Numerous previous studies have shown that such functional group–water interactions can lower the enthalpic barrier for dissociation through a compensation effect and reduce the entropic penalty by providing more available orientations for water molecules. This entropically and enthalpically favorable effect likely contributes to the excellent water flux of QBTS.

MS-Line 26 The results, observed by *in situ* liquid time-of-flight secondary ion mass spectrometry in combination with molecular dynamics simulation, reveal that the functional groups (-OH and -R_3N^+ quaternary-N^+) of membrane pore entrances can enthalpically/entropically favorably dissociate water clusters

MS-Line 98 First, through DFT and molecular dynamics (MD) simulations, we confirmed that -R_3N^+ (R represents Methyl) quaternary-N^+, -OH, and -COO^-, which act as hydrogen bond acceptors/donors, can enthalpically/entropically favorably dissociate water clusters via the strong interactions between functional group and water molecule.

MS-Line 103 In combination with *in situ* liquid time-of-flight secondary ion mass spectrometry (ToF-SIMS) and MD simulation, we found that the QBTS active sites, i.e., -OH and ~~R_3N^+~~ quaternary-N^+ dissociated the water clusters with larger sizes, i.e., $(H_2O)_5H^+$ into smaller species, i.e., $(H_2O)_3H^+$ with higher mobility at less entropic and enthalpic cost. This reduces the ~~entropy and enthalpy~~ energy barriers of water's transmembrane transport and increases their effective transport area, thus leading to higher permeability.

References:

1. Shin S, Willard AP. Water's Interfacial Hydrogen Bonding Structure Reveals the Effective Strength of Surface-Water Interactions. *J Phys Chem B* **122**, 6781-6789 (2018).

2. Zhou L, *et al.* Suppressing Hydrogen Evolution in Aqueous Lithium-Ion Batteries with Double-Site Hydrogen Bonding. *ACS Energy Letters* **8**, 40-47 (2022).

3. Ng LS, *et al.* Chaotropic Nanoelectrocatalysis: Chemically Disrupting Water Intermolecular Network at the Point-of-Catalysis to Boost Green Hydrogen Electrosynthesis. *Angew Chem Int Ed Engl* **63**, e202317751 (2024).

4. Zhu Y, Chen Q, Hao J, Jiao Y. Breaking hydrogen-bonds in aqueous electrolytes towards highly reversible zinc-ion batteries. *Journal of Materials Chemistry A* **12**, 20097-20106 (2024).

5. Guo L, *et al.* Formate-Anion-Induced Water Network Reshaping Enables Concurrent Hydrogen and Magnesium Hydroxide Production From Seawater. *Angew Chem Int Ed Engl*, e6937994 (2026).

6. Liu G, *et al.* New inspirations for efficient water transport in sub-nanopores: The effect of positive charges at the pore entrance. *Journal of Environmental Chemical Engineering* **13**, (2025).

7. Itoh Y, *et al.* Ultrafast water permeation through nanochannels with a densely fluorous interior surface. *Science* **376**, 738-743 (2022).

8. Tunuguntla RH, Henley RY, Yao Y-C, Pham TA, Wanunu M, Noy A. Enhanced water permeability and tunable ion selectivity in subnanometer carbon nanotube porins. *Science* **357**, 792-796 (2017).

9. Cohen-Tanugi D, Grossman JC. Water Desalination across Nanoporous Graphene. *Nano Letters* **12**, 3602-3608 (2012).

10. Wang M, *et al.* A coupled effect of dehydration and electrostatic interactions on selective ion transport through charged nanochannels. *Nanoscale* **10**, 18821-18828 (2018).

11. He Z, Zhou J, Lu X, Corry B. Bioinspired graphene nanopores with voltage-tunable ion

selectivity for Na^+ and K^+. *ACS Nano* **7**, 10148-10157 (2013).

12. Singh H, Sharma S. Understanding the Hydration Thermodynamics of Cationic Quaternary Ammonium and Charge-Neutral Amine Surfactants. *J Phys Chem B* **126**, 9810-9820 (2022).

<u>Reviewer (Comment 10)</u>: *Figure 5b, the water clusters in the bulk solution are mainly composed of 3-5 water molecules. After filtration through both PIP-TMC and QBTS-1 membranes, the water clusters are mainly composed of 3 water molecules, indicating that the pore structure itself can reduce the number of water molecules in the clusters. However, there is a difference in salt rejection between the two membranes. Is the reason merely the reduction in the number of water molecules in the clusters caused by hydration competition? Has the influence of pore size been considered?*

Our Response: We thank the reviewer for the insightful comment. First, we did consider the influence of pore size on structural changes of water clusters during transmembrane transport. The pore size indeed leads to changes in the size of water clusters—as we mentioned in the paper:

"**Line 357** *We thus hypothesize that the steric hindrance of the confined nanochannels and abundant carboxyl functional groups in the polyamide active layer may contribute to the dissociation of water clusters as we mentioned earlier*."

Previous studies have also shown that water clusters are transported through polyamide nanochannels in a form smaller than their bulk-phase size[1] due to the size confinement. We found that the pore size distributions of PIP-TMC and QBTS showed no significant changes, with average effective pore sizes of 0.29 nm and 0.32 nm (Fig. 3d), respectively. This size is smaller than that of most common hydrated species[1, 2, 3]. Therefore, it is reasonable that after filtration, $(H_2O)_nH^+$ exists predominantly as smaller-size $(H_2O)_3H^+$ in both membranes.

However, for QBTS, the polyhydroxy quaternary ammonium structure makes the dissociation of water clusters more favorable relative to the effect of steric hindrance, as evidenced by the higher proportion of $(H_2O)_3H^+$ observed after QBTS filtration compared with the PIP-TMC (Fig. 5b), as well as the lower energy required for the dissociation of water clusters (Fig. 5c and Fig. 5d). This mechanism reduces the transport resistance of water clusters and therefore enhances membrane flux.

As mentioned by the reviewer, QBTS exhibited a decrease in salt rejection compared with the unmodified PIP-TMC (Fig. 4d). Given that we observed no significant change in the pore size distribution of QBTS (Fig. 3d), we speculate that this may be attributed to the unique membrane surface characteristics of QBTS rather than size effect. Based on the solution-diffusion theoretical framework for

nanofiltration[4, 5], salt rejection is generally determined by the ion with the lower permeability (p_i), which is closely related to partitioning (K) and diffusion (D). As shown in Fig. R1, previous studies have demonstrated that the rejection of different salt types responds distinctly to membrane surface charge[6, 7]. For Na_2SO_4, rejection is typically governed by the co-ion SO_4^{2-}. Since QBTS maintains a relatively negative Zeta potential (Fig. 3j), its Na_2SO_4 rejection is well preserved. For $MgSO_4$, we speculate that the more negative Zeta potential, together with the specific complexation of the polyhydroxy structure with Mg^{2+} [8, 9, 10, 11], enhances the partitioning (K) of Mg^{2+} from the aqueous phase into the membrane phase, thereby increasing ion permeability and leading to reduced salt rejection. Furthermore, the Mg^{2+} adsorbed onto the membrane surface further induce charge neutralization[12, 13, 14], which weakens the repulsion of the membrane surface negative charge against co-ions. For $MgCl_2$, as shown in Fig. R1, the salt rejection is determined by the Mg^{2+}. Given the more negative Zeta potential of QBTS and the Mg^{2+} affinity of the polyhydroxy quaternary ammonium structure, the decrease in salt rejection is expected. In comparison, the affinity between Na^+ and QBTS is weaker than that of the divalent Mg^{2+}, which is why the observed reduction in NaCl rejection is less pronounced than that for $MgCl_2$. That is, we attribute the reduction in salt rejection of QBTS to its special surface characteristics rather than to size effects. Meanwhile, as we mentioned earlier, the difference in hydration distribution between PIP-TMC and QBTS is also attributed to the special surface properties of the modified membrane. Therefore, there is no contradiction between the two.

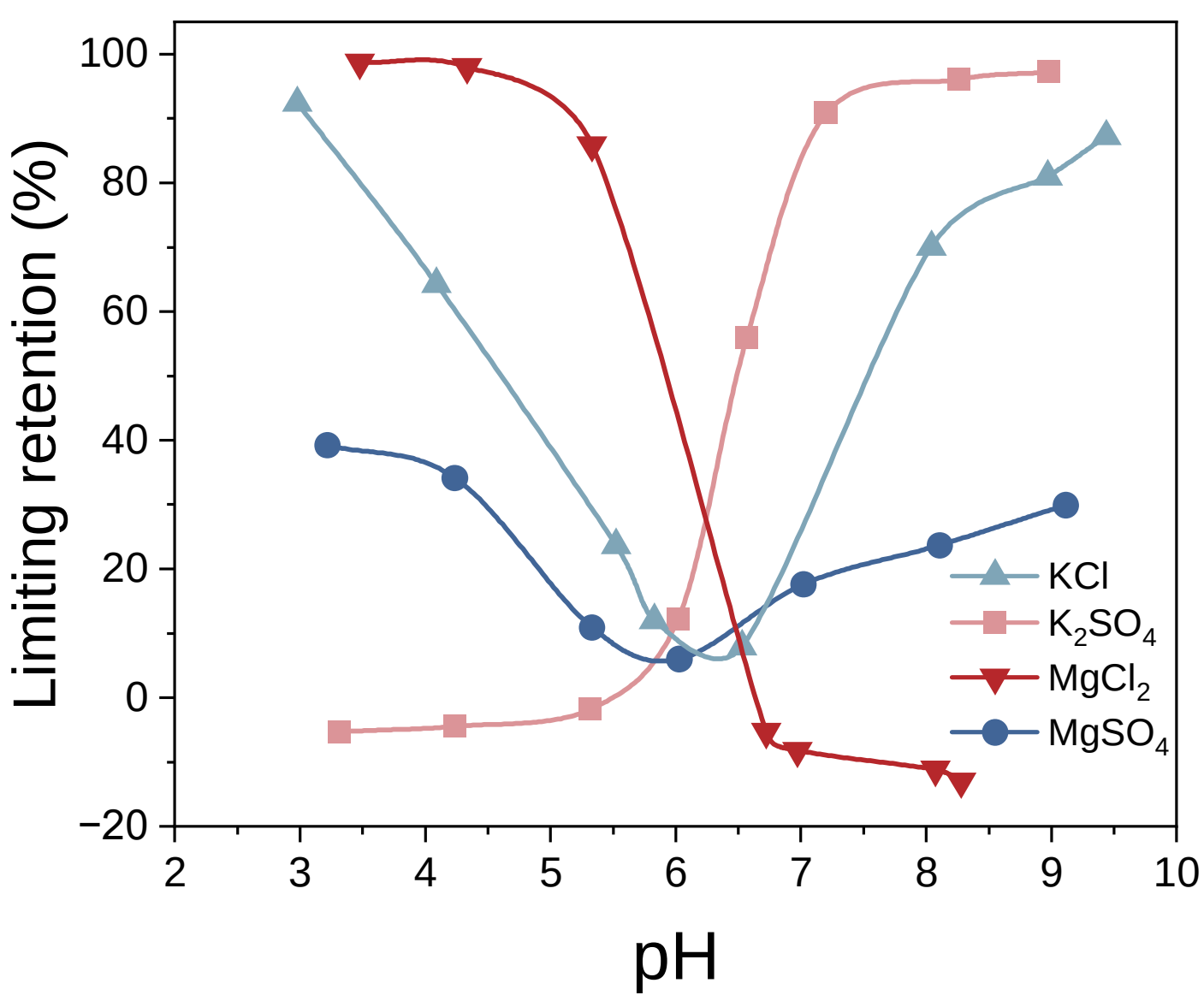

Fig. R1 Limiting retention of single solutions as a function of pH at fixed concentration of 1 mmol L^{-1}. Figure adapted from [6, 7].

In summary, the pore size indeed affects the dissociation of $(H_2O)_nH^+$, but the polyhydroxy quaternary ammonium structure enables this process to occur more energetic-favorably, producing higher proportion of smaller water clusters,

lowering the energy barrier and reducing resistance, thereby enhancing water flux. The main reasons for the decrease in salt rejection of the QBTS membrane are its more negative membrane surface zeta potential and the stronger affinity of the polyhydroxy structure for cations rather than size effect, which lead to a higher partitioning coefficient and greater permeability for ions. We have made corresponding revisions to the manuscript to address the reviewer's concerns.

MS-Line 505 We thus hypothesize that the steric hindrance of the confined nanochannels and abundant carboxyl functional groups in the polyamide active layer may contribute to the dissociation of water clusters as we mentioned earlier (Fig. 1c). Previous studies have also shown that water clusters are transported through polyamide nanochannel in a smaller form compared to that in the bulk phase.

MS-Line 516 Therefore, we can deduce that the hydration competition at the active sites of QBTS, i.e., -OH and $-R_3N^+$ might further promote the water clusters dissociation, facilitating ~~more favorable transmembrane transport of water molecules.~~ the formation of more small-sized water cluster species, thereby further promoting transmembrane water transport.

MS-Line 423 Simultaneously, we observed that QBTS-1 slightly reduced the retention of the four salts, particularly for salts containing monovalent Cl^-, i.e., $MgCl_2$, CaCl, LiCl and NaCl, which is reasonably attributed to that -OH and $-R_3N^+$ on the membrane surface breaking the hydrogen bonds within the water molecule clusters while assisting in the dehydration of hydrated ions and facilitating the transport of ions. We thus used molecular dynamics to investigate ion rejection behavior in simulated polyamide nanochannels (details in *Supplementary information*). As shown in Fig. 4e and 4f, we calculated the potential of mean force (PMF) and diffusion coefficients of Cl^- along the diffusion coordinate in PIP TMC and QBTS simulated nanochannels. Unexpectedly, the PMF of Cl^- in QBTS was lower, decreasing from 20.75 to 16.39 kcal mol-1 (Fig. 4f). Meanwhile, as shown in Fig. 4e, during transport through the QBTS pore, Cl^- exhibited several favorable transport regions, such as near the pore entrance (~90 Å), showing higher diffusion coefficients. Using the inhomogeneous solubility-diffusion model, the calculated permeability coefficient across the entire membrane increased correspondingly from 1.10×10^{-13} to 1.20×10^{-10} cm s^{-1}, indicating that Cl^- is more easily transported in QBTS. This phenomenon seems counterintuitive for QBTS, which has a more negative zeta potential. However, similar observations have been reported in many nanopores with -OH-modified entrances. Based these previous studies, we speculate two possible reasons for the reduction of Cl^- rejection: i) the polyhydroxy structure in QBTS can interact with Cl^- through a "dehydration compensation" mechanism, replacing some of the bound water lost from the hydrated Cl^-, thereby stabilizing the dehydration process of Cl^- and lowering the dehydration energy barrier; ii) the quaternary-N^+ near the pore entrance may help direct Cl^- toward the pore by electrostatic adsorption, making Cl^- transport more favorable. In addition, the polyhydroxy quaternary structure of QBTS can complex with cations, particularly Mg^{2+}, further enhancing the affinity between the cations and the membrane. These Mg^{2+} adsorbed onto the membrane surface further induce charge neutralization, thereby weakening the repulsion of the membrane surface negative charge against co-ions. These effects together result in a more pronounced decrease in rejection of $MgCl_2$, NaCl, LiCl, and $CaCl_2$ after QBTS modification.

References:

1. Li Wang JH, Mohammad Heiranian, Hanqing Fan, Lianfa Song, Ying Li, Menachem Elimelech. Water transport in reverse osmosis membranes is governed by pore flow, not a solution-diffusion mechanism. *Science advances* **9**, eadf8488 (2023).

2. Lu C, *et al.* In Situ Characterization of Dehydration during Ion Transport in Polymeric Nanochannels. *J Am Chem Soc* **143**, 14242-14252 (2021).

3. Lu C, *et al.* Dehydration-enhanced ion-pore interactions dominate anion transport and selectivity in nanochannels. **9**, eadf8412 (2023).

4. Shefer I, Lopez K, Straub AP, Epsztein R. Applying transition-state theory to explore transport and selectivity in salt-rejecting membranes: a critical review. *Environmental Science & Technology*, (2022).

5. Wijmans JG, Baker RW. The Solution-Diffusion Model - a Review. *Journal of Membrane Science* **107**, 1-21 (1995).

6. Luo J, Wan Y. Effects of pH and salt on nanofiltration—a critical review. *Journal of Membrane Science* **438**, 18-28 (2013).

7. Szabolcs Szoke GP, Laszlo Weiser. Characteristics of thin-film nanofiltration membranes at various pH-values. *Desalination* **151**, 123-129 (2002).

8. Al-Sogair FM, Operschall BP, Sigel A, Sigel H, Schnabl J, Sigel RKOJCr. Probing the metal-ion-binding strength of the hydroxyl group. **111 8**, 4964-5003 (2011).

9. Gyurcsik B, Nagy L. Carbohydrates as ligands: coordination equilibria and structure of the metal complexes. *Coordination Chemistry Reviews* **203**, 81-149 (2000).

10. Allscher T, Klüfers P, Mayer P. Carbohydrate-Metal ComplexesCarbohydrate–metal complexes: Structural Chemistry of Stable Solution Species. In: *Glycoscience: Chemistry and Chemical Biology* (eds Fraser-Reid BO, Tatsuta K, Thiem J). Springer Berlin Heidelberg (2008).

11. Tajmir-Riahi HA. Magnesium-sugar interaction: Synthesis, spectroscopic and structural characterization of Mg-sugar complexes containing β-d-fructose. *Biophysical Chemistry* **23**, 223-228 (1986).

12. Schaep J, Vandecasteele C, Mohammad AW, Bowen WR. Modelling the retention of ionic components for different nanofiltration membranes. *Separation and Purification Technology* **22-3**, 169-179 (2001).

13. Guo H-y, Gao X-q, Yu K-c, Wang X-m, Liu S-m. Ion adsorption on nanofiltration membrane surface and its effect on rejection of charged solutes: A zeta potential approach. *Separation and Purification Technology* **326**, 124830 (2023).

14. Roth RS, Birnhack L, Avidar M, Hjelvik EA, Straub AP, Epsztein R. Effect of solution ions on the charge and performance of nanofiltration membranes. *npj Clean Water* **7**, (2024).

Reviewer (Comment 11): *In Lines 338-341, it is proposed that "the poorest $MgCl_2$ rejection performance of QBTS-1 is due to hydrogen bond destruction", but this cannot explain "why the $MgSO_4$ rejection performance remains good"*

Our Response: We thank the reviewer for the valuable comment. Indeed, attributing the lowest rejection of $MgCl_2$ solely to "H-bond disruption" is not sufficiently comprehensive and detailed. According to the widely accepted nanofiltration separation mechanism[1, 2, 3, 4], salt rejection is typically determined by the ion with lower permeability[5, 6]. Factors affecting ion permeability include membrane surface charge (Donnan effect)[6], membrane pore size (size sieving)[6], and ion-membrane interactions (adsorption)[7, 8], among others. As shown in Fig. R1, previous studies have demonstrated that the rejection of different salt types responds distinctly to membrane surface charge [1, 5]. For $MgSO_4$, rejection is typically governed by the co-ion SO_4^{2-}. Since QBTS maintains a relatively negative Zeta potential (Fig. 3j) and SO_4^{2-} show relatively larger ion size and higher charge, the $MgSO_4$ rejection of QBTS is well preserved.

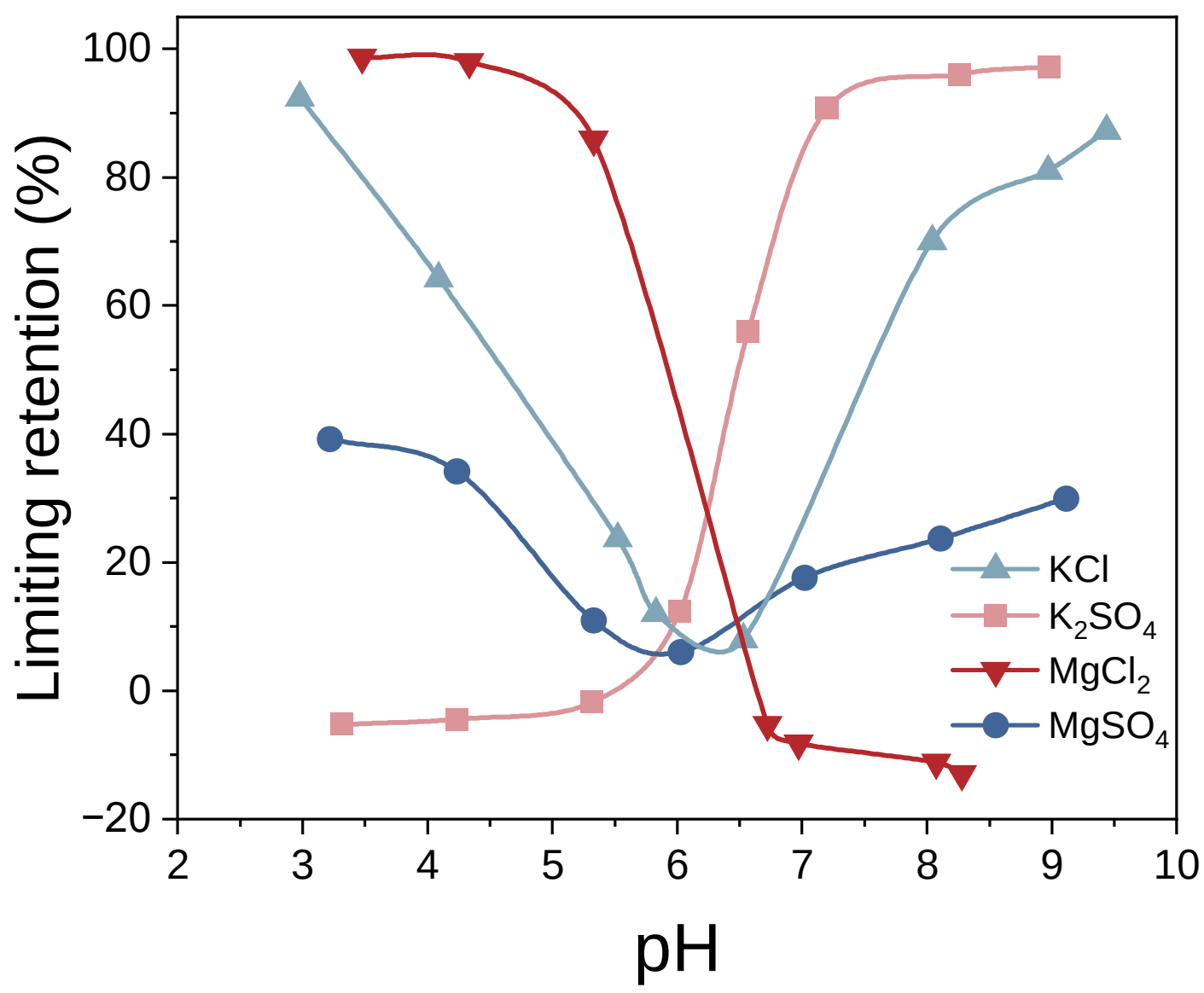

Fig. R1 Limiting retention of single solutions as a function of pH at fixed concentration of 1 mmol L^{-1}. Figure adapted from [1, 5].

For $MgCl_2$, the salt rejection is determined by the Mg^{2+}. Since the pore size distributions of QBTS-1 and PIP-TMC were almost identical (Fig. 3d), we speculate that this reduction in salt rejection originates from the unique surface characteristics of QBTS. First, the lower Zeta potential of QBTS facilitates the adsorption of cations onto the membrane surface (Fig. 3j). Second, the polyhydroxy quaternary ammonium structure can complex with cations[9, 10, 11, 12], particularly Mg^{2+}, further enhancing the affinity between the cations and the membrane. These Mg^{2+} ions adsorbed onto the membrane surface further induce charge neutralization, thereby weakening the repulsion of the membrane surface negative charge against co-ions[7,

[8, 13].

Finally, according to the suggestion of the reviewer, we took Cl^- as an example and used molecular dynamics to investigate ion rejection behavior (Details in Comment 13). As shown in Fig. 4f, the PMF of Cl^- in QBTS was lower, decreasing from 20.75 to 16.39 kcal mol^{-1} (Fig.4f). Meanwhile, as shown in Fig. 4e, during transport through the QBTS pore, Cl^- exhibited several favorable transport regions, such as near the pore entrance (~90 Å) and at the pore center (~60 Å), showing higher diffusion coefficients. The calculated permeability coefficient across the entire membrane increased correspondingly from 1.10×10^{-13} to 1.20×10^{-10} cm s^{-1}, indicating that Cl^- is more easily transported in QBTS. Based these previous studies, we speculate two possible reasons for the reduction of Cl^- rejection: i) the polyhydroxy structure in QBTS can interact with Cl^- through a "dehydration compensation" mechanism[14, 15, 16], replacing some of the bound water lost from the hydrated Cl^-, thereby stabilizing the dehydration process of Cl^- and lowering the dehydration energy barrier; ii) the quaternary-N^+ near the pore entrance may help direct Cl^- toward the pore by electrostatic adsorption, making Cl^- transport more favorable.

Overall, these effects from surface characteristic of QBTS membrane synergistically enhance salt partitioning and thus the permeability, leading to a decrease in salt rejection. Compared with SO_4^{2-}, Cl^- has a smaller hydrated size and less negative charge. Therefore, $MgCl_2$ and NaCl are more susceptible to the aforementioned factors, resulting in a lower rejection. We have also revised the relevant state to better clarify the reasons for the change in QBTS rejection.

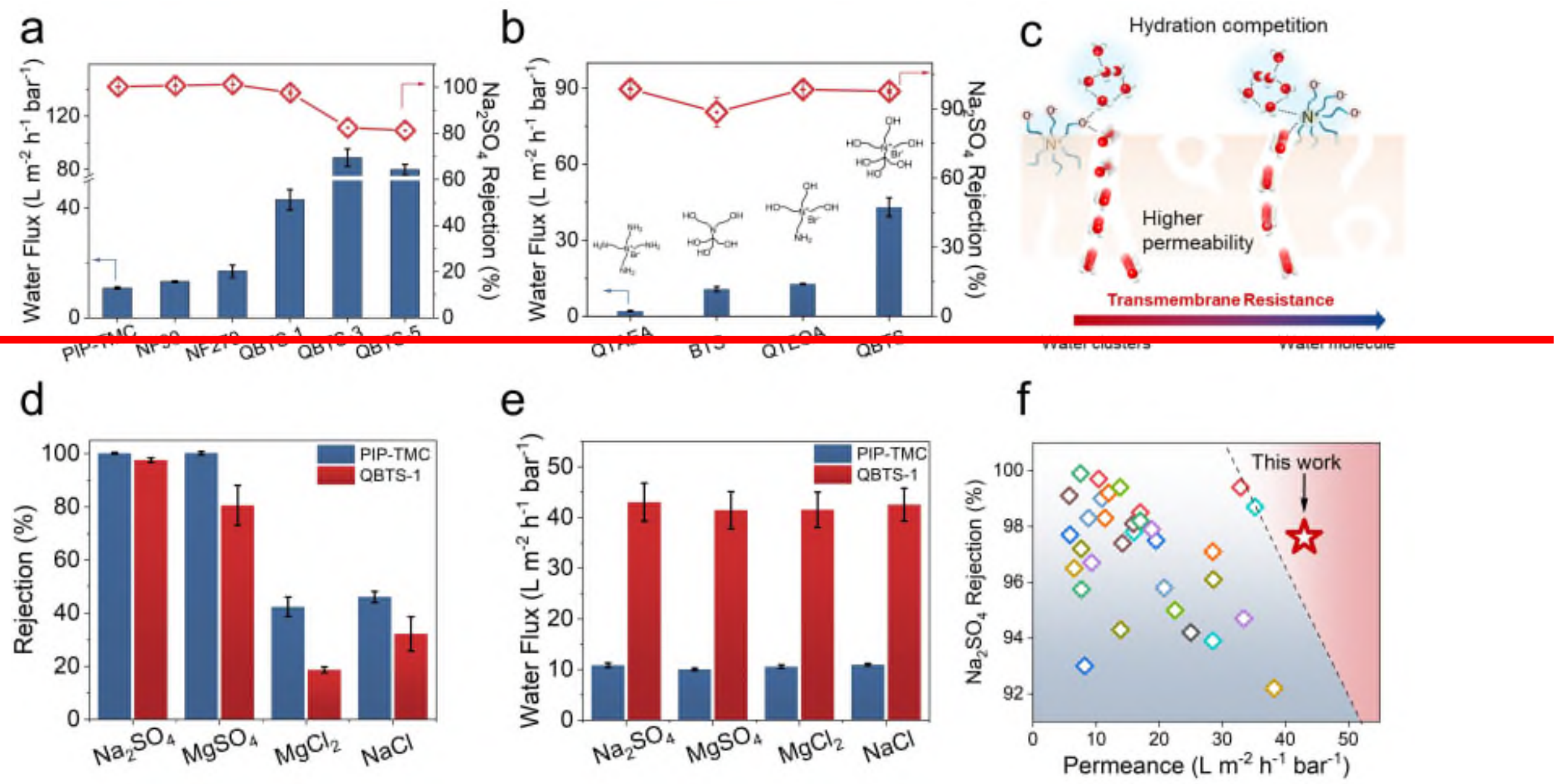

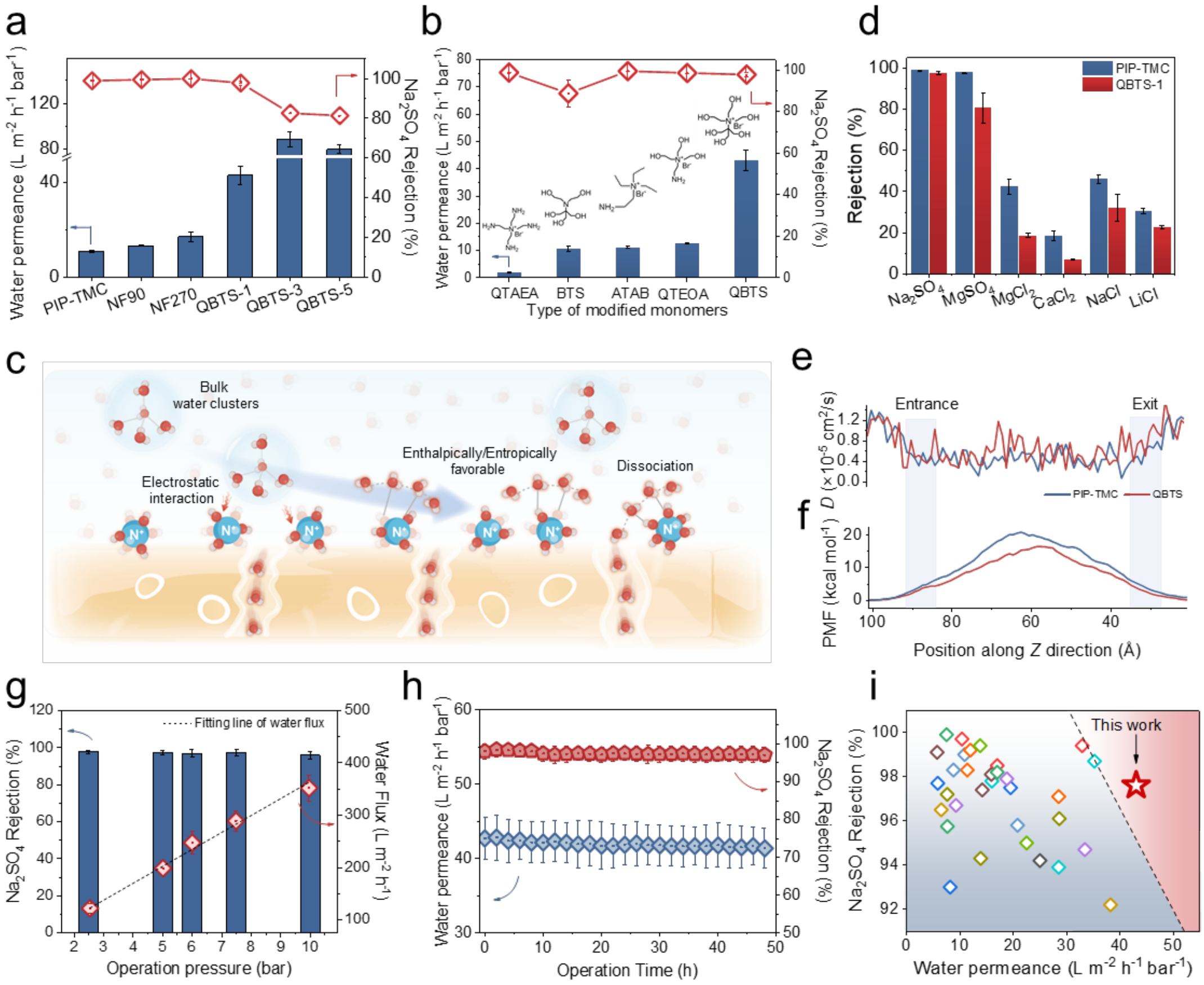

Fig. 4 Filtration performance of QBTS functionalized NF membranes. (a) Effect of QBTS monomer concentration on the separation performance of NF membranes (feed: 1000 ppm Na_2SO_4, 6 bar pressure). (b) Filtration performance of NF membranes modified with different ~~amine~~ monomers (feed: 1000 ppm Na_2SO_4, 6 bar pressure). (c) Schematic diagram of the suppositional mechanism of functional groups promoting the dissociation of water clusters. The dashed lines between water molecules indicate that the original water-water hydrogen bonds are weakened by the polyhydroxy structure of QBTS. (d) Salts rejection ~~and (e) water flux~~ of Na_2SO_4, $MgSO_4$, $MgCl_2$, $CaCl_2$, LiCl and NaCl by PIP-TMC and QBTS-1 membranes (Salt concentration, 1000 ppm; applied pressure, 6 bar~~; temperature, 25 °C~~). (e) The potential of mean force (PMF) and (f) diffusion coefficients (*D*) of Cl^- along the diffusion coordinate in PIP TMC and QBTS simulated nanochannels. The filtration performance of QBTS-1 under different (g) operation pressure and (h) operation Time (feed: 1000 ppm Na_2SO_4, 6 bar pressure). ~~(f)~~ (i) Performance comparison of QBTS-1 membrane with the state-of-the-art NF membranes using surface modification in reported literature (details in Table. S2).

MS-Line 423 Simultaneously, we observed that QBTS-1 slightly reduced the retention of the four salts, particularly for salts containing monovalent Cl^-, i.e., $MgCl_2$, CaCl, LiCl and NaCl, which is reasonably attributed to that -OH and -R_3N^+ on the membrane surface breaking the hydrogen bonds within the water molecule clusters while assisting in the dehydration of hydrated ions and facilitating the transport of ions. We thus used molecular dynamics to investigate ion rejection behavior in simulated polyamide nanochannels (details in *Supplementary information*). As shown in Fig. 4e and 4f, we calculated the potential of mean force (PMF) and diffusion coefficients of Cl^- along the diffusion coordinate in PIP TMC and QBTS simulated nanochannels. Unexpectedly, the PMF of Cl^- in QBTS was lower, decreasing from 20.75 to 16.39 kcal mol-1 (Fig. 4f). Meanwhile, as shown in Fig. 4e, during transport through the QBTS pore, Cl^- exhibited several favorable transport regions, such as near the pore entrance (~90 Å), showing higher diffusion coefficients. Using the inhomogeneous solubility-diffusion model, the calculated permeability coefficient across the entire membrane increased correspondingly from 1.10×10^{-13} to 1.20×10^{-10} cm s^{-1}, indicating that Cl^- is more easily transported in QBTS. This phenomenon seems counterintuitive for QBTS, which has a more negative zeta potential. However, similar observations have been reported in many nanopores with -OH-modified entrances. Based these previous studies, we speculate two possible reasons for the reduction of Cl^- rejection: i) the polyhydroxy structure in QBTS can interact with Cl^- through a "dehydration compensation" mechanism, replacing some of the bound water lost from the hydrated Cl^-, thereby stabilizing the dehydration process of Cl^- and lowering the dehydration energy barrier; ii) the quaternary-N^+ near the pore entrance may help direct Cl^- toward the pore by electrostatic adsorption, making Cl^- transport more favorable. In addition, the polyhydroxy quaternary structure of QBTS can complex with cations, particularly Mg^{2+}, further enhancing the affinity between the cations and the membrane. These Mg^{2+} adsorbed onto the membrane surface further induce charge neutralization, thereby weakening the repulsion of the membrane surface negative charge against co-ions. These effects together result in a more pronounced decrease in rejection of $MgCl_2$, NaCl, LiCl, and $CaCl_2$ after QBTS modification.

References:

1. Luo J, Wan Y. Effects of pH and salt on nanofiltration—a critical review. *Journal of Membrane Science* **438**, 18-28 (2013).

2. Shefer I, Lopez K, Straub AP, Epsztein R. Applying transition-state theory to explore transport and selectivity in salt-rejecting membranes: a critical review. *Environmental Science & Technology*, (2022).

3. Wijmans JG, Baker RW. The Solution-Diffusion Model - a Review. *Journal of Membrane Science* **107**, 1-21 (1995).

4. Epsztein R, Shaulsky E, Dizge N, Warsinger DM, Elimelech M. Role of ionic charge density in donnan exclusion of monovalent anions by nanofiltration. *Environmental Science & Technology* **52**, 4108-4116 (2018).

5. Szabolcs Szoke GP, Laszlo Weiser. Characteristics of thin-film nanofiltration membranes at various pH-values. *Desalination* **151**, 123-129 (2002).

6. Cevallos-Cueva N, Rahman MM, Hailu Kinfu H, Abetz V. Mass transport mechanism of nitrate selective nanofiltration membranes on the basis of the Donnan steric pore model with dielectric exclusion (DSPM-DE). *Chemical Engineering Journal* **493**, (2024).

7. Guo H-y, Gao X-q, Yu K-c, Wang X-m, Liu S-m. Ion adsorption on nanofiltration membrane surface and its effect on rejection of charged solutes: A zeta potential approach. *Separation and Purification Technology* **326**, 124830 (2023).

8. Roth RS, Birnhack L, Avidar M, Hjelvik EA, Straub AP, Epsztein R. Effect of solution ions on the charge and performance of nanofiltration membranes. *npj Clean Water* **7**, (2024).

9. Al-Sogair FM, Operschall BP, Sigel A, Sigel H, Schnabl J, Sigel RKOJCr. Probing the metal-ion-binding strength of the hydroxyl group. **111 8**, 4964-5003 (2011).

10. Gyurcsik B, Nagy L. Carbohydrates as ligands: coordination equilibria and structure of the metal complexes. *Coordination Chemistry Reviews* **203**, 81-149 (2000).

11. Allscher T, Klüfers P, Mayer P. Carbohydrate-Metal ComplexesCarbohydrate–metal complexes: Structural Chemistry of Stable Solution Species. In: *Glycoscience: Chemistry and Chemical Biology* (eds Fraser-Reid BO, Tatsuta K, Thiem J). Springer Berlin Heidelberg (2008).

12. Tajmir-Riahi HA. Magnesium-sugar interaction: Synthesis, spectroscopic and structural characterization of Mg-sugar complexes containing β-d-fructose. *Biophysical Chemistry* **23**, 223-228 (1986).

13. Schaep J, Vandecasteele C, Mohammad AW, Bowen WR. Modelling the retention of ionic components for different nanofiltration membranes. *Separation and Purification Technology* **22-3**, 169-179 (2001).

14. Cohen-Tanugi D, Grossman JC. Water Desalination across Nanoporous Graphene. *Nano Letters* **12**, 3602-3608 (2012).

15. Wang M, *et al.* A coupled effect of dehydration and electrostatic interactions on selective ion transport through charged nanochannels. *Nanoscale* **10**, 18821-18828 (2018).

16. He Z, Zhou J, Lu X, Corry B. Bioinspired graphene nanopores with voltage-tunable ion selectivity for Na^+ and K^+. *ACS Nano* **7**, 10148-10157 (2013).

Reviewer (Comment 12): *In the experiments, solutions containing Na_2SO_4, $MgCl_2$, etc., are used as research objects. The hydration of ions will form "hydration competition" with the functional groups on the membrane surface (e.g., the coordination between ions and water molecules may weaken the dissociation effect of functional groups on water clusters). However, the existing simulations do not include the ion system.*

Our Response: We thank the reviewer for the insightful comment. First, we would like to clarify that to maintain charge neutrality in the simulation system and to mimic the actual nanofiltration process in our study, we randomly placed different ions in the water box, as detailed below: The aqueous solution in PIP-TMC contained 15 Na^+, 1 Cl^-, 1 SO_4^{2-}, and 4329 H_2O molecules. The aqueous phase in QBTS included 15 Na^+, 1 Cl^-, 7 Br^-, 1 SO_4^{2-}, and 4218 H_2O molecules. Meanwhile, some of the carboxyl and hydroxyl groups in PIP-TMC and QBTS were ionized to ensure charge neutrality of the system.

It can be seen that in the presence of coexisting ions, we can still observe the effect of QBTS on the water cluster structure—on the one hand, it reduces the number of H-bonds in water clusters near the pore entrance (Fig. 5f); on the other hand, it promotes the transport speed of water molecules near the pore entrance (Fig. 5g). Furthermore, as shown in Fig. S15, we observed that the water permeance of QBTS did not change significantly in different salt solutions. This indicates that in the presence of competing coexisting salt ions, QBTS can still maintain excellent water permeance. Although, as the reviewer noted, ions can also interact with the polyhydroxy quaternary ammonium structure of QBTS, in our study the salt concentration was 1000 ppm. That is, the molar concentration of the salt is ~0.007 M, which is very low compared with that of water (~55.5 M), so this “competition” does not significantly affect the water flux. We have made the following revisions to address the reviewer’s concern.

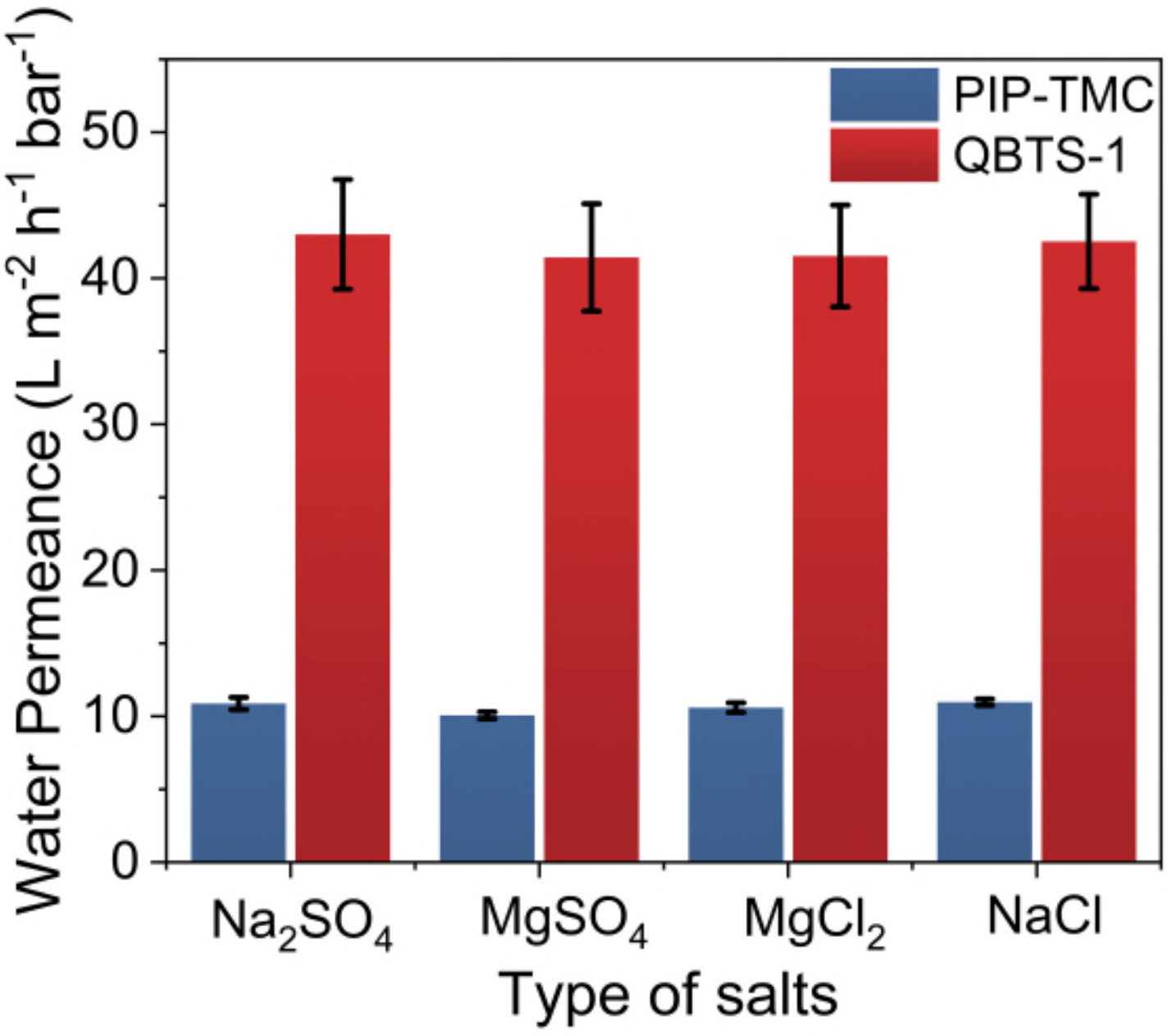

Fig S15. Water permeance of Na_2SO_4, $MgSO_4$, $MgCl_2$, and NaCl by PIP-TMC and QBTS-1 membranes (Salt concentration, 1000 ppm; applied pressure, 6 bar; temperature, 25 °C).

SI-Line 355 In PIP-TMC, the top surface of the membrane is ionized by replacing the exposed -COOH group with a $-COO^-$ group. In QBTS, the top surface of the membrane was first grafted with seven QBTS molecules by esterification. To maintain charge neutrality in the simulation system and to mimic the actual nanofiltration process in our study, we randomly placed different ions in the water box, as detailed below: the aqueous solution in PIP-TMC contained 15 Na^+, 1 Cl^-, 1 SO_4^{2-}, and 4329 H_2O molecules. The aqueous phase in QBTS included 15 Na^+, 1 Cl^-, 7 Br^-, 1 SO_4^{2-}, and 4218 H_2O molecules. Meanwhile, some of the carboxyl and hydroxyl groups in PIP-TMC and QBTS were ionized to ensure charge neutrality of the system.

MS-Line 453 Meanwhile, QBTS-1 demonstrated satisfactory flux of other salts ~~(Fig. 4e)~~ (Fig. S15). This indicates that in the presence of competing coexisting salt ions, QBTS can still maintain excellent water permeance.

<u>Reviewer (Comment 13)</u>: *It is suggested to analyze the ion rejection behavior through molecular dynamics simulations and add simulation details, such as the statistical range inside or at the entrance of the pores.*

Our Response: We thank the reviewer for the valuable suggestion. We would like to clarify that our study focused more on the water transport mechanism, and thus the analysis of ion rejection behavior was previously less emphasized.

As mentioned in Comment 11, according to the widely accepted nanofiltration separation mechanism[1, 2, 3, 4], salt rejection is typically determined by the ion with lower permeability[5, 6]. Factors affecting ion permeability include membrane surface charge (Donnan effect)[6], membrane pore size (size sieving)[6], and ion-membrane interactions (adsorption)[7, 8], among others. For negatively charged membranes in our study, salt rejection is in most cases governed by co-ions, such as SO_4^{2-} and Cl^-. Since SO_4^{2-} has a larger hydrated size and higher charge density, its contribution to rejection is usually dominant; therefore, we observed that Na_2SO_4 and $MgSO_4$ retained good rejection. In contrast, the rejection of $MgCl_2$ and NaCl changed more significantly after modification. We additionally investigated the rejection performance of PIP-TMC and QBTS for LiCl and $CaCl_2$, confirming that, like $MgCl_2$ and NaCl, the rejection rates of these two chlorine-containing salts are also significantly affected by QBTS surface modification (Fig. 4d).

Hence, we took Cl^- as an example and used molecular dynamics to investigate ion rejection behavior. As shown in Fig. 4e and 4f, we calculated the diffusion coefficients and potential of mean force (PMF) of Cl^- along the diffusion coordinate in PIP-TMC and QBTS simulated nanochannels. The PMF profiles were reconstructed from the biased umbrella sampling trajectories using the weighted histogram analysis method (WHAM). The position-dependent diffusion coefficients were extracted from the same umbrella sampling production trajectories using the Woolf-Roux method. Based on the more negative zeta potential of QBTS and reports by Peng et al. [9], we ionized a portion of the hydroxyl groups in QBTS.

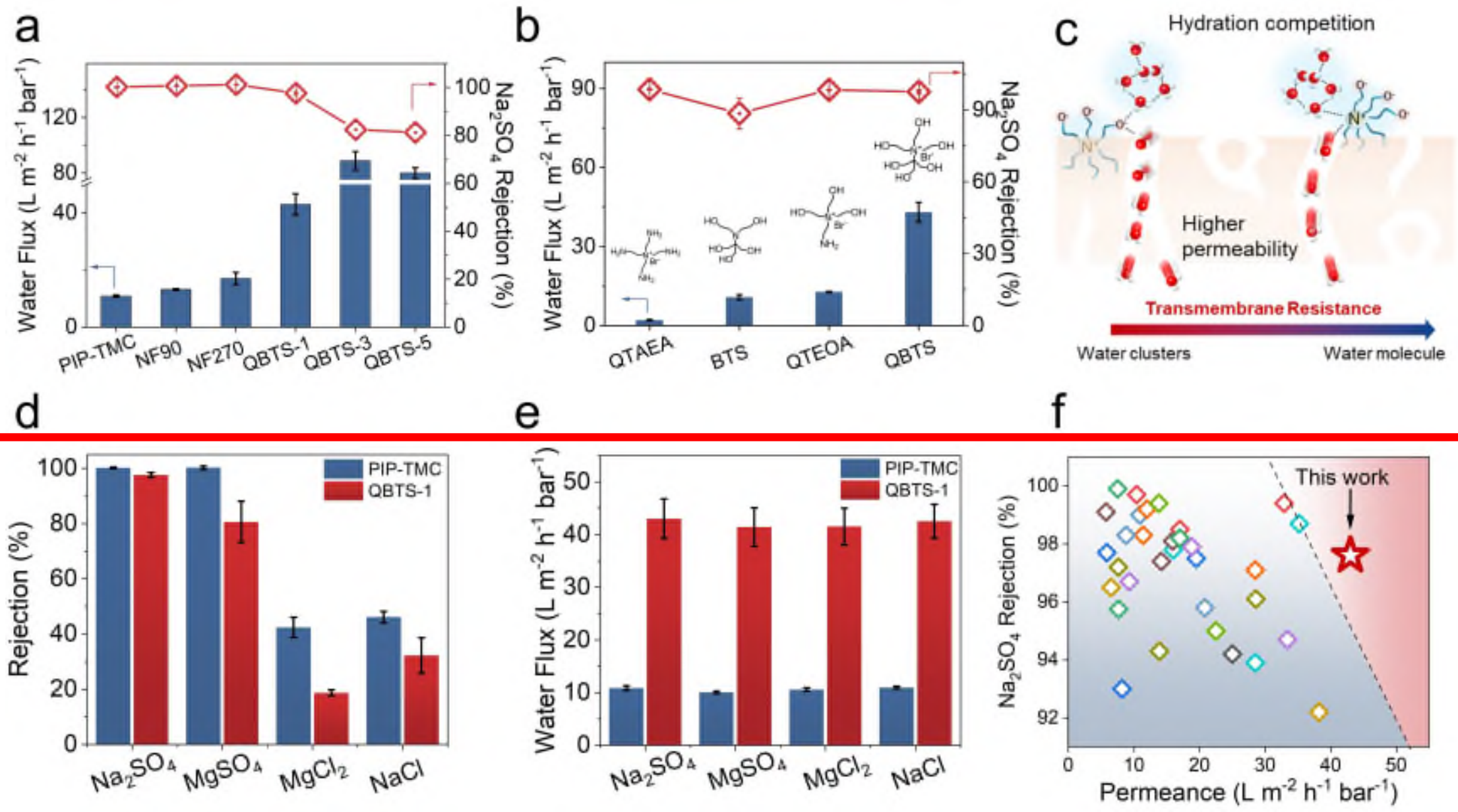

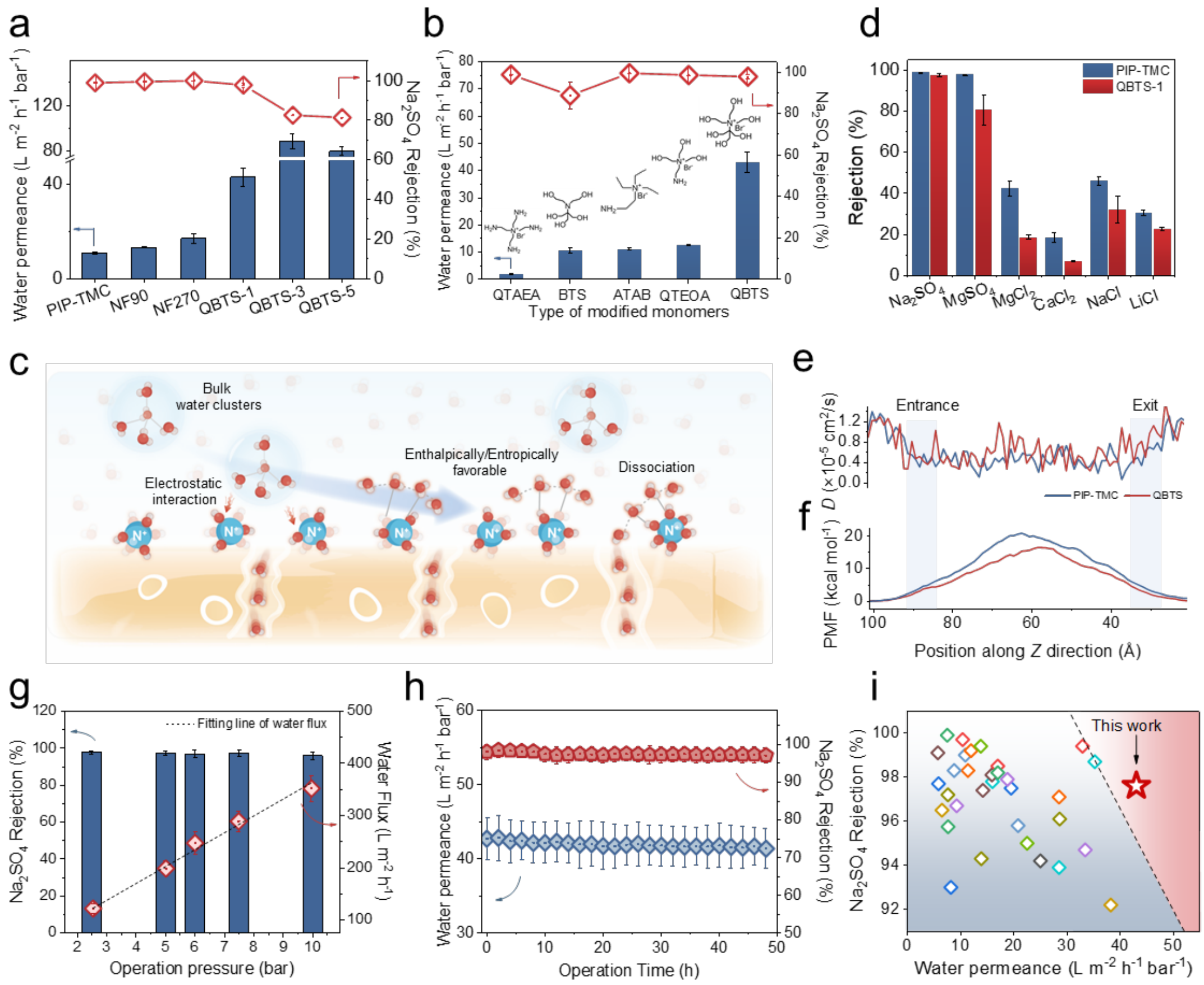

Fig. 4 Filtration performance of QBTS functionalized NF membranes. (a) Effect of QBTS monomer concentration on the separation performance of NF membranes (feed: 1000 ppm Na_2SO_4, 6 bar pressure). (b) Filtration performance of NF membranes modified with different ~~amine~~ monomers (feed: 1000 ppm Na_2SO_4, 6 bar pressure). (c) Schematic diagram of the suppositional mechanism of functional groups promoting the dissociation of water clusters. The dashed lines between water molecules indicate that the original water-water hydrogen bonds are weakened by the polyhydroxy structure of QBTS. (d) Salts rejection ~~and (e) water flux~~ of Na_2SO_4, $MgSO_4$, $MgCl_2$, $CaCl_2$, LiCl and NaCl by PIP-TMC and QBTS-1

membranes (Salt concentration, 1000 ppm; applied pressure, 6 bar~~; temperature, 25 °C~~). (e) The potential of mean force (PMF) and (f) diffusion coefficients (*D*) of Cl^- along the diffusion coordinate in PIP TMC and QBTS simulated nanochannels. The filtration performance of QBTS-1 under different (g) operation pressure and (h) operation Time (feed: 1000 ppm Na_2SO_4, 6 bar pressure). ~~(f)~~ (i) Performance comparison of QBTS-1 membrane with the state-of-the-art NF membranes using surface modification in reported literature (details in Table. S2).

Unexpectedly, the PMF of Cl^- in QBTS was lower, decreasing from 20.75 to 16.39 kcal mol^{-1} (Fig. 4f). Meanwhile, as shown in Fig. 4e, during transport through the QBTS pore, Cl^- exhibited several favorable transport regions, such as near the pore entrance (~90 Å) and at the pore center (~60 Å), showing higher diffusion coefficients. Using the inhomogeneous solubility-diffusion model, the calculated permeability coefficient across the entire membrane increased correspondingly from 1.10×10^{-13} to 1.20×10^{-10} cm s^{-1}, indicating that Cl^- is more easily transported in QBTS. This phenomenon seems counterintuitive for QBTS, which has a more negative zeta potential. However, similar observations have been reported in many nanopores with -OH-modified entrances[10, 11, 12]. Based these previous studies, we speculate two possible reasons for the reduction of Cl^- rejection: i) the polyhydroxy structure in QBTS can interact with Cl^- through a "dehydration compensation" mechanism[10, 13, 14], replacing some of the bound water lost from the hydrated Cl^-, thereby stabilizing the dehydration process of Cl^- and lowering the dehydration energy barrier; ii) the quaternary-N^+ near the pore entrance may help direct Cl^- toward the pore by electrostatic adsorption, making Cl^- transport more favorable. This may also contribute to the lower rejection rates of $MgCl_2$, NaCl, LiCl, and $CaCl_2$.

As mentioned earlier, the polyhydroxy quaternary structure of QBTS can complex with cations[15, 16, 17, 18], particularly Mg^{2+}, further enhancing the affinity between the cations and the membrane. These Mg^{2+} ions adsorbed onto the membrane surface further induce charge neutralization, thereby weakening the repulsion of the membrane surface negative charge against co-ions[7, 8, 19]. These effects explain why $MgCl_2$ and NaCl show a more pronounced decrease in rejection after QBTS modification.

In addition, we have added more simulation details, such as the statistical range inside or at the entrance of the pores. All revisions are as follows:

SI-Line 669 Molecular dynamics (MD) simulation platform of the polyamide nanochannels. Initial configurations showing (a) polyamide simulated nanochannels (PIP-TMC) and (b) QBTS modified polyamide simulated nanochannels. The highly cross-linked and porous polymeric membrane was simplified to a nanochannel composed of polyamide units. The cross-sectional illustration of the simulation platform showing (c) PIP-TMC nanochannel and (d) QBTS modified polyamide simulated nanochannels immersed in containers filled with water molecules to investigate hydration number and water diffusion velocities during transmembrane transport. Colors: O, red; C, green; H, white; N, blue; QBTS, deep red in QBTS grafted model. As shown in Fig. 4e, Fig. 4f, Fig. 5f, Fig. 5g and Fig. S25, we define the entrance of the nanochannels located ~92-80 Å along Z direction. This statistical range was used for analyzing the hydrogen bond/hydration number, diffusion coefficient and PMF.

MS-Line 423 Simultaneously, we observed that QBTS-1 slightly reduced the retention of the four salts, particularly for salts containing monovalent Cl^-, i.e., $MgCl_2$, CaCl, LiCl and NaCl~~, which is reasonably attributed to that -OH and $-R_3N^+$ on the membrane surface breaking the hydrogen bonds within the water molecule clusters while assisting in the dehydration of hydrated ions and facilitating the transport of ions~~. We thus used molecular dynamics to investigate ion rejection behavior in simulated polyamide nanochannels (details in *Supplementary information*). As shown in Fig. 4e and 4f, we calculated the potential of mean force (PMF) and diffusion coefficients of Cl^- along the diffusion coordinate in PIP TMC and QBTS simulated nanochannels. Unexpectedly, the PMF of Cl^- in QBTS was lower, decreasing from 20.75 to 16.39 kcal mol-1 (Fig. 4f). Meanwhile, as shown in Fig. 4e, during transport through the QBTS pore, Cl^- exhibited several favorable transport regions, such as near the pore entrance (~90 Å), showing higher diffusion coefficients. Using the inhomogeneous solubility-diffusion model, the calculated permeability coefficient across the entire membrane increased correspondingly from 1.10×10^{-13} to 1.20×10^{-10} cm s^{-1}, indicating that Cl^- is more easily transported in QBTS. This phenomenon seems counterintuitive for QBTS, which has a more negative zeta potential. However, similar observations have been reported in many nanopores with -OH-modified entrances. Based these previous studies, we speculate two possible reasons for the reduction of Cl^- rejection: i) the polyhydroxy structure in QBTS can interact with Cl^- through a “dehydration compensation” mechanism, replacing some of the bound water lost from the hydrated Cl^-, thereby stabilizing the dehydration process of Cl^- and lowering the dehydration energy barrier; ii) the quaternary-N^+ near the pore entrance may help direct Cl^- toward the pore by electrostatic adsorption, making Cl^- transport more favorable. In addition, the polyhydroxy quaternary structure of QBTS can complex with cations, particularly Mg^{2+}, further enhancing the affinity between the cations and the membrane. These Mg^{2+} adsorbed onto the membrane surface further induce charge neutralization, thereby weakening the repulsion of the membrane surface negative charge against co-ions. These effects together result in a more pronounced decrease in rejection of $MgCl_2$, NaCl, LiCl, and $CaCl_2$ after QBTS modification.

SI-Line 347 ***Confined transport of water molecules in different Nanochannels.*** In order to study the transport behavior of $(H_2O)_nH^+$ and ion in two types of polyamide nanochannels, two simulated nanochannel models were constructed, namely PIP-TMC and QBTS (Fig. S14). It is worth noting that both nanochannels are composed of polyamide units, thus retaining the basic physicochemical characteristics of the polyamide nanofiltration membrane prepared in this experiment. Both models have dimensions of 45.0×45.0×120.0 (X×Y×Z) $Å^3$. In both models, the original layer was stacked from duplicate polyamide units with a thickness of 6.2 nm and embedded in the center of the membrane with a cylindrical nanopore of a radius of about 0.3 nm along the Z-axis. In PIP-TMC, the top surface of the membrane is ionized by replacing the exposed -COOH group with a $-COO^-$ group. In QBTS, the top surface of the membrane was first grafted with seven QBTS molecules by esterification. To maintain charge neutrality in the simulation system and to mimic the actual nanofiltration process in our study, we randomly placed different ions in the water box, as detailed below: the aqueous solution in PIP-TMC contained 15 Na^+, 1 Cl^-, 1 SO_4^{2-}, and 4329 H_2O molecules. The aqueous phase in QBTS included 15 Na^+, 1 Cl^-, 7 Br^-, 1 SO_4^{2-}, and 4218 H_2O molecules. Meanwhile, some of the carboxyl and hydroxyl groups in PIP-TMC and QBTS were ionized to ensure charge neutrality of the system. The species of interest (hydronium hydrate ions or Cl^-) was initially placed above the top surface of the membrane, located at the central axis of the cylindrical nanopore (**Error! Reference**

SI-Line 378 For the umbrella sampling production runs, the ion of interest was released from its fixed position. A total of 86 umbrella sampling windows were deployed along the Z-axis, spanning from z = 102.7 Å to z = 17.7 Å with a uniform spacing of 1.0 Å between adjacent windows, thereby covering the entire membrane thickness and portions of the bulk solution on both sides. For each window, the simulation proceeded in three sequential stages: (i) the ion was first steered to the target position by applying a harmonic biasing potential with a force constant of 5.0 kcal/(mol·$Å^2$) tethered to the nanochannel central axis in the X and Y directions and to the target Z coordinate, and a 30 ps pulling simulation was conducted; (ii) the restraints in the X and Y directions were then released, retaining only the Z-direction harmonic restraint at the same force constant, and a 200 ps relaxation was performed to allow the ion and surrounding solvent molecules to equilibrate at the target Z position; (iii) a 500 ps production trajectory was then collected for data analysis, during which the center-of-mass position of the ion along the Z-axis was recorded every 100 fs. The polyamide membrane atoms were kept fixed throughout all simulations. The same umbrella sampling protocol, including the identical initial and final positions, window spacing, and spring constant, was applied to both hydronium hydrate ions and Cl^- ions in both PIP-TMC and QBTS. The PMF profiles were reconstructed from the biased umbrella sampling trajectories using the weighted histogram analysis method (WHAM).

The position-dependent diffusion coefficients were extracted from the same umbrella sampling production trajectories using the Woolf–Roux method. The membrane permeability coefficient was then determined based on the inhomogeneous solubility–diffusion model. This enables a direct comparison of the transport properties through the two membrane models.

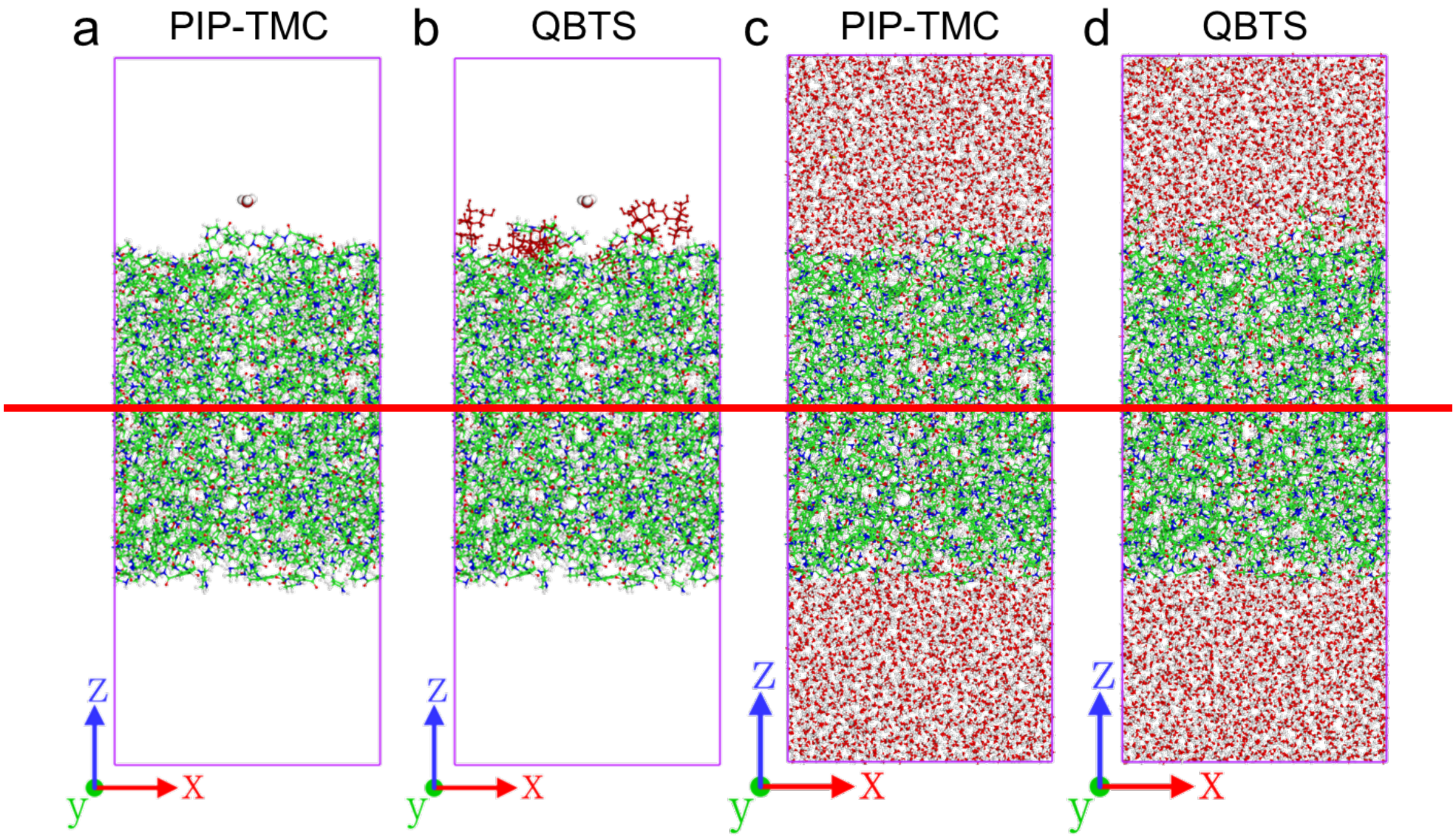

a
PIP-TMC
b
QBTS
c
PIP-TMC
d
QBTS
z
x
y

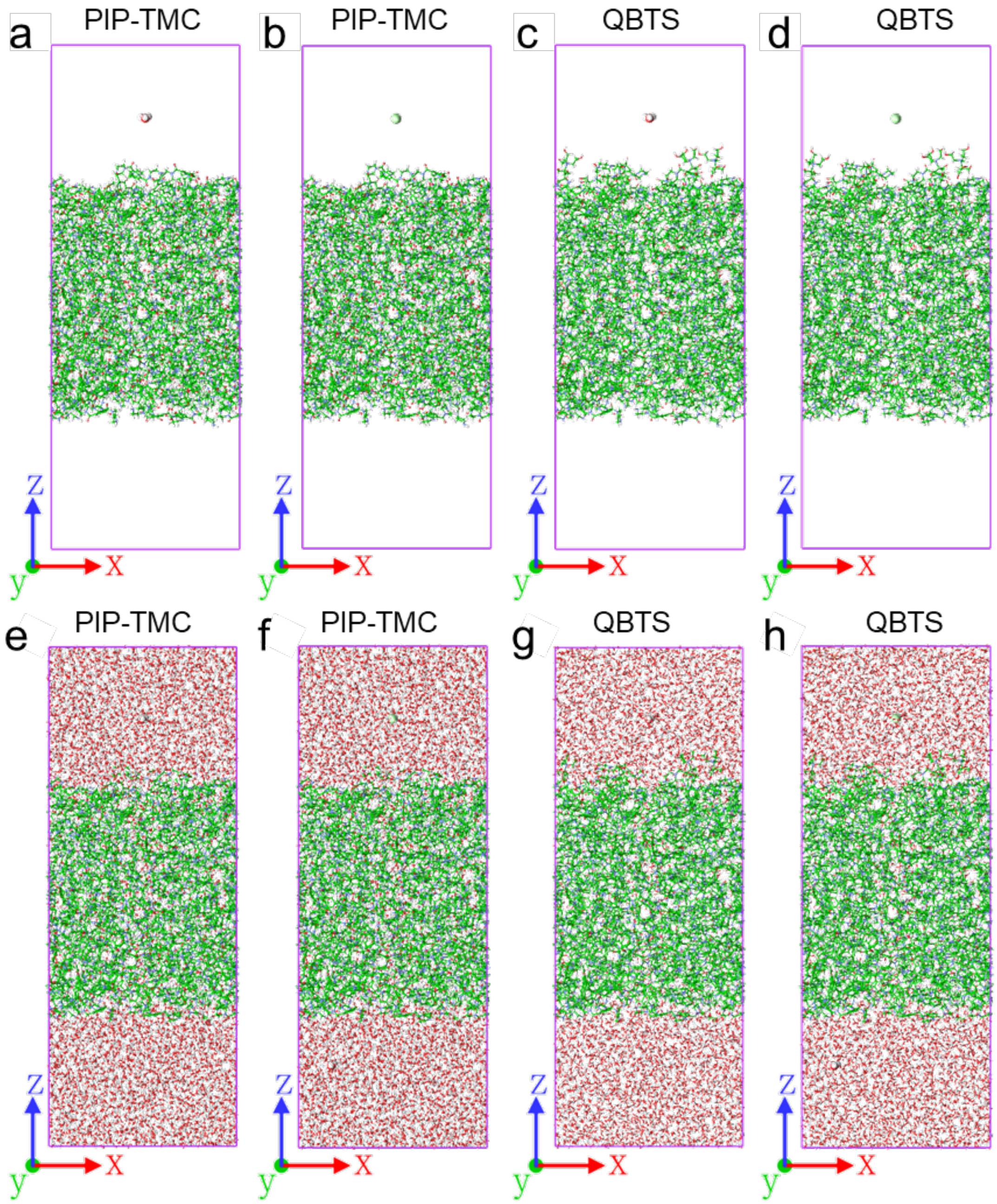

Fig ~~S14~~ S24. Molecular dynamics (MD) simulation platform of the polyamide nanochannels for investigating water and ion transport. Initial configurations showing (a)-(b) polyamide simulated nanochannels (PIP-TMC) for investigating hydronium ion and Cl^-, respectively. ~~and (b)~~(c)-(d) QBTS modified polyamide simulated nanochannels for investigating hydronium ion and Cl^-, respectively. The highly cross-linked and porous polymeric membrane was simplified to a nanochannel composed of polyamide units. The cross-sectional illustration of the simulation platform showing ~~(c)~~(e)-(f) PIP-TMC nanochannel and ~~(d)~~(g)-(h) QBTS modified polyamide simulated nanochannels immersed in containers filled with water molecules to investigate hydration number~~ and ~~, water diffusion velocities, and PMF during transmembrane transport. Colors: O, red; C, green; H, white; N, blue; Cl, green; QBTS~~, deep red in QBTS~~ was grafted ~~model~~ in pore entrance. As

shown in Fig. S25, Fig.5f, Fig. 5g, Fig. 4e and Fig. 4f, we define the entrance of the nanochannels located ~92-80 Å along Z direction. This statistical range was used for analyzing the hydrogen bond/hydration number, diffusion coefficient and PMF.

References:

1. Luo J, Wan Y. Effects of pH and salt on nanofiltration—a critical review. *Journal of Membrane Science* **438**, 18-28 (2013).

2. Shefer I, Lopez K, Straub AP, Epsztein R. Applying transition-state theory to explore transport and selectivity in salt-rejecting membranes: a critical review. *Environmental Science & Technology*, (2022).

3. Wijmans JG, Baker RW. The Solution-Diffusion Model - a Review. *Journal of Membrane Science* **107**, 1-21 (1995).

4. Epsztein R, Shaulsky E, Dizge N, Warsinger DM, Elimelech M. Role of ionic charge density in donnan exclusion of monovalent anions by nanofiltration. *Environmental Science & Technology* **52**, 4108-4116 (2018).

5. Szabolcs Szoke GP, Laszlo Weiser. Characteristics of thin-film nanofiltration membranes at various pH-values. *Desalination* **151**, 123-129 (2002).

6. Cevallos-Cueva N, Rahman MM, Hailu Kinfu H, Abetz V. Mass transport mechanism of nitrate selective nanofiltration membranes on the basis of the Donnan steric pore model with dielectric exclusion (DSPM-DE). *Chemical Engineering Journal* **493**, (2024).

7. Guo H-y, Gao X-q, Yu K-c, Wang X-m, Liu S-m. Ion adsorption on nanofiltration membrane surface and its effect on rejection of charged solutes: A zeta potential approach. *Separation and Purification Technology* **326**, 124830 (2023).

8. Roth RS, Birnhack L, Avidar M, Hjelvik EA, Straub AP, Epsztein R. Effect of solution ions on the charge and performance of nanofiltration membranes. *npj Clean Water* **7**, (2024).

9. Peng H, *et al.* Phosphonium Modification Leads to Ultrapermeable Antibacterial Polyamide Composite Membranes with Unreduced Thickness. *Adv Mater* **32**, e2001383 (2020).

10. Cohen-Tanugi D, Grossman JC. Water Desalination across Nanoporous Graphene. *Nano Letters* **12**, 3602-3608 (2012).

11. Corry B. Water and ion transport through functionalised carbon nanotubes: implications for desalination technology. *Energy & Environmental Science* **4**, (2011).

12. Guerrero-Aviles R, Orellana W. Energetics and diffusion of liquid water and hydrated ions through nanopores in graphene: ab initio molecular dynamics simulation. *Phys Chem Chem*

Phys **19**, 20551-20558 (2017).

13. Wang M*, et al.* A coupled effect of dehydration and electrostatic interactions on selective ion transport through charged nanochannels. *Nanoscale* **10**, 18821-18828 (2018).

14. He Z, Zhou J, Lu X, Corry B. Bioinspired graphene nanopores with voltage-tunable ion selectivity for Na^+ and K^+. *ACS Nano* **7**, 10148-10157 (2013).

15. Al-Sogair FM, Operschall BP, Sigel A, Sigel H, Schnabl J, Sigel RKOJCr. Probing the metal-ion-binding strength of the hydroxyl group. **111 8**, 4964-5003 (2011).

16. Gyurcsik B, Nagy L. Carbohydrates as ligands: coordination equilibria and structure of the metal complexes. *Coordination Chemistry Reviews* **203**, 81-149 (2000).

17. Allscher T, Klüfers P, Mayer P. Carbohydrate-Metal ComplexesCarbohydrate–metal complexes: Structural Chemistry of Stable Solution Species. In: *Glycoscience: Chemistry and Chemical Biology* (eds Fraser-Reid BO, Tatsuta K, Thiem J). Springer Berlin Heidelberg (2008).

18. Tajmir-Riahi HA. Magnesium-sugar interaction: Synthesis, spectroscopic and structural characterization of Mg-sugar complexes containing β-d-fructose. *Biophysical Chemistry* **23**, 223-228 (1986).

19. Schaep J, Vandecasteele C, Mohammad AW, Bowen WR. Modelling the retention of ionic components for different nanofiltration membranes. *Separation and Purification Technology* **22-3**, 169-179 (2001).

Reviewer #3:

<u>Reviewer</u>: *This manuscript presents interesting experimental and computational results on rapid water transport in functionalized NF membranes. The method is scalable, and the combination of experiments, performance tests, and simulation provides useful mechanistic insight and context. I recommend the article be published after several key revisions. I have provided specific comments below that the authors should address:*

Our Response: We thank the reviewer for pointing out the interesting insights of our study. All specific comments/questions have been addressed as below. We would like to kindly remind that in the point-by-point response, we have marked newly added text in blue, deleted text in red with a strikethrough, and placed all revision marks within grey text boxes. In the text, MS-Line refers to the line numbers in the Marked Manuscript, and SI-Line refers to the line numbers in the Marked Supplementary Information.

<u>Reviewer (Comment 1)</u>: *Lines 137-155, Figure 1: The data seem to suggest that water prefers to interact with -R_3N^+, -OH, and -COO^- over itself. I am skeptical of that so this point should be discussed more. If this is indeed the claim, then the authors should comment on and cite what has been observed in prior studies. There must be prior computational studies on this topic.*

Our Response: We thank the reviewer for the insightful comment. As the reviewer pointed out, numerous computational and even experimental studies have confirmed that certain surface functional groups, such as hydroxyl and carboxyl, can preferentially interact with water molecules within water clusters, thereby disrupting or even reconstructing the water cluster structure[1, 2, 3, 4, 5]. Specifically, in bulk water, water molecules tend to form a stable, ordered H-bond network that confines them[6, 7]. When functional groups possess H-bond acceptors/donors that can interact more strongly with water molecules or can grab water molecules in a multi-site manner, they tend to form stronger local interactions with water preferentially, thereby "capture" water molecules, creating oriented anchoring, cutting off or weakening the original water-water H-bond connectivity, reducing the thermodynamic benefit of maintaining the tetrahedral network, and transforming the continuous ordered water network into a more disordered, defect-rich, higher-entropy local structure. Typical examples are as follows:

i) Zhu et al.[4] found that the polyhydroxy structure of pentaerythritol (PTT), with its four symmetric -OH, can form stronger and denser H-bond interactions with water, thereby redistributing the proportions of different types of H-bonds among water molecules. Specifically, the average binding energy between water molecules is approximately -5.07 kcal/mol, while the average binding energy between water

and PPT is approximately -9.69 kcal/mol. Consequently, strong H-bonds (3-H-bond and 4-H-bond) within the water clusters decrease, weak hydrogen bonds (2-H-bond and 1-H-bond) increase, and the average number of hydrogen bonds decreases from about 2.3 to 1.7, indicating that the polyhydroxy structure indeed disrupts the water-water H-bond network.

ii) Gallina et al.[8] used FTIR and Raman spectroscopies to study the polyhydroxy groups on glucose molecules in aqueous solution and found that glucose, on one hand, can form water-glucose H-bonds, and on the other hand, exerts a "destructuring" effect on the ordered tetrahedral H-bond assembly of bulk water. As a result, the ordered tetrahedral/ice-like components within water clusters decrease, the number of defects increases, and the average H-bond strength decreases, indicating that the overall water network becomes looser and more disordered.

iii) Zhou et al.[2] found that the bifunctional sites (C=O and -NH) in caprolactam (CPL) can simultaneously serve as H-bond acceptors and donors, forming a dual-site anchor with a single water molecule. The binding energy of this interaction is stronger than that of water-water hydrogen bonds, thereby competing with and disrupting the original water-water H-bonds networks. As a result, the water clusters are broken apart, and the original continuous H-bond network is significantly suppressed.

iv) Guo et al.[5] investigated the formate groups ($-HCOO^-$) on the $NiFe_2O_4$ surface and found that they can disrupt the rigid H-bond network of the outer Helmholtz plane, promote the reorientation of interfacial water, and increase the proportion of free water. As a result, the interfacial water clusters are transformed from the more stable 4-HB-H_2O to 2-HB-H_2O and free water, while the rigidity and connectivity of the H-bond network decrease, making water dissociation more facile.

v) Wang et al.[9] studied the interfacial hydroxyl groups induced by the polarized Ti-O bonds on the TiO_2 surface. These hydroxyl groups can construct a weak H-bond interface with interfacial water and, with the assistance of the interfacial electric field, further disrupt the original water-water H-bond network. As a result, the proportion of strong H-bonds within water clusters decreases, the overall water activity is reduced, and the network is restructured.

vi) Grossman et al.[10] found that -OH at the edges of porous graphene can pull on water clusters, which increases the cross-sectional area for water cluster transport. Meanwhile, the interaction between the edge -OH groups and water clusters provides more water molecular orientations, reducing the entropic penalty during transmembrane transport and thereby enhancing water flux.

Thus, it has been widely reported across various scenarios—such as electrode interfaces, solution chemistry, nanofluidics, and membrane separation—that water molecules preferentially interact with functional groups like hydroxyl groups rather than to themselves. This process disrupts or even dissociates the water clusters, generating a disordered and loose water network.

Furthermore, using the same calculation method as in Fig. 1c, we calculated the interaction energy between the QBTS monomer and a single water molecule to be -18.3 kcal mol^{-1} (Fig. 2e), which is substantially higher than the reported stabilization energy of water (~4-10 kcal mol^{-1})[4, 6, 7, 11, 12]. Furthermore, the higher abundance of dangling bonds near the functional groups in Fig. 2b also demonstrates that QBTS promotes the formation of "free" water. Based on these literature reports, it is reasonable to infer that QBTS indeed possesses the ability to perturb or even dissociate water cluster structures.

Compared with the destruction of water clusters by extreme confinement, this mechanism is expected to be more favorable for water transport in nanochannels, because the functional group-water interactions can partially compensate the enthalpic loss associated with water-cluster dissociation (Fig 5d)[5, 13], while the increased availability of water molecular orientations helps reduce the entropic penalty during transmembrane transport[10] (Fig. 5c). We have added additional explanatory remarks in the manuscript to provide a more detailed discussion in this section.

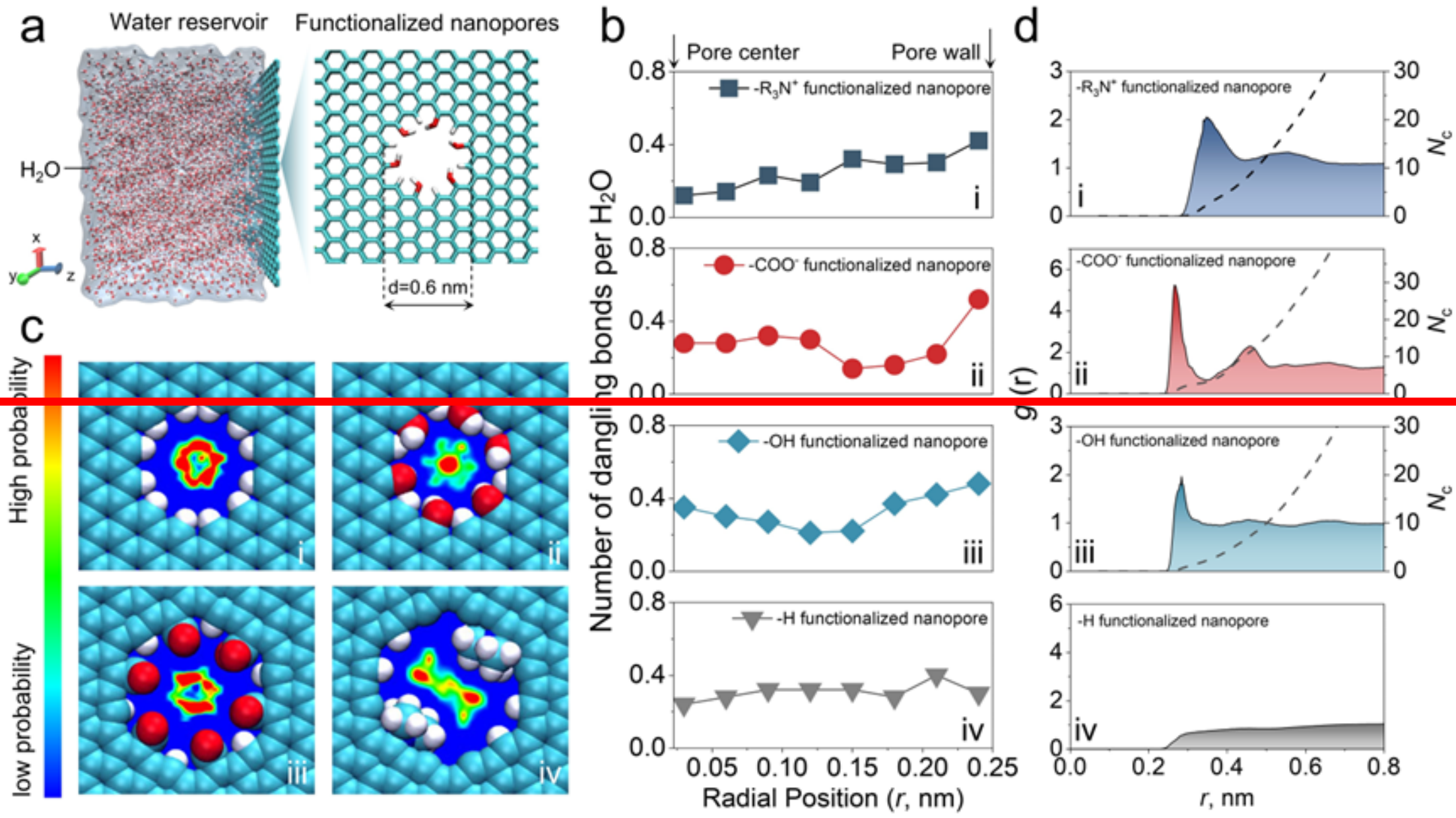

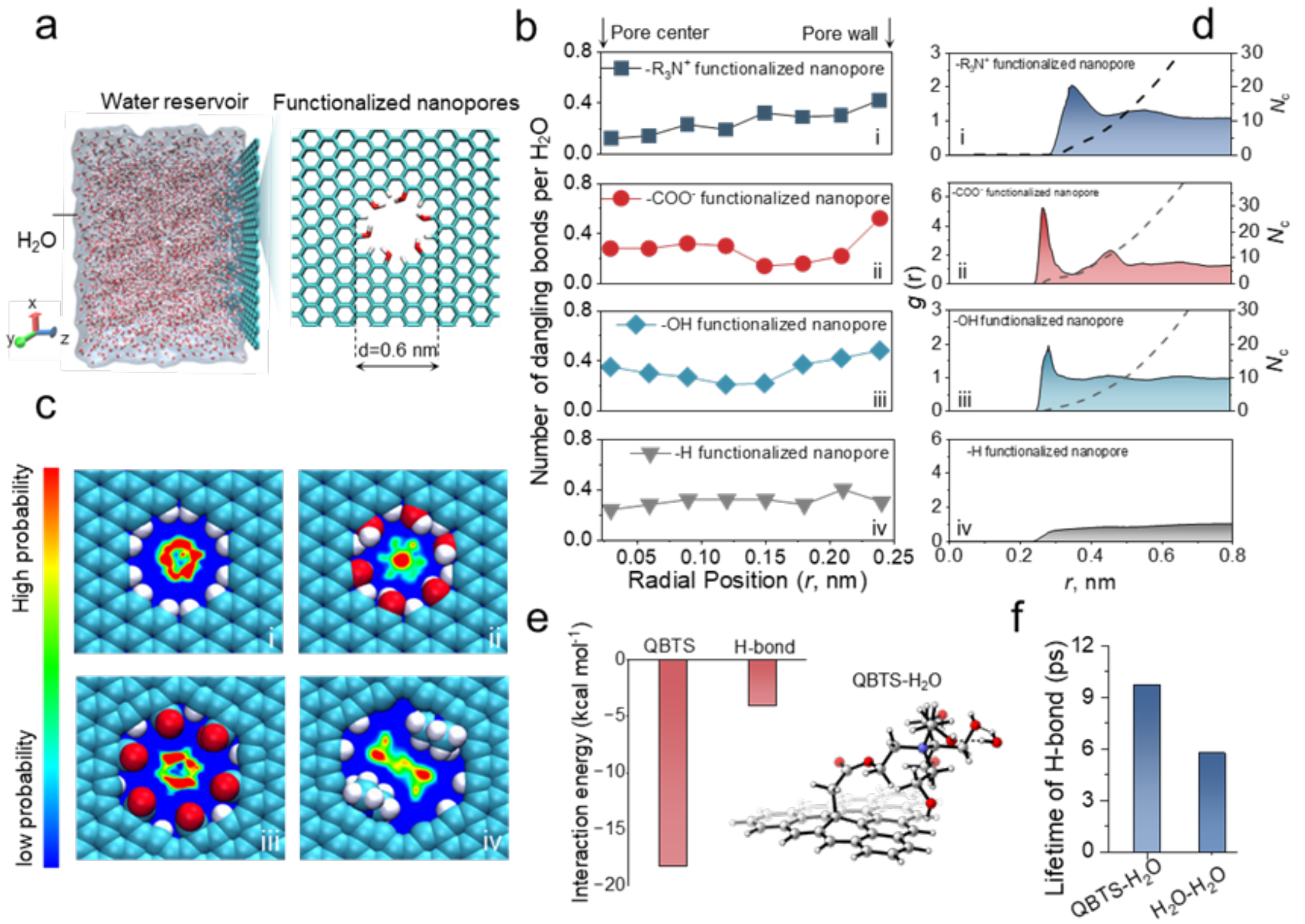

Fig. 2 Mechanisms of water cluster-dissociation from hydration competition by functional groups. (a) Side view of the MD simulation system investigated in this work. (b) Dangling bond radial distribution of water clusters in different functionalized pores. (c) Oxygen density maps in different functionalized pores, i-iv represents -H, -OH, $-COO^-$ and $-R_3N^+$ functionalized pores, respectively. (d) RDFs between water molecule and the functional groups, and the corresponding coordination numbers (N_c), i.e., N-O_w, O-H_w, and H-O_w for $-R_3N^+$, $-COO^-$ and -OH, respectively. (e) The interaction energy between a single water molecule and the QBTS functional group. The inset shows the double-sites interaction between QBTS and water molecule. (f) The H-bond lifetime of QBTS-water and water-water calculating by MD simulation.

MS-Line 135 In contrast, based on the formation mechanism of the water clusters with a hydrogen bonding network, hydration competition between functional groups with H-bonds donor/acceptor and water molecules could be another energy-favorable strategy to achieve dissociation of water clusters. ~~Stronger interactions between water molecules and H-bonds donor/acceptor may lead to the detachment of water molecules from cluster structures, resulting in the dissociation of water clusters.~~ Specifically, according to the previous studies on the disruption of water cluster networks by functional groups such as hydroxyl groups, when functional groups possess H-bond acceptors/donors that can interact more strongly with water molecules, they tend to form stronger local interactions with water preferentially, thereby "capture" water molecules and create oriented anchoring, cutting off or weakening the original water-water H-bond connectivity, reducing the thermodynamic benefit of maintaining the tetrahedral network, and transforming the continuous ordered water network into a smaller and unrestrained local structure. Compared with the destruction of water clusters by extreme confinement, this mechanism is expected to be more favorable for water transport in nanochannels, because the functional group–water interactions can partially compensate the enthalpic loss associated with water-cluster dissociation, while the increased availability of water molecular orientations helps reduce the entropic penalty during transmembrane transport.

MS-Line 211 In summary, a water cluster-breakage near the functionalized pore edges was found due to the hydration competition, which would be beneficial for enlarging the water transport area and reducing the transmembrane resistance. Such disruption/dissociation of water cluster structures, resulting from the stronger interactions between hydroxyl/carboxyl groups and water molecules, has also been reported in various scenarios such as electrode interfaces, solution chemistry, and nanofluidics.

References:

1. Shin S, Willard AP. Water's Interfacial Hydrogen Bonding Structure Reveals the Effective Strength of Surface-Water Interactions. *J Phys Chem B* **122**, 6781-6789 (2018).

2. Zhou L, *et al.* Suppressing Hydrogen Evolution in Aqueous Lithium-Ion Batteries with Double-Site Hydrogen Bonding. *ACS Energy Letters* **8**, 40-47 (2022).

3. Ng LS, *et al.* Chaotropic Nanoelectrocatalysis: Chemically Disrupting Water Intermolecular Network at the Point-of-Catalysis to Boost Green Hydrogen Electrosynthesis. *Angew Chem Int Ed Engl* **63**, e202317751 (2024).

4. Zhu Y, Chen Q, Hao J, Jiao Y. Breaking hydrogen-bonds in aqueous electrolytes towards highly reversible zinc-ion batteries. *Journal of Materials Chemistry A* **12**, 20097-20106 (2024).

5. Guo L, *et al.* Formate-Anion-Induced Water Network Reshaping Enables Concurrent Hydrogen and Magnesium Hydroxide Production From Seawater. *Angew Chem Int Ed Engl*, e6937994 (2026).

6. Wen Q, *et al.* Engineering a Local Free Water Enriched Microenvironment for Surpassing Platinum Hydrogen Evolution Activity. *Angewandte Chemie International Edition* **61**, e202206077 (2022).

7. Wu L, Huang W, Li D, Zhao B, Zhou H, Luo W. Role of Interfacial Water in Improving the Activity and Stability of Lattice-Oxygen-Mediated Acidic Oxygen Evolution on RuO2. *Angewandte Chemie International Edition* **64**, e202420848 (2025).

8. Gallina ME, Sassi P, Paolantoni M, Morresi A, Cataliotti RS. Vibrational analysis of molecular interactions in aqueous glucose solutions. Temperature and concentration effects. *J Phys Chem B* **110**, 8856-8864 (2006).

9. Wang S, *et al.* Weak H-Bond Interface Environment for Stable Aqueous Zinc Batteries. *ACS Nano* **19**, 4484-4495 (2025).

10. Cohen-Tanugi D, Grossman JC. Water Desalination across Nanoporous Graphene. *Nano Letters* **12**, 3602-3608 (2012).

11. Park H, *et al.* Spectroscopic and computational insight into the intermolecular interactions between Zwitter-type ionic liquids and water molecules. *Chemphyschem : a European journal of chemical physics and physical chemistry* **11**, 1711-1717 (2010).

12. Mukhopadhyay A, Xantheas SS, Saykally RJ. The water dimer II: Theoretical investigations. *Chemical Physics Letters* **700**, 163-175 (2018).

13. He Z, Zhou J, Lu X, Corry B. Bioinspired graphene nanopores with voltage-tunable ion selectivity for Na^+ and K^+. *ACS Nano* **7**, 10148-10157 (2013).

Reviewer (Comment 2): *Lines 220-223: The authors should provide citations for the interfacial polymerization used to fabricate membranes. For their QBTS NF membranes, the authors should clearly explain how the QBTS is bound to the polymer network based on their methodology, as it is unclear based on current description.*

Our Response: We thank the reviewer for the comment. In this study, QBTS was grafted onto the membrane surface via the reaction of its hydroxyl groups with residual acyl chloride groups of TMC in the freshly prepared polyamide active layer, i.e., an esterification reaction between acyl chloride and hydroxyl groups. This method is also commonly used to graft functional groups containing hydroxyl groups onto polyamide membranes [1, 2, 3, 4].

As shown in Fig. S5, we have added a schematic illustrating the esterification reaction mechanism between QBTS and TMC. Specifically, the interfacial polymerization reaction between PIP and TMC forms a nascent polyamide active layer. The residual acyl chloride groups of TMC undergo an esterification reaction with the hydroxyl groups of QBTS under the catalysis of 4-dimethylaminopyridine (DMAP), a nucleophilic acylation transfer catalyst[5, 6], thereby immobilizing QBTS onto the polyamide network.

In addition, following the reviewer's suggestion, we have added schematic illustrations of the grafting mechanisms of the other four grafting monomers used in this study, as shown in Fig. S5. These monomers contain hydroxyl or primary amine groups, where the hydroxyl groups react with acyl chloride groups to form ester bonds, while the primary amine groups react with acyl chloride groups to form amide bonds[7, 8, 9]. Accordingly, these monomers can react with the residual acyl chloride groups on the surface of the newly prepared polyamide nanofiltration membrane and become grafted onto the polyamide network through the formation of ester or amide bonds. We have added additional details in the Methods section of the manuscript to provide further clarification regarding this approach.

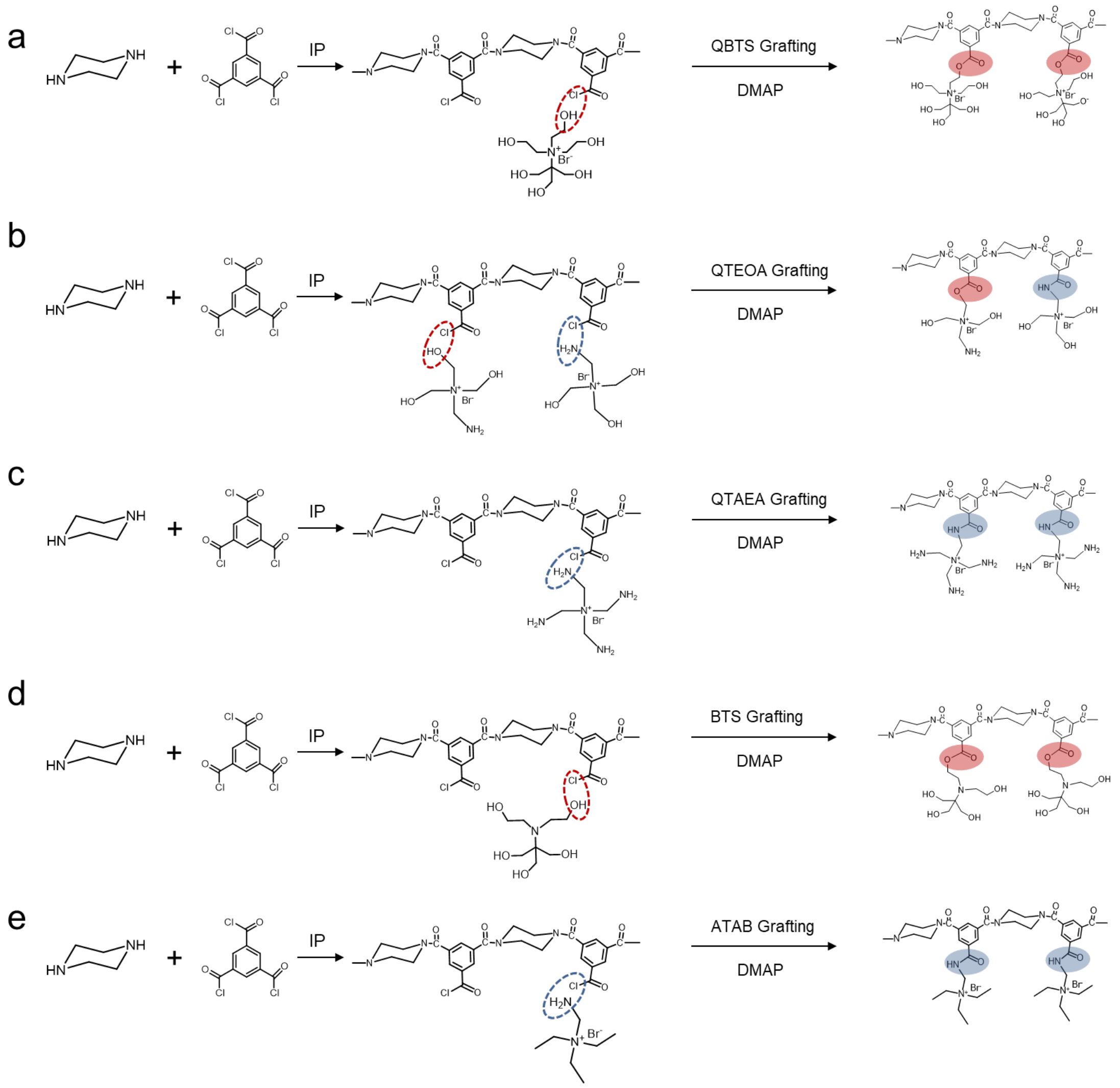

Fig S5. Schematic illustration of the principle of membrane fabrication. (a)-(e) The main reaction process of interfacial polymerization and surface modification by QBTS, QTEOA, QTAEA, BTS, and ATAB, respectively. In this study, QBTS was grafted onto the membrane surface via an esterification reaction between its hydroxyl groups and the residual acyl chloride groups of TMC within the freshly formed polyamide active layer. This grafting strategy is widely employed for anchoring hydroxyl-containing functional groups onto polyamide membranes. The process begins with the interfacial polymerization of PIP and TMC to generate a nascent polyamide active layer. Subsequently, under the catalysis of 4-dimethylaminopyridine (DMAP)—a nucleophilic acyl transfer catalyst—the remaining acyl chloride groups of TMC undergo esterification with the hydroxyl groups of QBTS, thereby covalently attaching QBTS to the polyamide network. (b)-(e) show the reaction principles of other monomers contain hydroxyl or primary amine groups for surface modification. The hydroxyl groups react with acyl chloride groups to form ester bonds, while the primary amine groups react with acyl chloride groups to form amide bonds.

MS-Line 665 For the preparation of QBTS NF membranes, ~~a freshly prepared PIP-TMC NF membrane was treated with an aqueous solution of QBTS (1 wt%) and DMAP (1 wt%) for 10 minutes.~~ QBTS was grafted onto the membrane surface via the reaction of its hydroxyl groups with residual acyl chloride groups of TMC in the freshly prepared polyamide active layer, i.e., an esterification reaction between acyl chloride and hydroxyl groups. This method is also commonly used to graft functional groups containing hydroxyl groups onto polyamide membranes. As shown in Fig. S5, a freshly prepared PIP-TMC NF membrane was treated with an aqueous solution of QBTS (1 wt%) and DMAP (1 wt%) for 10 minutes. The residual acyl chloride groups of TMC undergo an esterification reaction with the hydroxyl groups of QBTS under the catalysis of 4-dimethylaminopyridine (DMAP), a nucleophilic acyl transfer catalyst, thereby immobilizing QBTS onto the polyamide network. It is important to note that the PIP-TMC NF membrane was not dried prior to the QBTS modification. The residual reaction solution was then removed, and the membrane was heated at 50° C for 10 minutes.

MS-Line 683 Modifications of the PIP-TMC NF membrane with other monomers (QTAEA, BTS, ATAB, and QTEOA) followed similar protocols. Detailed explanation of the reaction mechanisms of the other monomers was shown in *Supplementary information*.

References:

1. Peng H, *et al.* Phosphonium Modification Leads to Ultrapermeable Antibacterial Polyamide Composite Membranes with Unreduced Thickness. *Adv Mater* **32**, e2001383 (2020).

2. Han R, *et al.* Piperazine-modulated bis-tris-based polyester-amide nanofilm for high-performance nanofiltration membrane. *Advanced Membranes* **8**, 100220 (2026).

3. Li N, *et al.* Tuning composite nanofiltration membranes with γ-cyclodextrin for improved Mg2+/Li+ selectivity. *Separation and Purification Technology* **330**, 125206 (2024).

4. Li J, Peng H, Liu K, Zhao Q. Polyester Nanofiltration Membranes for Efficient Cations Separation. *Advanced Materials* **36**, 2309406 (2024).

5. Cheng J, *et al.* A novel polyester composite nanofiltration membrane prepared by interfacial polymerization catalysed by 4-dimethylaminopyridine: Enhanced the water permeability and anti-fouling ability. *Polymer* **153**, 24-32 (2018).

6. Zhang R, *et al.* A novel polyesteramide thin film composite nanofiltration membrane prepared by interfacial polymerization of serinol and trimesoyl chloride (TMC) catalyzed by 4-dimethylaminopyridine (DMAP). *Journal of Membrane Science* **542**, 68-80 (2017).

7. Baig U, Waheed A. Exploiting interfacial polymerization to fabricate hyper-cross-linked nanofiltration membrane with a constituent linear aliphatic amine for freshwater production. *npj Clean Water* **5**, (2022).

8. Wanjiya M, Jin C-G, Shen Y, Yin M-J, An Q-F. Conversion of membrane surface charge property via post-crosslinking for heavy metal ions removal. *Separation and Purification Technology* **362**, (2025).

9. Yu D, Li M, Zhou H, Liang X, Shou Q, Liu H. Polyamide composite nanofiltration membrane via a combination of polydopamine grafting and reverse interfacial polymerization for high Mg2+/Li+ separation capacity. *Separation and Purification Technology* **355**, (2025).

Reviewer (Comment 3): *Figure 4 (a, b, e): Should the Y-axis here actually be water permeance? It is labeled as water flux currently but has units of permeance.*

Our Response: We thank the reviewer for the detailed comment. Indeed, the correct y-axis label should be “water permeance”. We apologize for this mistake and have corrected all related descriptions accordingly.

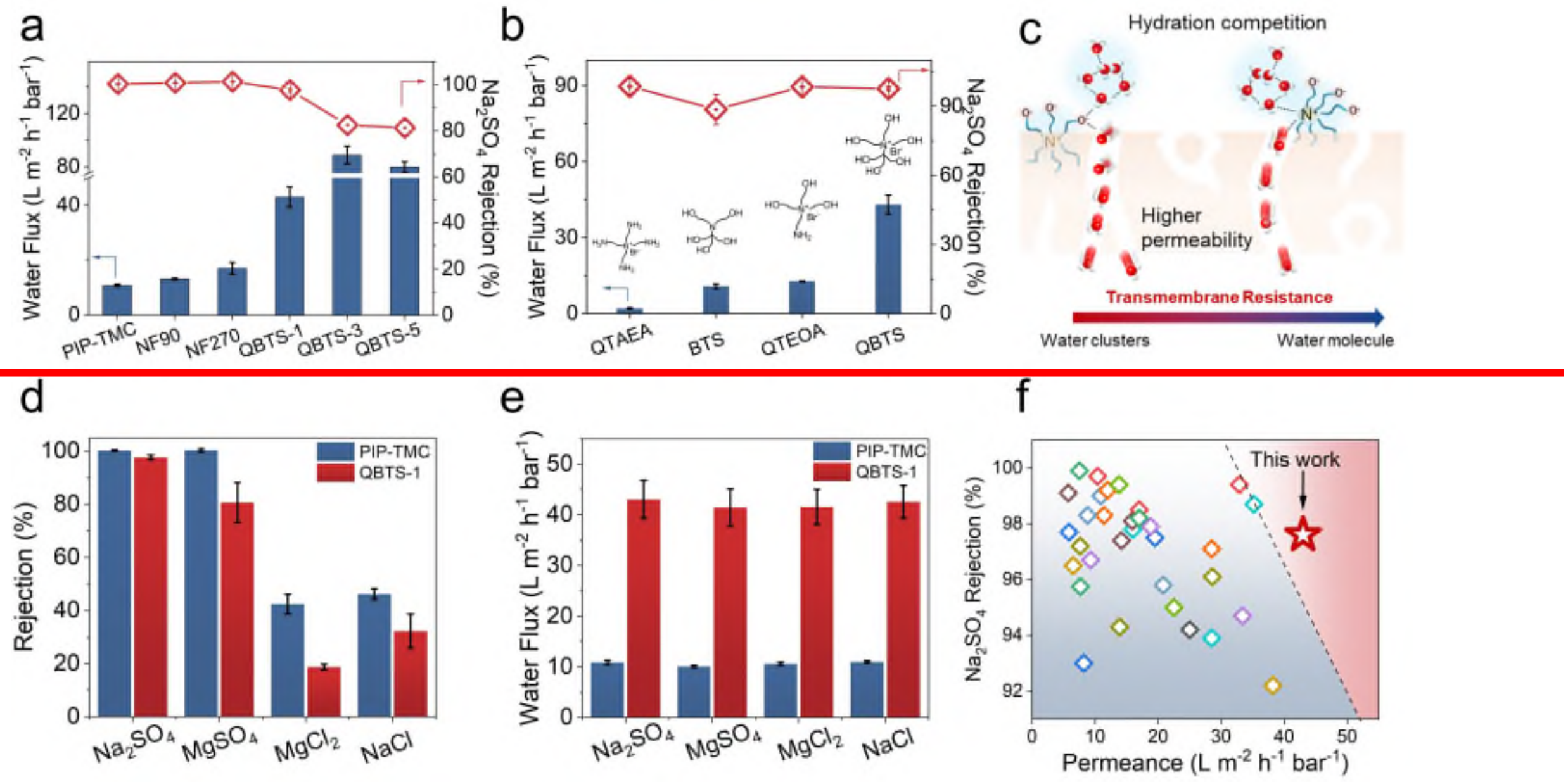

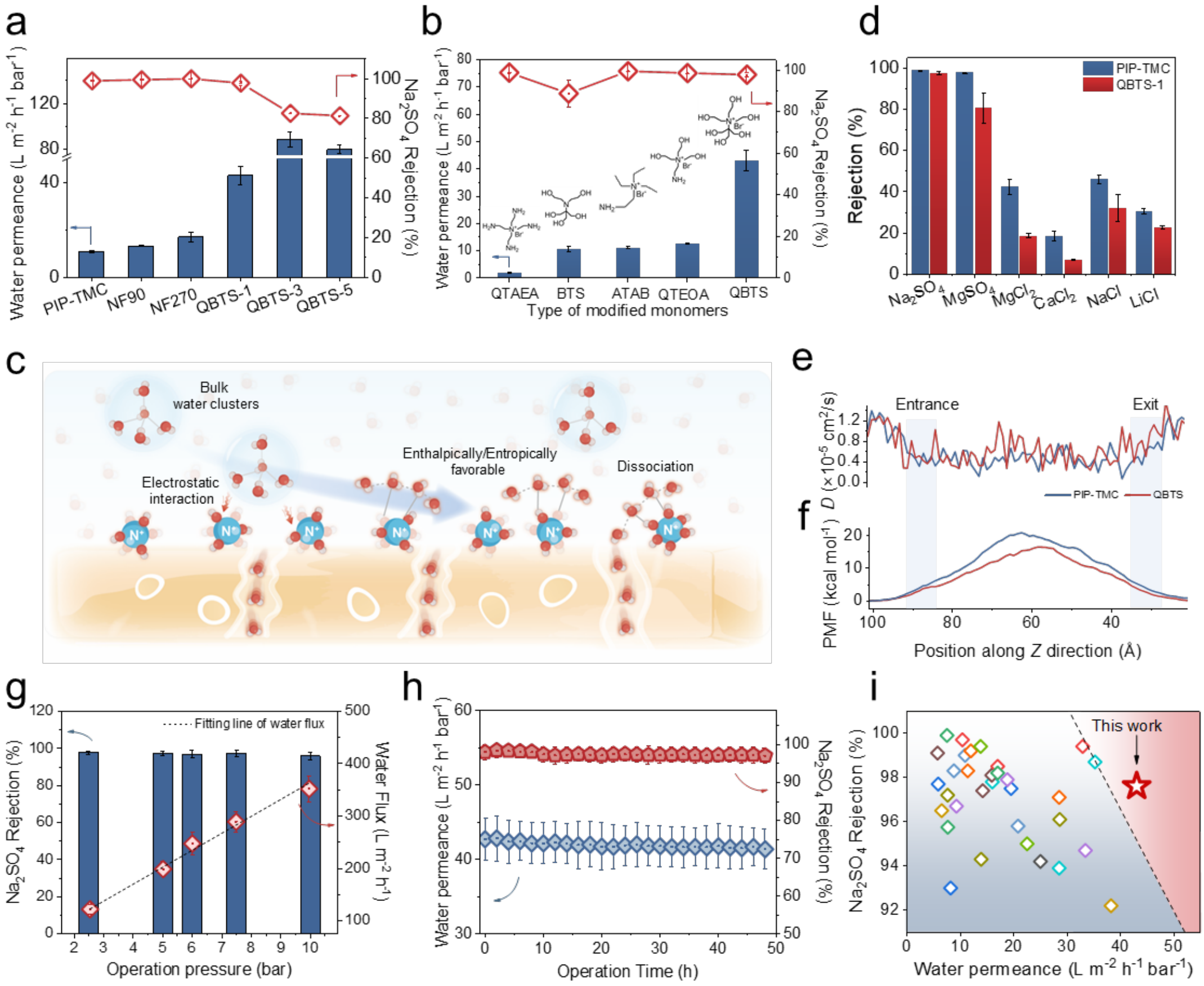

Fig. 4 Filtration performance of QBTS functionalized NF membranes. (a) Effect of QBTS monomer concentration on the separation performance of NF membranes (feed: 1000 ppm Na_2SO_4, 6 bar pressure). (b) Filtration performance of NF membranes modified with different ~~amine~~ monomers (feed: 1000 ppm Na_2SO_4, 6 bar pressure). (c) Schematic diagram of the suppositional mechanism of functional groups promoting the dissociation of water clusters. The dashed lines between water molecules indicate that the original water-water hydrogen bonds are weakened by the polyhydroxy structure of QBTS. (d) Salts rejection ~~and (e) water flux~~ of Na_2SO_4, $MgSO_4$, $MgCl_2$, $CaCl_2$, LiCl and NaCl by PIP-TMC and QBTS-1 membranes (Salt concentration, 1000 ppm; applied pressure, 6 bar~~; temperature, 25 °C~~). (e) The potential of mean force (PMF) and (f) diffusion coefficients (*D*) of Cl^- along the diffusion coordinate in PIP TMC and QBTS simulated nanochannels. The filtration performance of QBTS-1 under different (g) operation pressure and (h) operation Time (feed: 1000 ppm Na_2SO_4, 6 bar pressure). ~~(f)~~ (i) Performance comparison of QBTS-1 membrane with the state-of-the-art NF membranes using surface modification in reported literature (details in Table. S2).

Reviewer (Comment 4): *Figure 4 c and Lines 330-343: It should be made clear here (and perhaps elsewhere), that this is based on a proposed mechanism. The performance data is quite interesting, though the connection to mechanisms is somewhat speculative. It is reasonable to suggest these statements, though it should be clear these are proposed ideas based on observations.*

Our Response: We greatly appreciate the reviewer's comments. Following the reviewer's suggestions, we have thoroughly revised the manuscript to more clearly distinguish which conclusions are inferred from experimental observations. Through additional experiments and by drawing on previous reports, we have strengthened the connections between our experimental observations and inferences, thereby enhancing the rigor of the paper. Because this is a long comment, we split it into two parts for us to address all concerns raised by the reviewer.

Part 1 of comment 4: regarding Fig.4c

First, we would like to clarify that Fig.4c presents a hypothetical mechanism of how the polyhydroxy quaternary ammonium structure of QBTS may affect water cluster structure, which is proposed based on our experimental observations combined with previous studies on the influence of functional group–water interactions on water cluster structure. We will clarify the basis for proposing this mechanism from three aspects:

i) First, the reason for the enhanced water permeability of the QBTS-1 modified membrane originates from its unique surface characteristics, rather than structural changes such as membrane thickness, pore size, specific surface area, etc. As shown in Fig. 3f, the thickness of QBTS even increased to 179 nm, which would increase water transport resistance and is unfavorable for water permeation. We further calculated the hydraulic water permeabilities P_w^H of the membranes—a thickness-independent property that is governed by the physical confinement and chemical interactions experienced by water during transmembrane transport (Fig. S14)[1]. The P_w^H for QBTS (7.7 L μm · m^{-2} · h^{-1} · bar^{-1}) is much higher than that for the unmodified PIP-TMC (1.6 L μm · m^{-2} · h^{-1} · bar^{-1}). Given that key parameters such as pore size distribution, specific surface area, and surface roughness remain comparable between PIP-TMC and QBTS (Fig. 3d, 3e, 3g and 3h), this indicates that the increase in water flux of QBTS is mainly contributed by the chemical characteristics of the membrane surface. This is further supported by the enhanced hydrophilicity and increased negative surface charge of the membrane (Fig. 3i and 3j). Previous studies have also shown that the contribution of hydrophilicity and surface charge to improved water permeance is inseparable from the functional group-water cluster interactions. Typical hydrophilic functional groups, such as -OH, cause local distortion of water clusters through strong interactions[2], prolong the rotational relaxation time of water molecules[3], and orient water molecules in an ordered and restricted manner[3, 4]. These effects collectively cause water molecules

to spread along the membrane surface rather than aggregating into clusters. This finding is consistent with our observations in Fig. 2b and 2c. Furthermore, with respect to Zeta potential, an appropriate membrane surface charge can disrupt the H-bond network between water molecules through electrostatic interactions and reduce the entropic penalty, thereby lowering the energy barrier for water molecules entering the nanochannels and enhancing water flux[5].

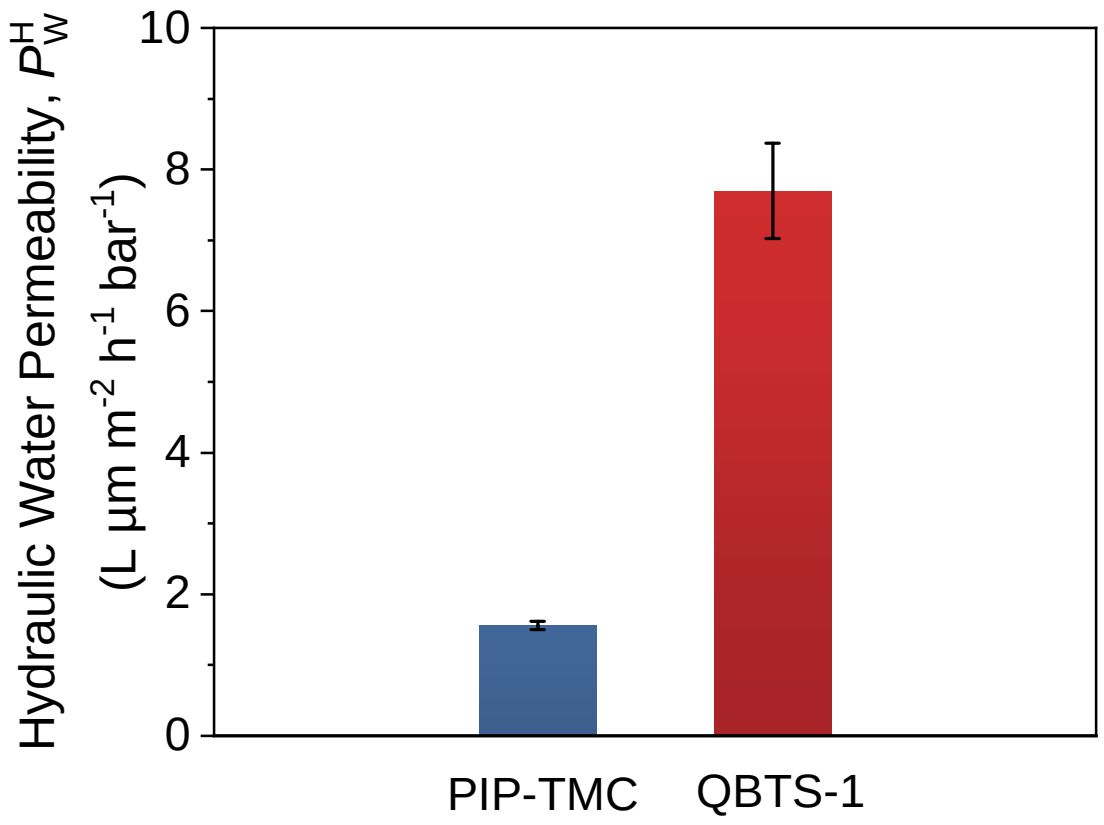

Fig. S14 The hydraulic water permeabilities P_w^H of the PIP-TMC and QBTS membranes. P_w^H is a thickness-independent property that is governed by the physical confinement and chemical interactions experienced by water during transmembrane transport. The P_w^H can be calculated by：

$$P_w^H = \frac{J_w \times \delta}{(\Delta P - \Delta\pi)}$$

where J_w is the water permeance, ΔP is the driven pressure, $\Delta\pi$ is theosmotic pressure. The P_w^H for QBTS (7.7 L μm · m^{-2} · h^{-1} · bar^{-1}) is much higher than that for the unmodified PIP-TMC (1.6 L μm · m^{-2} · h^{-1} · bar^{-1}). Given that key parameters such as pore size distribution, specific surface area, and surface roughness remain comparable between PIP-TMC and QBTS (Fig. 3d, 3e, 3g and 3h), this indicates that the increase in water flux of QBTS is mainly contributed by the chemical characteristics of the membrane surface.

ii) Second, based on existing literature and our observations, the polyhydroxy quaternary ammonium structure on the modified membrane surface has the ability to perturb/dissociate water clusters. As we mentioned in Comment 1， when functional groups possess H-bond acceptors/donors that can interact more strongly with water molecules, they tend to form stronger local interactions with water preferentially, thereby “capture” water molecules, creating oriented anchoring, cutting off or weakening the original water-water H-bond connectivity, reducing the thermodynamic benefit of maintaining the tetrahedral network, and transforming the continuous ordered water network into a more disordered, defect-rich, higher-entropy local structure[2, 6, 7, 8, 9]. The significantly strong interaction and longer H-bond lifetime between QBTS and water molecules supports this point (Fig. 2e and Fig. 2f). Meanwhile, it has been confirmed that the hydration free

energy of quaternary-N^+ is significantly more favorable than that of neutral amines[10], meaning that the quaternary-N^+ center itself may attract water clusters toward the membrane pore through electrostatic interactions between charge and water molecular dipole.

iii) Third, previous studies have shown that the dissociation of water clusters at the membrane surface can promote water permeation. Previous studies have indicated that such perturbation of water clusters by surface functional groups can generate smaller water clusters that are more favorable for transport[11, 12, 13], provide more water molecular orientations to reduce the entropic penalty[14], and simultaneously lower the energy barrier of the dissociation process through a compensation effect[14, 15, 16]. This facilitates water partitioning into the membrane phase[14].

Furthermore, based on the effects of modification with different monomers, we further discuss the respective roles of the quaternary ammonium center and hydroxyl groups in improving water flux. When both are present together, the enhancement in water permeance is more pronounced. These observations provide a reasonable basis for inferring that the polyhydroxy quaternary ammonium structure of QBTS near the membrane pores enhances water flux by influencing water cluster structure.

Overall, Fig. 4c can be described as follows: the polyhydroxy quaternary ammonium structure on the membrane surface can attract water clusters toward the membrane pore via electrostatic interactions. When water clusters interact with the polyhydroxy structure, dissociation of the water clusters occurs. This process has three effects: i) the formation of smaller water clusters; ii) a reduction in the enthalpic barrier required for dissociation through a compensation effect; and iii) providing more orientations for water molecules, thereby reducing the entropic penalty. Consequently, this process is entropically and enthalpically favorable. We must emphasize again, that Fig. 4c just presents a hypothetical mechanism based on partial experimental observations and previous studies, and this mechanism is supported by the results shown in Fig. 1, Fig. 2, and Fig. 5.

The specific revisions are as follows:

MS-Line 343 To gain deeper insights into the mechanism by which surface functional groups on the active layer enhance water flux, we selected ~~four~~ five amine monomers with similar spatial structures but different functional groups to functionalize the TMC-PIP membrane, i.e., *N*, *N*, *N*, *N*-Tetra (2-aminoethyl) ammonium bromide (QTAEA), Bis-(2-Hydroxyethyl) amino-tris (Hydroxymethyl) methane (BTS), *N*, *N*, *N*-triethyl-N-(2-aminoethyl) ammonium bromide (ATAB), *N*, *N*, *N*-Tris (2-hydroxyethyl)-*N*-(2-aminoethyl) ammonium bromide (QTEOA) and QBTS (details in Fig. 4b and Fig. S9). ~~As the hydroxyl content in the monomer increased, the water flux of the functionalized membranes also improved, following the order: QTAEA < BTS < QTEOA < QBTS (Fig. 4b). We observed that the water flux of the membrane modified with QTAEA was even lower than that of the pristine PIP-TMC membrane. More importantly, the water flux of the membranes modified with BTS was lower compared to QTEOA and QBTS functionalized membranes, i.e., BTS < QTEOA < QBTS (Fig. 4b).~~ The water flux order of the functional membranes is as follows: QTAEA < BTS < ATAB < QTEOA < QBTS (Fig. 4b). In the absence of hydrophilic hydroxyl groups, the highly reactive -NH_2 of QTAEA undergo multi-point reactions with residual acyl chlorides, forming a locally dense structure that is unfavorable for water permeation; consequently, its water permeance is even lower than that of PIP-TMC. For BTS, although it contains a relatively high number of hydroxyl groups, it lacks a quaternary-N^+, showing a modest improvement in water flux. Previous studies have shown that the hydration free energy of quaternary ammonium cations is significantly more favorable than that of neutral amines, meaning that the quaternary-N^+ center itself is a good hydration site—as reflected by the slight increase in water flux observed for ATAB compared with PIP-TMC. QTEOA possesses both a polyhydroxy structure and a quaternary-N^+ center, but its high reactive -NH_2 likely leads to a relatively dense active layer. In contrast, QBTS is grafted via a less reactive esterification reaction and possesses both polyhydroxy groups and a quaternary-N^+ center, thus exhibiting a significantly enhanced water-transport effect compared with the other monomers. Overall, we suppose that when a monomer contains both abundant polyhydroxy structures and a quaternary-N^+ center, it enhances the water permeance of the membrane.

MS-Line 371 ~~Based on the binding energy data in Fig. 1c, we hypothesize that there may be a synergistic effect between the -OH and -R_3N^+ on the dissociation of water clusters to facilitate the transport of water molecules.~~ The physicochemical characteristics and hydraulic permeability results of QBTS (Fig. S14) also indicate that the enhancement in water permeance mainly originates from the membrane surface chemistry. Based on the results shown in Fig. 2b-2c and 2e-2f, and previous reports, the polyhydroxy structure of QBTS is expected to exhibit stronger interactions with water molecules, thereby perturbing or even dissociating water clusters. This could help form a more disordered, defect-rich, higher-entropy local structure. Moreover, the positively charged quaternary-N$^+$ center has also been reported to attract water molecules, facilitating the formation of a hydration layer. Based on these facts, we speculate the following mechanism for the enhanced water permeance of QBTS. Specifically, as shown in Fig. 4c, during the partitioning process where water clusters from the aqueous phase go into the membrane phase driven by pressure, larger water clusters approach the active sites through the electrostatic adsorption by ~~-R_3N^+~~ quaternary-N$^+$ in QBTS~~ and undergo dissociation due to the hydration competition of -R_3N^+ and -OH with H-bond donor/acceptor. It allows water clusters to enter the nanochannels in polymeric membranes with smaller traversing species, thereby reducing the resistance to water transport for higher water flux.~~. Subsequently, the polyhydroxy network in QBTS preferentially interacts with water molecules, disrupting the original water-water network and forming a locally loose water structure. Numerous previous studies have shown that such functional group-water interactions can lower the enthalpic barrier for dissociation through a compensation effect and reduce the entropic penalty by providing more available orientations for water molecules. This entropically and enthalpically favorable effect likely contributes to the excellent water flux of QBTS.

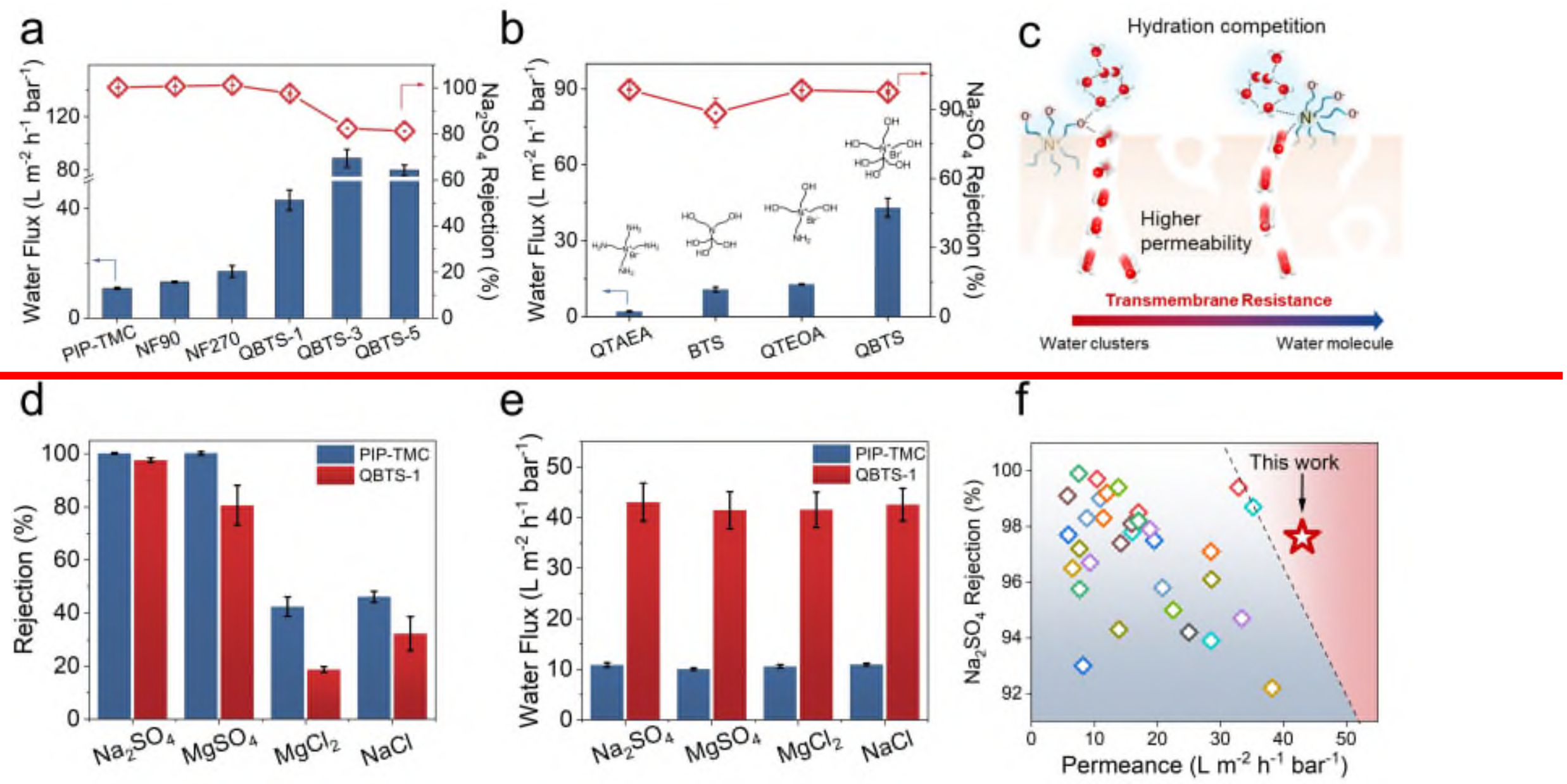

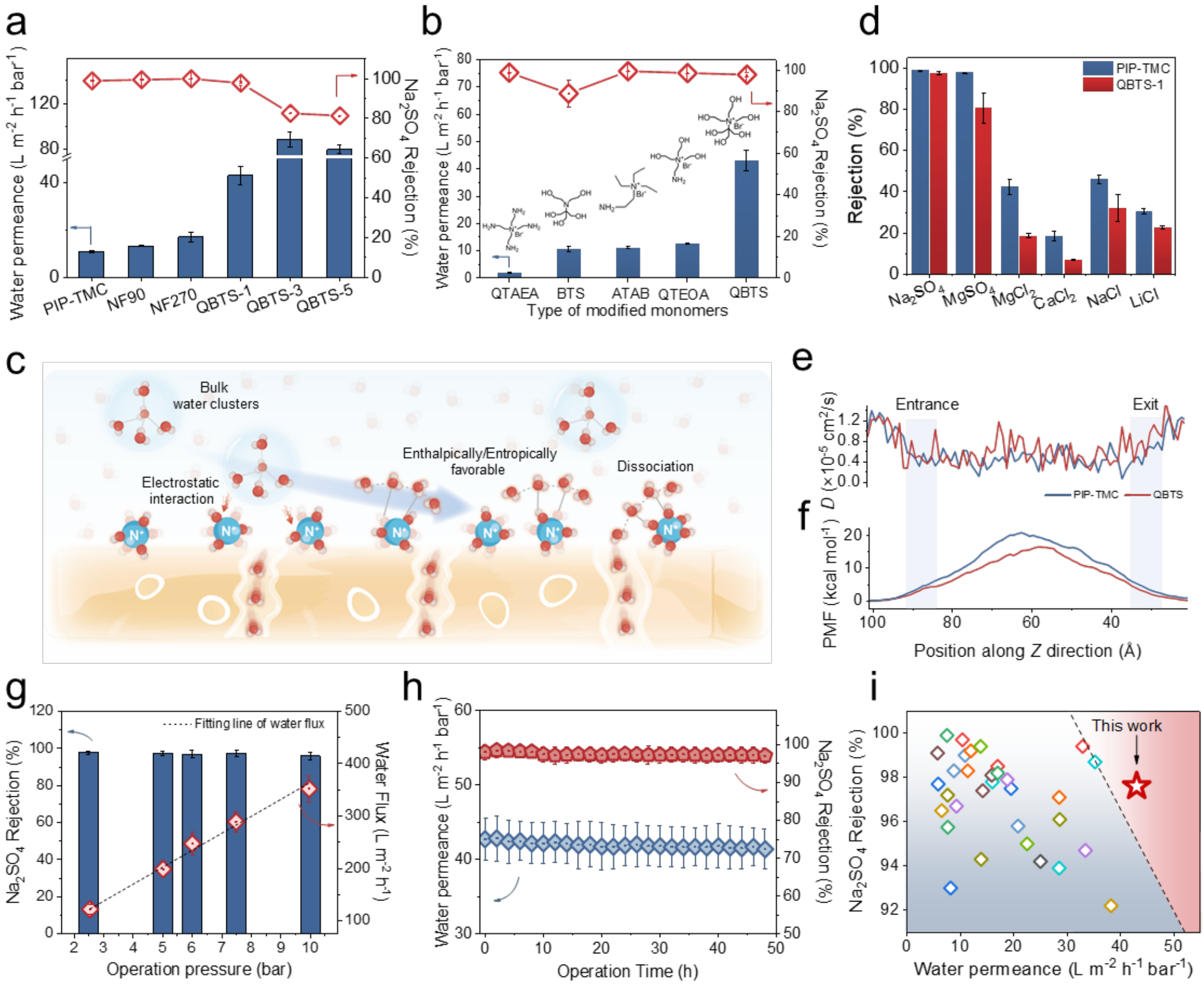

Fig. 4 Filtration performance of QBTS functionalized NF membranes. (a) Effect of QBTS monomer concentration on the separation performance of NF membranes (feed: 1000 ppm Na_2SO_4, 6 bar pressure). (b) Filtration performance of NF membranes modified with different amine monomers (feed: 1000 ppm Na_2SO_4, 6 bar pressure). (c) Schematic diagram of the suppositional mechanism of functional groups promoting the dissociation of water clusters. The dashed lines between water molecules indicate that the original water-water hydrogen bonds are weakened by the polyhydroxy structure of QBTS. (d) Salts rejection and (e) water flux of Na_2SO_4, $MgSO_4$, $MgCl_2$, $CaCl_2$, LiCl and NaCl by PIP-TMC and QBTS-1 membranes (Salt concentration, 1000 ppm; applied pressure, 6 bar; temperature, 25 °C). (e) The potential of mean force (PMF) and (f) diffusion coefficients (D) of Cl^- along the diffusion coordinate in PIP TMC and QBTS simulated nanochannels. The filtration performance of QBTS-1 under different (g) operation pressure and (h) operation Time (feed: 1000 ppm Na_2SO_4, 6 bar pressure). (f) (i) Performance comparison of QBTS-1 membrane with the state-of-the-art NF membranes using surface modification in reported literature (details in Table. S2).

Part 2 of comment 4: regarding Line 330-343 and elsewhere

Regarding the changes in salt rejection after QBTS modification discussed in Lines 330–343, we have also provided the following clarification.

Attributing the reduction of salt rejection, especially the lowest rejection of $MgCl_2$, solely to “H-bond disruption” is not sufficiently comprehensive and detailed. According to the widely accepted nanofiltration separation mechanism[17, 18, 19, 20],

salt rejection is typically determined by the ion with lower permeability[21, 22]. Factors affecting ion permeability include membrane surface charge (Donnan effect)[22], membrane pore size (size sieving)[22], and ion-membrane interactions (adsorption)[23, 24], among others. As shown in Fig. R1, previous studies have demonstrated that the rejection of different salt types responds distinctly to membrane surface charge [17, 21]. For $MgSO_4$, rejection is typically governed by the co-ion SO_4^{2-}. Since QBTS maintains a relatively negative Zeta potential (Fig. 3j) and SO_4^{2-} show relatively larger ion size and higher charge, the $MgSO_4$ rejection of QBTS is well preserved.

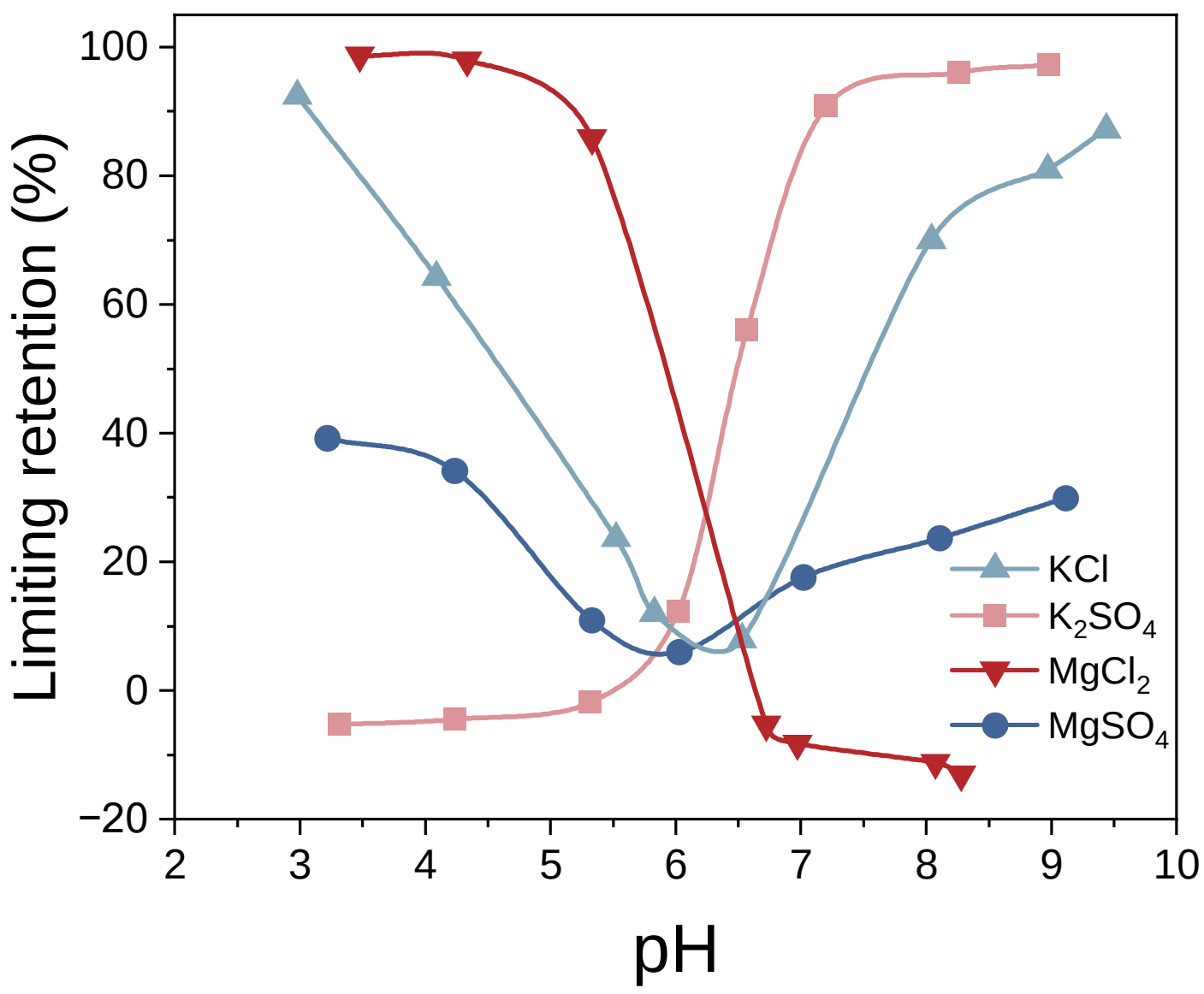

Fig. R1 Limiting retention of single solutions as a function of pH at fixed concentration of 1 mmol L^{-1}. Figure adapted from [17, 21].

For $MgCl_2$, the salt rejection is determined by the Mg^{2+}. Since the pore size distributions of QBTS-1 and PIP-TMC were almost identical (Fig. 3d), we speculate that this reduction in salt rejection originates from the unique surface characteristics of QBTS. First, the lower Zeta potential of QBTS facilitates the adsorption of cations onto the membrane surface (Fig. 3j). Second, the polyhydroxy quaternary ammonium structure can complex with cations, particularly Mg^{2+}, further enhancing the affinity between the cations and the membrane. These Mg^{2+} ions adsorbed onto the membrane surface further induce charge neutralization, thereby weakening the repulsion of the membrane surface negative charge against co-ions[23, 24, 25].

Finally, we took Cl^- as an example and used molecular dynamics to investigate ion rejection behavior. As shown in Fig. 4f, the PMF of Cl^- in QBTS was lower, decreasing from 20.75 to 16.39 kcal mol^{-1}. Meanwhile, as shown in Fig. 4e, during transport through the QBTS pore, Cl^- exhibited several favorable transport regions, such as near the pore entrance (~90 Å) and at the pore center (~60 Å), showing higher diffusion coefficients. The calculated permeability coefficient across the

entire membrane increased correspondingly from 1.10×10^{-13} to 1.20×10^{-10} cm s^{-1}, indicating that Cl^- is more easily transported in QBTS. Based these previous studies, we speculate two possible reasons for the reduction of Cl^- rejection: i) the polyhydroxy structure in QBTS can interact with Cl^- through a "dehydration compensation" mechanism[14, 15, 16], replacing some of the bound water lost from the hydrated Cl^-, thereby stabilizing the dehydration process of Cl^- and lowering the dehydration energy barrier; ii) the quaternary-N^+ near the pore entrance may help direct Cl^- toward the pore by electrostatic adsorption, making Cl^- transport more favorable.

These effects synergistically enhance salt partitioning and thus the permeability, thereby leading to a decrease in salt rejection. Compared with SO_4^{2-}, Cl^- has a smaller hydrated size and less negative charge. Therefore, $MgCl_2$, $CaCl_2$, NaCl, and LiCl are more susceptible to the aforementioned factors, resulting in a lower rejection (Fig. 4d). We have also revised the relevant state to better clarify the reasons for the change in QBTS rejection.

The remaining revisions for enhancing the connections between our experimental observations and inferences in the paper are as follows:

MS-Line 211 In summary, a water cluster-breakage near the functionalized pore edges was found due to the hydration competition, which, according to published claims, would be beneficial for enlarging the water transport area and reducing the transmembrane resistance.

MS-Line 415 This entropically and enthalpically favorable effect likely contributes to the excellent water flux of QBTS. We will discuss the mechanism of QBTS for enhancing water transport in more detail in the next section.

MS-Line 423 Simultaneously, we observed that QBTS-1 slightly reduced the retention of the four salts, particularly for salts containing monovalent Cl^-, i.e., $MgCl_2$, CaCl, LiCl and NaCl, which is reasonably attributed to that -OH and -R_3N^+ on the membrane surface breaking the hydrogen bonds within the water molecule clusters while assisting in the dehydration of hydrated ions and facilitating the transport of ions. We thus used molecular dynamics to investigate ion rejection behavior in simulated polyamide nanochannels (details in *Supplementary information*). As shown in Fig. 4f and 4e, we calculated the potential of mean force (PMF) and diffusion coefficients of Cl^- along the diffusion coordinate in PIP TMC and QBTS simulated nanochannels. Unexpectedly, the PMF of Cl^- in QBTS was lower, decreasing from 20.75 to 16.39 kcal mol-1 (Fig. 4f). Meanwhile, as shown in Fig. 4e, during transport through the QBTS pore, Cl^- exhibited several favorable transport regions, such as near the pore entrance (~90 Å), showing higher diffusion coefficients. Using the inhomogeneous solubility-diffusion model, the calculated permeability coefficient across the entire membrane increased correspondingly from 1.10×10^{-13} to 1.20×10^{-10} cm s^{-1}, indicating that Cl^- is more easily transported in QBTS. This phenomenon seems counterintuitive for QBTS, which has a more negative zeta potential. However, similar observations have been reported in many nanopores with -OH-modified entrances. Based these previous studies, we speculate two possible reasons for the reduction of Cl^- rejection: i) the polyhydroxy structure in QBTS can interact with Cl^- through a "dehydration compensation" mechanism, replacing some of the bound water lost from the hydrated Cl^-, thereby stabilizing the dehydration process of Cl^- and lowering the dehydration energy barrier; ii) the quaternary-N^+ near the pore entrance may help direct Cl^- toward the pore by electrostatic adsorption, making Cl^- transport more favorable. In addition, the polyhydroxy quaternary structure of QBTS can complex with cations, particularly Mg^{2+}, further enhancing the affinity between the cations and the membrane. These Mg^{2+} adsorbed onto the membrane surface further induce charge neutralization, thereby weakening the repulsion of the membrane surface negative charge against co-ions. These effects together result in a more pronounced decrease in rejection of $MgCl_2$, NaCl, LiCl, and $CaCl_2$ after QBTS modification.

References:

1. Ritt CL, *et al.* Ionization behavior of nanoporous polyamide membranes. *Proc Natl Acad Sci U S A* **117**, 30191-30200 (2020).

2. Shin S, Willard AP. Water's Interfacial Hydrogen Bonding Structure Reveals the Effective Strength of Surface-Water Interactions. *J Phys Chem B* **122**, 6781-6789 (2018).

3. Zhang H, Sundaresan S, Webb MA. Molecular Dynamics Investigation of Nanoscale Hydrophobicity of Polymer Surfaces: What Makes Water Wet? *J Phys Chem B* **127**, 5115-5127 (2023).

4. Li E, Du Z, Yuan S. Properties of a water layer on hydrophilic and hydrophobic self-assembled monolayer surfaces: A molecular dynamics study. *Science China Chemistry* **56**, 773-781 (2013).

5. Xu X, *et al.* Water desalination through charged graphene nanoslits: Role of edge chirality and

electrostatic effects. *Colloids and Surfaces A: Physicochemical and Engineering Aspects* **725**, (2025).

6. Zhou L*, et al.* Suppressing Hydrogen Evolution in Aqueous Lithium-Ion Batteries with Double-Site Hydrogen Bonding. *ACS Energy Letters* **8**, 40-47 (2022).

7. Ng LS*, et al.* Chaotropic Nanoelectrocatalysis: Chemically Disrupting Water Intermolecular Network at the Point-of-Catalysis to Boost Green Hydrogen Electrosynthesis. *Angew Chem Int Ed Engl* **63**, e202317751 (2024).

8. Zhu Y, Chen Q, Hao J, Jiao Y. Breaking hydrogen-bonds in aqueous electrolytes towards highly reversible zinc-ion batteries. *Journal of Materials Chemistry A* **12**, 20097-20106 (2024).

9. Guo L*, et al.* Formate-Anion-Induced Water Network Reshaping Enables Concurrent Hydrogen and Magnesium Hydroxide Production From Seawater. *Angew Chem Int Ed Engl*, e6937994 (2026).

10. Singh H, Sharma S. Understanding the Hydration Thermodynamics of Cationic Quaternary Ammonium and Charge-Neutral Amine Surfactants. *J Phys Chem B* **126**, 9810-9820 (2022).

11. Liu G*, et al.* New inspirations for efficient water transport in sub-nanopores: The effect of positive charges at the pore entrance. *Journal of Environmental Chemical Engineering* **13**, (2025).

12. Itoh Y*, et al.* Ultrafast water permeation through nanochannels with a densely fluorous interior surface. *Science* **376**, 738-743 (2022).

13. Tunuguntla RH, Henley RY, Yao Y-C, Pham TA, Wanunu M, Noy A. Enhanced water permeability and tunable ion selectivity in subnanometer carbon nanotube porins. *Science* **357**, 792-796 (2017).

14. Cohen-Tanugi D, Grossman JC. Water Desalination across Nanoporous Graphene. *Nano Letters* **12**, 3602-3608 (2012).

15. Wang M*, et al.* A coupled effect of dehydration and electrostatic interactions on selective ion transport through charged nanochannels. *Nanoscale* **10**, 18821-18828 (2018).

16. He Z, Zhou J, Lu X, Corry B. Bioinspired graphene nanopores with voltage-tunable ion selectivity for Na^+ and K^+. *ACS Nano* **7**, 10148-10157 (2013).

17. Luo J, Wan Y. Effects of pH and salt on nanofiltration—a critical review. *Journal of Membrane Science* **438**, 18-28 (2013).

18. Shefer I, Lopez K, Straub AP, Epsztein R. Applying transition-state theory to explore transport

and selectivity in salt-rejecting membranes: a critical review. *Environmental Science & Technology*, (2022).

19. Wijmans JG, Baker RW. The Solution-Diffusion Model - a Review. *Journal of Membrane Science* **107**, 1-21 (1995).

20. Epsztein R, Shaulsky E, Dizge N, Warsinger DM, Elimelech M. Role of ionic charge density in donnan exclusion of monovalent anions by nanofiltration. *Environmental Science & Technology* **52**, 4108-4116 (2018).

21. Szabolcs Szoke GP, Laszlo Weiser. Characteristics of thin-film nanofiltration membranes at various pH-values. *Desalination* **151**, 123-129 (2002).

22. Cevallos-Cueva N, Rahman MM, Hailu Kinfu H, Abetz V. Mass transport mechanism of nitrate selective nanofiltration membranes on the basis of the Donnan steric pore model with dielectric exclusion (DSPM-DE). *Chemical Engineering Journal* **493**, (2024).

23. Guo H-y, Gao X-q, Yu K-c, Wang X-m, Liu S-m. Ion adsorption on nanofiltration membrane surface and its effect on rejection of charged solutes: A zeta potential approach. *Separation and Purification Technology* **326**, 124830 (2023).

24. Roth RS, Birnhack L, Avidar M, Hjelvik EA, Straub AP, Epsztein R. Effect of solution ions on the charge and performance of nanofiltration membranes. *npj Clean Water* **7**, (2024).

25. Schaep J, Vandecasteele C, Mohammad AW, Bowen WR. Modelling the retention of ionic components for different nanofiltration membranes. *Separation and Purification Technology* **22-3**, 169-179 (2001).

__Reviewer (Comment 5)__: Lines 346-366 and Figure 5 b: The section was very interesting but challenging to parse through. For example, it took me multiple reads to understand "after filtration" was a solution phase measurement. Is the argument that the reader is meant to walk away with that if the hydration distribution shifts, it means that a particular structure of water is more likely to permeate through the membrane? More clarity and few additional sentences in the discussion would be helpful.

Our Response: Thank you for this helpful comment. We agree that the wording around "after filtration" in Fig. 5b may cause ambiguity. First, please allow us to briefly introduce the working principle of the *in situ* liquid ToF-SIMS coupled with a microfluidic filtration device[1, 2, 3, 4]. As shown in Fig. S20 right panel, the aqueous solution is enclosed within a microfluidic filtration device to isolate it from the vacuum. At the start of the experiment, Bi_3^+ continuously bombards the SiN_x film to form a detection aperture, connecting the solution/membrane to the external vacuum. Thus, driven by the transmembrane pressure difference under the external vacuum of the TOF-SIMS instrument, the aqueous solution enters the nanochannels of the membrane and transport to the other side, i.e., completing transmembrane transport (denoted as "after filtration"). Once the aqueous solution exits the membrane pores and reaches the membrane interface, the hydrated species in the solution, such as water clusters $(H_2O)_nH^+$, are exposed to the continuously applied Bi_3^+ beam and are immediately ionized into secondary ions. That is to say, this is a process that occurs in the liquid phase. These secondary ions then enter the ToF-SIMS detector (Fig. S20), where they are detected and distinguished based on their different mass-to-charge ratios. In other words, the water clusters were ionized immediately in the aqueous phase rather than in the gas phase (i.e., *a solution phase measurement*), which helps to ensure that the detected SIMS signals maintain the hydration properties of $(H_2O)_nH^+$ from permeate.

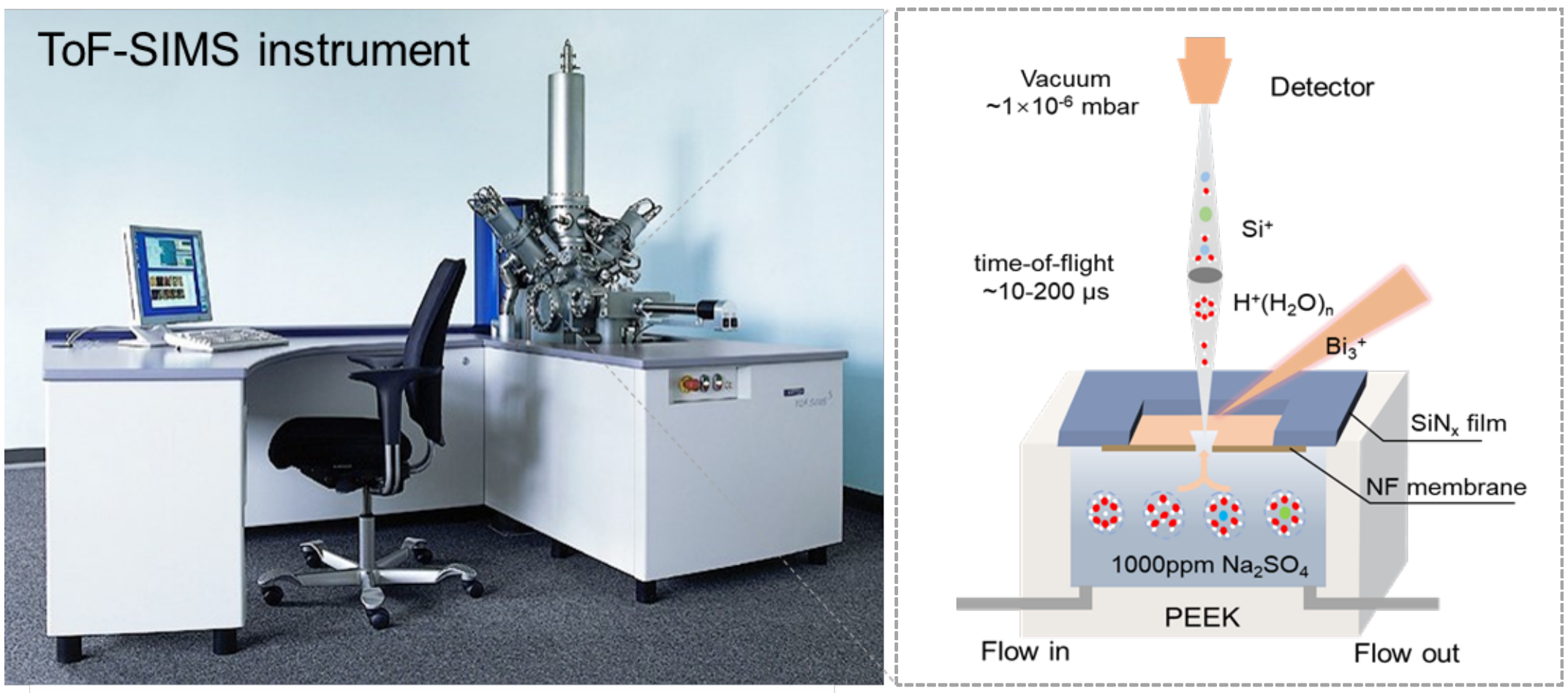

Fig. S20 Schematic diagram of liquid ToF-SIMS. Left: ToF-SIMS instrument; Right: microfluidic filtration device placed in the ToF-SIMS vacuum chamber.

For the bulk solution, we removed the nanofiltration membrane shown in Fig. S20 and performed the measurement following the same procedure. During the

measurement, due to surface tension, the solution forms a liquid surface at the detection aperture. In this way, we are able to detect the mass spectrometric information of water clusters in the bulk solution using the same principle. We define the information of water clusters in the bulk solution as “before filtration”. Conversely, the mass spectrometric information of water clusters detected immediately after they pass through the nanofiltration membrane is defined as “after filtration”. This means that, compared with the hydration distribution in the bulk solution (i.e., before filtration), the hydration distribution of water clusters “after filtration” reflects the influence of the physicochemical characteristics of the nanofiltration membrane, such as surface functional groups and membrane pore size, on their hydration state. For example, in our previous work[1, 3, 5], using this technique we demonstrated that when the pore size is smaller than the size of a hydrated ion, the larger hydrated ion undergoes dehydration and transforms into smaller hydrated species. More intuitively, this means that species such as $(H_2O)_6Na^+$ in the bulk solution (before filtration) transform into $(H_2O)_2Na^+$ (after filtration).

Returning to the results of this study, as shown in Fig. 5b, in the bulk solution, $(H_2O)_nH^+$ exists mainly in the forms of $(H_2O)_5H^+$, $(H_2O)_4H^+$, and $(H_2O)_3H^+$. After filtration through QBTS, $(H_2O)_nH^+$ exists predominantly in the form of $(H_2O)_3H^+$. Meanwhile, the proportions among different water clusters also change after filtration. This indicates that the change in hydration distribution before and after filtration is not simply due to size exclusion retaining larger $(H_2O)_nH^+$ species, but rather involves a structural transformation from larger water clusters to smaller ones[1]. Combining the results shown in Figs. 2b, 2c, 5c, 5d, 5f, and 5g, we conclude that the ToF-SIMS results demonstrate: i) water clusters are indeed transported through nanochannels in a reduced size form, as previously claimed by Wang et al.[6]; ii) QBTS promotes this process with a lower energy requirement, generating more and smaller water clusters. We have reorganized this section to improve its readability.

MS-Line 472 The *operando* characterization of the dissociation of water clusters, i.e., $(H_2O)_nH^+$ as the indicators, during transmembrane transport was conducted on an *in situ* liquid ToF-SIMS microfluidic filtration platform with pristine and functionalized NF membranes (Fig. 5a). The spectra peak intensities of $(H_2O)_nH^+$ (n=1-6) were normalized to the total water clusters mass spectra intensity to form the hydration distributions (denote as h_{H_2O}, Fig. 5b). As shown in Fig. 5a and Fig.S20, we used an *in situ* liquid ToF-SIMS microfluidic filtration platform to analyze the changes in hydration species of water clusters in solution before and after filtration by the pristine and functionalized NF membranes, in order to *operando* analyze the dissociation of water clusters during transmembrane transport (details are provided in the *Supplementary Information*). The spectral peak intensities of $(H_2O)_nH^+$ (n = 1–6) were normalized to the total water cluster mass spectral intensity to obtain the hydration distributions (denoted as h_{H_2O}, Fig. 5b). Briefly, during the measurement, when the SiN_x film is perforated by the Bi_3^+, hydrated species in the aqueous solution, such as $(H_2O)_nH^+$, are driven by the external vacuum pressure difference to pass through the nanofiltration membrane and reach the detection liquid surface. The hydrated species in the liquid phase are immediately ionized and detected *operando* (denoted as “after filtration”). For the bulk solution, we removed the nanofiltration membrane shown in Fig. 5a and performed the measurement following the same procedure. The species detected in this way represent the hydration state of $(H_2O)_nH^+$ in the bulk solution, i.e., the “before filtration”. Therefore, compared with h_{H_2O} before filtration, the h_{H_2O} of water clusters after filtration reflects the influence of the physicochemical characteristics of the nanofiltration membrane, such as surface functional groups and membrane pore size, on their hydration state.

MS-Line 502 After filtration, it shows that the proportion of $(H_2O)_5H^+$ decreased, while that of $(H_2O)_3H^+$ significantly increased compared to the bulk solution (Fig. 5b, left panels). Water clusters are indeed transported through nanochannels in a reduced size form as previous study claimed. We thus hypothesize that the steric hindrance of the confined nanochannels and abundant carboxyl functional groups in the polyamide active layer may contribute to the dissociation of water clusters as we mentioned earlier (Fig. 1c).

MS-Line 510 More importantly, in the h_{H_2O} after filtration through the QBTS-functionalized membrane, this increase in the proportion of $(H_2O)_3H^+$ is more pronounced compared with that of TMC-PIP membrane, as the proportion of $(H_2O)_5H^+$ decreased from 0.24 to 0.15, while that of $(H_2O)_3H^+$ increased from 0.30 to 0.46 (Fig. 5b). As we mentioned earlier, the polyhydroxy quaternary ammonium structure is expected to promote the dissociation of water clusters through strong interactions with water molecules. Therefore, we can deduce that the hydration competition at the active sites of QBTS, i.e., -OH and $-R_3N^+$ might further promote the water clusters dissociation the observed increase in the proportion of $(H_2O)_3H^+$ is likely evidence that the polyhydroxy quaternary ammonium structure promotes the generation of more small-sized clusters, facilitating more favorable transmembrane transport of water molecules.

SI-Line 87 Thus, the $(H_2O)_nH^+$ in feed solution were consequently exposed to the Bi_3^+ primary ion beam and were ~~immediately~~ ionized to secondary ions, forming the instantaneous mass spectrum of interested $(H_2O)_nH^+$. It should be particularly noted that the water clusters from permeate will be ionized immediately once they exit the nanochannel. In other words, the water clusters were ionized immediately in the aqueous phase rather than in the gas phase, which helps to ensure that the detected SIMS signals maintain the hydration properties of ions from permeate. The dynamic ToF-SIMS depth profiling also indicated that the signals of representative species from permeate were immediately increased once the ions reached the detective area. On the other hand, the *in situ* liquid ToF-SIMS has been demonstrated to be a surprisingly soft technique for investigation of water-water interactions. During the determination, hydrates fragmentation in liquid SIMS analysis could be alleviated due to the continuously updated liquid surface around the punched pore via diffusion as well as the flexible liquid environments, which is favored for the formation of intact water clusters. In addition, once the SiN_x film was perforated, the pulse width was immediately reduced to 100 ns, which reduces the intensity of the beam current from 0.27 pA to 0.15 pA. This can mitigate the impact on the hydrated ions from ion irradiation. Furthermore, the ionized hydrates can go through the time-of-flight tube and be detected in an extremely short time (duration ~10-200 μs)—greatly minimizing the possibility of collisions and additional evaporation. As such, the original structure of the water clusters is well maintained after water clusters leave the nanochannel, which can represent the operando changes in the ion hydration structure during transport through nanochannels.

References:

1. Lu C, *et al.* In Situ Characterization of Dehydration during Ion Transport in Polymeric Nanochannels. *J Am Chem Soc* **143**, 14242-14252 (2021).

2. Liu YY, Zhang SZ, Ying YL, Xia HL, Hua X, Long YT. Ion-Specific Effects on Hydrogen Bond Network at a Submicropore Confined Liquid-Vacuum Interface: An in Situ Liquid ToF-SIMS Study. *J Phys Chem Lett* **10**, 4935-4941 (2019).

3. Lu C, *et al.* Dehydration-enhanced ion-pore interactions dominate anion transport and selectivity in nanochannels. **9**, eadf8412 (2023).

4. Zhang Y, *et al.* In situ liquid secondary ion mass spectrometry: A surprisingly soft ionization process for investigation of halide ion hydration. *Anal Chem* **91**, 7039-7046 (2019).

5. Chen Z, *et al.* Steric Hindrance-Induced Dehydration Promotes Cation Selectivity in Trans-Subnanochannel Transport. *ACS Nano* **17**, 12629-12640 (2023).

6. Li Wang JH, Mohammad Heiranian, Hanqing Fan, Lianfa Song, Ying Li, Menachem Elimelech. Water transport in reverse osmosis membranes is governed by pore flow, not a solution-diffusion mechanism. *Science advances* **9**, eadf8488 (2023).

<u>Reviewer (Comment 6)</u>: *Lines 382-383: The authors should mention the temperatures probed here and reference the SI.*

Our Response: Thank you for the reviewer's suggestion. We determined the enthalpic barrier ($\Delta H^{\ddagger}$) and entropic barrier ($\Delta S^{\ddagger}$) by measuring water permeance over a series of temperature gradients: 298.15 K, 303.15 K, 308.15 K, and 313.15 K (25, 30, 35, and 40 °C). The corresponding supplementary information has been added to the manuscript as follows:

> **MS-Line 529** (c) Enthalpic and entropic barriers for the transport of water through the PIP-TMC and QBTS-1 membrane. The energy barriers were calculation fitted by intrinsic water permeability measured at 298.15, 303.15, 308.15, and 313.15 K (details in *supplementary materials*).

> **SI-Line 184** We measured P_i at different temperature of 298.15, 303.15, 308.15, and 313.15 K (i.e., 25, 30, 35, 40°C), respectively.

<u>Reviewer (Comment 7)</u>: *I do not necessarily agree that stronger interactions relate to lower diffusion energy barriers. Stronger interactions have been shown to increase the energy barrier in many simulation studies. Can the authors provide direct evidence that suggests that stronger interactions reduce the energy barrier? Moreover, the authors should cite a broader set of literature in these discussions.*

Our Response: We greatly appreciate the reviewer's critical comment. We strongly agree that directly correlating stronger interactions with lower diffusion barriers is not rigorous. As the reviewer noted, many studies have discussed the influence of functional group-water interactions on water diffusion, however, their conclusions remain controversial.

On the one hand, as the reviewer pointed out, stronger interactions have been shown to increase the energy barrier in many simulation studies. For example, the hindering effects of functional groups on water permeation include electrostatic friction between charged functional groups and water[1, 2], additional steric hindrance from grafted functional groups[3], and extra resistance caused by strong functional group-water interactions[4, 5]. Some typical examples are as follows:

i) Corry et al.[2] found that CNTs functionalized at the pore entrance with $-COO^-$, $-NH_3^+$, -OH, or $-CONH_2$ all exhibited lower water flux than pristine, non-functionalized CNTs. This is because the polar groups attract water molecules, creating residence sites at the pore entrance and slowing down the overall flow velocity.

ii) Qiu et al.[3] found that multi-layer graphene oxide membranes with edge-grafted -COOH groups exhibited a steric hindrance effect at the entrance, blocking water molecules from entering the interlayer gaps. However, in their study, when modified with -OH, the OH group promotes water transport by uniquely “pulling” water molecules across the nanosheet layer because of its relatively stronger interaction with water. This instead enhanced water permeance.

iii) Suk et al.[5] found that when the hydrophilicity at the edges of two-dimensional nanoporous membranes exceeds a threshold, the hydrophilicity causes water molecules to form a highly ordered layered structure in the region proximal to the pore entrance. This strong interaction increases the potential energy barrier for water molecules entering the pore and significantly reduces the diffusion coefficient in that region.

iv) Tunuguntla et al.[6] found that when the -COOH groups at the edges of carbon nanotube porins are ionized, the electrostatic stickiness between the charged functional groups and water molecules drags on water transport.

However, many previous studies have clearly indicated that hydroxyl, carboxyl, and amino groups near the pore entrance can significantly enhance water permeance. Some typical examples are provided as follows:

i) Cohen-Tanugi et al.[7] studied water transport through perforated graphene membranes with -H-terminated and -OH-terminated pores. They found that -OH makes the pore entrance more hydrophilic, increases the effective water transport area, and enhances the accessible H-bonding orientations of water at the entrance, thereby reducing the entropic penalty when water enters the nanopore. As a result, overall water transport is more favorable.

ii) Andrei et al.[8] found that in pillar[5]arene artificial water channels with glycosylated pore entrances, the hydrophilic glycosyl groups act as water-sponge relays: they first adsorb and reorient water molecules, form water reservoirs at the entrance, and then organize the water into a structure conducive to transmembrane transport.

iii) Jafarzadeh et al.[9] reported that -F and -OH functionalized nanoporous BNNS membranes can enhance water permeance. The -F and -OH groups attract water molecules from both sides of the membrane to the pore entrance, facilitate the formation of a chain-like water structure through the pore, and lower the free energy barrier for water passing through the pore.

iv) Fan et al.[10] suggested that the enhancement in water permeance after -OH and $-COO^-$ modification arises because the hydrophilic/charged groups at the pore entrance enhance molecular affinity and preferential occupancy of water at the entrance, reshape the H-bond network and layered distribution inside the pore, thereby reducing pore resistance and promoting water entry into the nanopore.

v) Liu et al.[11] found that carbon nanotube porins with $-NH_3^+$-modified pore entrances significantly enhance water transport. The mechanism is that the positively charged pore entrance induces smaller water clusters, more uniform

dipole orientations, and more readily formed single-file water chains, which not only lower the free energy barrier at the entrance but also reduce intra-pore friction, thus significantly improving overall permeability.

Overall, the partitioning of water from the bulk phase into a nanochannel is a complex process. It is generally accepted that water molecules tend to form stable tetrahedral cluster structures in the bulk phase[12, 13, 14, 15, 16]. The following steps in the transport process may all affect the energy barrier for water permeation: i) whether water clusters can accurately accumulate near the pore entrance without additional energy loss from repeated attempts to approach the pore; ii) when water clusters transition from the bulk phase into an extremely confined environment, they inevitably incur a large entropic and enthalpic penalty due to size reduction. Whether this penalty can be compensated, or whether water clusters have more accessible orientations when entering the pore, is crucial for lowering the transmembrane energy barrier; iii) the interactions between water clusters and the membrane may introduce unexpected transport resistance; iv) the configuration of water clusters after entering the pore is closely related to the intra-pore diffusion resistance.

Returning to our study, it is clear that the stronger interactions between the polyhydroxy functional groups and water molecules inevitably introduce additional resistance to water transport[2]. Nevertheless, based on our experimental observations, we speculate that the polyhydroxy structure of QBTS also offers the following beneficial effects on water transport:

i) As shown in Fig. 2b, the -OH functional groups promote the generation of more free water, which facilitates rapid water transport[11]. Our liquid ToF-SIMS results also confirm that QBTS promotes the formation of more small-sized water clusters (Fig. 5b).

ii) As shown in Fig. 2c, the pulling effect of the polyhydroxy functional groups on water clusters increases the effective water transport area[7].

iii) As shown in Fig. 5d, when QBTS participates in the dissociation of water clusters, the interaction between the functional groups and the defective water clusters takes over part of the stabilization role originally provided by the water-water H-bond network. This compensates for the energetic cost of losing water-water H-bonds[17, 18]. Therefore, in the presence of QBTS, the dissociation process of water clusters is relatively more energetically favorable (Fig. 5c).

iv) We speculate that the polyhydroxy structure can provide more orientations for water molecules during transmembrane transport, thereby helping to reduce the entropic penalty of the water cluster transmembrane process[7]. To verify this, we employed the simplified Arrhenius kinetic model proposed by Jeffrey C. Grossman[7]. Based on the migration data of water molecules near the entrances of the two types of pores shown in Fig. 5g, we estimated the entropic barrier near the pore entrance during water transport through PIP-TMC and QBTS membranes (detailed methods

are provided in the Supplementary Information). The results show that compared with the PIP-TMC pore, the effective entropic penalty near the pore entrance of the QBTS pore is reduced by approximately 0.799 kcal mol^{-1}. This value is comparable to the entropic barrier change calculated using the Eyring equation from transition state theory (Fig. 5c), indicating that QBTS plays a significant entropic modulation role in the local water entry process at the pore entrance.

v) As shown in Fig. 5c, using the Eyring equation based on transition state theory, we confirmed that after QBTS modification, both the apparent enthalpy barrier and the entropic barrier for transmembrane water transport are reduced, which is consistent with the above reasoning.

vi) Finally, in the simulated QBTS and PIP-TMC polyamide nanochannels, we further analyzed the potential of mean force (PMF) during transmembrane water transport using MD simulation. As shown in the Fig. S26, the energy barrier experienced during water transport is lower after QBTS grafting, which also supports the observations in this experiment.

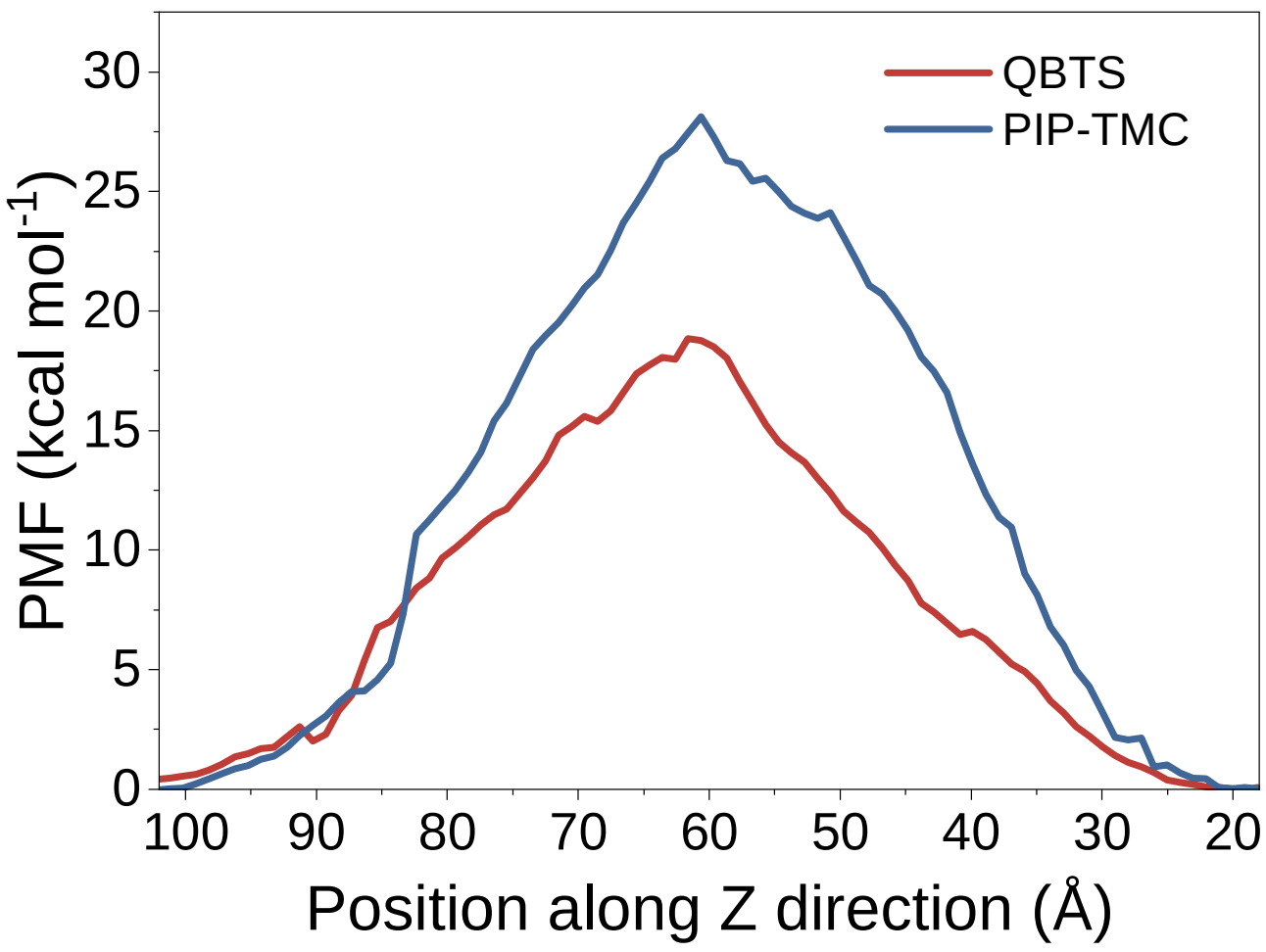

Fig. S26 The potential of mean force (PMF) of H_2O along the diffusion coordinate in PIP TMC and QBTS simulated nanochannels.

In summary, taking into account the reviewer’s suggestions, we have reorganized the mechanism section of the paper to more clearly explain why the stronger interaction between the polyhydroxy quaternary ammonium structure and water molecules is beneficial for water transport. The specific revisions are described as follows:

MS-Line 542 As shown in Fig. 5c and Fig. S11, compared to the PIP-TMC membrane, water molecules traverse the QBTS-1 membrane with a lower enthalpic energy barrier, decreasing from 4.00 kcal mol^{-1} to 3.25 kcal mol^{-1}. ~~This indicates a lower diffusion energy barrier for water molecules, which may result from stronger interactions between the water molecules and the QBTS-1 membrane surface, promoting their partitioning into the membrane for transport.~~ Additionally, water molecules experience a lower entropic barrier when crossing the QBTS-1 membrane, suggesting a higher degree of freedom during permeation, which further facilitates their transmembrane transport. Using the migration data of water molecules in different nanochannels from molecular dynamics simulations (Fig. 5g), the entropic penalty calculated based on the simplified Arrhenius kinetic model further confirms that water diffusion in QBTS is entropically more favorable (*Supplementary Information*). From the perspective of the effective energy barrier calculated by Arrhenius-type equation, we also observed a significant reduction in the energy required for water molecules to cross the modified membrane (Fig. S12).

MS-Line 561 We calculated the Gibbs free energy (ΔG) of a serial of typical dehydration process with and without the participation of functional groups to further evaluate their roles in water clusters dissociation (details in Fig. S13). For the process of dehydration from $(H_2O)_6H^+$ to $(H_2O)_3H^+$, the positive ΔG_1 showed a non-spontaneous dehydration process in the bulk solution. In contrast, the involvement of functional groups (-COO^-, -OH, and -R_3N^+) in the dehydration process (details in Fig. S13) led to a negative ΔG (Fig. 5d), indicating that the hydration competition is favorable for water clusters to spontaneous dissociation to transform as smaller water species. The lower ΔG_3 and ΔG_4 signified that the QBTS functional groups on the active layer make the dissociation of water clusters easier compared to the -COOH in polyamide (Fig. 5d), which also corresponds well with our previous observation by *in situ* liquid ToF-SIMS (Fig. 5b) and membrane performance evaluation (Fig. 4a). Based on the dehydration compensation mechanism, we speculate that when QBTS participates in the dissociation of water clusters, the interaction between the functional groups and the defective water clusters takes over part of the stabilization role originally provided by the water-water H-bond network. This compensates for the energetic cost of losing water-water H-bonds.

MS-Line 591 Specifically, the water clusters can more rapidly enter the entrance of QBTS nanochannel, as their transport speed at the entrance of QBTS nanochannel is approximately three times that at the entrance of PIP-TMC nanochannel (Fig. 5g inset), which can be attributed to the additional "acceleration" to water clusters through energy compensation of hydration competition. Finally, we further analyzed the potential of mean force (PMF) during transmembrane water transport in the PIP-TMC and QBTS simulated nanochannels. As shown in the Fig. S26, the PMF experienced during water transport is lower after QBTS grafting, which also supports the observations of favored water permeance in QBTS.

Although it is undeniable that the stronger interaction between the QBTS and water molecules may introduce unfavorable viscous resistance, however, it promotes water permeation through the following effects. Specifically, based on existing knowledge of the influence of functional groups on water transport and these observations, the polyhydroxy quaternary ammonium structure, through its stronger interactions with water molecules, disrupts the water cluster network and dissociates it into more small-sized water clusters, thereby lowering the enthalpic barrier. Meanwhile, the polyhydroxy quaternary ammonium structure provides more accessible orientations for water molecules near the pore entrance, thereby reducing the entropic penalty for water clusters entering the confined environment. As a result, the overall transport velocity of water clusters is higher in QBTS nanochannel—although that inside the PIP-TMC and QBTS are similar due to the identical internal environments (Fig. 5g). Our results indicate that the pore entrance functionalized with QBTS functional groups can promote fast transmembrane transport of water molecules by providing additional "acceleration" due to hydration competition, further reducing water transport resistance, and improving water flux.

SI-Line 447 Method for estimating the entropic penalty at the pore entrance

Following the method proposed by Jeffrey C. Grossman, we estimated the change in the entropic penalty for water molecules at the entrances of the PIP-TMC and QBTS-1 pores. Specifically, under the assumption that water transport through the nanopores follows Arrhenius model, the transport rate of water molecules can be written as follows:

$$k = A_0 exp\left[-\frac{\Delta E + T\Delta S - \Omega P}{RT}\right]$$

Here, k denotes the transport rate of water molecules, which was estimated from the water displacement–time curves shown in Fig. 5g, with the region from 92 to 80 Å defined as the pore-entrance vicinity. A_0 is the pre-exponential factor. Ω represents the effective molecular volume, and P is the applied pressure; the product ΩP is regarded as the driving force for species transport through the pore. ΔE is the enthalpic barrier for species translocation through the pore, ΔS is the entropic barrier for species translocation through the pore, R is the ideal gas constant, and T is the absolute temperature in Kelvin.

By taking the ratio of the transport rates of water molecules through the two pore entrances, the ratio of the transport rates through the two pore entrances can be simplified as:

$$RTln\frac{k_{QBTS}}{k_{PIP-TMC}} = -T\left(\Delta S_{QBTS} - \Delta S_{PIP-TMC}\right)$$

where k_{QBTS} and $k_{PIP\text{-}TMC}$ denote the transport rates of water molecules in the vicinity of the pore entrances of the QBTS and PIP-TMC channels, respectively. ΔS_{QBTS} and $\Delta S_{PIP\text{-}TMC}$ represent the entropic barriers for water molecules passing through the pore-entrance regions of the QBTS and PIP-TMC channels, respectively. $-T(\Delta S_{QBTS}-\Delta S_{PIP\text{-}TMC})$ corresponds to the reduction in the entropic barrier at the QBTS pore entrance relative to that of the PIP-TMC channel.

The results show that compared with the PIP-TMC pore, the effective entropic penalty near the pore entrance of the QBTS pore is reduced by approximately 0.799 kcal mol^{-1}. This value is comparable to the entropic barrier change calculated using the Eyring equation from transition state theory, indicating that QBTS plays a significant entropic modulation role in the local water entry process at the pore entrance. This result is also consistent with our hypothesis that the polyhydroxy structure can provide more orientations for water molecules during transmembrane transport, thereby helping to reduce the entropic penalty of the water cluster transmembrane process and consequently promoting water transport.

References:

1. Fang C, Yu Z, Qiao R. Impact of Surface Ionization on Water Transport and Salt Leakage through Graphene Oxide Membranes. *The Journal of Physical Chemistry C* **121**, 13412-13420 (2017).

2. Corry B. Water and ion transport through functionalised carbon nanotubes: implications for desalination technology. *Energy & Environmental Science* **4**, (2011).

3. Qiu R, *et al.* Effects of Edge Functional Groups on Water Transport in Graphene Oxide Membranes. *ACS Appl Mater Interfaces* **11**, 8483-8491 (2019).

4. Hughes ZE, Shearer CJ, Shapter J, Gale JD. Simulation of Water Transport Through Functionalized Single-Walled Carbon Nanotubes (SWCNTs). *The Journal of Physical Chemistry C* **116**, 24943-24953 (2012).

5. Suk ME. Single-File Water Flux Through Two-Dimensional Nanoporous Membranes. *Nanoscale Res Lett* **15**, 204 (2020).

6. Tunuguntla RH, Henley RY, Yao Y-C, Pham TA, Wanunu M, Noy A. Enhanced water permeability and tunable ion selectivity in subnanometer carbon nanotube porins. *Science* **357**, 792-796 (2017).

7. Cohen-Tanugi D, Grossman JC. Water Desalination across Nanoporous Graphene. *Nano Letters* **12**, 3602-3608 (2012).

8. Andrei IM, Chen W, Baaden M, Vincent SP, Barboiu M. Proton- versus Cation-Selective Transport of Saccharide Rim-Appended Pillar[5]arene Artificial Water Channels. *J Am Chem Soc* **145**, 21904-21914 (2023).

9. Jafarzadeh R, Azamat J, Erfan-Niya H, Hosseini M. Molecular insights into effective water desalination through functionalized nanoporous boron nitride nanosheet membranes. *Applied Surface Science* **471**, 921-928 (2019).

10. Fan J, Pan Y, Wang H, Song F. Efficient reverse osmosis-based desalination using functionalized graphene oxide nanopores. *Applied Surface Science* **674**, (2024).

11. Liu G, *et al.* New inspirations for efficient water transport in sub-nanopores: The effect of positive charges at the pore entrance. *Journal of Environmental Chemical Engineering* **13**, (2025).

12. Wen Q, *et al.* Engineering a Local Free Water Enriched Microenvironment for Surpassing Platinum Hydrogen Evolution Activity. *Angewandte Chemie International Edition* **61**, e202206077 (2022).

13. Wu L, Huang W, Li D, Zhao B, Zhou H, Luo W. Role of Interfacial Water in Improving the Activity and Stability of Lattice-Oxygen-Mediated Acidic Oxygen Evolution on RuO2. *Angewandte Chemie International Edition* **64**, e202420848 (2025).

14. Zhu Y, Chen Q, Hao J, Jiao Y. Breaking hydrogen-bonds in aqueous electrolytes towards highly reversible zinc-ion batteries. *Journal of Materials Chemistry A* **12**, 20097-20106 (2024).

15. Park H, *et al.* Spectroscopic and computational insight into the intermolecular interactions between Zwitter-type ionic liquids and water molecules. *Chemphyschem : a European journal of chemical physics and physical chemistry* **11**, 1711-1717 (2010).

16. Mukhopadhyay A, Xantheas SS, Saykally RJ. The water dimer II: Theoretical investigations. *Chemical Physics Letters* **700**, 163-175 (2018).

17. Heiranian M, DuChanois RM, Ritt CL, Violet C, Elimelech M. Molecular Simulations to Elucidate Transport Phenomena in Polymeric Membranes. *Environmental Science & Technology* **56**, 3313-3323 (2022).

18. Zhang N, *et al.* Effect of Hydrogen-Bonding Interaction on the Arrangement and Dynamics of Water Confined in a Polyamide Membrane: A Molecular Dynamics Simulation. *The Journal of Physical Chemistry B* **122**, 4719-4728 (2018).

RESPONSE TO REVIEWERS' COMMENTS

Reviewer #1:

Reviewer: *The authors have addressed all my comments in the previous review. The manuscript is good for publication.*

Our Response: We sincerely thank the reviewers for their thorough review and recognition of our paper.

Reviewer #2:

<u>Reviewer</u>: *The authors have addressed most of the comments. Here are some minor suggestions.*

Our Response: We thank the reviewers for their thorough and insightful review of our work, and we also appreciate the opportunity they have given us once again to improve the quality of our paper.

We would like to kindly remind that in the point-by-point response, we have marked newly added text in blue, deleted text in red with a strikethrough, and placed all revision marks within grey text boxes. In the text, MS-Line refers to the line numbers in the Marked Manuscript, and SI-Line refers to the line numbers in the Marked Supplementary Information.

<u>Reviewer (Comment 1)</u>: *For the molecular dynamics simulations, the choice of force field parameters exerts a significant influence on the simulation results and may even lead to completely opposite conclusions. In this work, two different force fields, OPLS AA and GAFF2, were separately employed for simulations involving the graphene system, which is insufficiently rigorous to ensure the consistency, comparability, and reliability of the simulation methodology. For the same type of system (e.g., the functionalized graphene pore system in this study), it is recommended to use a unified force field to avoid uncontrollable errors arising from force field discrepancies. Although the authors argue that the force field mainly affects quantitative details rather than the overall physical trends and theoretical conclusions, this viewpoint still requires further validation. Therefore, I suggest that the authors repeat the validation of key conclusions using at least one additional independent force field to confirm the robustness of the findings. Alternatively, the rationality and accuracy of the selected force fields should be demonstrated by quantitative/qualitative consistency between simulation results and experimental data.*

Our Response: Thank you for the detailed comment. Indeed, as the reviewer pointed out, a consistent force field in MD simulations is crucial for robustly confirming the reliability of the simulation results, especially for the two similar functionalized graphene systems in this study. Following the reviewer's suggestion, we employed an additional OPLS-AA force field to re-analyze the HBs lifetime between QBTS and water molecules (detailed simulation information is provided below). As shown in Fig. 2f, we found that the HBs lifetime near QBTS calculated using the OPLS-AA force field is longer than that obtained with the previously used GAFF2 force field. However, after unifying the OPLS-AA force field, we still clearly reached the same conclusion as before—compared with water-water interactions, the HBs lifetime between QBTS and H_2O is longer. The H_2O-H_2O hydrogen bond lifetime calculated using OPLS-AA force field is also similar to

values reported in other studies [1, 2]. Therefore, the interaction between QBTS and water molecules is stronger, and the resulting HBs are more stable. This confirms that QBTS can preferentially "capture" water molecules from water clusters, further leading to the dissociation phenomenon. The other graphene-based simulations shown in Fig. 2 were already performed using OPLS-AA; therefore, all graphene-based simulations now use a consistent force field. We have replaced the results originally simulated with the GAFF2 force field with those obtained using the OPLS-AA force field to improve the robustness of the results for the same type of simulation system. The relevant text in the manuscript has also been revised accordingly.

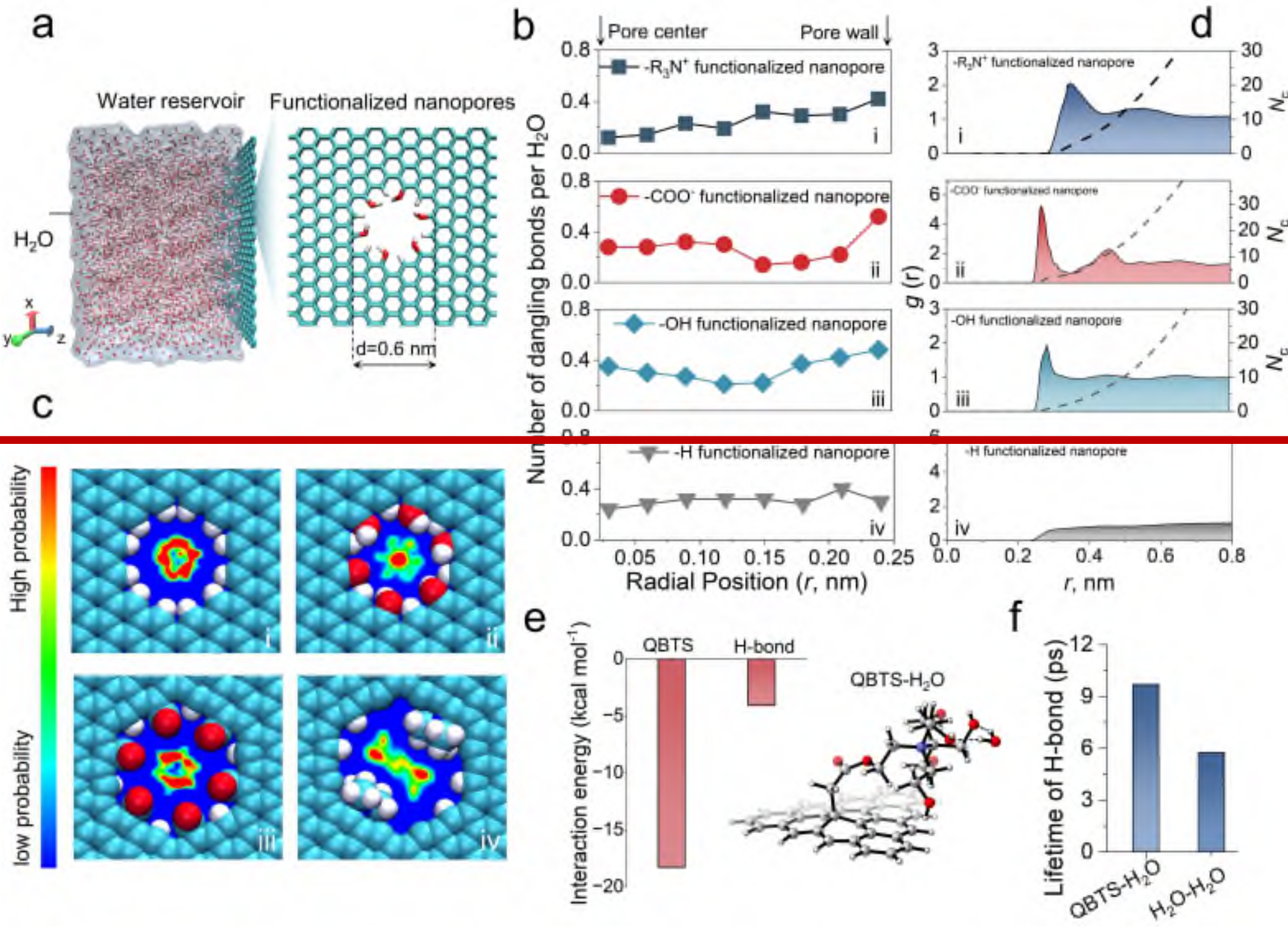

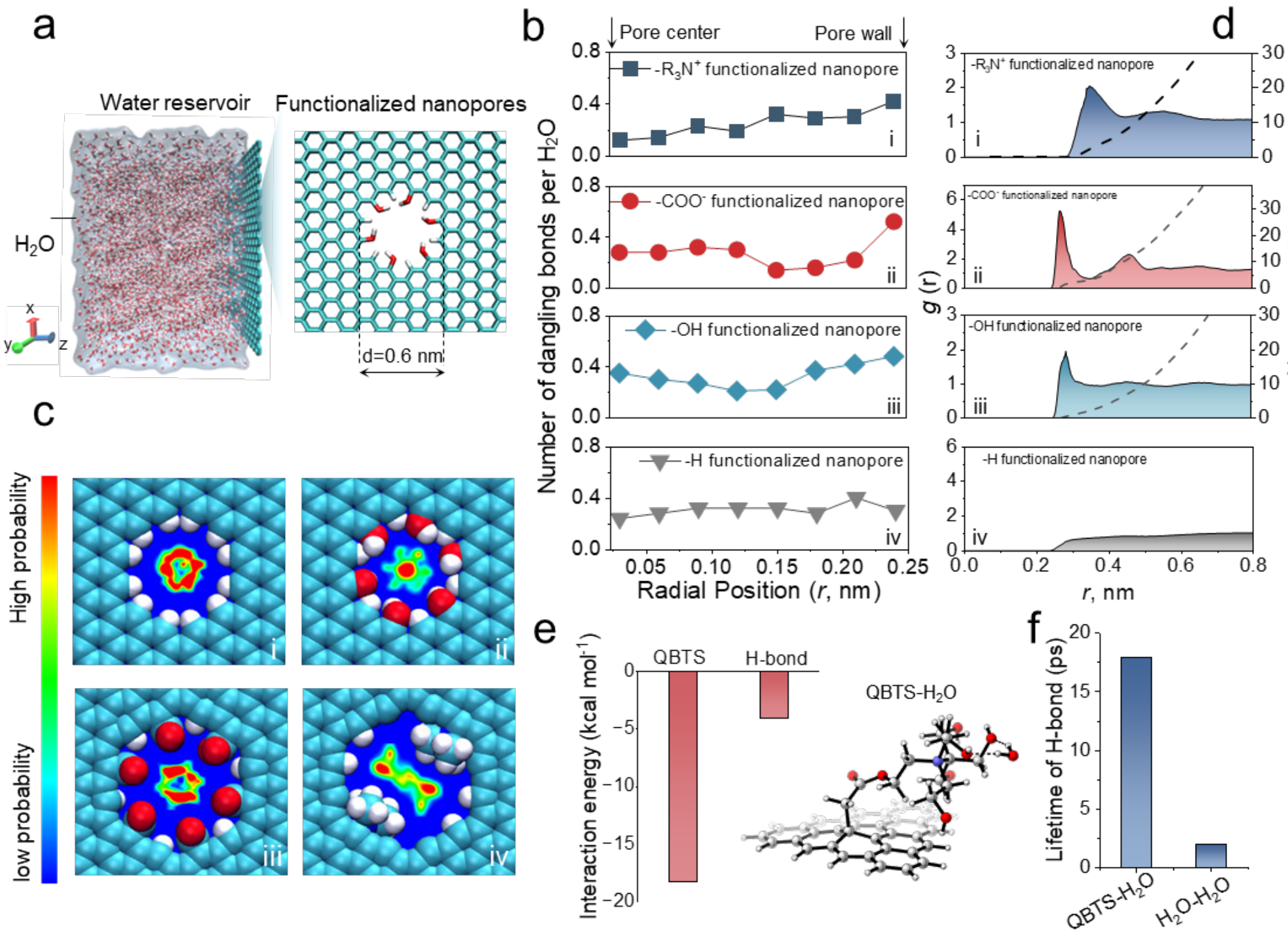

Fig. 2 Mechanisms of water cluster-dissociation from hydration competition by functional groups. (a) Side view of the MD simulation system investigated in this work. (b) Dangling bond radial distribution of water clusters in different functionalized pores. (c) Oxygen density maps in different functionalized pores, i-iv represents -H, -OH, -COO^- and -R_3N^+ functionalized pores, respectively. (d) RDFs between water molecule and the functional groups, and the corresponding coordination numbers (N_c), i.e., N-O_w, O-H_w, and H-O_w for -R_3N^+, -COO^- and -OH, respectively. (e) The interaction energy between a single water molecule and the QBTS functional group. The inset shows the double-sites interaction between QBTS and water molecule. (f) The H-bond lifetime of QBTS-water and water-water calculating by MD simulation.

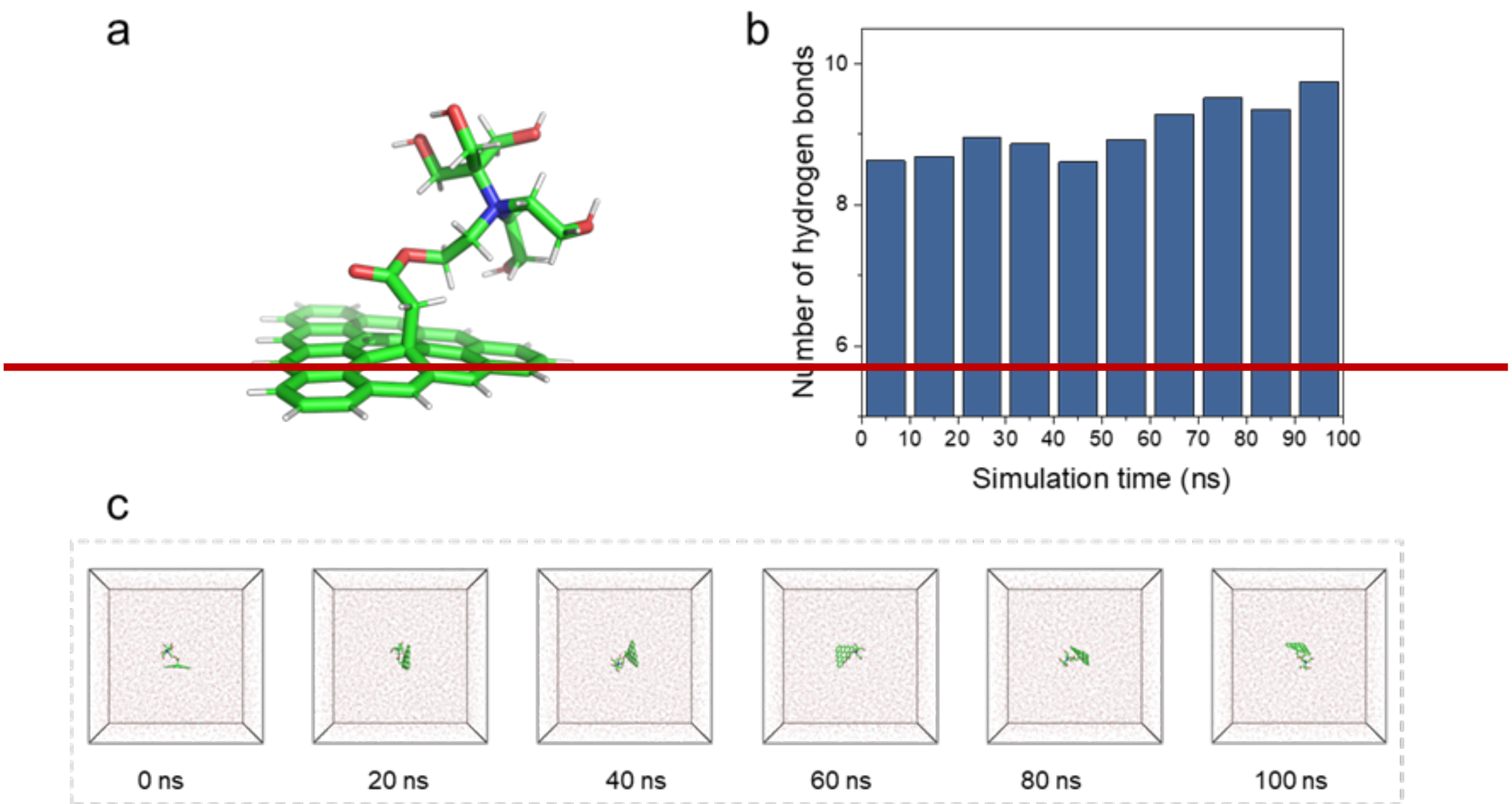

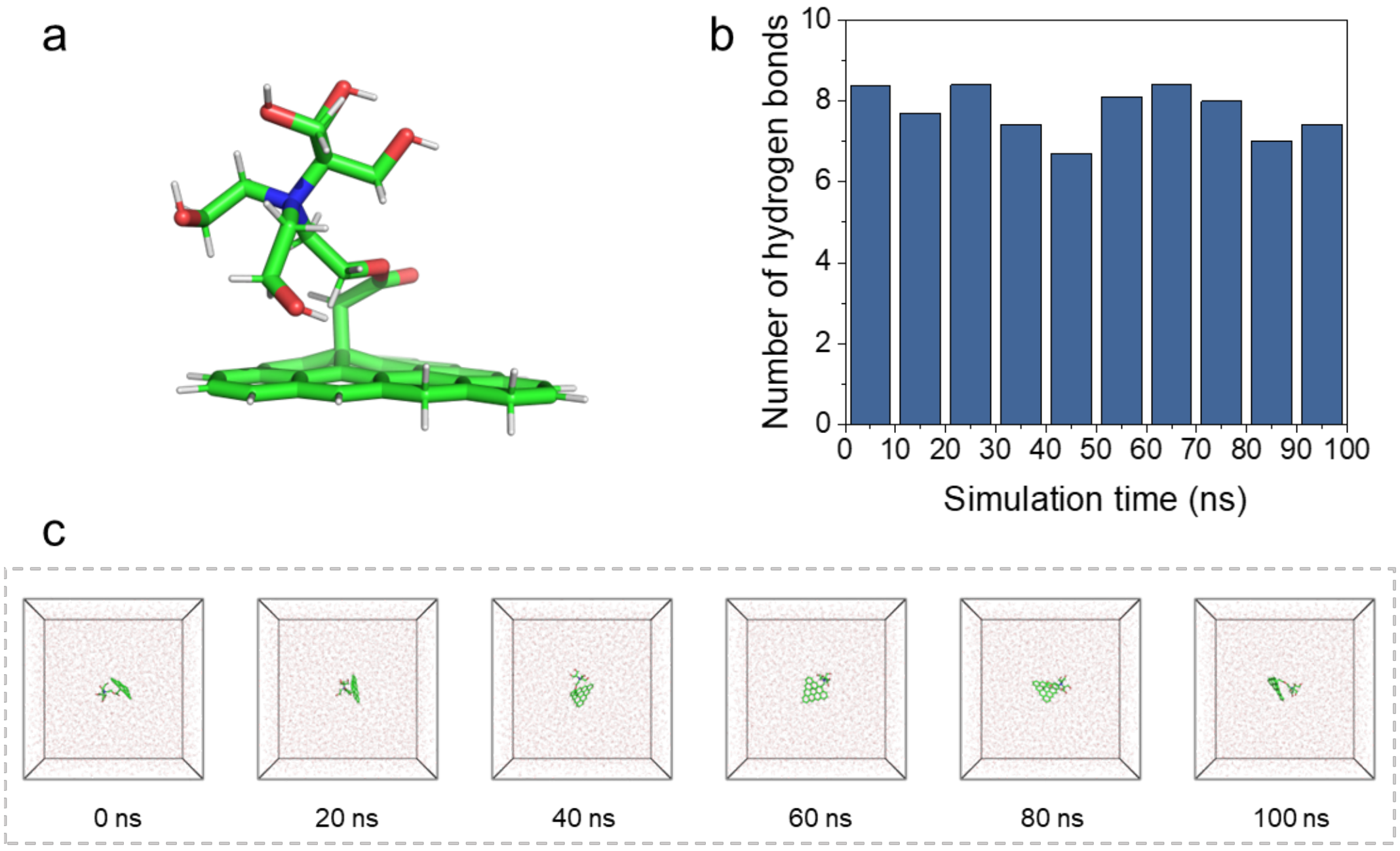

Fig S3. Simulation details for calculating H-bonds lifetime. (a) A graphene grafted by QBTS unit. (b) The variation of H-bonds number between QBTS and nearby water molecules as the simulation time increased. (c) The simulation aqueous box at different simulation time.

SI-Line 290 In this study, we did not use exactly the same force field across three independent and distinct MD simulations, namely: i) Hydration competition between functional groups and water molecules; ii) Confined transport of water molecules in different Nanochannels; and iii) calculation of H-bond lifetimes near QBTS. Because the specific sub-questions addressed by these three MD simulations are different and the compositions of their models are not identical, we did not enforce a uniform force field across all three simulations. Instead, we selected a more appropriate force field for each specific system—as has been reported in many other studies. Specifically, for the two similar simulation systems based on functionalized graphene, namely i) and iii), we used a consistent OPLS-AA force field, whereas for the polyamide nanochannel simulation system, i.e., ii), we chose the PCFF-INTERFACE force field.

SI-Line 413 ***Hydrogen bond characteristic simulation between functional groups and water molecules.*** ~~RESP charges were fitted using MultiWFN, and GAFF2 force field parameters for the corresponding molecules were generated using Sobtop. GAFF2 is an improved version of GAFF (General Amber Force Field), specifically designed to cover a broad range of organic chemical space, including well-parameterized oxygen-containing functional groups, nitrogen-containing groups, and aromatic systems[61-63]. Molecular dynamics simulations were performed using the Gromacs 2021 software. Gromacs is a widely used open-source molecular dynamics simulation software, primarily employed for simulating biomolecular systems such as proteins, lipids, nucleic acids, etc., as well as various molecular systems in materials science[34]. The force field used in this simulation was GAFF2, and the water model was TIP3P. A simulation box of 6 × 6 × 6 nm^3 was constructed, with the multi-hydroxy quaternary ammonium group-modified graphene molecule placed in the center of the box, and the box was filled with water. Na^+ and Cl^- were used to balance the system charge, rendering the overall system electrically neutral. Prior to simulation, energy minimization was first performed to eliminate unreasonable contacts in the initial conformation. This was followed by a 100 ps NVT pre-equilibration simulation. The NVT ensemble refers to a thermodynamic ensemble that maintains constant particle number (N), volume (V), and temperature (T) during the simulation, used to raise the temperature to the target simulation temperature. Subsequently, a 100 ps NPT pre-equilibration simulation was executed. The NPT ensemble refers to a thermodynamic ensemble that maintains constant particle number (N), pressure (P), and temperature (T) during the simulation, used to adjust the system density to the equilibrium value at the target pressure. After system relaxation, the production simulation was carried out. The production simulation used the leap-frog algorithm to integrate Newton's equations of motion, with an integration time step of 2 fs and a total of 50,000,000 integration steps, resulting in a total simulation time of 100.0 ns. The production simulation employed the V-rescale temperature coupling method, with a simulation temperature of 298.15 K. The production simulation used the Parrinello-Rahman pressure coupling method, with a simulation pressure of 1.0 bar. Due to the use of a cutoff distance method, the Particle Mesh Ewald (PME) method was applied to correct for long-range electrostatic interactions. Also due to the use of a cutoff distance method, dispersion correction was applied for long-range van der Waals interactions. Periodic boundary conditions were applied in all three directions throughout the simulation.~~

SI-Line 413 ***Hydrogen bond characteristic simulation between functional groups and water molecules.*** Molecular dynamics simulations were performed using the GROMACS software. The force field was chosen to be OPLS-AA, consistent with the simulation system for functionalized graphene nanopores. The simulation box was filled with water using the TIP3P water model. Cl^- ions were added to neutralize the system charge, ensuring overall charge neutrality. Prior to the production simulations, energy minimization was performed to eliminate unreasonable contacts in the initial conformation. Subsequently, 100 ps of pre-equilibration simulation in the NVT ensemble and 100 ps of pre-equilibration simulation in the NPT ensemble were carried out. After the system was fully relaxed, production simulations were performed. The production simulations employed the leap-frog algorithm to integrate Newton's equations of motion, with a time step of 0.002 ps and a total of 50,000,000 steps, resulting in a total simulation time of 100.0 ns. Due to the use of a cutoff distance for short-range interactions, long-range electrostatic interactions were corrected using the PME method. For long-range van der Waals interactions, a dispersion correction was applied. Periodic boundary conditions were applied in all three directions.

Next, following the reviewer's suggestion, we carefully compared the simulation results with the experimental results to further demonstrate the robustness and physical relevance of the simulation results in this work.

For the simulations related to functionalized graphene nanopores, i.e., Fig. 2a, Fig. S2 and Fig. S3, we first analyzed the fluctuation of dangling bonds (DBs) near the functional groups using the radial fluctuation amplitude (i.e., standard deviation, σ_{DB}):

$$\sigma_{\mathrm{DB}} = \sqrt{\frac{1}{N}\sum_{i}^{n}(DB_{r_\mathrm{i}} - \overline{DB})^2}$$

The results showed that the σ_{DB} of the -H nanopore was 0.044, while those of the -OH, $-COO^-$, and $-R_3N^+$ nanopores were 0.089, 0.110, and 0.094, respectively, indicating that the -OH, $-COO^-$, and $-R_3N^+$ can reconstruct the hydrogen-bond network of water molecules near the pore entrance. A marked increase in HBs breaking was also observed near the functional groups (i.e., increased DBs values, Fig. 2b). In addition, semi-quantitative statistics of the oxygen density maps (Fig. 2c) were performed using ImageJ software. Using the water-accessible area of the -H nanopore as a reference, we found that the normalized water-occupied areas at the nanopore entrance of the -OH (1.080), $-COO^-$ (1.051), and $-R_3N^+$ (1.050) nanopores were all higher than that of the -H control nanopore, suggesting that the functional groups at the pore entrance can expand or reconstruct the spatial distribution of water molecules within the pore cross-section. In our experiment, as shown in Fig. 5b, using *in situ* liquid ToF-SIMS, we observed that after grafting QBTS near the pores of the polyamide membrane, water clusters transitioned from $(H_2O)_5H^+$ into smaller $(H_2O)_3H^+$, indicating that QBTS indeed facilitate the

dissociation of water clusters. This trend is consistent with the simulation results described above. Meanwhile, using transition state theory, we experimentally determined the entropic energy barrier ($-T\Delta S^{\ddagger}$) for water cluster transmembrane transport. The results showed smaller $-T\Delta S^{\ddagger}$ for water clusters transport through QBTS-1 compared to PIP-TMC (Fig. 5c). This suggests that water clusters undergo less entropy loss when permeating through the QBTS-1 membrane, indicating that grafted QBTS at the pore entrance of the polyamide membrane endowed water molecules a relatively higher degree of freedom during partitioning into membrane[3]. Such experimental observation also supports the simulation findings of increased DB values (i.e., more "free" water molecule, Fig. 2b) and the "pulling" effect of functional groups on water clusters (Fig. 2c).

For the simulations related to polyamide nanochannels, i.e., Fig. 5e, it was similarly found that after QBTS modification, the HBs number of water clusters near the pore entrance decreased (Fig. 5f), which is consistent with the trend observed by *in situ* liquid ToF-SIMS—water clusters shifted from $(H_2O)_5H^+$ into smaller $(H_2O)_3H^+$ (Fig. 5b). Meanwhile, the MD simulation results showed that water clusters exhibit a faster transport rate near the pore entrance of QBTS-modified nanochannels (approximately three times faster than that in PIP-TMC nanochannels, Fig. 5g), which numerically supports the experimentally observed higher water permeance of QBTS-1 compared with pristine membrane (Fig. 4a). Furthermore, MD simulations revealed a lower PMF for water cluster transport through the QBTS nanochannels (Fig. S26), a result consistent with the lower enthalpic barrier (Fig. 5c) and activation energy (Fig. S22) experimentally determined by transition state theory. Finally, MD simulations also observed a lower PMF for Cl^- transport through the QBTS nanochannels (Fig. 4f), which is in agreement with the lower rejection rates of $MgCl_2$, $CaCl_2$, NaCl, and LiCl observed experimentally for QBTS-1 (Fig. 4d).

On the other hand, we have also conducted further literature review. Although different simulation details may have been adopted, previous simulation studies have also observed similar effects of functional groups on water clusters. For example, Kueh et al. found that hydroxyl groups in hydroxyl-functionalized graphene nanosheets can disrupt the hydrogen-bond network between water molecules and promote water transport and evaporation[4]. Lee et al. further demonstrated in oxygen-functionalized angstrom-scale graphene nanopores that oxygen-containing pore edges reduce water-water hydrogen bonds and shorten water-water hydrogen-bond lifetimes[5], thereby endowing water molecules with faster rotational and translational dynamics. Willcox and Kim also pointed out that hydroxyl groups on adjacent graphene surfaces perturb water structure and form a more intermittent water-water hydrogen-bond network[6]. These findings are consistent with our observations of functional group-induced enhancement of radial DB fluctuations and perturbation of the local water-water hydrogen-bond network. Cohen et al used water-oxygen density maps to analyze the spatial distribution of

water molecules within hydrogenated and hydroxylated graphene nanopores, and showed that the water transport area of hydrogenated pores is reduced by approximately 25% compared to hydroxylated pores; they further indicated that hydroxyl pore edges promote water flow by forming hydrogen bonds with water and providing a smoother entropic landscape[7]. This aligns with our use of oxygen density maps to quantify normalized water-occupied areas, and our finding that functional groups can expand/reconstruct the water distribution region within the pores.

In summary, these findings indicate that the MD simulation results presented in this study qualitatively and/or quantitatively support the experimental conclusions in four aspects: i) QBTS-functionalized pore entrance can dissociate or reconstruct the water clusters, corresponding to the increased proportion of water clusters with lower hydration numbers observed in TOF-SIMS and the decreased entropic energy barrier determined by transition state theory; ii) the enhanced transport rate of water clusters at the pore entrance of QBTS-functionalized nanochannel corresponds to the experimentally measured increase in water permeance; iii) the reduced PMF for water clusters crossing QBTS-functionalized nanochannels aligns with the experimentally observed decrease in energy barrier and higher water permeance; and iv) the decreased PMF for Cl^- crossing QBTS-functionalized nanochannels is consistent with the observed lower rejection of $MgCl_2$, $CaCl_2$, NaCl, and LiCl by QBTS-1. These findings demonstrate that the water/ion transport trends obtained from MD simulations corroborate the experimental observations, thereby supporting the rationality of the simulation model and force field parameters selected in this study for interpreting the key transport mechanisms. We have also added clarifying statements in the relevant sections to strengthen the connection between the simulation results and the experimental data.

MS-Line 411 Using the inhomogeneous solubility-diffusion model, the calculated permeability coefficient across the entire membrane increased correspondingly from 1.10×10^{-13} to 1.20×10^{-10} cm s^{-1}, indicating that Cl^- is more easily transported in QBTS. These simulation results are consistent with the experimentally observed lower rejection of $MgCl_2$, $CaCl_2$, NaCl, and LiCl by QBTS-1.

MS-Line 481 More importantly, in the h_{H_2O} after filtration through the QBTS-functionalized membrane, this increase in the proportion of $(H_2O)_3H^+$ is more pronounced compared with that of TMC-PIP membrane, as the proportion of $(H_2O)_5H^+$ decreased from 0.24 to 0.15, while that of $(H_2O)_3H^+$ increased from 0.30 to 0.46 (Fig. 5b). These results are consistent with the observation of more DBs near the functional groups in Fig. 2b.

MS-Line 515 Additionally, water molecules experience a lower entropic barrier when crossing the QBTS-1 membrane, suggesting a higher degree of freedom during permeation, which further facilitates their transmembrane transport (Fig. 5c). Using the migration data of water molecules in different nanochannels from molecular dynamics simulations (Fig. 5g), the entropic penalty calculated based on the simplified Arrhenius kinetic model further confirms that water diffusion in QBTS is entropically more favorable (*Supplementary Information*). After QBTS modification, water clusters show a lower entropic barrier and greater degrees of freedom, consistent with our MD observations of increased DBs near the functional groups and the expanding of water clusters.

MS-Line 559 Specifically, the water clusters can more rapidly enter the entrance of QBTS nanochannel, as their transport speed at the entrance of QBTS nanochannel is approximately three times that at the entrance of PIP-TMC nanochannel (Fig. 5g inset), which can be attributed to the additional "acceleration" to water clusters through energy compensation of hydration competition. This phenomenon supports the significantly enhanced water permeance observed in QBTS-1 (Fig. 4a).

MS-Line 567 As shown in the Fig. S26, the PMF experienced during water transport is lower after QBTS grafting, which also supports the observations of the lower enthalpic barrier (Fig. 5c) and favored water permeance in QBTS (Fig. 4a).

References:

1. Luzar A. Resolving the hydrogen bond dynamics conundrum. *The Journal of Chemical Physics* **113**, 10663-10675 (2000).

2. Paul S, Patey GN. Structure and Interaction in Aqueous Urea−Trimethylamine-N-oxide Solutions. *Journal of the American Chemical Society* **129**, 4476-4482 (2007).

3. Shefer I, Lopez K, Straub AP, Epsztein R. Applying transition-state theory to explore transport and selectivity in salt-rejecting membranes: a critical review. *Environmental Science & Technology*, (2022).

4. Kueh TC, Hung YM. Enhanced film-wise water evaporation through graphene nanostructures: A molecular dynamics insight. *International Journal of Heat and Mass Transfer* **233**, 126010 (2024).

5. Lee W-C, *et al.* Enhanced Water Evaporation from Å-Scale Graphene Nanopores. *ACS Nano* **16**, 15382-15396 (2022).

6. Willcox JAL, Kim HJ. Molecular Dynamics Study of Water Flow Across Multiple Layers of Pristine, Oxidized, and Mixed Regions of Graphene Oxide: Effect of Graphene Oxide Layer-to-Layer Distance. *The Journal of Physical Chemistry C* **121**, 23659-23668 (2017).

7. Cohen-Tanugi D, Grossman JC. Water Desalination across Nanoporous Graphene. *Nano Letters* **12**, 3602-3608 (2012).

Reviewer #3:

<u>**Reviewer**</u>**:** *The authors have satisfactorily addressed the requested revisions, so my recommendation is to publish this manuscript.*

> **Our Response:** Thank you for the thorough analysis of our work. We believe this work will be of great interest to the broad field of nanofluidics and membrane science.

Reviewer 3 Review Attachment 1

The authors have satisfactorily addressed the requested revisions, so my recommendation is to publish this manuscript.
