# Supplementary material for: Dissociating water clusters via polyhydroxy quaternized interface for enhanced water permeation in nanochannels
Source: Nat Commun. 2026 Aug 5;17:9435. doi: 10.1038/s41467-026-76291-z (PMC13538467; doi:10.1038/s41467-026-76291-z)
Supplement: Supplementary file 3 [file 41467_2026_76291_MOESM3_ESM.pdf]

nature portfolio

Corresponding author(s): NCOMMS-25-89601-C

Last updated by author(s): July 2, 2026

Reporting Summary

Nature Portfolio wishes to improve the reproducibility of the work that we publish. This form provides structure for consistency and transparency in reporting. For further information on Nature Portfolio policies, see our Editorial Policies and the Editorial Policy Checklist.

Statistics

For all statistical analyses, confirm that the following items are present in the figure legend, table legend, main text, or Methods section.

n/a	Confirmed	
☐	☒	The exact sample size (n) for each experimental group/condition, given as a discrete number and unit of measurement
☐	☒	A statement on whether measurements were taken from distinct samples or whether the same sample was measured repeatedly
☒	☐	The statistical test(s) used AND whether they are one- or two-sided<br>*Only common tests should be described solely by name; describe more complex techniques in the Methods section.*
☒	☐	A description of all covariates tested
☒	☐	A description of any assumptions or corrections, such as tests of normality and adjustment for multiple comparisons
☒	☐	A full description of the statistical parameters including central tendency (e.g. means) or other basic estimates (e.g. regression coefficient) AND variation (e.g. standard deviation) or associated estimates of uncertainty (e.g. confidence intervals)
☒	☐	For null hypothesis testing, the test statistic (e.g. F, t, r) with confidence intervals, effect sizes, degrees of freedom and P value noted<br>*Give P values as exact values whenever suitable.*
☒	☐	For Bayesian analysis, information on the choice of priors and Markov chain Monte Carlo settings
☒	☐	For hierarchical and complex designs, identification of the appropriate level for tests and full reporting of outcomes
☒	☐	Estimates of effect sizes (e.g. Cohen's d, Pearson's r), indicating how they were calculated

Our web collection on statistics for biologists contains articles on many of the points above.

Software and code

Policy information about availability of computer code

Data collection	No custom code or software was used in this study.
Data analysis	No custom code or software was used in this study.

For manuscripts utilizing custom algorithms or software that are central to the research but not yet described in published literature, software must be made available to editors and reviewers. We strongly encourage code deposition in a community repository (e.g. GitHub). See the Nature Portfolio guidelines for submitting code & software for further information.

Data

Policy information about availability of data

All manuscripts must include a data availability statement. This statement should provide the following information, where applicable:

- Accession codes, unique identifiers, or web links for publicly available datasets
- A description of any restrictions on data availability
- For clinical datasets or third party data, please ensure that the statement adheres to our policy

The data supporting the findings of this work are available within the article and its Supplementary Information files. Source data are provided with this paper.

Research involving human participants, their data, or biological material

Policy information about studies with human participants or human data. See also policy information about sex, gender (identity/presentation), and sexual orientation and race, ethnicity and racism.

Reporting on sex and gender	This study does not involve human participants, their data, or biological material.
Reporting on race, ethnicity, or other socially relevant groupings	This study does not involve human participants, their data, or biological material.
Population characteristics	This study does not involve human participants, their data, or biological material.
Recruitment	This study does not involve human participants, their data, or biological material.
Ethics oversight	No ethics approval was required as this study does not involve human participants or related materials

Note that full information on the approval of the study protocol must also be provided in the manuscript.

Field-specific reporting

Please select the one below that is the best fit for your research. If you are not sure, read the appropriate sections before making your selection.

☐ Life sciences ☐ Behavioural & social sciences ☒ Ecological, evolutionary & environmental sciences

For a reference copy of the document with all sections, see nature.com/documents/nr-reporting-summary-flat.pdf

Ecological, evolutionary & environmental sciences study design

All studies must disclose on these points even when the disclosure is negative.

Study description	This study aimed to elucidate how interfacial functional groups regulate water-cluster structures and transmembrane water transport in sub-nanometer confined nanochannels, and to develop a high-permeability nanofiltration membrane accordingly. The study was based on a polyamide nanofiltration membrane prepared by interfacial polymerization of piperazine (PIP) and trimesoyl chloride (TMC), followed by introduction of a polyhydroxy quaternized interfacial functional layer. The design included comparisons among the unmodified control membrane, membranes modified with different monomers for mechanistic verification, and membranes modified with 1 wt%, 3 wt%, and 5 wt% QBTS for optimization. By combining membrane separation performance tests, characterization of the Physical and Chemical Properties of Membranes (SEM, AFM, surface zeta potential, water contact angle Test , etc), in situ liquid time-of-flight secondary ion mass spectrometry (ToF-SIMS), and molecular dynamics (MD) simulations, we investigated how hydroxyl (-OH) and quaternary ammonium (quaternary-N^+) groups at the pore entrance induced water-cluster dissociation, reduced transport energy barriers, and enhanced water permeance.
Research sample	The research samples were laboratory-fabricated polyamide nanofiltration membranes. The unmodified membrane was prepared by interfacial polymerization of piperazine (PIP) and trimesoyl chloride (TMC), whereas the modified membranes were obtained by introducing QBTS (N-(trimethylolmethane)-N,N,N-tris(2-hydroxyethyl) ammonium bromide) to construct a polyhydroxy quaternized interface on the membrane surface. In addition, membranes modified with different monomers were prepared for mechanistic verification. This sample system was selected because such nanofiltration membranes possess typical sub-nanometer confined pore structures and allow chemical regulation of functional groups at the pore entrance, making them suitable for investigating the relationship between water-cluster structural transformation and transmembrane water transport. For in situ ToF-SIMS and MD simulations, the objects of study were the water-cluster ionic species at the membrane/water interface and the corresponding model nanochannel systems, respectively.
Sampling strategy	Membrane samples were prepared under predefined conditions and subjected to performance testing and structural characterization under identical experimental conditions. The study included the unmodified control membrane, membranes modified with different monomers, and membranes modified with 1 wt%, 3 wt%, and 5 wt% QBTS. For each experiment, three membrane pieces prepared under the same conditions were tested, and all key measurements were repeated three times to verify reproducibility and reduce the influence of chance variation. Sample sizes were determined according to standard practice in membrane materials research to ensure consistency of membrane fabrication and reliability of the measurements.
Data collection	Data collection included membrane separation performance data, membrane structural and interfacial chemistry characterization data, and mechanistic analysis data. Membrane performance measurements mainly comprised water flux and salt rejection tests. All samples were tested under the same conditions, including an operating pressure of 6 bar, a solution temperature of 25 °C, and a salt concentration of 1000 ppm. In situ liquid ToF-SIMS was used to collect information on water-cluster ionic species at the membrane interface, and molecular dynamics simulations were used to obtain molecular-level evidence for water-cluster dissociation and transport behavior. All experimental and control groups were subjected to the same testing procedures and conditions, and the data were recorded and organized by the Lei Xu.
Timing and spatial scale	Data collection for this study was conducted in stages following membrane preparation, including membrane performance testing, structural characterization, and in-situ ToF-SIMS analysis. Prior to testing, all membrane samples were stored in deionized water at room temperature, and all analyses were performed within one week after membrane preparation. Each test was conducted according to established procedures following sample preparation, with parallel comparisons made between different membrane samples. The spatial scale of the study encompasses the membrane surface interface, the membrane pore entrances, and the

	interior of sub-nanometer confined channels; the mechanistic analysis focuses on the structural transformation of water clusters at the nano- and molecular scales and its impact on the macroscopic membrane permeability.
Data exclusions	No data were excluded from the analyses. All data that met the experimental quality-control criteria were included in the final analysis.
Reproducibility	To verify reproducibility, membrane samples were repeatedly fabricated under the same conditions, and key experiments were repeated independently. For each experiment, three membrane pieces prepared under the same conditions were tested, and all key measurements were repeated three times. Consistent trends were observed across repeated experiments, indicating that the membrane fabrication procedure was stable and that the measurements were reliable. The in situ ToF-SIMS results were further corroborated by MD simulations, which strengthened the robustness of the main conclusions. All repeated experiments successfully reproduced the main trends of the results.
Randomization	This study did not involve biological subjects or clinical participants; therefore, formal randomization was not directly applicable. Membrane samples were prepared according to predefined experimental schemes and tested under identical conditions. No specific artificial order was imposed during testing, and both the experimental and control groups were subjected to the same testing sequence and conditions to minimize systematic bias as much as possible.
Blinding	Blinding was not used in this study. Because the study involved material samples rather than human or animal subjects, and the primary results were derived from instrument-based measurements and simulation outputs, including membrane flux, salt rejection, ToF-SIMS signals, and MD simulation results, blinding was not considered relevant. To reduce subjective bias, data acquisition relied primarily on standardized experimental procedures and instrument-generated readouts rather than subjective assessment.

Did the study involve field work? ☐ Yes ☒ No

Reporting for specific materials, systems and methods

We require information from authors about some types of materials, experimental systems and methods used in many studies. Here, indicate whether each material, system or method listed is relevant to your study. If you are not sure if a list item applies to your research, read the appropriate section before selecting a response.

Materials & experimental systems

n/a	Involved in the study
☒	☐ Antibodies
☒	☐ Eukaryotic cell lines
☒	☐ Palaeontology and archaeology
☒	☐ Animals and other organisms
☒	☐ Clinical data
☒	☐ Dual use research of concern
☒	☐ Plants

Methods

n/a	Involved in the study
☒	☐ ChIP-seq
☒	☐ Flow cytometry
☒	☐ MRI-based neuroimaging

Plants

Seed stocks	Plants experiment was not involved in our study.
Novel plant genotypes	Plants experiment was not involved in our study.
Authentication	Plants experiment was not involved in our study.
